# Practical Route for Catalytic Ring-Opening Metathesis Polymerization

**DOI:** 10.1021/jacsau.2c00566

**Published:** 2022-12-02

**Authors:** Indradip Mandal, Andreas F. M. Kilbinger

**Affiliations:** Department of Chemistry, University of Fribourg, Chemin du Musée 9, 1700 Fribourg, Switzerland

**Keywords:** catalytic ROMP, chain transfer agents, styrene, regioselective metathesis, functional polymers

## Abstract

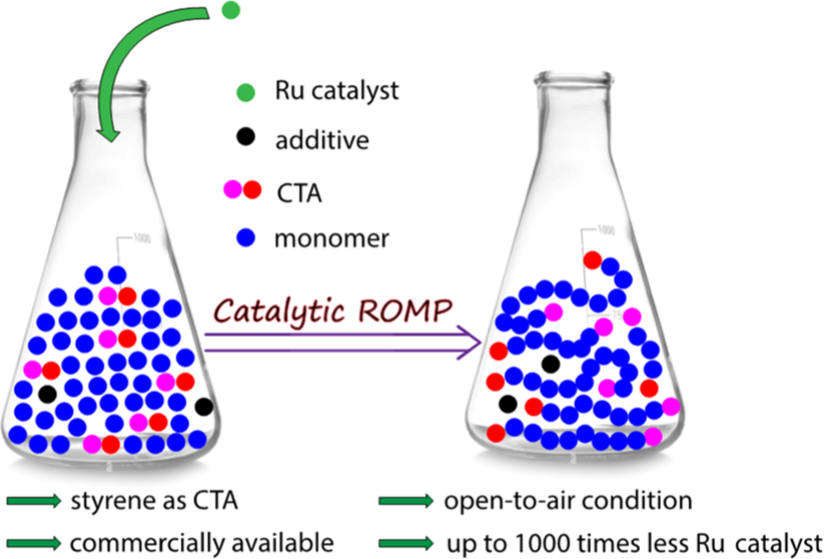

Norbornene derivatives are typical monomers for ring-opening metathesis polymerization (ROMP) for synthesizing highly functional polymers. However, the lack of catalytic methods, that is, the lack of readily available chain transfer agents (CTAs) for these monomers has been a significant cost limitation when large-scale syntheses are required. Here, we report commercially available styrene and its derivatives as efficient regioselective CTAs for the catalytic synthesis of metathesis polymers requiring up to 1000 times less ruthenium than in classical ROMP experiments. The molecular weight of the synthesized polymers was controlled by the monomer-to-CTA ratio. Low molecular weight ROMP polymers known for their antimicrobial properties were also synthesized on a gram scale in this report. Polymers were characterized by SEC, ^1^H NMR spectroscopy, and isotopically resolved MALDI-TOF MS. This approach describes a greener, more cost-effective, and eco-friendly methodology for the preparation of metathesis-based materials on the multigram scale.

## Introduction

**Scheme 1:**
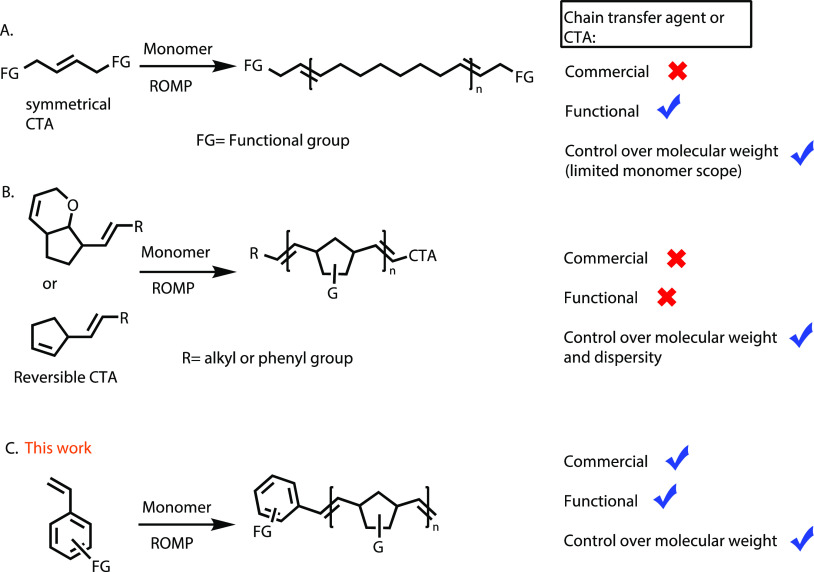
(A,B) Previously Reported CTAs for Metathesis Polymerization with Good Control Over Molecular Weight. (C) New Simple yet Effective CTAs (Styrene and Its Derivatives) for Synthesizing Large-Scale ROMP Polymers

The molecular weight control of polymers is a matter of immense interest as many of the physical and mechanical properties depend on chain length. Well-defined transition-metal-based metathesis catalysts discovered by Grubbs and Schrock allow the synthesis of highly functional and complex ring-opening metathesis polymers^1−5^ that showed applications in biology/medicinal chemistry,^6,7^ electronics,^8−11^ for membranes,^12^ and many others.^13−16^ The highly robust and functional group tolerant Grubbs second-generation (**G2**) and Grubbs third-generation (**G3**) catalysts are frequently used for polymerizations^17,18^ to obtain monotelechelic^19−25^ and heterotelechelic polymers^26−29^ in a controlled manner. In the conventional ring-opening metathesis polymerization (ROMP), excellent control of the polymer chain length could be achieved using those catalysts (as initiators) in stoichiometric amounts with respect to the number of polymer chains formed. For this reason, it is often challenging to synthesize metathesis-based polymers on a large scale, especially when shorter polymer chains are required. Catalytic methods for the preparation of ROMP polymers have been reported by Grubbs and co-workers using symmetrical chain transfer agents (CTAs).^30−32^ This method relies on “backbiting” to the polymer backbone to obtain homotelechelic polymers and is limited to very few monomers^33^ (such as cyclooctene or cyclooctadiene) (Scheme 1). Other methods to synthesize norbornene imide-based ROMP polymers using a sub-stoichiometric amount of ruthenium-based catalysts (Grubbs catalyst or Hoveyda–Grubbs catalyst) include pulsed addition of monomer,^34−36^ a degenerative reversible chain transfer mechanism (Scheme 1),^37,38^ or a kinetically controlled catalytic process.^39−43^ While each of these methods has proven to be helpful to achieve catalytic ROMP, there is no report of any functional, commercial, and inexpensive CTA that can be used to synthesize norbornene imide-based ROMP polymers on a multigram scale at low cost.

Herein, we report that styrene and its derivatives, in the presence of 3-bromopyridine, can be used as effective and regioselective CTAs to produce ROMP polymers catalytically via a kinetically controlled chain transfer mechanism. Unlike traditional ROMP polymerization methods, the procedures used in this report are simpler as no Schlenk line conditions were required to carry out the polymerizations.

## Results and Discussion

### Regioselective Cross-Metathesis

**Figure 1:**
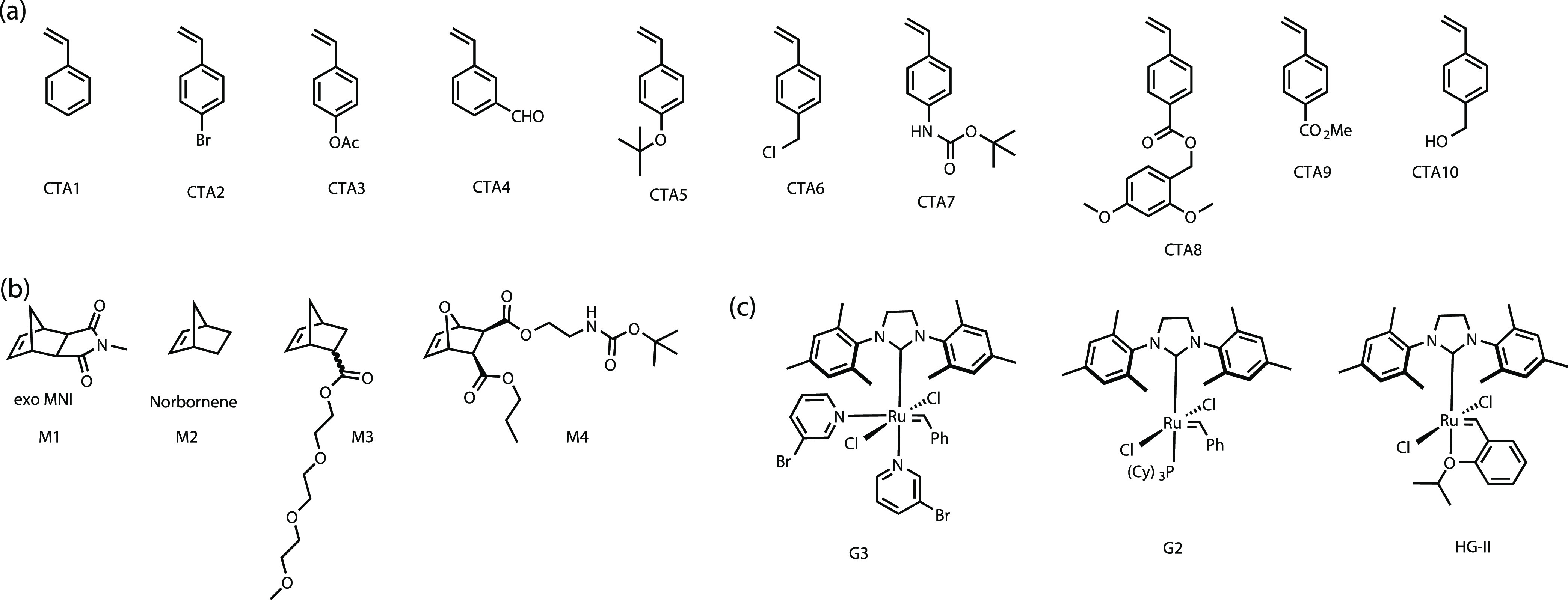
Chemical structures of compounds studied here. (a) Structures of CTAs. (b) Structures of monomers. (c) Structures of Ru-catalysts.

We recently showed that monosubstituted 1,3 diene derivatives could be used as regioselective CTAs in ROMP to produce monotelechelic polymers catalytically.^44^ The reaction of those derivatives with **G3** generates styrene as a side product in a catalytic amount. When we performed the polymerization using a 1,3-diene and **G3** in a ratio of 1:1, thus yielding 1 equiv of styrene, we observed a substantial reduction in the molecular weight of the synthesized polymer. We anticipated this could only happen due to chain transfer with styrene during polymerization. Thus, we performed an independent ^1^H NMR tube reaction mixing styrene (**CTA1**) (15 equiv) and **M1** (see Figure 1) (300 equiv) in chloroform-*d*. Then, 1 equiv of **G3** was added to the NMR tube and the ^1^H NMR spectrum was recorded immediately. To our surprise, complete consumption of **M1** (no triplet peak at 6.25 ppm) and >97% consumption of **CTA1** (quartet signal at 6.65 ppm) was observed within the first measurement (<5 min, see Figure S1). SEC analysis of the precipitated polymer (**P1**) showed an excellent agreement of the observed molecular weight (*M*_n,SEC(CHCl_3_)_ = 4.3 kDa) with that of the theoretical value (*M*_n,**M1**/**CTA1**_ = 3.6 kDa). MALDI-TOF MS analysis, on the other hand, showed a major distribution of peaks corresponding to polymer chains with a phenyl group on one end and methylene group on the other, along with two other minor distributions showing a mixture of chain ends (see Figure S2). Surprisingly, the addition of 3-bromopyridine (3BPY) as an additive to the polymerization mixture suppressed the non-regioselective chain transfer completely. A chain end-capping experiment was further performed where a **G3**-benzylidene initiated poly(*exo*-*N*-methyl norbornene imide) (**M1**:**G3** = 20), in the presence of 30 equiv of 3BPY, was terminated with 5 equiv of **CTA1** (see Figure S3). A ^1^H NMR measurement after 10 min showed that the propagating ruthenium carbene signal (**G3**-alkylidene, 18.55 ppm) had vanished, and a new signal for **G3**-benzylidene (19.15 ppm) was observed, suggesting a fast and regioselective chain transfer. Besides, this exceptionally fast cross-metathesis with **CTA1** could also be observed visually as the deep yellow color of **G3**-alkylidene immediately changed into green, which is the typical color of **G3**-benzylidene^45^ (see Figure S4). Furthermore, ^1^H NMR spectroscopic data and MALDI-TOF MS analysis of the precipitated polymer (**P2**) were in agreement with the proposed regioselective chain transfer (see Figures S4 and S5).

**Figure 2:**
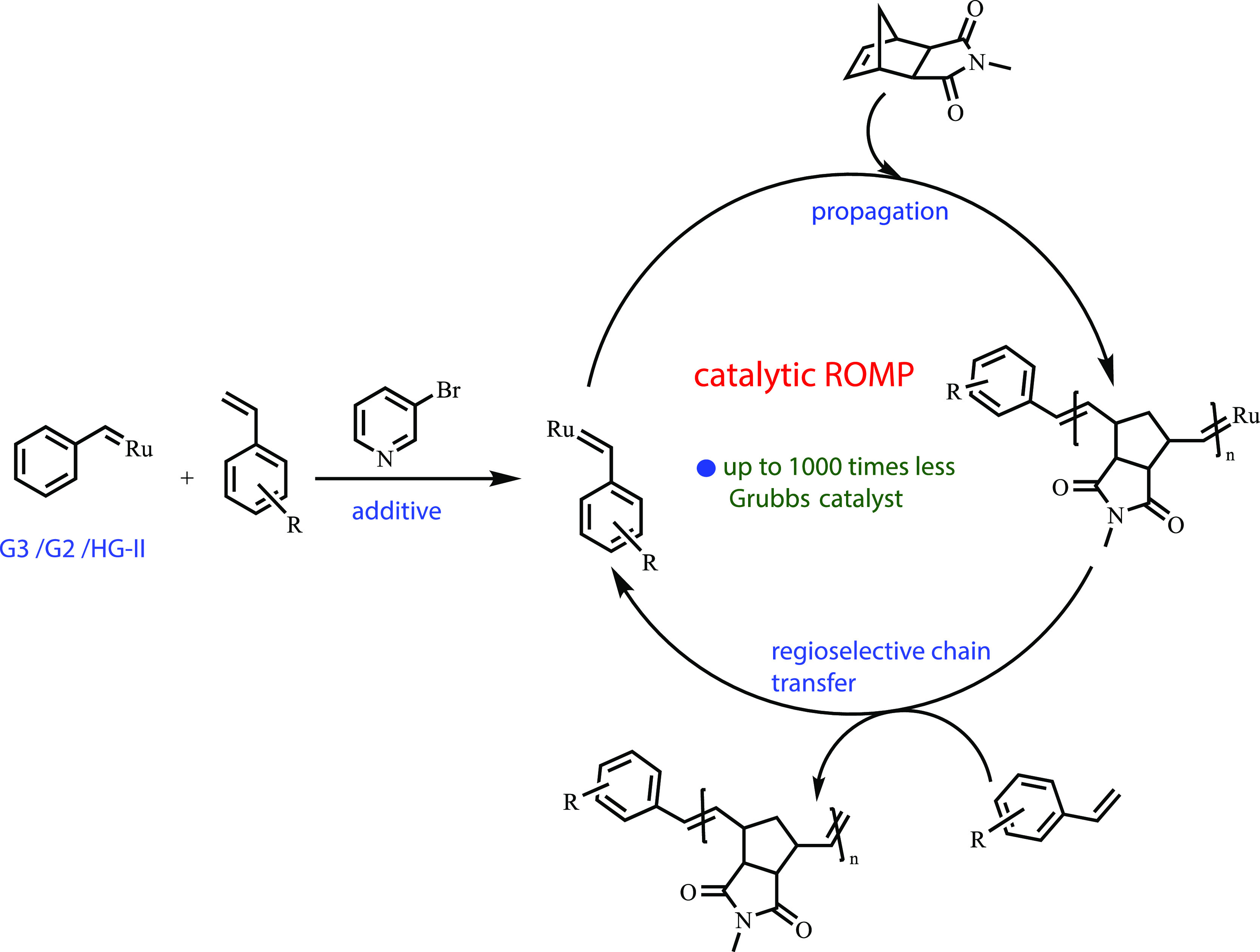
Mechanism for catalytic ROMP. First, the **G3**/**G2**/**HG-II** catalyst reacts with CTAs regioselectively in the presence of 3BPY. The functionalized catalyst reacts with monomer (here only **M1** is shown as an example) to form the propagating species, which reacts regioselectively with styrene derivatives to give back the functional catalyst closing the catalytic cycle.

**Figure 3:**
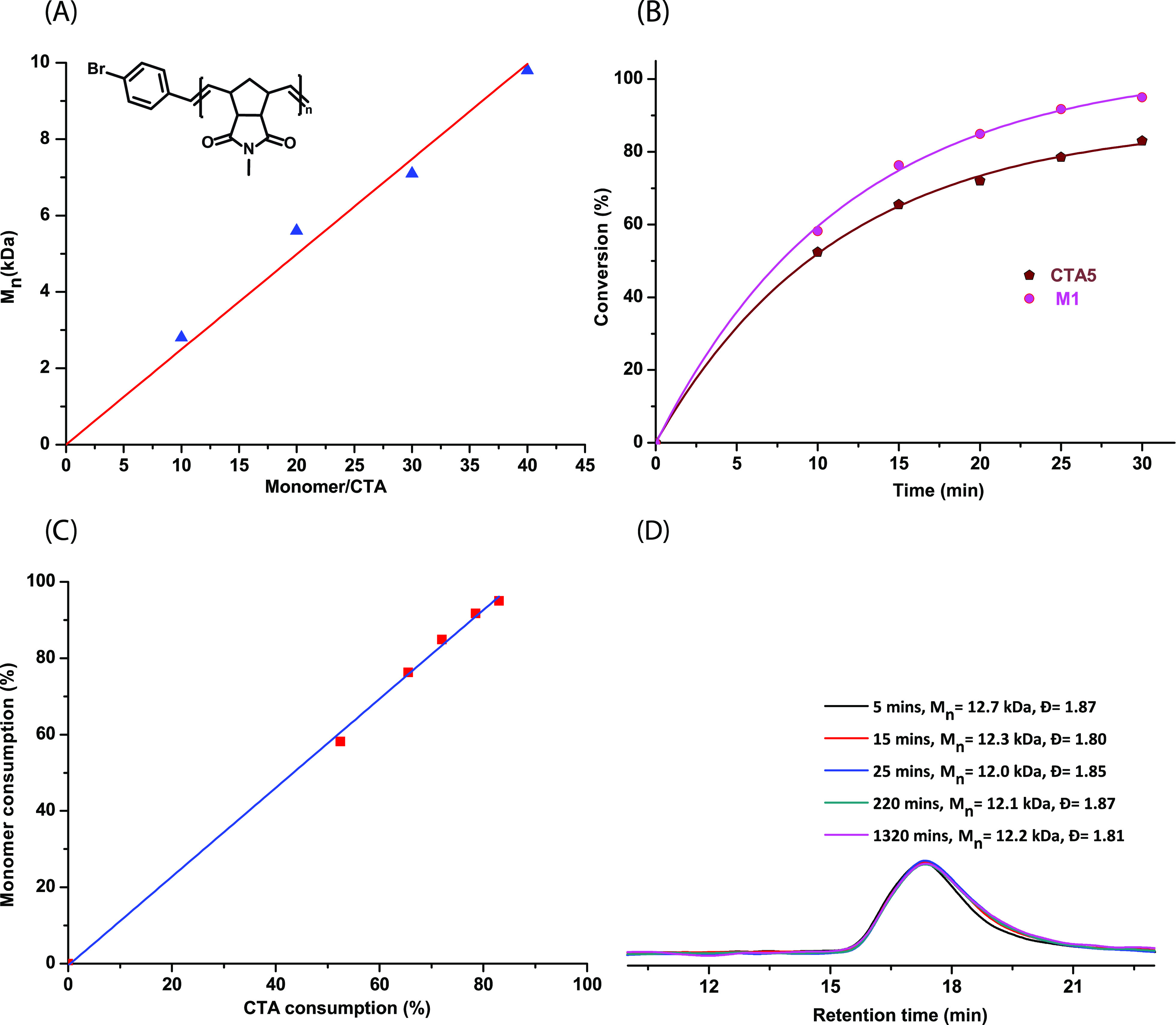
(A) Plot of the number average molecular weight (*M*_n_, measured by SEC in CHCl_3_) vs the monomer (**M1**) to CTA (**CTA2**) ratio shows a linear correlation. The polymerizations were carried out using a constant ratio of **G3**:3BPY:**CTA2** = 1:30:50 and varying **M1** accordingly. (B) Plot of monomer (**M1**) and **CTA5** conversion with time as determined by ^1^H NMR spectroscopy (CD_2_Cl_2_, 400 MHz) showing both were consumed with almost the same proportion throughout the polymerization. (C) In the same experiment, a plot of consumption of **M1** vs consumption of **CTA5** showed a linear relationship, further proving a kinetically controlled chain transfer mechanism. (D) Final proof for the kinetically controlled mechanism given by SEC measurements showed an almost constant *M*_n_ value over the whole polymerization time.

Having established the regioselective cross-metathesis of **CTA1** with the propagating Ru complex, we explored the applicability of other styrene derivatives. **CTA2-6** was commercially available, and **CTA7-10** was synthesized in a few straightforward steps (see the Supporting Information). **CTA2** was then utilized in a one-pot catalytic polymerization maintaining the ratio of **G3**:3BPY:**CTA2**:**M1** = 1:30:50:500. SEC analysis of the resulting polymer revealed a monomodal distribution with the molecular weight as determined by the **M1**:**CTA2** ratio (**P4**, *M*_n,**M1**/**CTA2**_ = 1.9 kDa vs *M*_n,SEC(CHCl_3_)_ = 2.8 kDa) and a dispersity of 1.84 suggesting a kinetically controlled mechanism (Figure 2). Different ratios of **M1**:**CTA2** were further investigated, and the resulting polymers showed a linear dependence between the number average molecular weight (determined by SEC in CHCl_3_) and the monomer to **CTA2** ratio (see Figure 3A). Unlike the conventional ROMP mechanism, in a catalytic ROMP, incomplete initiation of the Ru complex does not influence the target molecular weight of the polymers. Therefore, we used the cheaper and more stable **G2** and the Hoveyda–Grubbs catalyst (**HG-II**) to perform a polymerization under similar conditions. ^1^H NMR tube polymerization reactions using **CTA2** and **M1** with either **G2** or **HG-II** produced polymers **P8** (*M*_n,SEC(CHCl_3_)_ = 2.5 kDa with **G2**) and **P9** (*M*_n,SEC(CHCl_3_)_ = 2.4 kDa with **HG-II**) with excellent control over the molecular weight (see Table 1 and Figures S8 and S9). Next, **CTA3** was used under similar polymerization conditions with **M1** (**P10**, **G3**:**CTA3** = 1:100, *M*_n,SEC(CHCl_3_)_ = 5.3 kDa, Figure 5A) and **M3** (**P11**, **HG-II**:**CTA3** = 1:20, *M*_n,SEC(CHCl_3_)_ = 5.0 kDa) and **CTA4** was used with **M3** (**P12**, **HG-II**:**CTA4** = 1:100, *M*_n,SEC(CHCl_3_)_ = 8.5 kDa) to produce monotelechelic polymers catalytically. The purified polymers were fully characterized using ^1^H NMR spectroscopy and MALDI-TOF MS (see the Supporting Information).

**Table 1:** Catalytic Polymerization Data Using Functional Styrene Derivatives as CTAs

polymer	catalyst (Cat)	CTA	monomer (M)	Cat/CTA/M^a^	*M*_n_ (non-catalytic, M/Cat) kDa	*M*_n,theoretical_(catalytic, M/CTA) kDa	*M*_n,observed_ (SEC, CHCl_3_)^b^ kDa	dispersity (*D̵*)
P3	**G3**	**CTA1**	**M1**	1:5:60	10.7	2.2	2.6	1.71
P4	**G3**	**CTA2**	**M1**	1:50:500	88.5	1.9	2.8	1.84
P8	**G2**	**CTA2**	**M1**	1:20:200	35.4	1.9	2.5	2.20
P9	**HG-II**	**CTA2**	**M1**	1:20:200	35.4	1.9	2.4	2.15
P10	**G3**	**CTA3**	**M1**	1:100:2000	354	3.7	5.3	2.05
P12	**HG-II**	**CTA4**	**M3**	1:100:2000	569	5.8	8.5	2.07
P13	**G3**	**CTA5**	**M1**	1:20:200	35.5	1.9	3.0	1.86
P14	**G3**	**CTA5**	**M1**	1:20:1200	212.4	11	14.5	1.96
P15	**G2**	**CTA6**	**M1**	1:100:2000	569	6.0	7.0	1.72
P16	**G3**	**CTA7**	**M1**	1:100:3000	531	5.5	8.1	1.95
P17	**HG-II**	**CTA8**	**M1**	1:20:200	35.4	1.9	3.5	2.22
P18	**HG-II**	**CTA8**	**M1**	1:150:4500	797	5.6	10	2.10
P19	**G3**	**CTA9**	**M1**	1:20:200	35.6	1.9	3.0	2.00
P20	**HG-II**	**CTA10**	**M1**	1:20:200	35.4	1.9	3.0	1.93
P21	**G2**	**CTA6**	**M2**	1:200:6000	564	2.9	20	2.20
P22	**HG-II**	**CTA1**	**M1**	1:500:5000	900	1.8	3.0	1.72
P23	**HG-II**	**CTA1**	**M1**	1:1000:15,000	2655	2.7	4.0	1.80
P29	**G2**	**CTA11**	**M1**	1:20:400	71	3.7	5.4	1.96
P30	**G2**	**CTA12**	**M1**	1:20:400	71	3.7	4.9	2.08

a 3-Bromopyridine (3BPY) was used (typically 30 equiv) as an additive in the polymerization.

b SEC (CHCl_3_) was calibrated against poly(styrene) standards.

### Kinetic and Mechanistic Studies

To elucidate the proposed kinetically controlled mechanism (see Figure 2), we performed a ^1^H NMR kinetic analysis using **M1**, **CTA5**, and **G3**. The reagents were mixed in a ratio of 200:20:1, respectively, and the consumption of both, **CTA5** and **M1**, was followed by ^1^H NMR spectroscopy over time. As expected for a kinetically controlled mechanism, both reagents were consumed proportionately over the entire polymerization time (see Figure 3B).

Additionally, a plot of monomer versus **CTA5** consumption (see Figure 3C) showed a linear relationship, which should be the case for a kinetically controlled chain transfer polymerization. Further proof of the mechanism was obtained by following the polymerization (**G3**:3BPY:**CTA5**:**M1** = 1:30:40:2000) by SEC (via quenching aliquots at a different time intervals, Figure 3D) over time. An almost constant number average molecular weight (*M*_n_ = 12 kDa) was obtained over 1320 min suggesting that both, the rate of propagation and the rate of chain transfer with the CTA were similar, supporting once more a kinetically controlled mechanism. The exceptionally high rate of chain transfer and regioselectivity of styrene derivatives was unexpected because styrene has been utilized numerous times in the literature as a cross-metathesis partner or even in self-metathesis to produce stilbene derivatives.^45−48^ We believe that in the presence of 3BPY, the reactivity of the Ru–carbene complex is suppressed to a great extent,^49^ thus making it more selective to form Ru–benzylidene, which has kinetic and thermodynamic preferences over Ru–methylidene.^50^ It is noteworthy that in the SEC trace (see Figure 3D), even after 1320 min, no higher molecular weight shoulder peak was observed as the elugram maintained its monomodal distribution suggesting that no chain end coupled product was formed by the removal of ethylene. Furthermore, a detailed kinetic analysis was performed using ^1^H NMR spectroscopy to determine the rate constants for the consumption of both CTA and monomer during the kinetically controlled polymerization (see Figures S24–S28). Unlike our previous report,^44^ here, the rate constant for the consumption of **CTA2** was 1.53 times less than that of the monomer indicating a lower chain transfer rate constant in the case of styrenes compared to monosubstituted 1,3 dienes. Moreover, rate constants for both the chain transfer event and monomer propagation were determined using the Mayo equation.^51,52^ A plot of 1/DP (DP = degree of polymerization) versus CTA to monomer concentrations produced a linear relationship indicating that efficient chain transfer was involved (Figure S29). Additional kinetic information showed that the ratio of monomer propagation constant to chain transfer constant was 1.68 which, again suggesting that the rate constants of monomer propagation and chain transfer are of almost similar magnitude. This criterion should be fulfilled to achieve control over the molar mass of the polymers synthesized via a kinetically controlled chain transfer process. Our present study clearly shows a kinetically controlled chain transfer mechanism allowing us to obtain good to excellent control over the molecular weight of the synthesized polymers (see Table 1).

### Functional Catalytic ROMP

**M1** was further used to synthesize a library of highly end-functional ROMP polymers, maintaining a high CTA:Ru complex ratio. For instance, **P15** (**G2**:**CTA6** = 1:100, *M*_n,SEC(CHCl_3_)_ = 7.0 kDa), **P16** (**G3**:**CTA7** = 1:100, *M*_n,SEC(CHCl_3_)_ = 8.1 kDa), and **P18** (**HG-II**:**CTA8** = 1:150, *M*_n,SEC(CHCl_3_)_ = 10.0 kDa) (see Table 1) were synthesized under non-Schlenk conditions carrying an atom transfer radical polymerization initiator, a Boc-protected aromatic amine, or a DMB-protected aromatic acid group at one chain end. Both **P16** and **P18** were further deprotected using trifluoroacetic acid to give the free amine (**P24**) and acid functional (**P25**) ROMP polymers on a gram scale using at least 100 times less Grubbs catalyst than in a standard ROMP polymerization. These polymers were fully characterized by ^1^H NMR spectroscopy (see the Supporting Information) and MALDI-TOF MS (see Figure 5B,C). Benzylic alcohol functional **CTA10** was also utilized effectively using **HG-II** and **M1** to produce **P20**. The presence of the alcohol group was confirmed via MALDI-TOF MS. When a more reactive monomer, **M2**, was employed in our method using **CTA6**, poor control over molecular weight was observed (*M*_n,**M3**/**CTA6**_ = 2.9 kDa vs *M*_n,SEC(CHCl_3_)_ = 20.0 kDa). Nonetheless, the molecular weight calculated by the monomer to initiator ratio (assuming no chain transfer with **CTA6**) would have been as high as 564 kDa. A polymer of *M*_n_ = 20 kDa was obtained suggesting a substantial degree of chain transfer was involved.

**Figure 4:**
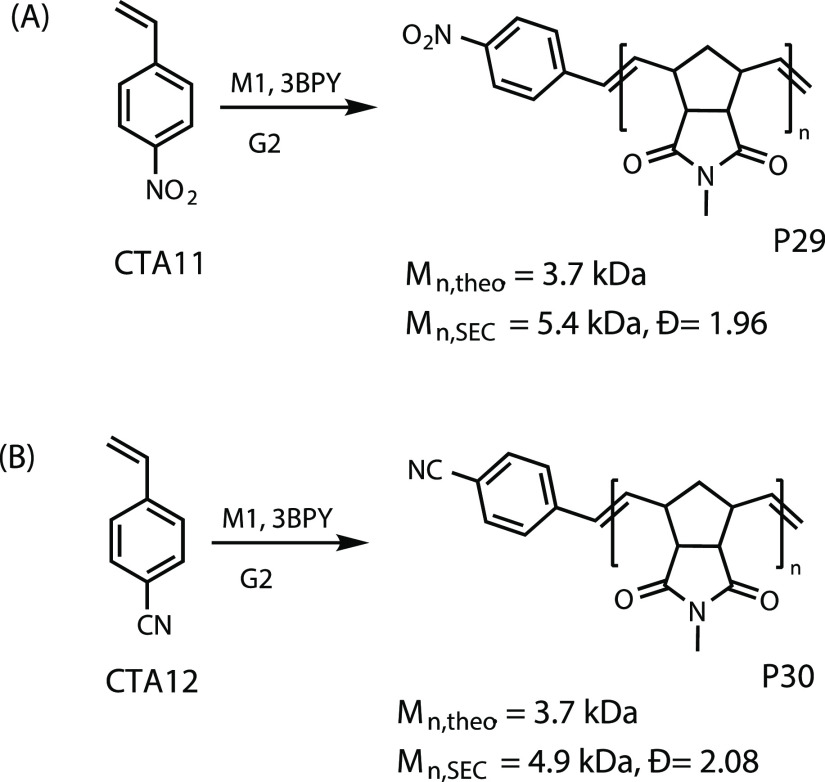
Controlled catalytic ROMP with highly electron-withdrawing CTAs.

Our recent study^44^ revealed that electron-rich CTAs are more suitable for catalytic ROMP, probably, due to higher chain transfer rates of Ru–carbene complexes in the case of electron-rich olefins. To illustrate the versatility of our method, we chose to test the applicability of highly electron deficient styrenes in our system. **CTA11** and **CTA12**, containing a nitro group and a cyano group at the para position, respectively, were studied using ^1^H NMR spectroscopy. In both cases, almost full consumption of monomer (>95%) was observed, whereas the CTA consumption was around 85%. It means even electron deficient styrenes could be used in our catalytic system (see Figure 4). Gratifyingly, these CTAs maintained same kind of regioselective chain transfer as observed before, as only one type of mass distribution was detected in MALDI-TOF mass spectra of the polymers (see Figures S94 and S95).

Slow monomer, such as, **endo-MNI** (**endo-M1**) was also studied for our catalytic ROMP. We found that due to slow reactivity of the endo isomer, catalyst decomposition competes with the propagation and chain transfer events, resulting in very low monomer conversion (35%) in our standard reaction conditions (Figure S21). When, the same polymerization was performed without 3BPY, full consumption of both CTA and monomer was observed (Figure S22), but MALDI-TOF MS showed mixture of chain ends indicating that non-regioselective chain transfer was involved (Figure S23).

Our catalytic ROMP method is ideal for synthesizing low molecular weight polymers as their established synthesis requires a very high loading of costly and toxic transition metal–carbene complex. This limits the application of those polymers where metal contamination could play a critical role. In 2008, the Tew group showed that oligomers of an oxanorbornene monomer (**M4**) could be used as potential antimicrobial ROMP polymers that have a very high selectivity toward bacteria cells over mammalian cells.^53^ The molecular weight of the synthesized oligomers proved to be an essential parameter in determining the selectivity. Only very short oligomers (degree of polymerization = 8) prevented further growth of bacteria cells and showed the essential selectivity. Due to the lack of any suitable CTAs, those antimicrobial ROMP polymers could only be synthesized on the milligram scale using a very high loading of the Grubbs initiator. Here, we were able to synthesize an oligomer of **M4** (**P27**, *M*_n,SEC(CHCl_3_)_ = 2.8 kDa) on a 1.3 g scale using only 0.07 mol % of the **HG-II** catalyst (150 times less than previously reported) to exemplify the potential of our method. The high chain-end fidelity of **P27** was established by ^1^H NMR spectroscopic and MALDI-TOF MS data (see Figures S74 and S93). Further deprotection using trifluoroacetic acid produced **P28** having the same backbone functionality (cationic amphiphilic groups) as shown by Tew et al. This methodology could further be explored in synthesizing many biologically useful functional ROMP polymers where high loading of toxic ruthenium metal has so far limited the applicability.

### Robust Catalytic ROMP

**Figure 5:**
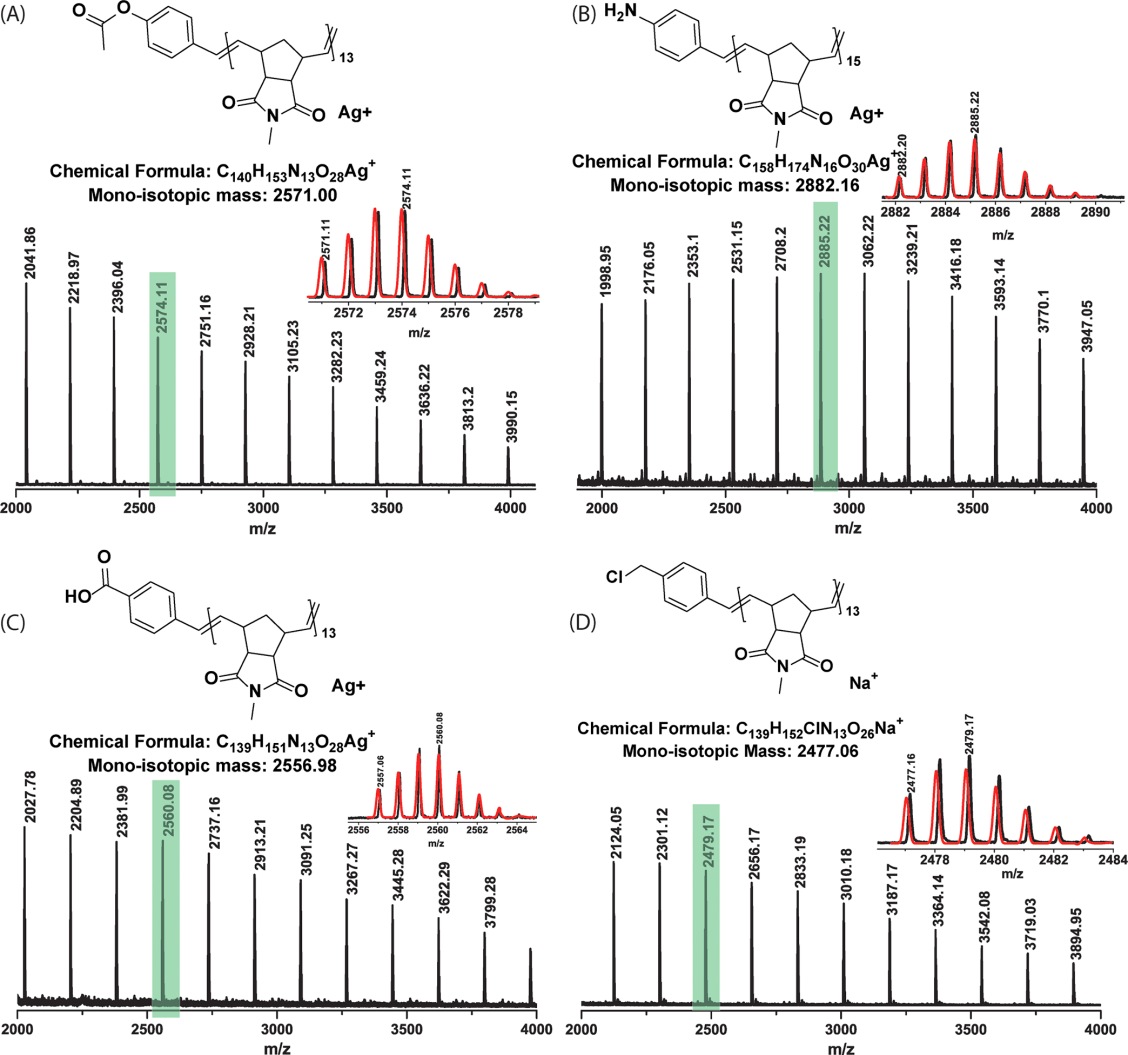
Isotopically resolved MALDI-TOF mass spectrum (DCTB, AgTFA/NaTFA) of the synthesized polymer (A) **P10**, (B) **P24**, (C) **P25**, and (D) **P26** matching the expected end groups. The zoomed mono-isotopic distributions (black lines) were compared with the simulated (red lines) spectrum in each case, providing a good agreement between the experimentally observed and the simulated mass distributions.

The mechanism of our catalytic method suggests that the resting state of the catalyst after chain transfer with a CTA is a Ru–benzylidene complex. This was also observed in the ^1^H NMR kinetics experiment (see Figures S8–S13). We, therefore, exploited the high stability of the benzylidene complex by showing that even technical grade dichloromethane could be used as a solvent, and the polymerization could be performed under non-degassed conditions in an open Erlenmeyer flask (see Figure S16). A ratio of **G2**:3BPY:**CTA6**:**M1** = 1:30:200:10,000 was used to obtain polymer **P26** (*M*_n,SEC(CHCl_3_)_ = 11.5 kDa) with 90% yield. MALDI-TOF analysis further proved the anticipated polymeric species (see Figure 5D).

Encouraged by the unusual robustness of our system, we focused on polymerization conditions using even less Ru initiator. When **G2** was used as a catalyst using **CTA1** and **M1**, a catalytic polymerization using 500 times less Ru initiator than required classically resulted in very low monomer conversion (<10%) even with extended reaction time.

A higher monomer concentration increased the conversion to only 35% after 48 h of reaction time (see Table S2). When **HG-II** was used instead of **G2**, a reasonable monomer conversion (90%) was achieved within 20 h along with a controlled polymerization (**HG-II**:**CTA1** = 1:500, **P22**, *M*_n,SEC(CHCl_3_)_ = 3.0 kDa). This could be attributed to the known higher turnover number for **HG-II** over **G2**.^54^ Having successfully carried out catalytic polymerization using 500 times less Ru initiator than typically required, we aimed for lowering the catalyst to 1/1000 of the usual amount. Thus, **P23** was synthesized (**HG-II**:**CTA1**:**M1** = 1:1000:15,000, *M*_n,SEC(CHCl_3_)_ = 4.0 kDa) with a monomer conversion of 85%. **P23** was fully characterized by ^1^H NMR spectroscopy and MALDI-TOF MS and showed the expected end groups. This is to date the lowest reported catalytic ROMP polymerization to synthesize norbornene imide-based metathesis polymers.

## Conclusions

In conclusion, we have successfully developed a simple yet highly robust polymerization method to prepare ROMP polymers catalytically under non-degassed conditions using commercially available and inexpensive styrenes as CTAs. Telechelic polymers with functional groups such as aldehyde, atom transfer radical polymerization initiator (benzyl chloride), aromatic amine, aromatic acid, nitro, cyano, and primary alcohol were synthesized using a catalytic amount of costly and toxic metathesis-based catalysts. Antimicrobial ROMP polymers that were previously shown to be very effective against specific bacteria could be prepared on the gram scale with our method. In this report, the catalyst to CTA ratio examined was as low as 1:1000, which simply means a 1000-fold saving of expensive ruthenium–metal complex. Ring-opening metathesis polymerization offers access to a pool of highly functional and valuable materials for many different disciplines. The limiting factor regarding the synthesis of those materials appears to be the stoichiometric use of expensive metathesis catalysts. Our newly discovered CTAs based on styrene and its derivatives could overcome this limitation.

## Methods

Catalytic ROMP was performed at room temperature using dry dichloromethane (non-degassed) as the solvent. A typical procedure (reaction equivalents are given for the synthesis of polymer **P18**) is as follows: in a round-bottom flask equipped with a magnetic stirrer bar, **M1** (4500 equiv, 2.5 g) was added followed by the addition of 25 mL of dichloromethane. In a vial, 3BPY (30 equiv, 15 mg) and **CTA8** (150 equiv, 143 mg) were dissolved in 3 mL of dichloromethane and added to the flask. To this stirred mixture, **HG-II** (1 equiv, 2 mg) dissolved in 0.5 mL dichloromethane was added (to give a final concentration of 0.5 M with respect to **M1**), and the flask was capped with a rubber septum and stirred at room temperature until all the monomer was consumed (monitored by ^1^H NMR spectroscopy). Then, few drops of ethyl vinyl ether were added. The solvent was removed by rotary evaporation, and the crude polymer was precipitated once from cold methanol (150 mL) to obtain a colorless solid with a yield of 91%.

## Supporting Information Available

Instrument’s data, experimental methods, NMR data, MALDI-TOF data, and SEC data (PDF)
