# Supplementary material for: Practical Route for Catalytic Ring-Opening Metathesis Polymerization
Source: JACS Au. 2022 Dec 2;2(12):2800–8. doi: 10.1021/jacsau.2c00566 (PMC9795566; doi:10.1021/jacsau.2c00566)
Supplement: Supplementary file 1 — au2c00566_si_001.pdf [file au2c00566_si_001.pdf]

A Practical Route for Catalytic Ring opening Metathesis Polymerization

Indradip Mandal and Andreas F. M. Kilbinger*

Department of chemistry, University of Fribourg, Chemin du Musée 9, 1700 Fribourg (Switzerland)

andreas.kilbinger@unifr.ch

Table of Contents

Materials and Instruments 3

Materials 3

Instruments 3

Synthesis of Chain Transfer Agents (CTAs): 4

CTA7 4

CTA8 4

CTA9 5

CTA10 6

CTA11 7

CTA12 7

Synthesis of Monomers: 8

M1 8

M3 8

M4 9

Endo-N-methylnorbornene imide (endo-MNI) 11

NMR tube reactions: 12

Polymerization of **M1** using **CTA1** and **G3**: 12

End capping experiment: 14

Polymerization of **M1** in presence of **3BPY** using **CTA1** and **G3**: 17

General Procedure for catalytic ROMP: 20

Table S1: Polymerization data 21

Optimization for higher catalytic Polymerization: 25

Table S2 25

NMR tube polymerization: 26

P8 : 26

P9: 27

P11: 29

P13: 31
P17: 33
P20: 35
P29: 37
P30 : 38
Amine and acid functional ROMP polymers: 40
Catalytic ROMP in an Erlenmeyer flask: 40
Antimicrobial ROMP polymer: 41
Effect of additives on catalytic ROMP: 42
(a)Pyridine as an additive- 42
(b)Triphenylphosphine as an additive- 45
Slow propagating monomer for catalytic ROMP: 47
Catalytic polymerization at higher temperature: 52
Kinetics Experiment: 52
Determination of rate constants by Mayo equation: 56
Zoomed MALDI-ToF with mono-isotopic mass simulation: 57
NMR spectra of chain transfer agents and monomers: 58
NMR spectra of Polymers: 70
MALDI-ToF mass spectra: 84
SEC data of polymers: 99
High-Resolution Mass Spectrometric Data (HRMS data): 106
References: 109

Materials and Instruments

Materials

Styrene (**CTA1**), 4-Acetoxystyrene (**CTA3**), 3-Vinylbenzaldehyde (**CTA4**), 4-*tert*-Butoxystyrene (**CTA5**), 4-Vinylbenzyl chloride (**CTA6**), Methyl 4-formylbenzoate, and Terephthalaldehyde was purchased from Sigma-Aldrich. 4-Bromostyrene (**CTA2**) was purchased from Combi-Blocks. 4-Vinylaniline, and 4-Vinylbenzoic acid were purchased from Fluorochem. All other reagents were purchased from either Sigma-Aldrich or Acros organics. All of them were used without further purification. Deuterated solvents (CD_2Cl_2, $CDCl_3$, and DMSO-D_6) were purchased from Cambridge Isotope Laboratories, Inc. Grubbs 3rd generation catalyst (**G3**) was prepared as reported previously.[1] $CDCl_3$ was passed through a plug of basic alumina before using in a polymerization reaction.

Instruments

All ^{1}H NMR, ^{13}C NMR NMR spectra were recorded on a Bruker Avance DPX (400 MHz and 300 MHz) FT NMR spectrometer. Chemical shifts for ^{1}H and ^{13}C were given in ppm relative to the residual solvent peak ($CDCl_3$: 7.27 for ^{1}H; $CDCl_3$: 77.16 for ^{13}C and CD_2Cl_2: 5.32 for ^{1}H; CD_2Cl_2: 53.84 for ^{13}C). HR MALDI FT-ICR mass spectra were measured on a Bruker FTMS 4.7T BioAPEX II in positive mode using trans-2-[3-(tert-butylphenyl)-2-methyl-2-propenylidene]malononitrile (DCTB) as matrix and sodium trifluoroacetate (NaTFA) or silver trifluoroacetate (AgTFA) as the counter ion source. HR-MS (ESI+) mass spectra were measured on a Bruker FTMS 4.7T BioAPEX II and Thermo Scientific LTQ Orbitrap XL equipped with a static nanospray ion source. Relative molecular weights and molecular weight distributions were measured by gel permeation chromatography (GPC) with either chloroform or DMF as eluent with a flow rate of 1 mL/min at 40°C and 60°C, respectively. The chloroform GPC system was calibrated with polystyrene standards in a range from $10^3$to 3×10^6 Da. The Chloroform GPC is an automated PSS security System (Agilent Technologies 1260 infinity ll) with a set of two MZ-Gel SDplus linear columns (300 x 8 mm, 5 µm particle size). Signals were recorded by an interferometric refractometer (Agilent 1260 series) (as refractive index or RI traces). All polymer samples were filtered through a PTFE syringe membrane filter (0.45 µm pore size, VWR) before GPC measurements.

Synthesis of Chain Transfer Agents (CTAs):

CTA7

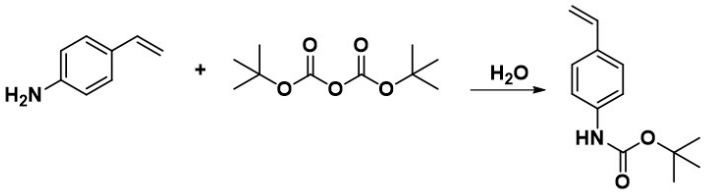

CTA7 was prepared according to a previous reported literature,[2]

4-Vinylaniline (1.0 equiv., 12.8 mmol, 1.5 mL) was added to a solution of di-tert-butyl dicarbonate (1.1 equiv., 14 mmol, 3.06 g) in water (16 mL). The reaction mixture was stirred for 23 h at ambient temperature. After that time ethyl acetate (30 mL) was added and the layers separated. The aqueous layer was extracted with ethyl acetate (3x10 mL). The combined organic layers were washed with brine and dried over $MgSO_4$. After filtration the solvent was removed under reduced pressure. The crude product was purified by column chromatography (8% ethyl acetate-hexane). tert-Butyl (4-vinylphenyl)carbamate (**CTA7**) was obtained in 97% yield (2.70 g, 12 mmol) as a white solid.

^{1}H NMR (400 MHz, CHLOROFORM-d) δ ppm: 1.38 - 1.56 (m, 9 H), 5.03 - 5.19 (m, 1 H), 5.49 - 5.66 (m, 1 H), 6.43 - 6.68 (m, 2 H). ^{13}C NMR (101 MHz, CHLOROFORM-d) δ ppm: 14.1, 27.4, 28.3, 60.4, 76.7, 77.3, 80.5, 112.3, 118.4, 126.8, 132.5, 136.2, 137.9, 152.6.

HR-MS (ESI) calculated for $C_{13}H_{17}NO_2H^+$ $[M+H]^+$: 220.1338, Found : 220.1332.

CTA8

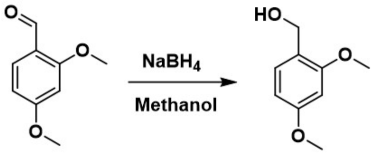

I1

To a solution of a 2,4-dimethoxybenzaldehyde (1 equiv., 20 mmol, 3.3 g) in MeOH (50 mL) was added $NaBH_4$ (0.90 g, 24 mmol) at 0°C under argon. After stirring the reaction mixture until the substrate was completely consumed, water was added to the reaction mixture, which was concentrated in vacuo, and the obtained residue was extracted with ethyl acetate (30 mL × 3). The combined organic layers were dried over $MgSO_4$ and concentrated in vacuo. I1 was obtained as a grey solid in quantitative yield which was used in the next step without further purification.

^1H NMR (300 MHz, CHLOROFORM-d) δ ppm: 3.85 (s, 3 H), 3.82 (s, 3 H), 4.62 (s, 2 H), 6.36 - 6.56 (m, 2 H), 7.08 - 7.24 (m, 1 H). ^{13}C NMR (101 MHz, CHLOROFORM-d) δ ppm: 55.2, 55.3, 61.6, 76.7, 77.3, 98.5, 103.8, 121.8, 129.6, 158.5, 160.6.

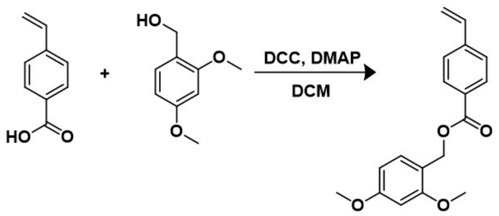

4-vinylbenzoic acid (1 equiv., 2.76 mmol, 408 mg), I1 (1.1 equiv., 3.03 mmol, 510 mg), and DMAP (0.1 equiv., 0.28 mmol, 34 mg) were dissolved in 15 mL DCM and cooled to 0^0C. DCC (1.1 equiv., 3.03 mmol, 625 mg) was dissolved in 5 mL DCM and added to the mixture slowly. Then, the resulting solution was stirred at room temperature overnight. Next, it was filtered, and the filtrate was worked up with ethyl acetate and brine. The organic part dried over magnesium sulfate, concentrated and purified by column chromatography (10% ethyl acetate-hexane) to give **CTA8** as a white solid (660 mg, 2.2 mmol, 80%).

^1H NMR (400 MHz, CHLOROFORM-d) δ ppm: 3.84 (s, 3 H), 3.83 (s, 3 H), 5.29 - 5.44 (m, 3 H), 5.86 (dd, *J* = 17.61, 0.86 Hz, 1 H), 6.50 (dd, *J* = 4.34, 2.26 Hz, 2 H), 6.75 (dd, *J* = 17.61, 10.88 Hz, 1 H), 7.34 (d, *J* = 8.80 Hz, 1 H), 7.39 - 7.53 (m, 2 H), 7.95 - 8.10 (m, 2 H). ^{13}C NMR (101 MHz, CHLOROFORM-d) δ ppm: 55.4, 55.5, 62.1, 76.7, 77.3, 98.6, 104.1, 116.3, 116.9, 126.0, 129.7, 130.0, 131.1, 136.1, 141.8, 159.0, 161.2, 166.4.

HR-MS (ESI) calculated for $C_{18}H_{18}O_4H^+$ $[M+H]^+$: 299.1283, Found : 299.1278.

CTA9

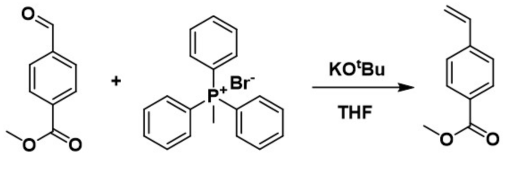

Methyltriphenylphosphonium bromide (1.2 equiv., 15.35 mmol, 5.5 g) was dissolved in 30 mL THF and cooled to 0^0C. Solid potassium tert-butoxide (1.2 equiv., 15.35 mmol, 1.73 g) was added in one shot, and the THF solution immediately became yellow. The solution was stirred at 0^0C for 10 mins. Methyl 4-formylbenzoate (1 equiv., 12.80 mmol, 2.1 g) was dissolved in 1 mL THF and added slowly to the precooled mixture. Then, the resulting solution was stirred at room temperature for 15 mins. THF was evaporated under reduced pressure, and crude was dissolved in ethyl acetate and worked up against brine two times. The organic part was dried over magnesium sulfate, concentrated under reduced

pressure, and further purified by column chromatography (5% ethyl acetate-hexane) to obtain **CTA9** as a colorless crystals (1.7 g, 11 mmol, 83% yield).

^{1}H NMR (400 MHz, CHLOROFORM-d) δ ppm: 3.92 (s, 3 H), 5.29 - 5.45 (m, 1 H), 5.86 (dd, *J* = 17.61, 0.73 Hz, 1 H), 6.75 (dd, *J* = 17.61, 10.88 Hz, 1 H), 7.39 - 7.51 (m, 2 H), 7.87 - 8.08 (m, 2 H). ^{13}C NMR (101 MHz, CHLOROFORM-d) δ ppm: 28.2, 52.0, 76.7, 77.3, 116.0, 116.4, 125.9, 126.1, 129.2, 129.7, 129.8, 136.0, 136.1, 141.9, 166.8.

HR-MS (ESI) calculated for $C_{10}H_{10}O_2H^+$ $[M+H]^+$: 163.0759, Found : 163.0753.

CTA10

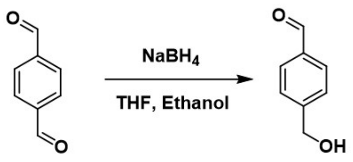

I2

Terephthalaldehyde (1 equiv., 76 mmol, 10.2 g) was dissolved in 100 mL THF and 50 mL MeOH mixture and cooled to 0^{0}C. To this solution, sodium borohydride (0.35 equiv., 26.3 mmol, 1 g) was added in one shot and the solution was stirred at that temperature for 1 h. Full consumption of starting material was observed during that time. THF and MeOH were evaporated under reduced pressure and the solid crude was worked up with ethyl acetate and brine solution for two times. Organic part was dried over magnesium sulfate, the resulting solution was then concentrated and further purified with silica gel column chromatography (30%-40% ethyl acetate and hexane) to obtain I2 (7g, 51.5 mmol, 68% yield) as a white solid.

^{1}H NMR (300 MHz, CHLOROFORM-d) δ ppm: 2.37 - 2.76 (m, 1 H), 4.78 (s, 2 H), 7.44 - 7.64 (m, 2 H), 7.77 - 7.94 (m, 2 H), 9.97 (s, 1 H). ^{13}C NMR (75 MHz, CHLOROFORM-d) δ ppm: 64.4, 76.6, 77.4, 126.9, 130.0, 192.2.

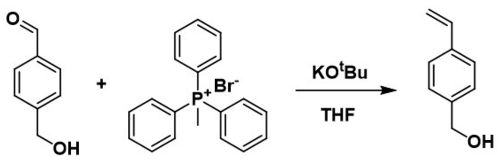

Methyltriphenylphosphonium bromide (1.2 equiv., 8.8 mmol, 3.15 g) was dissolved in 20 mL THF and cooled to 0^{0}C. Solid potassium tert-butoxide (2.4 equiv., 17.63 mmol, 2.00 g) was added in one shot, and the THF solution immediately became yellow. The solution was stirred at 0^{0}C for 10 mins. I2 (1 equiv., 7.35 mmol, 1.0 g) was dissolved in 5 mL THF and added slowly to the precooled mixture. Then, the resulting solution was stirred at room temperature overnight. 20 mL saturated aqueous ammonium chloride solution was added on the next day. THF was evaporated under reduced pressure,

and crude was dissolved in ethyl acetate and worked up against brine two times. The organic part was dried over magnesium sulfate, concentrated under reduced pressure, and further purified by column chromatography (20-30% ethyl acetate-hexane) to obtain **CTA10** as a yellow oil which became a solid upon storage inside the fridge (760 mg, 5.7 mmol, 77% yield).

^{1}H NMR (300 MHz, CHLOROFORM-d) δ ppm: 1.81 - 2.02 (m, 1 H), 4.67 (s, 2 H), 5.15 - 5.38 (m, 1 H), 5.65 - 5.88 (m, 1 H), 6.73 (dd, *J* = 17.65, 10.87 Hz, 1 H), 7.22 - 7.57 (m, 4 H). ^{13}C NMR (75 MHz, CHLOROFORM-d) δ ppm: 65.0, 76.6, 77.4, 113.9, 126.3, 127.2, 136.4, 137.0, 140.4.

HR-MS (ESI) calculated for $C_9H_{10}O^+$ $[M]^+$: 134.0732, Found : 134.0728.

CTA11

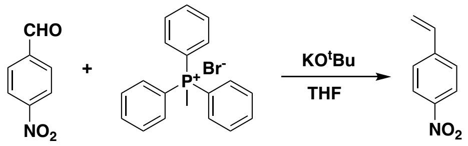

Methyltriphenylphosphonium bromide (1.2 equiv., 7.9 mmol, 2.83 g) was dissolved in 20 mL THF and cooled to 0^{0}C. Solid potassium tert-butoxide (1.2 equiv., 7.9 mmol, 890 mg) was added in one shot, and the THF solution immediately became yellow. The solution was stirred at 0^{0}C for 10 mins. 4-nitrobenzaldehyde (1 equiv., 6.6 mmol, 1.0 g) was dissolved in 5 mL THF and added slowly to the precooled mixture. Then, the resulting solution was stirred at room temperature overnight. 20 mL saturated aqueous ammonium chloride solution was added on the next day. THF was evaporated under reduced pressure, and crude was dissolved in ethyl acetate and worked up against brine two times. The organic part was dried over magnesium sulfate, concentrated under reduced pressure, and further purified by column chromatography (20-30% ethyl acetate-hexane) to obtain **CTA11** as a yellow oil which became a solid upon storage inside the fridge (650 mg, 4.3 mmol, 66% yield).

^{1}H NMR (300 MHz, CHLOROFORM-d) δ ppm: 5.51 (dd, *J* = 10.87, 0.41 Hz, 1 H), 5.77 - 6.09 (m, 1 H), 6.55 - 6.92 (m, 1 H), 7.46 - 7.68 (m, 2 H), 8.05 - 8.36 (m, 2 H). ^{13}C NMR (75 MHz, CHLOROFORM-d) δ ppm: 76.6, 77.2, 77.4, 118.6, 123.9, 126.8, 135.0, 143.8.

CTA12

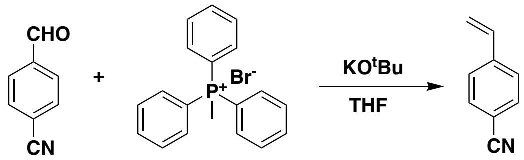

Methyltriphenylphosphonium bromide (1.1 equiv., 8.4 mmol, 3 g) was dissolved in 20 mL THF and cooled to 0^{0}C. Solid potassium tert-butoxide (1.1 equiv., 8.4 mmol, 942 mg) was added in one shot, and the THF solution immediately became yellow. The solution was stirred at 0^{0}C for 10 mins. 4-

formylbenzonitrile (1 equiv., 7.6 mmol, 1.0 g) was dissolved in 5 mL THF and added slowly to the precooled mixture. Then, the resulting solution was stirred at room temperature overnight. 20 mL saturated aqueous ammonium chloride solution was added on the next day. THF was evaporated under reduced pressure, and crude was dissolved in dichloromethane and worked up against brine two times. The organic part was dried over magnesium sulfate, concentrated under reduced pressure, and further purified by column chromatography (10% ethyl acetate-hexane) to obtain **CTA12** as a colorless liquid (870 mg, 6.7 mmol, 88% yield).

^{1}H NMR (300 MHz, CHLOROFORM-d) δ ppm: 5.35 - 5.55 (m, 1 H), 5.79 - 6.01 (m, 1 H), 6.73 (dd, *J* = 17.61, 10.91 Hz, 1 H), 7.41 - 7.55 (m, 2 H), 7.57 - 7.77 (m, 2 H). ^{13}C NMR (75 MHz, CHLOROFORM-d) δ ppm: 76.6, 77.4, 111.0, 117.7, 118.9, 126.7, 132.3, 135.3, 141.8.

Synthesis of Monomers:

M1

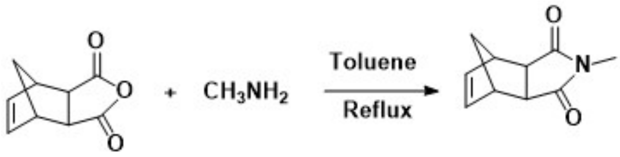

Exo-N-methylnorbornene imide (exo-MNI) (**M1**) was synthesized according to the previously reported procedure[3].

M3

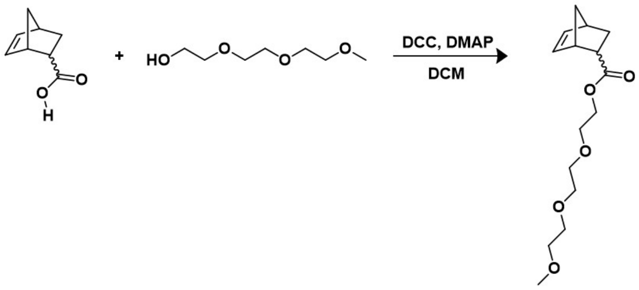

Bicyclo[2.2.1]hept-5-ene-2-carboxylate (1 equiv., 14.47 mmol, 2.0 g), triethylene glycol monomethyl ether (1.1 equiv., 15.92 mmol, 2.62 g), and DMAP (0.1 equiv., 1.45 mmol, 177 mg) were dissolved in 15 mL DCM and cooled to 0^{0}C. DCC (1.2 equiv., 17.37 mmol, 3.56 g) was dissolved in 10 mL DCM and added to the mixture slowly. Then, the resulting solution was stirred at room temperature overnight. Next, it was filtered, and the filtrate was worked up with ethyl acetate and brine. The organic part dried over magnesium sulfate, concentrated and purified by column chromatography (30% ethyl acetate-hexane) to give **M3** as a viscous liquid which was stored at -20^{0}C (3.2 g, 11 mmol, 78% yield).

^{1}H NMR (400 MHz, CHLOROFORM-d) δ ppm: 1.23 - 1.34 (m, 1 H), 1.34 - 1.48 (m, 2 H), 1.85 - 1.99 (m, 1 H), 2.88 - 3.09 (m, 2 H), 3.22 (tdd, 1 H), 3.34 - 3.41 (m, 3 H), 3.53 - 3.61 (m, 2 H), 3.61 - 3.74 (m, 9 H), 4.09 - 4.29 (m, 2 H), 5.94 (dd, *J* = 5.69, 2.87 Hz, 1 H), 6.19 (dd, *J* = 5.62, 3.06 Hz, 1 H). ^{13}C NMR (101 MHz, CHLOROFORM-d) δ ppm: 29.2, 30.3, 41.6, 42.5, 43.0, 43.2, 45.7, 46.3, 46.7, 49.6, 59.0, 63.3, 63.5, 69.2, 70.5, 70.6, 70.6, 71.9, 76.7, 77.3, 132.4, 135.7, 137.7, 138.1, 174.7.

HR-MS (ESI) calculated for $C_{15}H_{24}O_5H^+$ $[M+H]^+$: 285.1702, Found : 285.1703; $C_{15}H_{24}O_5Na^+$ $[M+Na]^+$: 307.1521, Found : 307.1521.

M4

M4 was prepared according to the reported literature procedure[4] with some minor changes as follow-

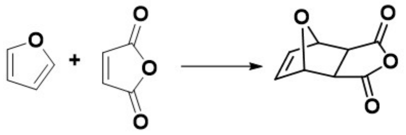

I3

Maleic anhydride (9.8 g, 100 mmol) was dissolved in 30 mL toluene. 150 mL (13.6 g, 200 mmol) furan were added. The solution was stirred overnight. The product was then filtered, washed with toluene and dried under vacuum. A colorless powder was obtained. Yields and spectroscopic data matched those reported previously.[5]

^{1}H NMR (400 MHz, CHLOROFORM-d) δ ppm: 3.18 (s, 2 H), 5.46 (t, *J* = 0.98 Hz, 2 H), 6.58 (t, *J* = 1.04 Hz, 2 H). ^{13}C NMR (101 MHz, CHLOROFORM-d) δ ppm: 48.7, 76.7, 77.2, 77.3, 82.2, 109.4, 136.5, 137.0, 142.5, 169.9.

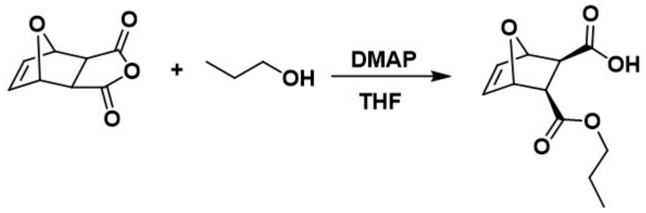

I4

I3 (1 equiv., 60.2 mmol, 10 g) and DMAP (0.1 equiv., 6 mmol, 736 mg) was dissolved in 70 mL THF. Then, prpan-1-ol (2 equiv., 120.4 mmol, 7.24g, 9 mL) was added to the flask and stirred overnight. The next day, solvent was removed by vacuum evaporation at room temperature. A highly viscous liquid was obtained which under high vacuum became a solid due to evaporation of excess alcohol. This solid was further crystallized from dichloromethane/hexane mixture to give I4 as a colorless solid (10 g, 44.2 mmol, 73.4% yield).

^1H NMR (400 MHz, DMSO-d_6) δ ppm: 0.87 (t, *J* = 7.40 Hz, 3 H), 1.46 - 1.65 (m, 2 H), 2.71 (s, 2 H), 3.82 - 3.99 (m, 2 H), 5.00 - 5.14 (m, 2 H), 6.45 (ddd, *J* = 2.96, 1.31, 0.61 Hz, 2 H). ^{13}C NMR (101 MHz, DMSO-d_6) δ ppm: 10.5, 21.5, 39.1, 39.3, 39.7, 39.9, 40.1, 40.3, 46.2, 46.8, 65.8, 79.9, 80.2, 136.7, 136.9, 171.7, 172.8.

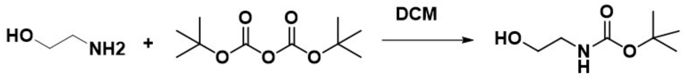

I5

Ethanolamine (1 equiv., 89.8 mmol, 5.5 g) and di-tert-butyl dicarbonate (1 equiv., 89.8 mmol, 19.6 g) were dissolved in 100 mL DCM and stirred overnight at room temperature. Solvent was evaporated and the mixture was purified via column chromatography (50% ethyl acetate-hexane) to give I5 as a viscous colorless liquid (13g, 81 mmol, 90% yield).

^1H NMR (400 MHz, CHLOROFORM-d) δ ppm: 1.42 (s, 9 H), 3.24 (q, *J* = 5.42 Hz, 3 H), 3.65 (q, *J* = 5.34 Hz, 2 H), 5.17 (br. s., 1 H). ^{13}C NMR (101 MHz, CHLOROFORM-d) δ ppm: 28.3, 43.0, 62.2, 76.7, 77.3, 79.5, 156.8.

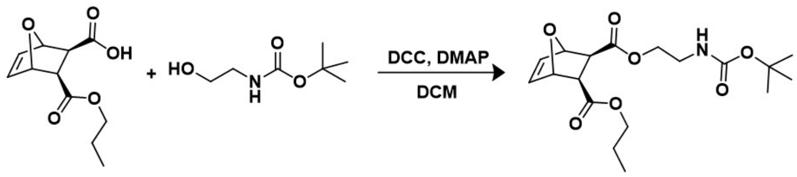

I4 (1 equiv., 22.10 mmol, 5.0 g), and DMAP (0.1 equiv., 2.21 mmol, 270 mg) were dissolved in 30 mL DCM and cooled to 0^0C. I5 (1 equiv., 22.10 mmol, 3.57 g) was dissolved in 10 mL DCM and added to the pre-cooled mixture. Then-, DCC (1.1 equiv., 24.31 mmol, 5.1 g) was dissolved in 10 mL DCM and added to the mixture slowly. The resulting solution was stirred at room temperature overnight. Next, it was filtered, and the filtrate was worked up with ethyl acetate and brine. The organic part dried over magnesium sulfate, concentrated and purified by column chromatography (40% ethyl acetate-hexane) to give **M4** as a colorless viscous liquid which was stored at -20^0C (5.8 g, 16 mmol, 71% yield).

^1H NMR (400 MHz, CHLOROFORM-d) δ ppm: 0.96 (t, *J* = 7.46 Hz, 3 H), 1.45 (s, 9 H), 1.63 - 1.74 (m, 2 H), 2.72 - 2.95 (m, 2 H), 3.35 - 3.50 (m, 2 H), 4.00 - 4.27 (m, 4 H), 5.06 (br. s., 1 H), 5.19 - 5.35 (m, 2 H), 6.47 (dt, *J* = 1.44, 0.69 Hz, 2 H). ^{13}C NMR (101 MHz, CHLOROFORM-d) δ ppm: 10.4, 21.9, 28.4, 39.5, 46.8, 47.2, 64.8, 66.9, 76.7, 77.2, 77.3, 79.3, 80.4, 80.7, 136.6, 136.7, 171.5, 171.7.

HR-MS (ESI) calculated for $C_{18}H_{27}NO_7H^+$ $[M+H]^+$: 370.1866, Found : 370.1860.

Endo-N-methylnorbornene imide (endo-MNI)

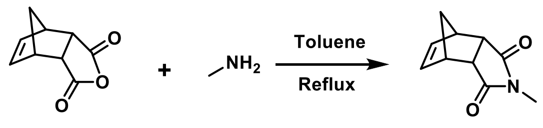

A similar synthetic procedure as of **M1** was followed to obtain endo-MNI as a colorless solid.

^{1}H NMR (400 MHz, CHLOROFORM-d) δ ppm : 1.45 - 1.59 (m, 1 H), 1.71 (dt, *J* = 8.77, 1.73 Hz, 1 H), 2.79 (s, 3 H), 3.14 - 3.29 (m, 2 H), 3.29 - 3.48 (m, 2 H), 6.06 (t, *J* = 2.02 Hz, 2 H). ^{13}C NMR (101 MHz, CHLOROFORM-d) δ ppm : 24.1, 44.7, 45.9, 52.1, 76.7, 77.3, 134.3, 177.7.

NMR tube reactions:

Polymerization of M1 using CTA1 and G3:

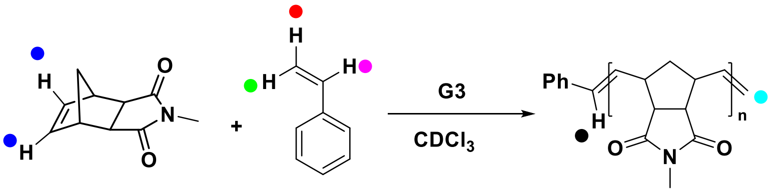

In an NMR tube, styrene (**CTA1**) (15 equiv., 0.0254 mmol, 2.65 mg) and exo-MNI (**M1**) (300 equiv., 0.5087 mmol, 90 mg) were mixed in 1 mL chloroform-d ($CDCl_3$) and ^{1}H NMR was measured (see green spectrum below). Then, to it, Grubbs 3rd generation catalyst (**G3**) (0.0017 mmol, 1.5 mg) dissolved in 0.3 mL $CDCl_3$ was added quickly and ^{1}H NMR was measured immediately (<10 mins) (see blue spectrum below). Within this time frame, to our surprise, total consumption of monomer (**M1**) was observed along with more than 97% consumption of **CTA1**. **CTA1** peak at 6.55 ppm was vanished almost completely. Moreover, ruthenium alkylidene region (see 18-19.5 ppm below) showed both **G3**-alkylidene (propagating carbene) and **G3**-benzylidene (due to chain transfer with styrene after all the monomer is consumed). A very little peak at 18.9 ppm could be due to catalyst decomposition. The resulting $CDCl_3$ solution was concentrated and precipitated into cold methanol to obtain polymer **P1**.

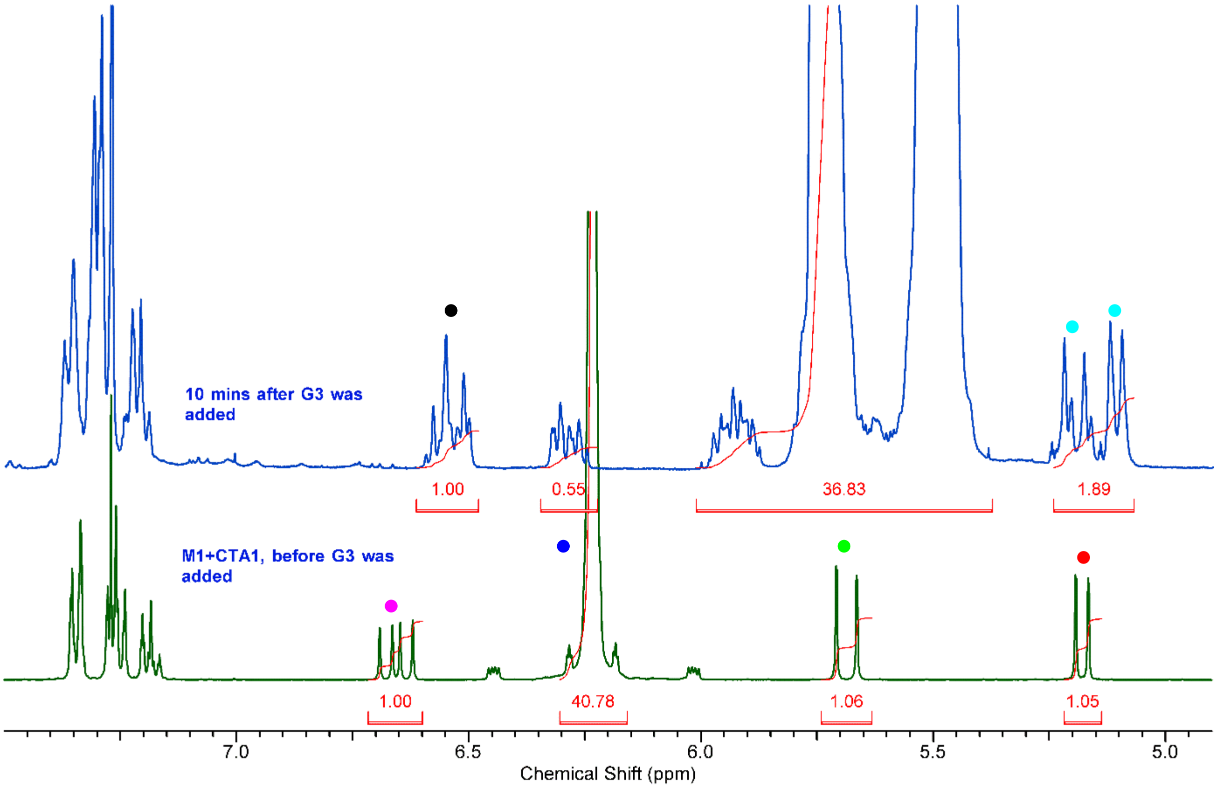

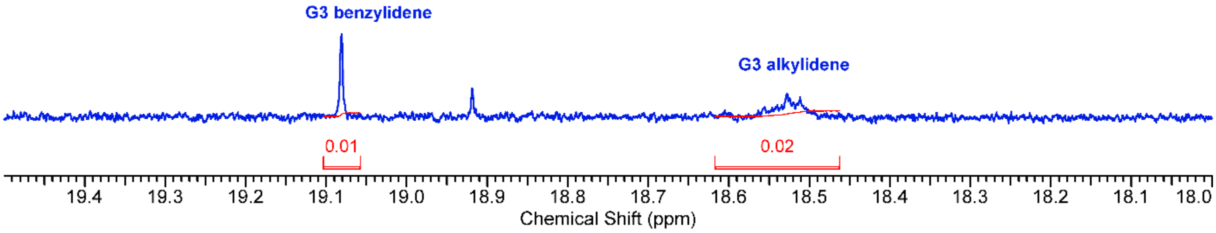

Fig. S1: ^{1}H NMR spectra ($CDCl_3$, 400 MHz) of polymerization of **M1** (300 equiv.) using **CTA1** (15 equiv.) and **G3** (1 equiv.) as the catalyst.

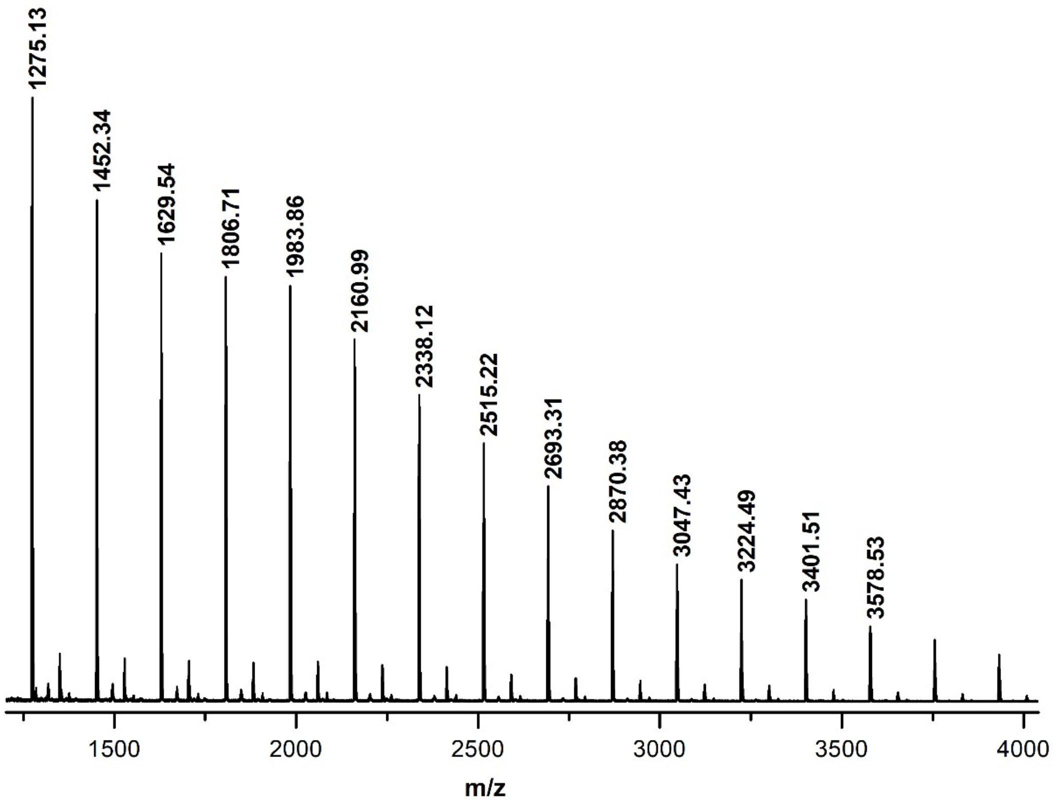

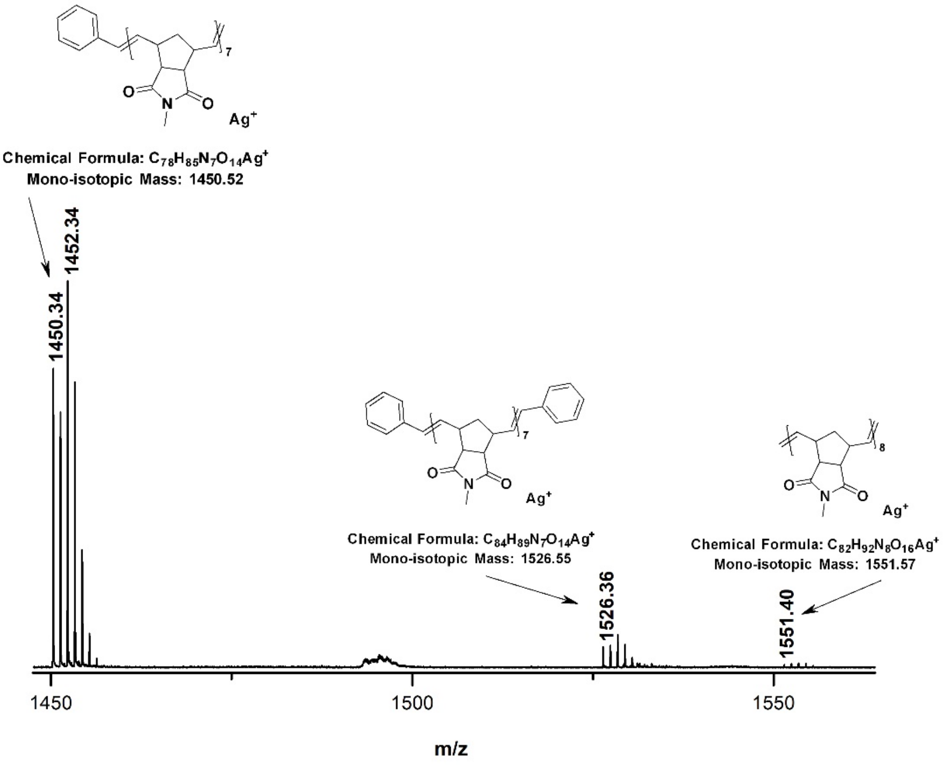

Fig. S2: MALDI-ToF mass spectrum (DCTB, AgTFA) of **P1**.

End capping experiment:

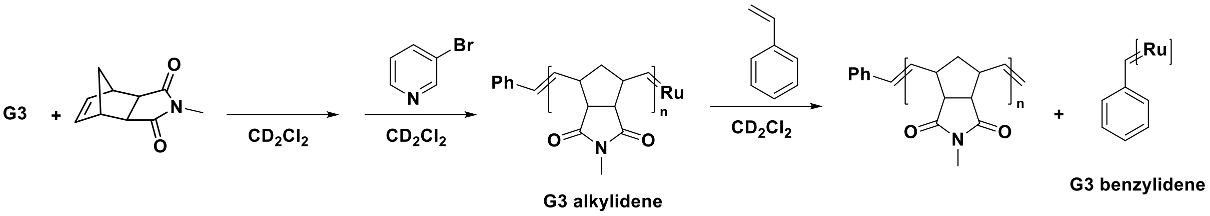

G3 (1 equiv., 0.0045 mmol, 4 mg) was dissolved in 0.5 mL dichloromethane-d_2 (CD_2Cl_2) in an NMR tube and to it exo-MNI (**M1**) (20 equiv., 0.09 mmol, 16 mg) in 0.2 mL CD_2Cl_2 was quickly added. ^{1}H NMR was measured. 3-bromopyridine (**3BPY**) (30 equiv., 0.1357 mmol, 21.5 mg) was dissolved in 0.2 mL and added to the NMR tube. Then, **CTA1** (5 equiv., 0.0226 mmol, 4 mg) dissolved in 0.3 mL CD_2Cl_2 was added, and ^{1}H NMR was measured over time. After 15 mins, the CD_2Cl_2 mixture was concentrated under reduced pressure at room temperature, and the formed polymer was precipitated in methanol. The polymer (**P2**) was obtained as a grey solid.

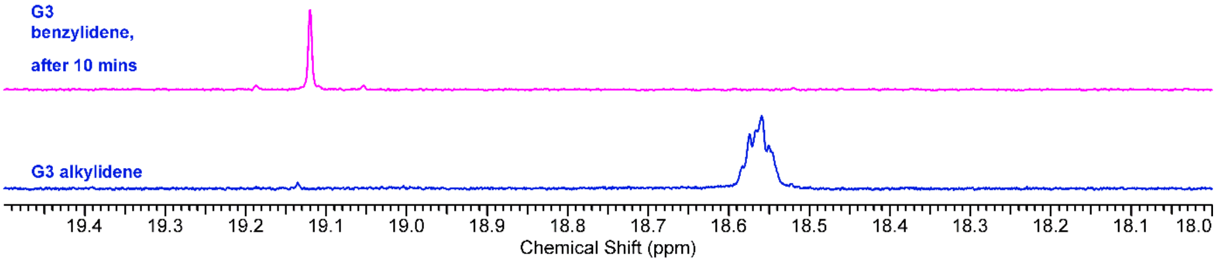

Fig. S3: ^{1}H NMR spectra (CD_2Cl_2, 300 MHz) of reaction of a propagating **G3** complex (**G3**-alkylidene, 18.57 ppm) with 5 equiv. of **CTA1** generating **G3**-benzylidene (19.13 ppm) within the first measurement (<10 mins).

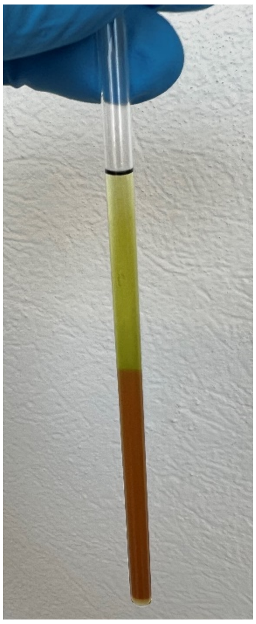

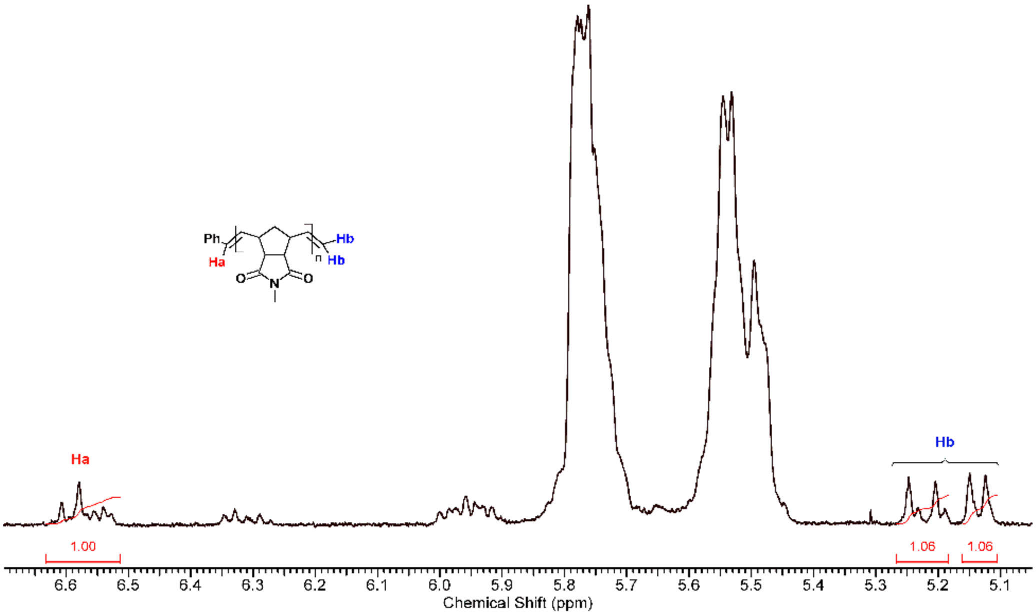

Fig. S4: Left image: After addition of 5 equiv. of **CTA1** (without shaking the NMR tube) to the **G3**-benzylidene initiated poly (**M1**) (**G3**-alkylidene) showing green color of the upper portion of the NMR tube because of the formation of **G3**-benzylidene due to fast, regioselective chain transfer with **CTA1**. Lower portion of the NMR tube still showed deep yellow color of **G3**-alkylidene. Right image: Zoomed ^{1}H NMR spectrum of **P2** showing efficient end capping with **CTA1**. With respect to the integration of 1 of the styrenic proton Ha (comes from **G3**), the methylene proton Hb showed the integration of 1.06, which should be the case if **CTA1** (styrene) transfers methylene group regioselectively to the propagating polymer chain (**G3**-alkylidene).

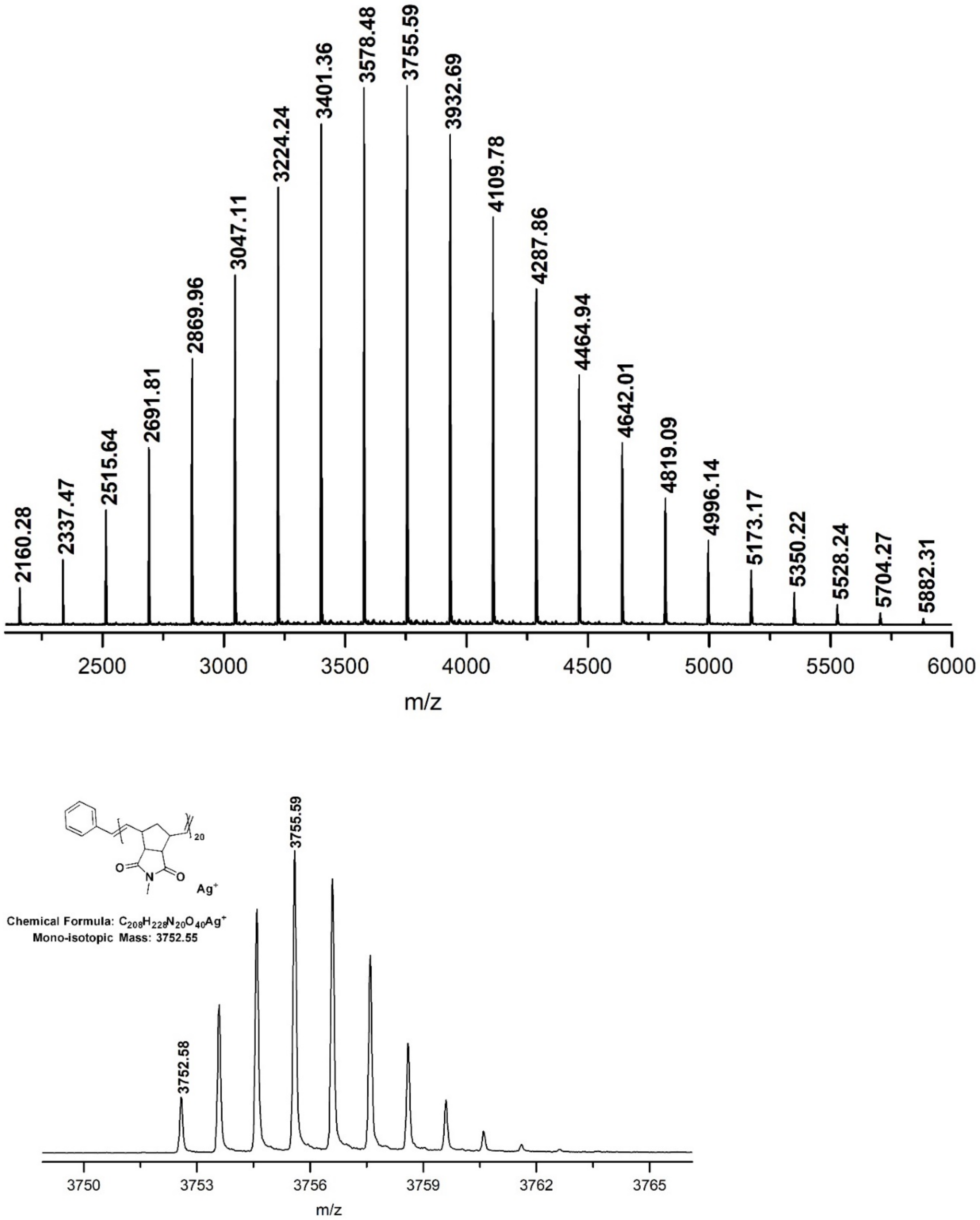

Fig. S5: MALDI-ToF mass spectrum (DCTB, AgTFA) of **P2**, further confirmed the regioselective chain transfer of **CTA1** under the given reaction conditions.

Polymerization of M1 in presence of 3BPY using CTA1 and G3:

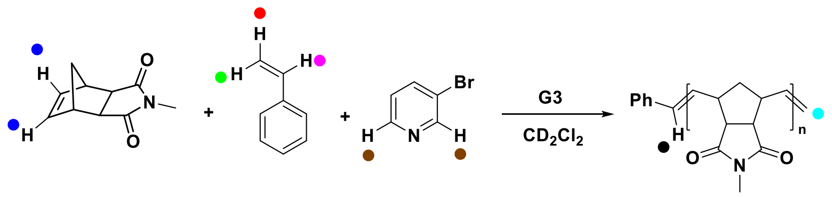

In an NMR tube, **G3** (0.0034 mmol, 3 mg), and 3-bromopyridine (**3BPY**) (30 equiv., 0.101 mmol, 16.1 mg) were mixed 0.5 mL CD_2Cl_2 and ^1H NMR was measured. Styrene (**CTA1**) (5 equiv., 0.014 mmol, 1.77 mg) was dissolved in 0.2 mL CD_2Cl_2 and added to the same NMR tube and ^1H NMR was recorded. Then, **M1** (60 equiv., 0.204 mmol, 36 mg) dissolved in 0.2 mL CD_2Cl_2 was added immediately followed by addition of **G3** (1 equiv., 0.0034 mmol, 3 mg) in 0.2 mL of CD_2Cl_2. ^1H NMR was measured over time. Consumption of both **CTA1** and **M1** was observed via ^1H NMR spectroscopy which is a typical proof for kinetically controlled polymerization. After 25 mins, both monomer and **CTA1** were consumed almost fully (>95%). The resulting solution was further concentrated and precipitated into cold methanol to obtain polymer **P3**.

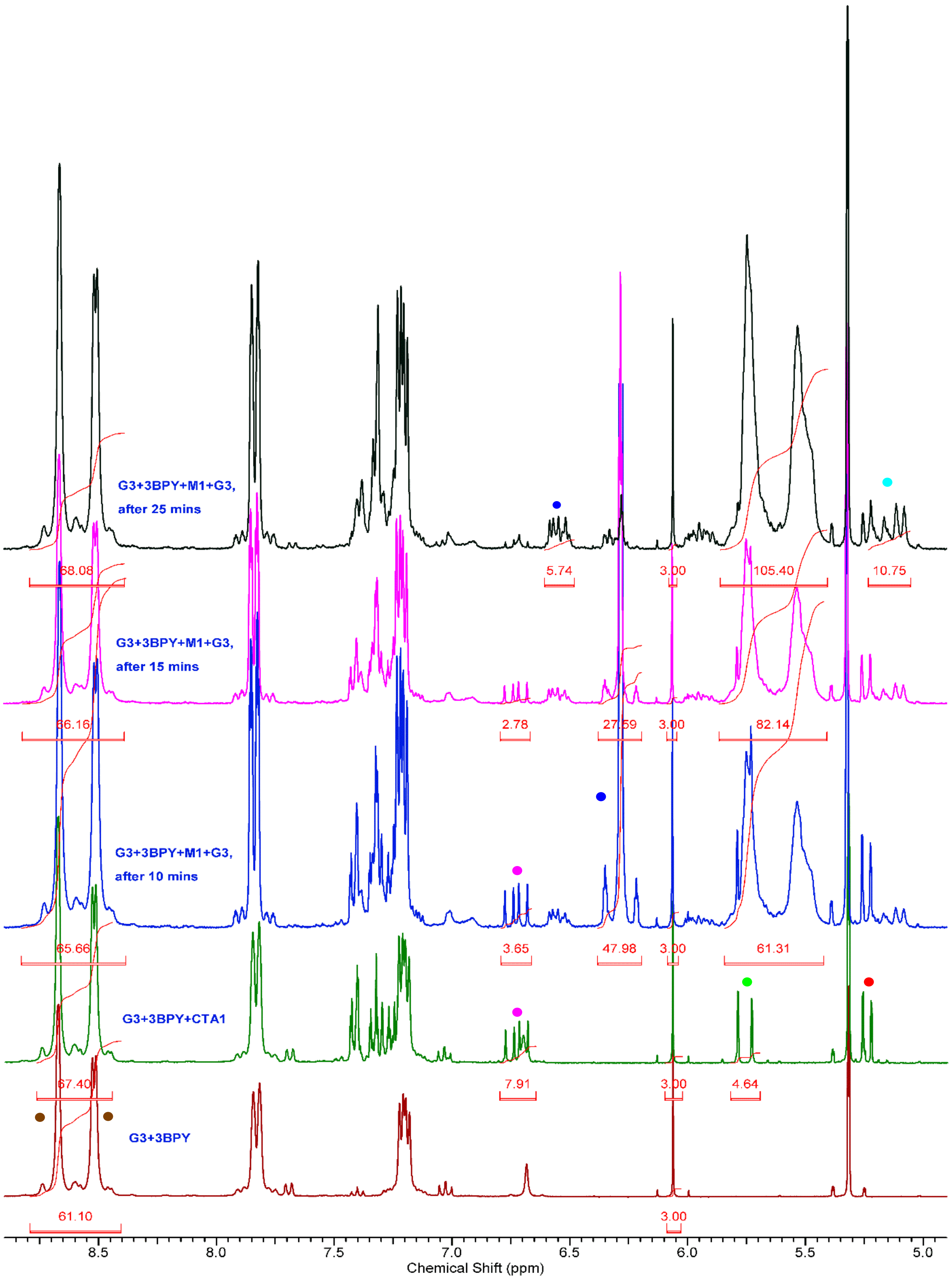

Fig. S6: Stacked ^{1}H NMR spectra of polymerization of **M1** in presence of **3BPY** using **CTA1** and **G3**. For clarity, only olefinic region is shown. It could be observed that both **M1** peak (at 6.25 ppm) and **CTA1**

peak (at 6.7 ppm) vanished after 25 mins implying a kinetically controlled chain transfer polymerization.

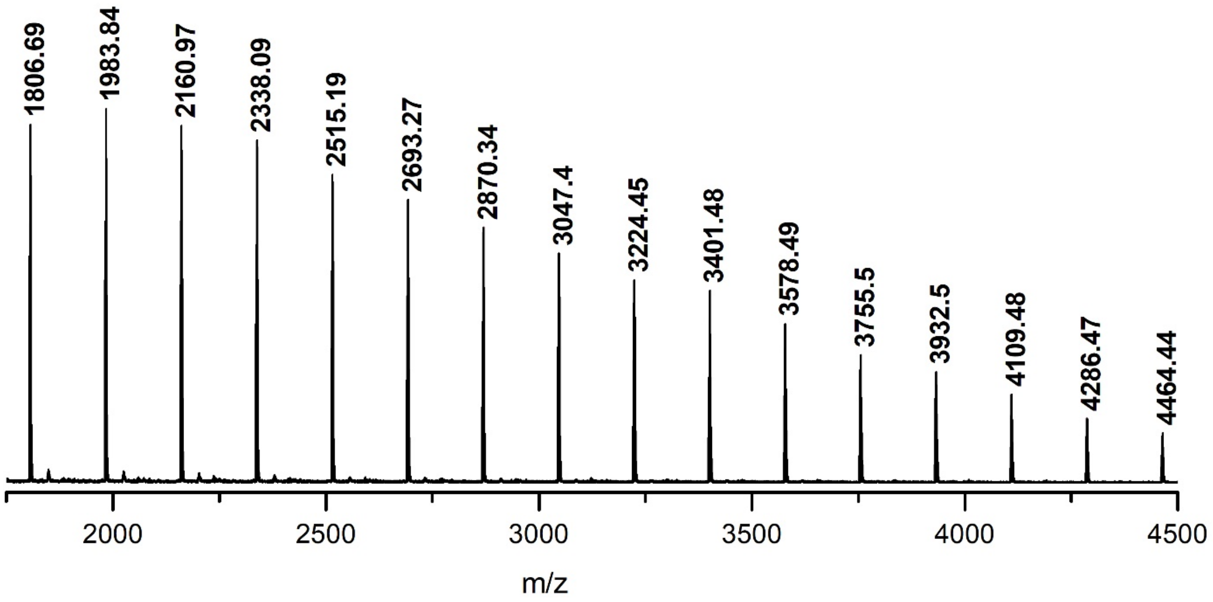

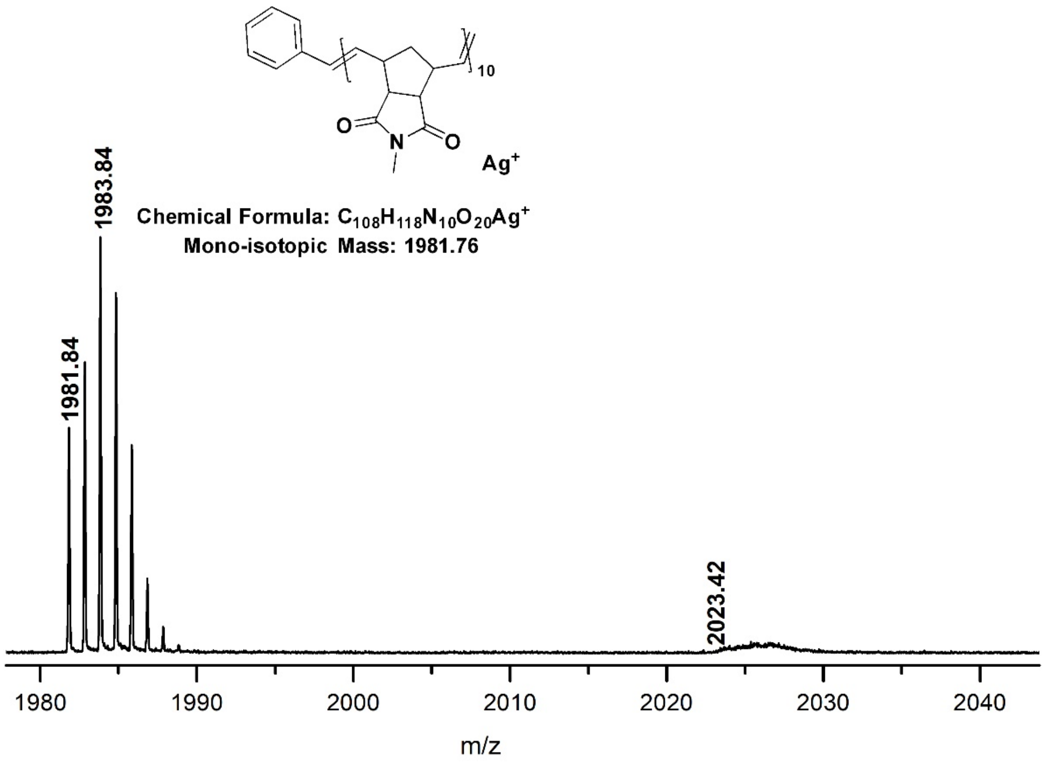

Fig. S7: MALDI-ToF mass spectrum (DCTB, AgTFA) of **P3**. Only one mass distribution (corresponding to polymer chain having a phenyl group on end and the methylene group on the other) was in good agreement of the proposed regioselective chain transfer of styrene. The very small broad distribution associated with a mass of 2023.42 did not match with any possible non-regioselective metathesis reaction.

General Procedure for catalytic ROMP:

In a round bottom flask equipped with a magnetic stirrer bar, monomer (**M1-M3**), 3-bromoopyridine, and chain transfer agent (**CTA1-10**) were dissolved in dry dichloromethane (non-degassed). Then, catalyst was dissolved in the same dichloromethane and added to the flask. The solution was stirred for required period of time after which few drops of ethyl vinyl ether was added to the flask. The resulting solution was concentrated under reduced pressure and further precipitated into methanol (except for **M3** where polymer was precipitated from cold pentane) two times to obtain respective polymers. A solid was obtained, filtered, and dried under a high vacuum. Respective yields of all the polymers were measured after drying under a high vacuum overnight. Typical polymerization temperature was between 20^0-25^0C.

Table S1: Polymerization data

Entry No.	Catalyst (Cat.)	CTA	Monomer (M)	Cat.:3BPY:CTA:M	Time	M_n (non-catalytic, M/Cat.) kDa	M_n (catalytic, M/CTA) kDa	M_n (SEC, $CHCl_3$) kDa	Dispersity (Đ)	Yield (%)
**P1**[a]	G3	**CTA1**	M1	1:-:15:300	<10 mins	53.2	3.6	4.3	1.83	82
**P3**[a]	G3	**CTA1**	M1	1:30:5:60	30 mins	10.7	2.2	2.6	1.71	78
**P4**	G3	**CTA2**	M1	1:30:50:500	7 h	88.5	1.9	2.8	1.84	76
**P5**	G3	**CTA2**	M1	1:30:50:1000	10 h	177	3.7	5.5	1.95	84
**P6**	G3	**CTA2**	M1	1:30:50:1500	14 h	266	5.5	7.1	2.12	84
**P7**	G3	**CTA2**	M1	1:30:50:2000	14 h	354	7.3	9.7	2.10	89
**P8**[a]	G2	**CTA2**	M1	1:30:20:200	3.5 h	35.4	1.9	2.5	2.20	85
**P9**[a]	HG-II	**CTA2**	M1	1:30:20:200	1 h	35.4	1.9	2.4	2.15	83
**P10**	G3	**CTA3**	M1	1:30:100:2000	18 h	354	3.7	5.3	2.05	88
**P11**[a]	HG-II	**CTA3**	M3	1:30:20:200	90 mins	57	3.0	5.0	1.80	85
**P12**	HG-II	**CTA4**	M3	1:30:100:2000	8 h	569	5.8	8.5	2.07	90
**P13**[a]	G3	**CTA5**	M1	1:30:20:200	1 h	35.5	1.9	3.0	1.86	86
**P14**	G3	**CTA5**	M1	1:30:20:1200	1 h	212.4	11	14.5	1.96	84
**P15**	G2	**CTA6**	M1	1:30:100:2000	10 h	569	6.0	7.0	1.72	75
**P16**	G3	**CTA7**	M1	1:30:100:3000	16 h	531	5.5	8.1	1.95	91
**P17**[a]	HG-II	**CTA8**	M1	1:30:20:200	50 mins	35.4	1.9	3.5	2.22	84

**P18**	HG-II	**CTA8**	M1	1:30:150:4500	2 h	797	5.6	10	2.10	91
**P19**	G3	**CTA9**	M1	1:30:20:200	85 mins	35.6	1.9	3.0	2.00	84
**P20**[a]	HG-II	**CTA10**	M1	1:40:20:200	1 h	35.4	1.9	3.0	1.93	82
**P21**	G2	**CTA6**	M2	1:30:200:6000	30 mins	564	2.9	20	2.2	90

a: Polymerization was carried out in an NMR tube (see below).

P4:

G3: 3 mg; **3BPY**: 16.1 mg; **CTA2**: 31 mg; **M1**: 301 mg; **DCM**: 5.6 mL.

P5:

G3: 3 mg; **3BPY**: 16.1 mg; **CTA2**: 31 mg; **M1**: 601 mg; **DCM**: 11.3 mL.

P6:

G3: 3 mg; **3BPY**: 16.1 mg; **CTA2**: 31 mg; **M1**: 902 mg; **DCM**: 17 mL.

^{1}H NMR (400 MHz, CHLOROFORM-d) δ ppm: 1.48 - 1.76 (m, 11 H), 2.00 - 2.21 (m, 7 H), 2.24 - 2.31 (m, 1 H), 2.66 - 2.84 (m, 9 H), 2.89 - 3.14 (m, 46 H), 3.18 - 3.36 (m, 7 H), 3.48 (s, 1 H), 5.44 - 5.59 (m, 8 H), 5.66 - 5.82 (m, 9 H), 6.53 - 6.53 (m, 1 H), 7.17 - 7.28 (m, 2 H).

P7:

G3: 3 mg; **3BPY**: 16.1 mg; **CTA2**: 31 mg; **M1**: 1.2 g; **DCM**: 23 mL.

P8:

G2: 2 mg; **3BPY**: 11.2 mg; **CTA2**: 8.7 mg; **M1**: 84 mg; **DCM-d$_2$**: 1.2 mL.

^{1}H NMR (300 MHz, CHLOROFORM-d) δ ppm: 5.09 - 5.28 (m, 2 H), 5.43 - 5.63 (m, 12 H), 5.65 - 5.84 (m, 13 H), 5.85 - 6.02 (m, 1 H), 6.32 (s, 1 H), 6.42 - 6.59 (m, 1 H), 7.38 - 7.53 (m, 2 H).

P9:

HG-II: 2 mg; **3BPY**: 15.1 mg; **CTA2**: 11.7 mg; **M1**: 113 mg; **DCM-d$_2$**: 1.2 mL.

P10:

G3: 4 mg; **3BPY**: 22 mg; **CTA3**: 74 mg; **M1**: 1.6 g; **DCM**: 31 mL.

^{1}H NMR (400 MHz, CHLOROFORM-d) δ ppm: 1.41 - 1.69 (m, 9 H), 1.95 - 2.15 (m, 7 H), 2.18 - 2.25 (m, 2 H), 2.59 - 2.78 (m, 9 H), 2.81 - 3.09 (m, 46 H), 3.12 - 3.29 (m, 6 H), 5.04 - 5.19 (m, 2 H), 5.38 - 5.55 (m, 8 H), 5.57 - 5.76 (m, 9 H), 6.96 - 7.01 (m, 1 H), 7.25 - 7.35 (m, 1 H).

P11:

HG-II: 1.5 mg; **3BPY**: 11.3 mg; **CTA3**: 7.8 mg; **M3**: 136 mg; **DCM-d$_2$**: 1.2 mL.

^{1}H NMR (400 MHz, CHLOROFORM-d) δ ppm: 2.23 - 2.36 (m, 3 H), 4.02 - 4.32 (m, 23 H), 4.82 - 5.11 (m, 2 H), 5.17 - 5.47 (m, 18 H), 6.32 - 6.46 (m, 1 H), 6.92 - 7.11 (m, 2 H), 7.46 - 7.60 (m, 1 H), 7.66 - 7.77 (m, 1 H).

P12:

HG-II: 1.5 mg; **3BPY**: 11.3 mg; **CTA4**: 32 mg; **M3**: 1.35 g; **DCM**: 9.5 mL.

^{1}H NMR (400 MHz, CHLOROFORM-d) δ ppm: 0.83 - 0.94 (m, 1 H), 1.06 - 1.20 (m, 2 H), 1.22 - 1.45 (m, 5 H), 1.66 - 1.81 (m, 5 H), 1.84 - 2.07 (m, 8 H), 2.39 - 2.67 (m, 2 H), 2.80 (dd, *J* = 16.69, 8.13 Hz, 3 H), 2.86 - 3.02 (m, 4 H), 3.03 - 3.22 (m, 3 H), 3.35 - 3.42 (m, 11 H), 3.45 - 3.74 (m, 40 H), 4.02 - 4.30 (m, 8 H), 5.17 - 5.45 (m, 37 H).

P13:

G3: 2 mg; **3BPY**: 11 mg; **CTA5**: 8 mg; **M1**: 80 mg; **DCM-d$_2$**: 1.1 mL.

P14:

G3: 2 mg; **3BPY**: 11 mg; **CTA5**: 8 mg; **M1**: 481 mg; **DCM**: 6.8 mL.

P15:

G2: 3 mg; **3BPY**: 17 mg; **CTA6**: 54 mg; **M1**: 1.25 g; **DCM**: 14 mL.

^{1}H NMR (400 MHz, CHLOROFORM-d) δ ppm: 1.48 - 1.72 (m, 9 H), 1.77 (s, 3 H), 2.00 - 2.21 (m, 7 H), 2.23 - 2.30 (m, 1 H), 2.66 - 2.83 (m, 9 H), 2.88 - 3.16 (m, 45 H), 3.18 - 3.36 (m, 7 H), 4.57 - 4.59 (m, 2 H), 5.10 - 5.25 (m, 1 H), 5.43 - 5.59 (m, 8 H), 5.65 - 5.82 (m, 8 H), 7.26 - 7.38 (m, 2 H).

P16:

G3: 3 mg; **3BPY**: 16 mg; **CTA7**: 75 mg; **M1**: 1.8 g; **DCM**: 25 mL.

^{1}H NMR (400 MHz, CHLOROFORM-d) δ ppm: 1.48 - 1.75 (m, 13 H), 2.02 - 2.24 (m, 7 H), 2.26 - 2.33 (m, 1 H), 2.68 - 2.86 (m, 9 H), 2.89 - 3.18 (m, 45 H), 3.21 - 3.37 (m, 6 H), 5.45 - 5.61 (m, 8 H), 5.71 - 5.83 (m, 33 H), 7.24 - 7.36 (m, 2 H).

P17:

HG-II: 2 mg; **3BPY**: 15 mg; **CTA8**: 19 mg; **M1**: 113 mg; **DCM-d_2**: 1.2 mL.

P18:

HG-II: 2 mg; **3BPY**: 15 mg; **CTA8**: 143 mg; **M1**: 2.54 g; **DCM**: 29 mL.

^{1}H NMR (300 MHz, CHLOROFORM-d) δ ppm: 1.45 - 1.78 (m, 12 H), 1.98 - 2.22 (m, 7 H), 2.22 - 2.39 (m, 2 H), 2.67 - 2.83 (m, 8 H), 2.86 - 3.13 (m, 44 H), 3.20 - 3.34 (m, 6 H), 3.49 (s, 1 H), 3.81 - 3.84 (m, 6 H), 5.30 - 5.40 (m, 1 H), 5.42 - 5.62 (m, 8 H), 5.66 - 5.84 (m, 8 H), 6.43 - 6.62 (m, 1 H), 7.20 (s, 1 H), 7.31 - 7.45 (m, 1 H).

P19:

G3: 2 mg; **3BPY**: 11 mg; **CTA9**: 7.4 mg; **M1**: 80 mg; **DCM-d_2**: 1.2 mL.

^{1}H NMR (400 MHz, CHLOROFORM-d) δ ppm: 3.87 - 3.98 (m, 3 H), 5.08 - 5.27 (m, 2 H), 5.40 - 5.65 (m, 12 H), 5.65 - 5.86 (m, 12 H), 5.86 - 6.03 (m, 1 H), 6.54 - 6.67 (m, 1 H), 7.33 - 7.49 (m, 2 H), 7.90 - 8.07 (m, 2 H).

P20:

HG-II: 2 mg; **3BPY**: 20.1 mg; **CTA10**: 8.5 mg; **M1**: 113 mg; **DCM**: 1.2 mL.

^{1}H NMR (400 MHz, CHLOROFORM-d) δ ppm: 1.47 - 1.73 (m, 13 H), 2.00 - 2.21 (m, 7 H), 2.24 - 2.31 (m, 1 H), 2.67 - 2.89 (m, 9 H), 2.91 - 3.15 (m, 42 H), 3.19 - 3.36 (m, 6 H), 4.66 - 4.71 (m, 2 H), 5.11 - 5.25 (m, 1 H), 5.44 - 5.60 (m, 7 H), 5.62 - 5.82 (m, 8 H), 5.87 - 6.00 (m, 1 H), 7.26 - 7.39 (m, 4 H).

P21:

G2: 3 mg; **3BPY**: 28 mg; **CTA6**: 108 mg; **M2**: 2 g; **DCM**: 43 mL.

^{1}H NMR (400 MHz, CHLOROFORM-d) δ ppm: 0.95 - 1.14 (m, 10 H), 1.28 - 1.47 (m, 18 H), 1.52 - 1.67 (m, 2 H), 1.70 - 1.99 (m, 28 H), 2.35 - 2.58 (m, 8 H), 2.69 - 2.89 (m, 11 H), 3.43 - 3.53 (m, 3 H), 5.14 - 5.43 (m, 18 H), 7.24 - 7.37 (m, 1 H).

Optimization for higher catalytic Polymerization:

Table S2

Entry No.	Catalyst (Cat.)	CTA	Monomer concentra-tion	Cat.:3BPY:CTA:M	Time	Monomer conversion	M_n (non-catalytic, M/Cat.) kDa	M_n (catalytic, M/CTA) kDa	M_n (SEC,$CHCl_3$) kDa	Đ	Yield (%)
**1**	**G2**	**CTA1**	0.5 (M)	1:50:500:10000	48 h	<10%	-	-	-	-	-
**2**	**G2**	**CTA1**	2 (M)	1:50:500:10000	48 h	35%	-	-	-	-	-
**3 (P22)**	**HG-II**	**CTA1**	1 (M)	1:50:500:5000	20 h	90%	900	1.8	3	1.72	82
**4 (P23)**	**HG-II**	**CTA1**	2.5 (M)	1:50:1000:15000	18 h	85%	2655	2.7	4	1.80	75

P22:

HG-II: 0.5 mg; **3BPY**: 6.3 mg; **CTA1**: 42 mg; **M1**: 705 mg; **DCM**: 4 mL.

P23:

HG-II: 0.5 mg; **3BPY**: 6.3 mg; **CTA1**: 83 mg; **M1**: 2.1 g; **DCM**: 4.8 mL.

NMR tube polymerization:

P8 :

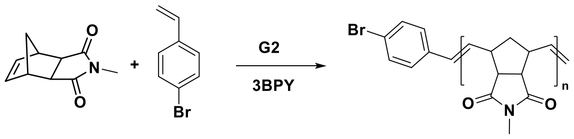

In an NMR tube, **G2** (1 equiv., 2.36 µmol, 2 mg), **CTA2** (20 equiv., 47.12 µmol, 8.6 mg) and 3-bromopyridine (**3BPY**) (30 equiv., 70.7 µmol, 11.2 mg) were dissolved 1.0 mL DCM-d_2 and ^{1}H NMR was recorded. 1,3,5 trimethoxybenzene was used as an internal standard. Then, **M1** (200 equiv., 471.2 µmol, 84 mg) dissolved in 0.2 mL DCM-d_2 was added to the NMR tube and the polymerization was followed by ^{1}H NMR spectroscopy over time.

CTA2 signal at 6.4 ppm decreased over time along with monomer (**M1**) peak at 6.25 ppm. Besides, integral of polymer backbone (5.4-5.75 ppm) increased during the polymerization. Full consumption of monomer was observed while the **CTA** consumption was >97%.

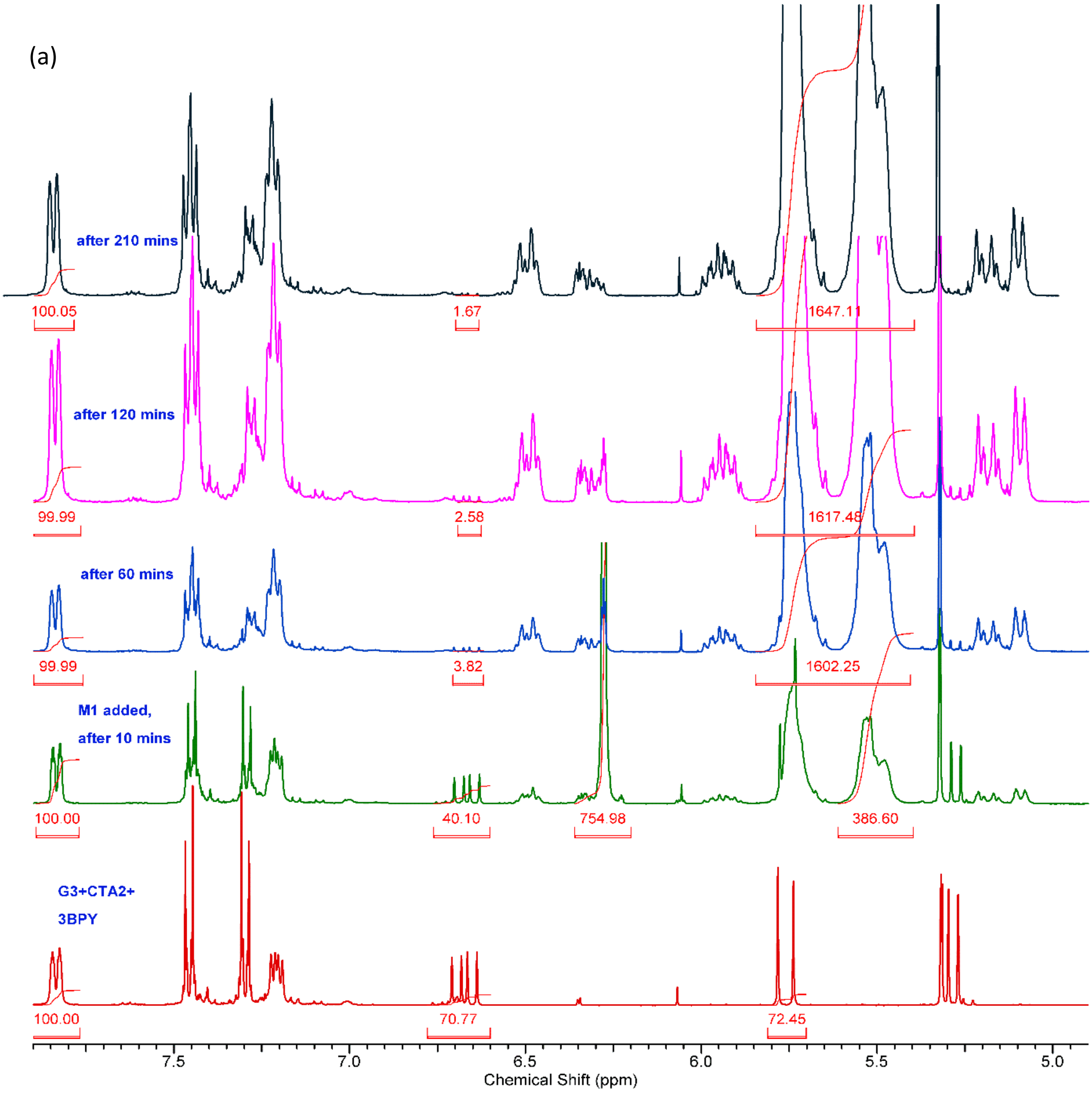

(b)

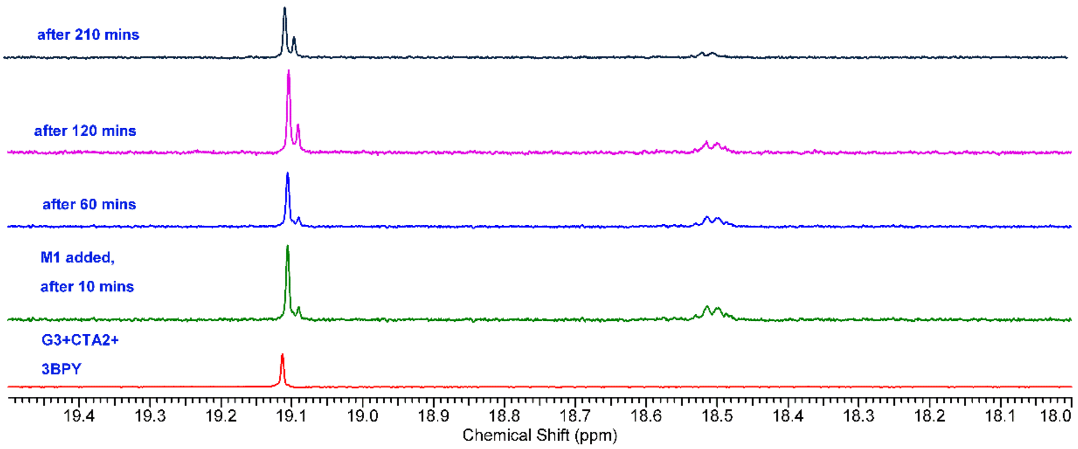

Fig. S8: ^{1}H NMR polymerization reaction of **M1** (200 equiv.) with **CTA2** (20 equiv.) using **G2** (1 equiv.). (a) stacked ^{1}H NMR spectra of olefinic region over time showing consumption of both monomer and CTA. (b) stacked ^{1}H NMR spectra of Ru-alkylidene region over time. Propagating Ru-alkylidene signal at 18.5 ppm while **G2**-benzylidene at 19.12 ppm and **G2**-Br benzylidene (due to chain transfer with **CTA2**) at 18.99 ppm. Due to poor initiation capacity of **G2**, most of the **G2**-benzylidene did not react.

P9:

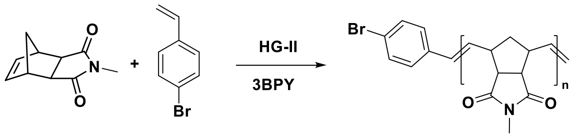

In an NMR tube, **HG-II** (1 equiv., 3.18 µmol, 2 mg), **CTA2** (20 equiv., 63.63 µmol, 11.7 mg) and 3-bromopyridine (**3BPY**) (30 equiv., 95.4 µmol, 15 mg) were dissolved 1.0 mL DCM-d_2 and ^{1}H NMR was recorded over time. 1,3,5 trimethoxybenzene was used as an internal standard. Interestingly, within 60 mins, **HG-II** (16.51 ppm) catalyst converted (78% conversion) to a new **G3-**Br benzylidene complex (19.15 ppm). Although for catalytic polymerization, pre-functionalization of any metathesis-based catalyst is not necessary, but this reaction showed how easily **HG-II** can be pre-functionalized using simple styrene derivatives.

After 60 mins, **M1** (200 equiv., 636.3 µmol, 113 mg) dissolved in 0.2 mL DCM-d_2 was added to the NMR tube and the polymerization was followed by ^{1}H NMR spectroscopy over time. **M1** was consumed fully and terminal double bond protons of **CTA2** (5.25 ppm and 5.75 ppm) transformed into polymer chain ends (5.05-5.20 ppm).

(a)

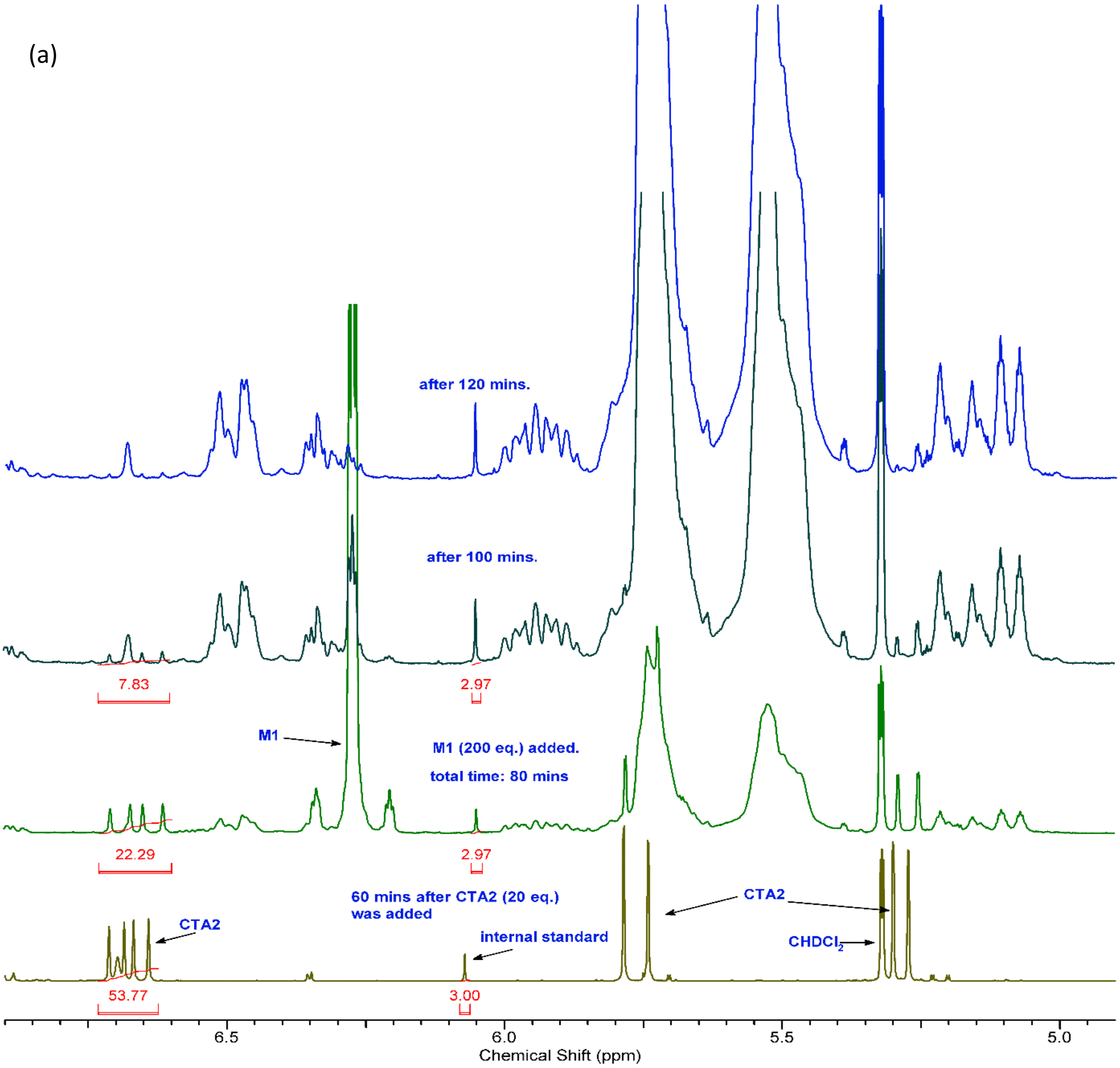

after 120 mins.
after 100 mins.
7.83
2.97
M1
M1 (200 eq.) added.
total time: 80 mins
22.29
2.97
60 mins after CTA2 (20 eq.) was added
CTA2
CTA2
internal standard
$CHDCl_2$
53.77
3.00
6.5
6.0
5.5
5.0
Chemical Shift (ppm)

(b)

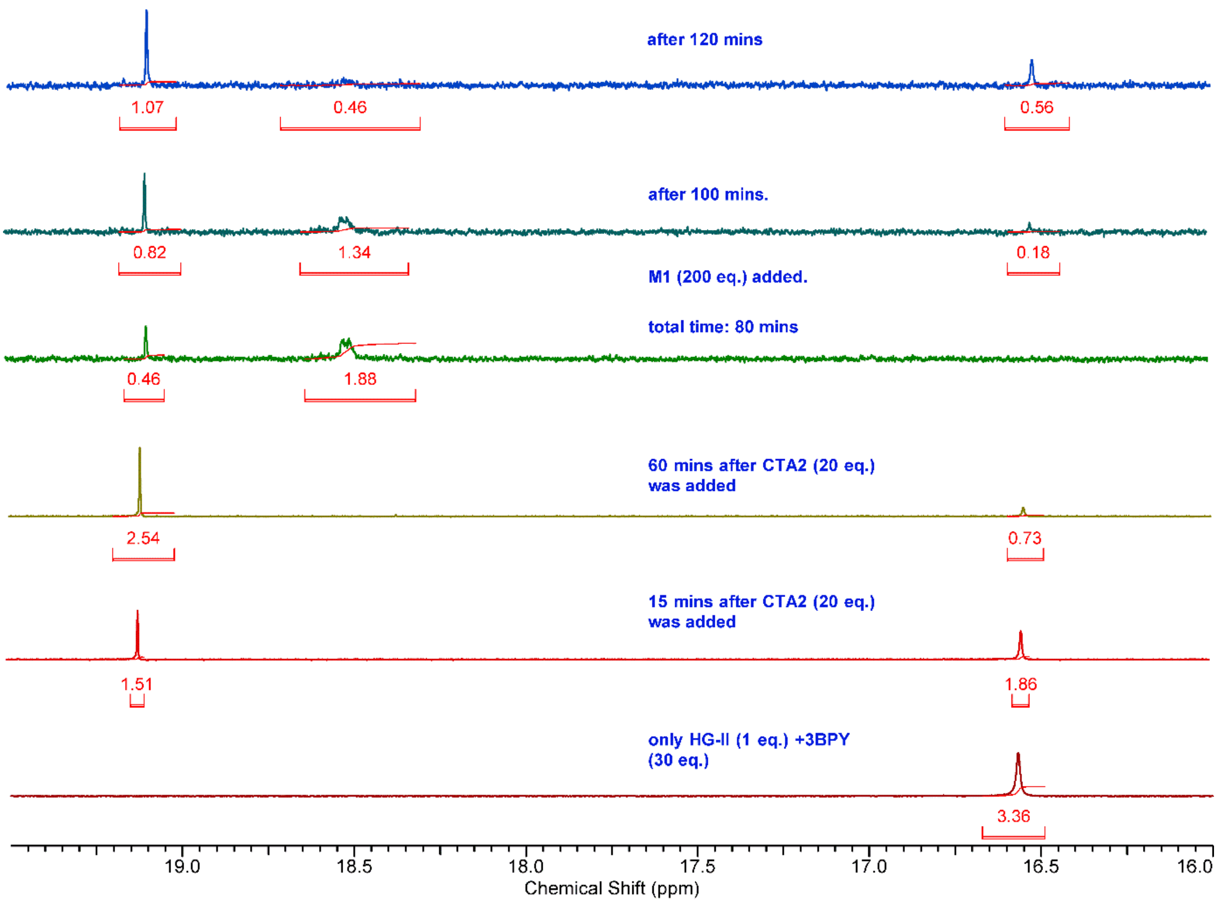

Fig. S9: ^{1}H NMR polymerization reaction of **M1** (200 equiv.) with **CTA2** (20 equiv.) using **HG-II** (1 equiv.). (a) stacked ^{1}H NMR spectra of olefinic region over time showing consumption of both monomer and CTA. (b) stacked ^{1}H NMR spectra of Ru-alkylidene region over time. **HG-II** (16.51 ppm) reacted with **CTA2** to produce a new **G3**-Br benzylidene complex (19.15 ppm) with 78% conversion after 60 mins. After **M1** was added both propagating alkylidene (18.52 ppm) and Ru-benzylidene complex (19.15 ppm) was observed after 80 mins. Chain transfer with 1-isopropoxy-2-vinylbenzene (generated after reaction of **HG-II** with **CTA2**) produced **HG-II** (16.51 ppm) back after 100 mins.

P11:

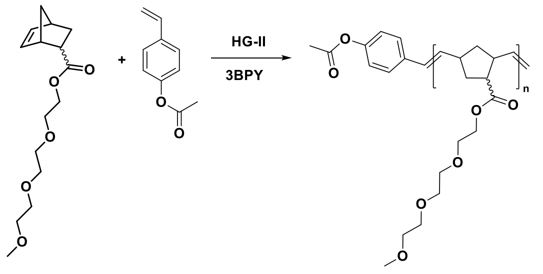

In an NMR tube, **HG-II** (1 equiv., 2.39 µmol, 1.5 mg), **CTA3** (20 equiv., 47.72 µmol, 7.75 mg) and 3-bromopyridine (**3BPY**) (30 equiv., 71.6 µmol, 11.3 mg) were dissolved 1.0 mL DCM-d_2 and ^{1}H NMR was recorded over time. 1,3,5 trimethoxybenzene was used as an internal standard. After 60 mins, **HG-II**

(16.51 ppm) catalyst converted (76% conversion) to a new **G3**-OAc benzylidene complex (19.05 ppm). After 60 mins, **M3** (200 equiv., 477.2 μmol, 136 mg) dissolved in 0.2 mL DCM-d_2 was added to the NMR tube and the polymerization was followed by ^{1}H NMR spectroscopy over time. Full consumption of monomer was observed while the **CTA** consumption was 86%.

(a)

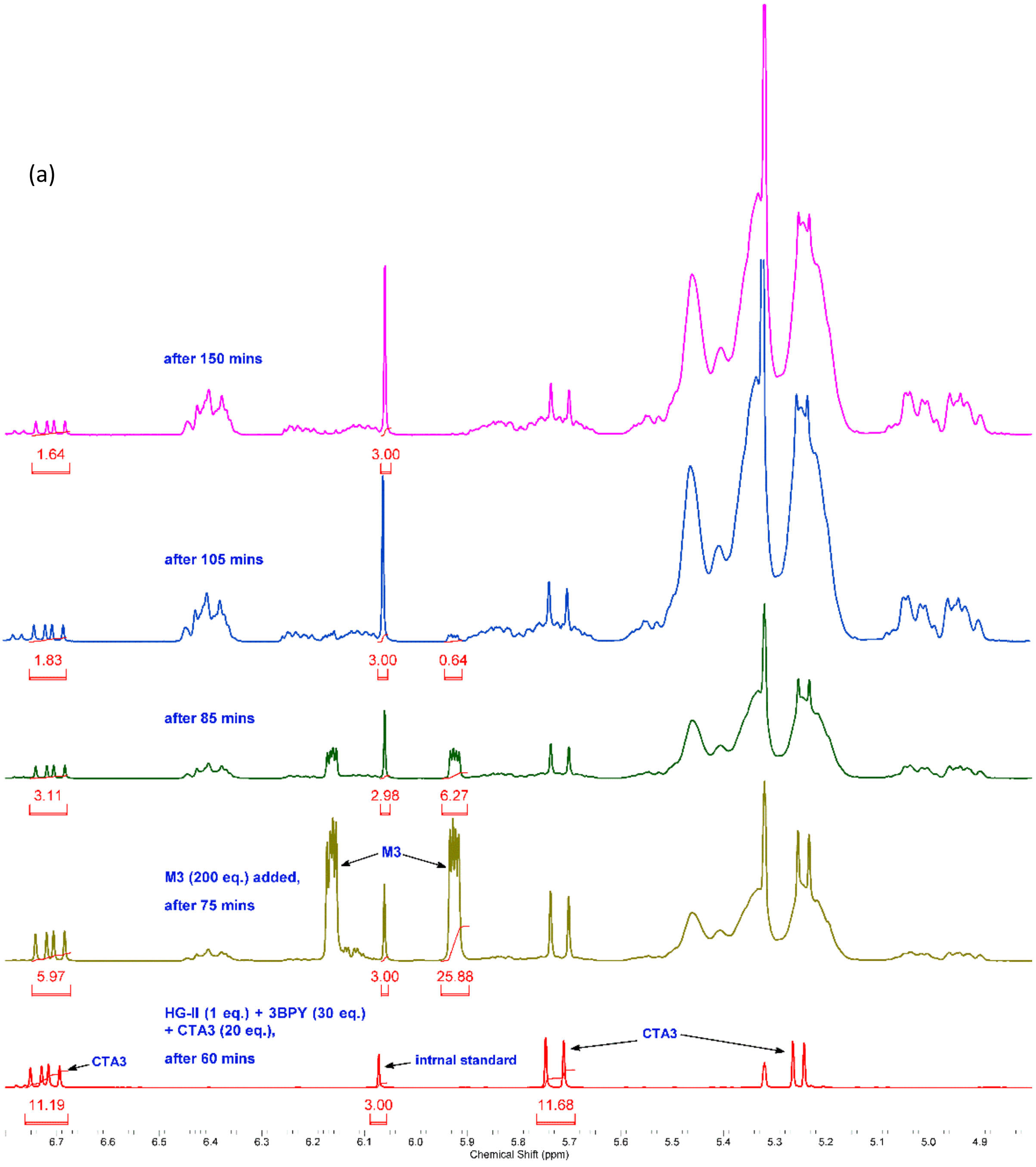

(b)

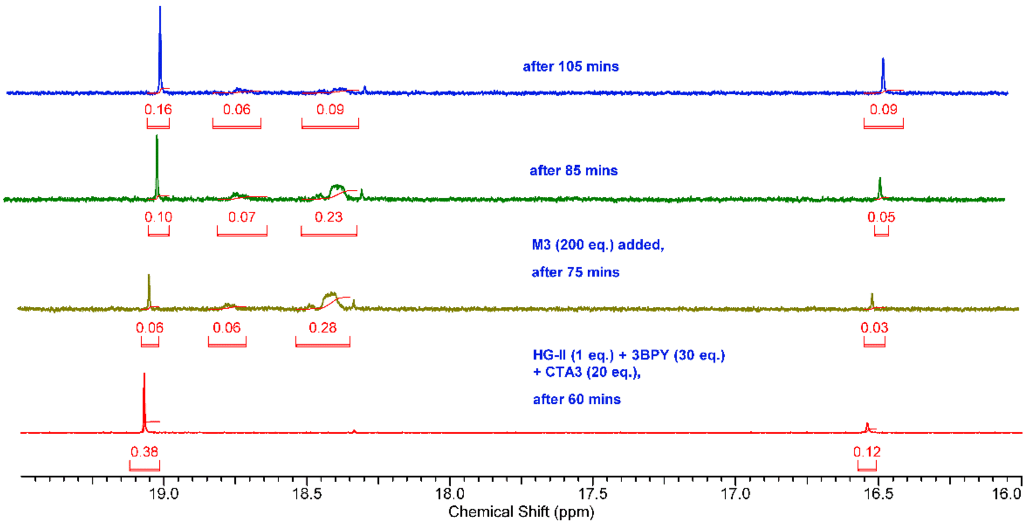

Fig. S10: ^{1}H NMR polymerization reaction of **M3** (200 equiv.) with **CTA3** (20 equiv.) using **HG-II** (1 equiv.). (a) stacked ^{1}H NMR spectra of olefinic region over time showing consumption of both monomer and CTA. (b) stacked ^{1}H NMR spectra of Ru-alkylidene region over time. **HG-II** (16.51 ppm) reacted with **CTA3** to produce a new **G3**-OAc benzylidene complex (19.15 ppm) with 76% conversion after 60 mins. After **M3** was added, both propagating alkylidene (18.52 ppm) and Ru-benzylidene complex (19.15 ppm) was observed after 75 mins. Chain transfer with 1-isopropoxy-2-vinylbenzene (generated after reaction of **HG-II** with **CTA3**) produced **HG-II** (16.51 ppm) back.

P13:

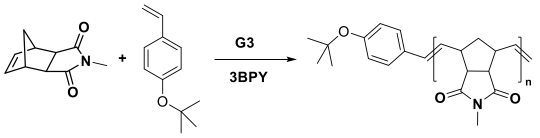

In an NMR tube, **G3** (1 equiv., 2.26 µmol, 2 mg), **CTA5** (20 equiv., 45.22 µmol, 8 mg) and 3-bromopyridine (**3BPY**) (30 equiv., 68 µmol, 11 mg) were dissolved 1.0 mL DCM-d_2 and ^{1}H NMR was recorded over time. 1,3,5 trimethoxybenzene was used as an internal standard. After 20 mins, **G3** (19.12 ppm) catalyst converted (100% conversion) to a new **G3**-O^{t}Bu benzylidene complex (18.66 ppm). After 20 mins, **M1** (200 equiv., 452.2 µmol, 81 mg) dissolved in 0.2 mL DCM-d_2 was added to the NMR tube and the polymerization was followed by ^{1}H NMR spectroscopy over time. Full consumption of monomer was observed while the **CTA** consumption was 89%.

(a)

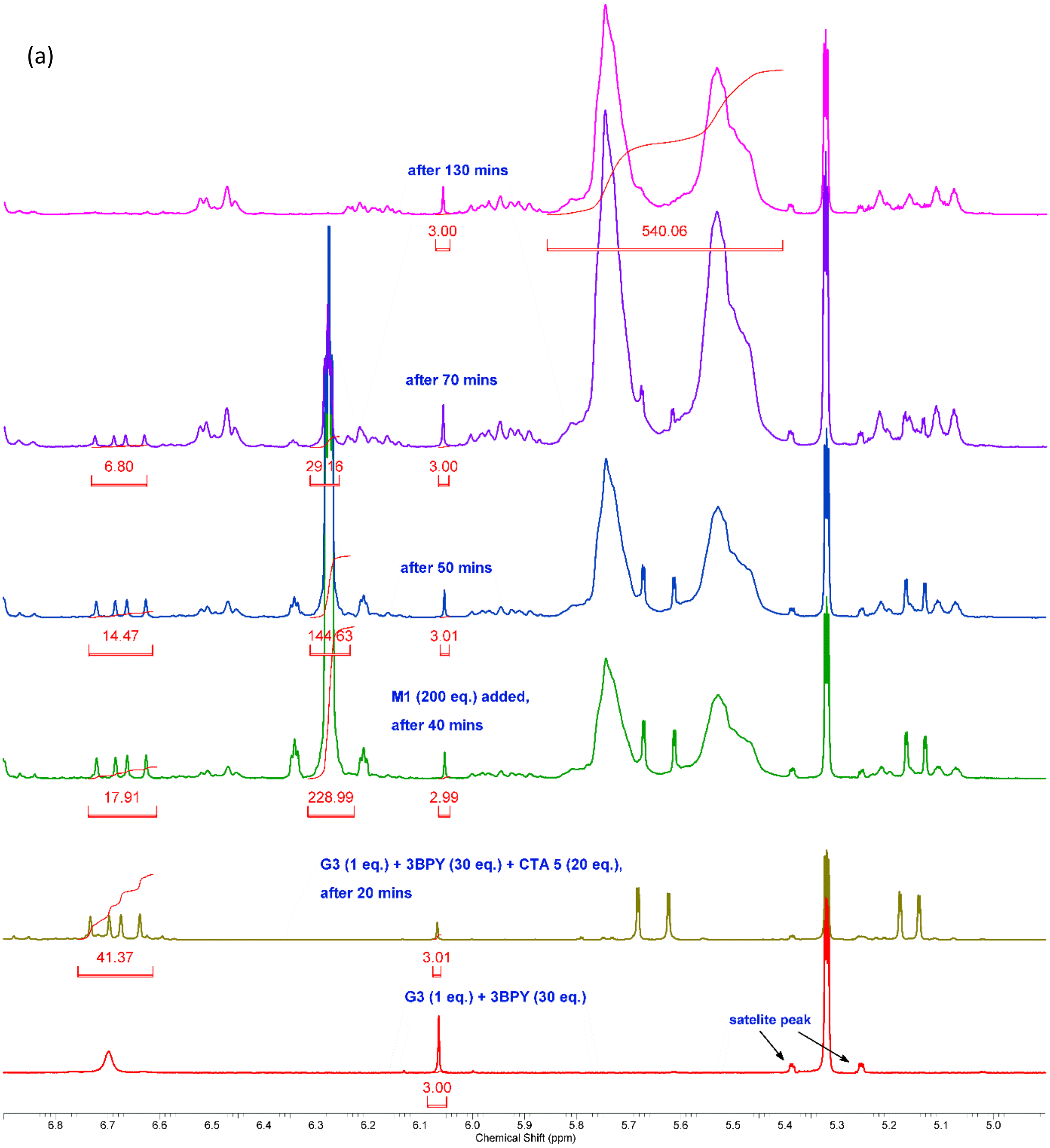

after 130 mins
3.00
540.06
after 70 mins
6.80
29.16
3.00
after 50 mins
14.47
144.63
3.01
M1 (200 eq.) added,
after 40 mins
17.91
228.99
2.99
G3 (1 eq.) + 3BPY (30 eq.) + CTA 5 (20 eq.),
after 20 mins
41.37
3.01
G3 (1 eq.) + 3BPY (30 eq.)
satelite peak
3.00
6.8 6.7 6.6 6.5 6.4 6.3 6.2 6.1 6.0 5.9 5.8 5.7 5.6 5.5 5.4 5.3 5.2 5.1 5.0
Chemical Shift (ppm)

(b)

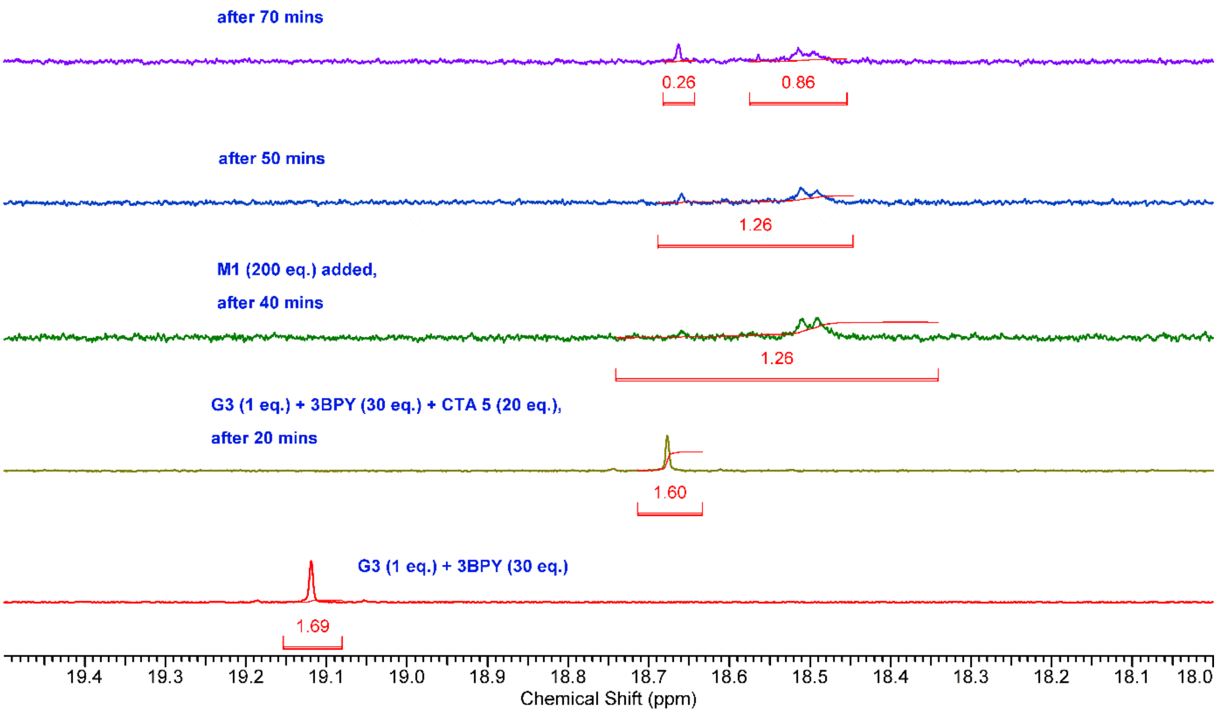

Fig. S11: ^{1}H NMR polymerization reaction of **M1** (200 equiv.) with **CTA5** (20 equiv.) using **G3** (1 equiv.). (a) stacked 1H NMR spectra of olefinic region over time showing consumption of both monomer and CTA. (b) stacked ^{1}H NMR spectra of Ru-alkylidene region over time. **G3** (19.12 ppm) reacted with **CTA5** to produce a new **G3**-OtBu benzylidene complex (18.66 ppm) with 100% conversion after 20 mins. After **M1** was added both propagating alkylidene (18.50 ppm) and Ru-benzylidene complex (18.66 ppm) was observed after 50 mins.

P17:

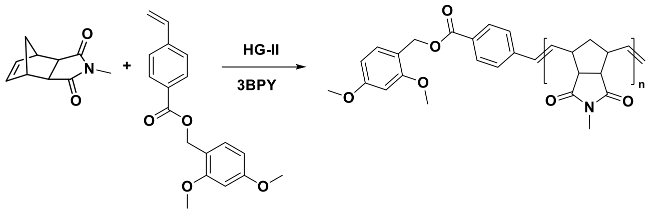

In an NMR tube, **HG-II** (1 equiv., 3.18 µmol, 2 mg), **CTA8** (20 equiv., 63.63 µmol, 19 mg) and 3-bromopyridine (**3BPY**) (30 equiv., 95.4 µmol, 15 mg) were dissolved 1.0 mL DCM-d_2 and ^{1}H NMR was recorded over time. 1,3,5 trimethoxybenzene was used as an internal standard. After 60 mins, **HG-II** (16.51 ppm) catalyst converted (62% conversion) to a new **G3** type Ru-benzylidene complex (19.51 ppm). After 60 mins, **M1** (200 equiv., 636.3 µmol, 113 mg) dissolved in 0.2 mL DCM-d_2 was added to the NMR tube and the polymerization was followed by ^{1}H NMR spectroscopy over time. Monomer consumption was >99% observed while the **CTA** consumption was 84.2%.

(a)

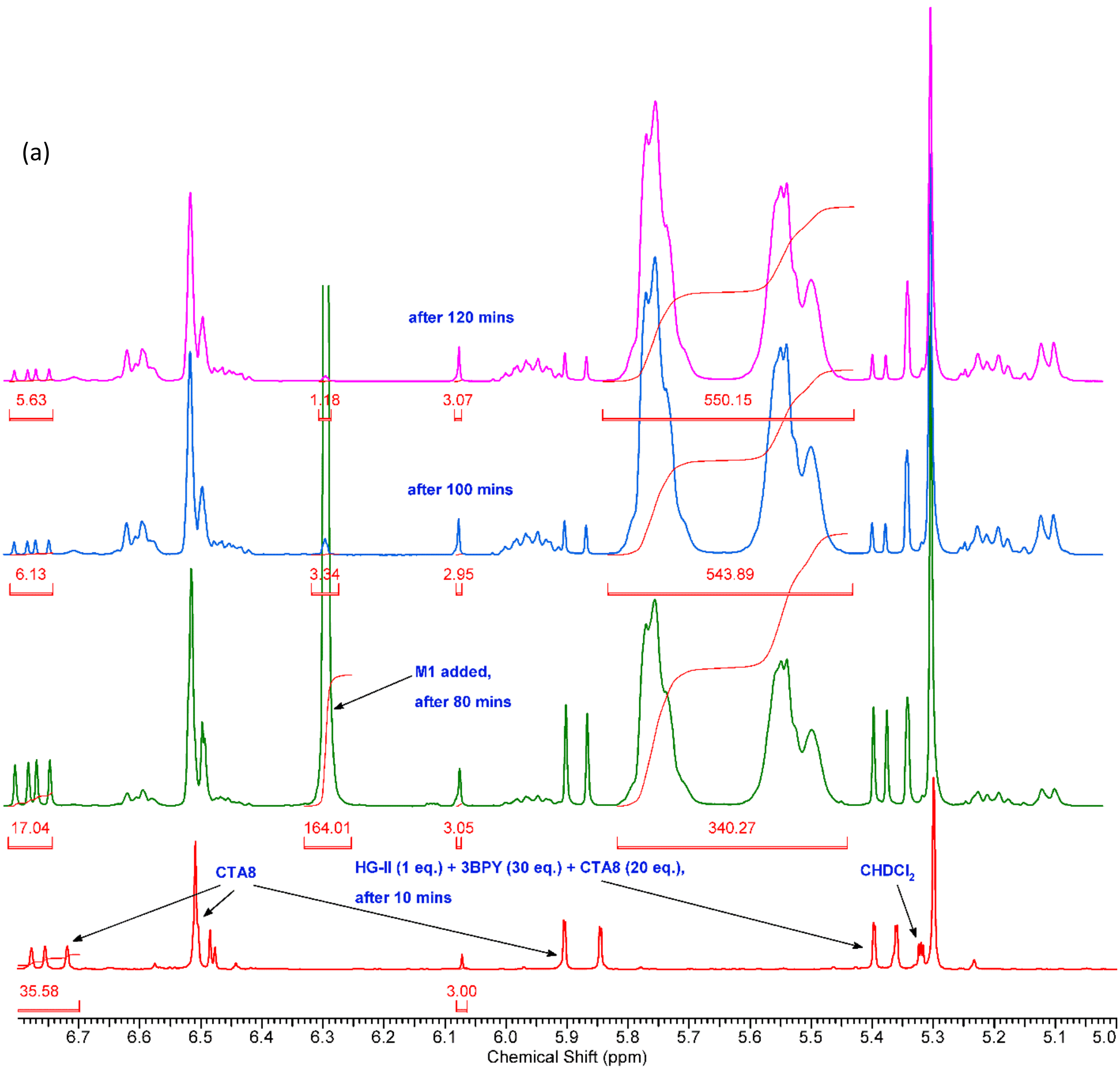
after 120 mins
5.63
1.18
3.07
550.15
after 100 mins
6.13
3.34
2.95
543.89
M1 added,
after 80 mins
17.04
164.01
3.05
340.27
CTA8
HG-II (1 eq.) + 3BPY (30 eq.) + CTA8 (20 eq.),
after 10 mins
CHDCl2
35.58
3.00
6.7 6.6 6.5 6.4 6.3 6.2 6.1 6.0 5.9 5.8 5.7 5.6 5.5 5.4 5.3 5.2 5.1 5.0
Chemical Shift (ppm)

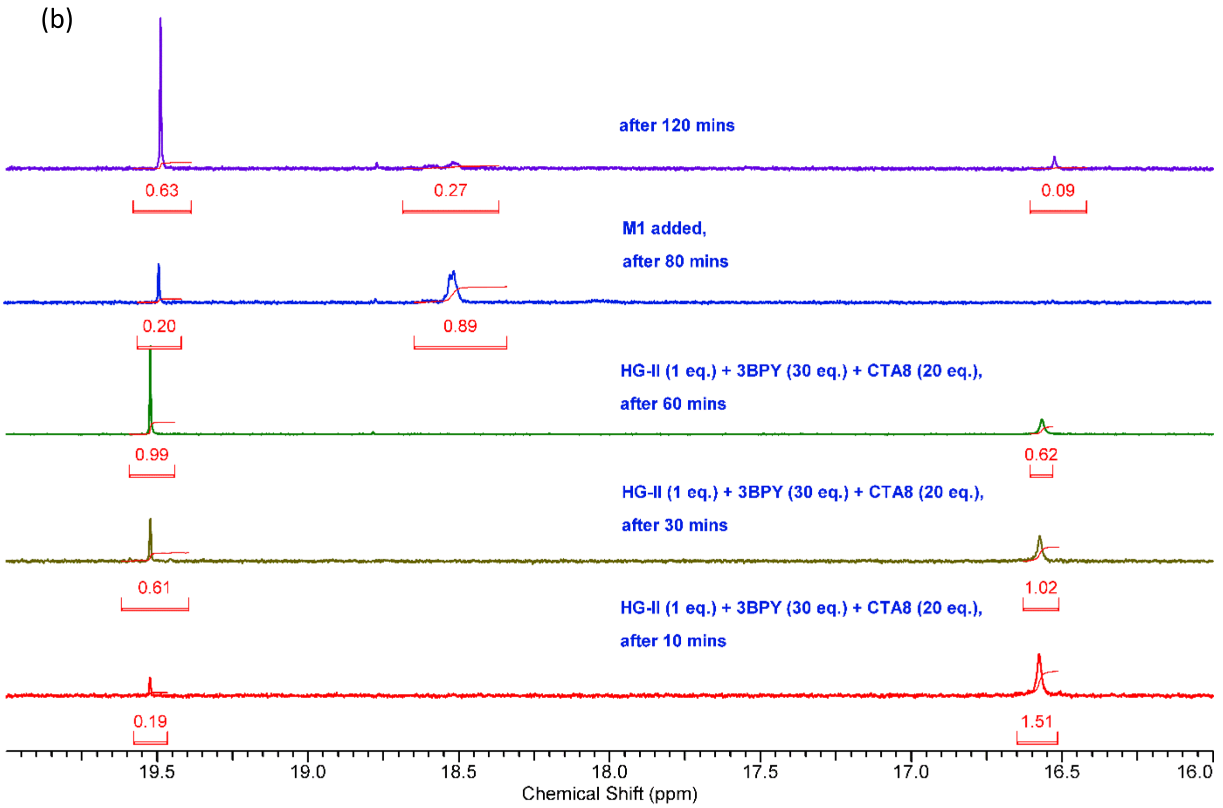

Fig. S12: ^{1}H NMR polymerization reaction of **M1** (200 equiv.) with **CTA8** (20 equiv.) using **HG-II** (1 equiv.). (a) stacked ^{1}H NMR spectra of olefinic region over time showing consumption of both monomer and CTA. (b) stacked ^{1}H NMR spectra of Ru-alkylidene region over time. **HG-II** (16.51 ppm) reacted with **CTA8** to produce a new **G3**-CO_2DMB benzylidene complex (19.51 ppm) with 62% conversion after 60 mins. After **M1** was added both propagating alkylidene (18.52 ppm) and **G3**-CO_2DMB benzylidene complex (19.51 ppm) was observed after 80 mins. Chain transfer with 1-isopropoxy-2-vinylbenzene (generated after reaction of **HG-II** with **CTA8**) produced **HG-II** (16.51 ppm) back.

P20:

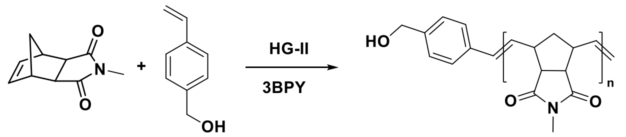

In an NMR tube, **HG-II** (1 equiv., 3.18 µmol, 2 mg), **CTA10** (20 equiv., 63.63 µmol, 8.6 mg) and 3-bromopyridine (**3BPY**) (40 equiv., 127.3 µmol, 20 mg) were dissolved 1.0 mL DCM-d_2 and ^{1}H NMR was recorded over time. 1,3,5 trimethoxybenzene was used as an internal standard. After 65 mins, **HG-II** (16.51 ppm) catalyst converted (73% conversion) to a new **G3** type Ru-benzylidene complex (19.06 ppm). After 65 mins, **M1** (200 equiv., 636.3 µmol, 113 mg) dissolved in 0.2 mL DCM-d_2 was added to the NMR tube and the polymerization was followed by ^{1}H NMR spectroscopy over time. Monomer consumption was >99% observed while the **CTA** consumption was 95%.

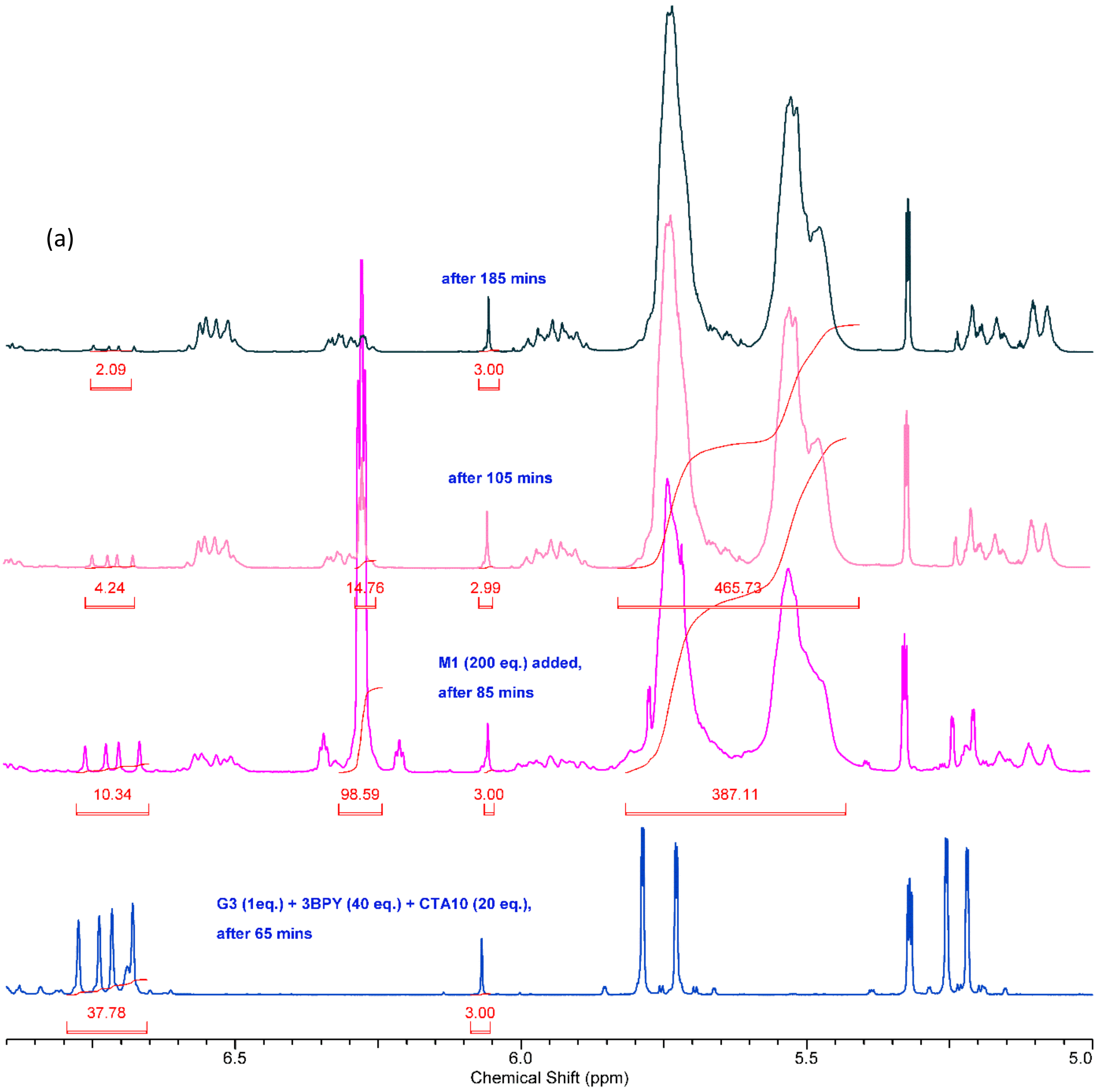

(a)
after 185 mins
2.09
3.00
after 105 mins
4.24
14.76
2.99
465.73
M1 (200 eq.) added,
after 85 mins
10.34
98.59
3.00
387.11
G3 (1eq.) + 3BPY (40 eq.) + CTA10 (20 eq.),
after 65 mins
37.78
3.00
6.5
6.0
5.5
5.0
Chemical Shift (ppm)

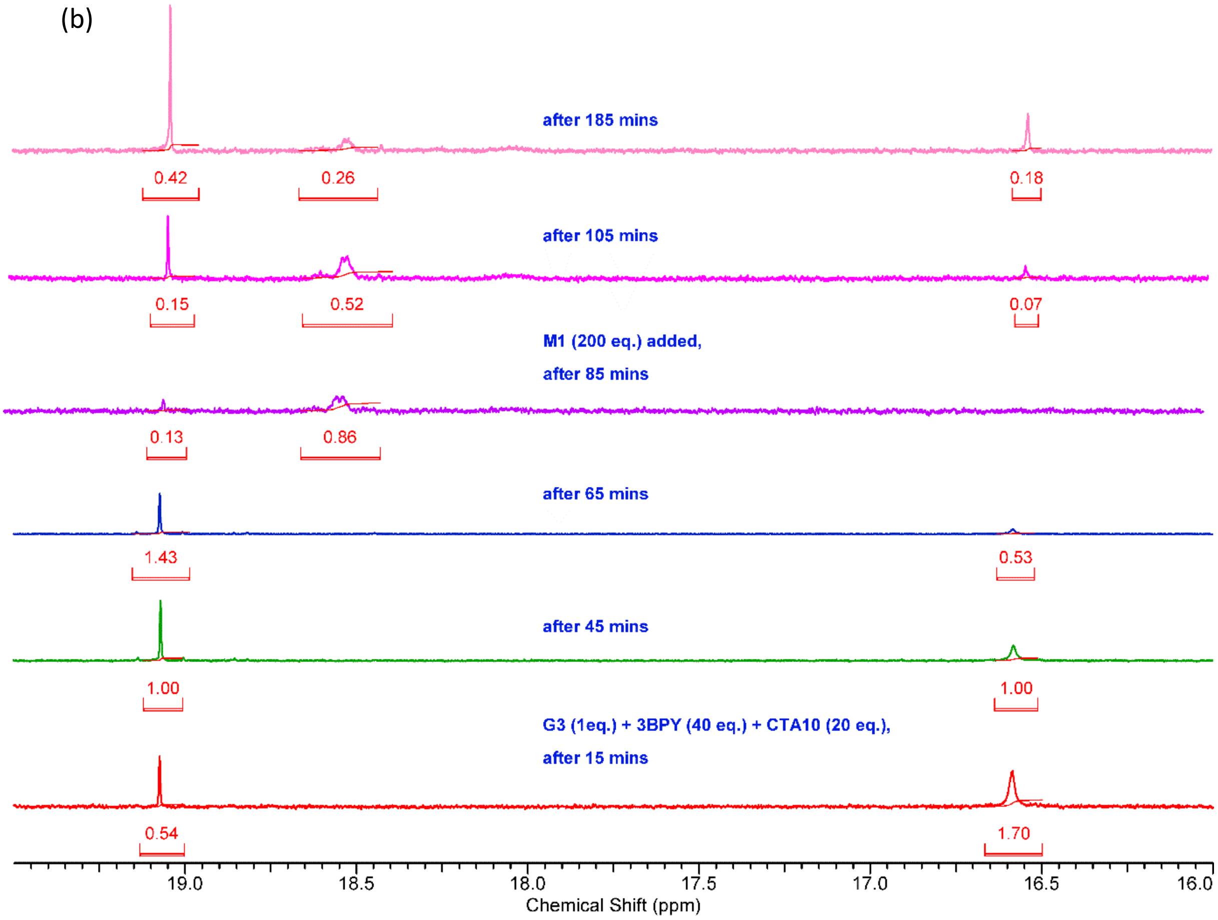

Fig. S13: ^{1}H NMR polymerization reaction of **M1** (200 equiv.) with **CTA10** (20 equiv.) using **HG-II** (1 equiv.). (a) stacked ^{1}H NMR spectra of olefinic region over time showing consumption of both monomer and CTA. (b) stacked ^{1}H NMR spectra of Ru-alkylidene region over time. **HG-II** (16.51 ppm) reacted with **CTA10** to produce a new **G3**-OH benzylidene complex (19.06 ppm) with 73% conversion after 65 mins. After **M1** was added both propagating alkylidene (18.52 ppm) and **G3**-OH benzylidene complex (19.06 ppm) was observed after 80 mins. Chain transfer with 1-isopropoxy-2-vinylbenzene (generated after reaction of **HG-II** with **CTA10**) produced **HG-II** (16.51 ppm) back.

P29:

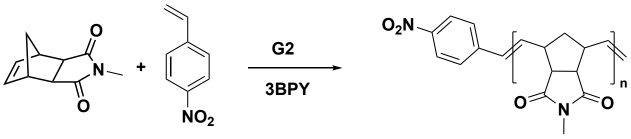

In an NMR tube, **M1** (400 equiv., 471.2 µmol, 84 mg), **CTA11** (20 equiv., 23.56 µmol, 3.5 mg) and 3-bromopyridine (**3BPY**) (30 equiv., 35.34 µmol, 5.6 mg) were dissolved 1.0 mL $CDCl_3$ and ^{1}H NMR was recorded over time. 1,3,5 trimethoxybenzene was used as an internal standard. Then, **G2** (1 equiv., 1.17 µmol, 1 mg) dissolved in 0.2 mL $CDCl_3$ was added to the NMR tube and the polymerization was followed by ^{1}H NMR spectroscopy over time. Monomer consumption was >99% observed while the **CTA** consumption was 88%.

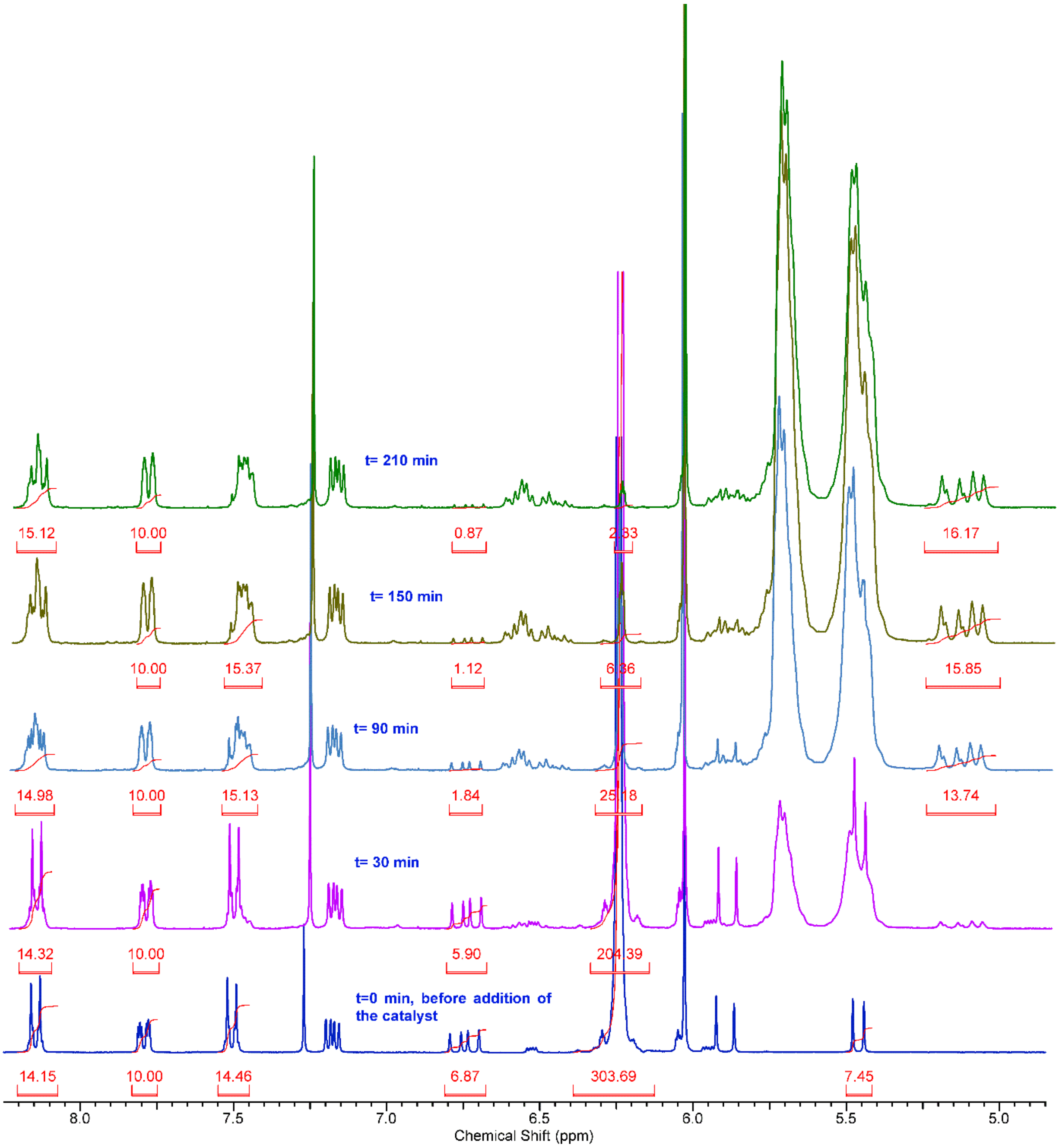

Fig. S14: ^{1}H NMR polymerization reaction of **M1** (400 equiv.) with **CTA11** (20 equiv.) using **G2** (1 equiv.). Stacked ^{1}H NMR spectra of olefinic region over time showing consumption of both monomer (6.25 ppm) and CTA (6.55 ppm). Peak at 7.8 ppm corresponds to the 3BPY. Signal at 6.05 ppm is of 1,3,5 trimethoxybenzene. Due to overlapping peaks at 1,3,5 trimethoxybenzene region, 3BPY was chosen as a standard for integration.

P30 :

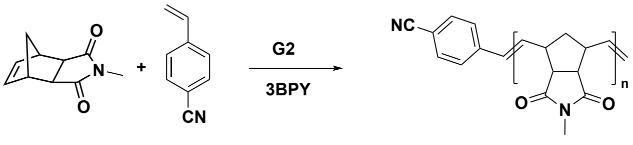

In an NMR tube, **M1** (400 equiv., 471.2 µmol, 84 mg), **CTA12** (20 equiv., 23.56 µmol, 3.0 mg) and 3-bromopyridine (**3BPY**) (30 equiv., 35.34 µmol, 5.6 mg) were dissolved 1.0 mL $CDCl_3$ and ^1H NMR was recorded over time. 1,3,5 trimethoxybenzene was used as an internal standard. Then, **G2** (1 equiv., 1.17 µmol, 1 mg) dissolved in 0.2 mL $CDCl_3$ was added to the NMR tube and the polymerization was followed by ^1H NMR spectroscopy over time. Monomer consumption was 97% while the **CTA** consumption was 85%.

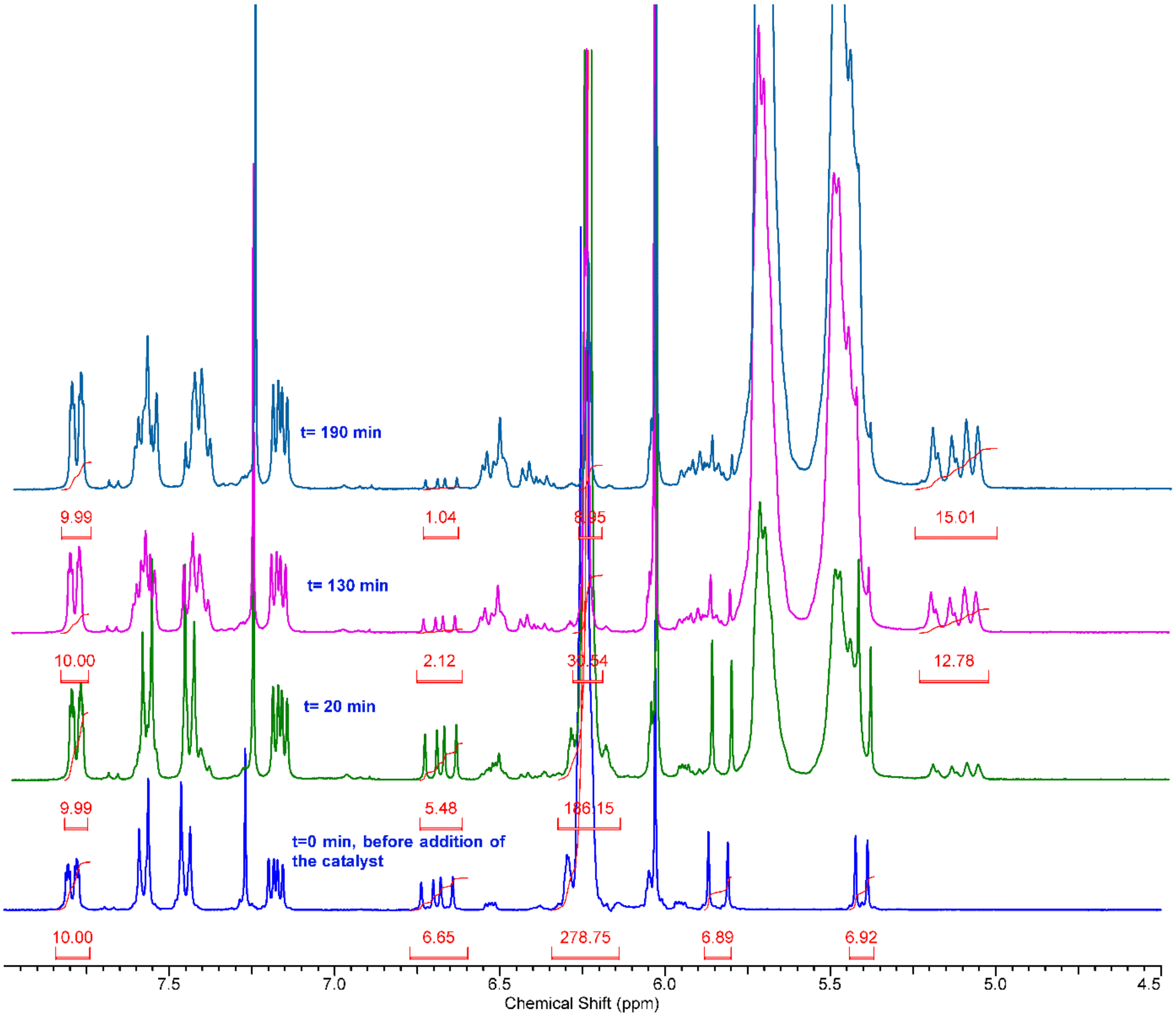

Fig. S15: ^1H NMR polymerization reaction of **M1** (400 equiv.) with **CTA12** (20 equiv.) using **G2** (1 equiv.). Stacked ^1H NMR spectra of olefinic region over time showing consumption of both monomer (6.25 ppm) and CTA (6.53 ppm). Peak at 7.8 ppm corresponds to the 3BPY. Signal at 6.05 ppm is of 1,3,5 trimethoxybenzene. Due to overlapping peaks at 1,3,5 trimethoxybenzene region, 3BPY was chosen as a standard for integration.

Amine and acid functional ROMP polymers:

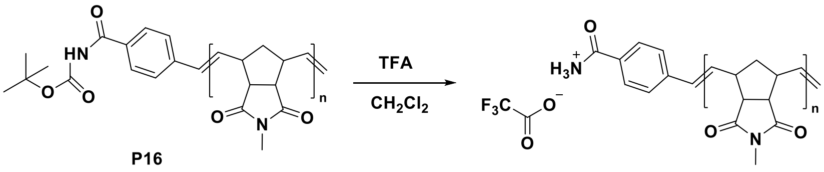

In a round bottom flask, **P16** (1.136 g) was dissolved in 6 mL dry DCM and cooled to 0^0 C. To this cooled mixture, 3 mL trifluoroacetic acid was added dropwise and the solution was stirred at room temperature overnight. Next, the solution was concentrated under reduced pressure and precipitated twice into cold methanol to obtain protonated amine functional ROMP polymer, **P24** with a yield of 90% (1.00 g).

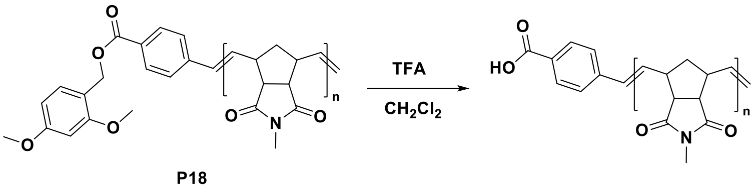

In a round bottom flask, **P18** (1.0 g) was dissolved in 4 mL dry DCM and cooled to 0^0 C. To this cooled mixture, few drops of tri-isopropyl silane and 0.2 mL trifluoroacetic acid (5 vol %) was added dropwise and the solution was stirred at room temperature overnight. Next, the solution was concentrated under reduced pressure and precipitated twice into cold methanol to obtain carboxylic acid functional ROMP polymer, **P25** with a yield of 91% (894 mg).

Catalytic ROMP in an Erlenmeyer flask:

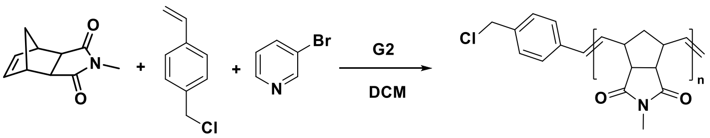

A magnetic stirrer bar equipped Erlenmeyer flask was charged with **M1** (10000 equiv., 11.78 mmol, 2.1 g). To it, 11 mL of non-dry, non-degassed dichloromethane solution (see the image below) was added in open air condition. **CTA6** (200 equiv., 0.235 mmol, 36 mg) and 3-bromopyridene (30 equiv., 0.035 mmol, 5.6 mg) were also dissolved in the same dichloromethane and added to the flask and stirred. **G2** (1 equiv., 0.0012 mmol, 1 mg) predissolved in the DCM was then added to the flask quickly. The flask was covered with an aluminum foil to avoid evaporation of solvent. After 3h, >98% of monomer (**M1**) was observed via ^{1}H NMR spectroscopy. Then, the polymerization was quenched with few drops of ethyl vinyl ether. Solvent was evaporated under reduced pressure and the concentrated DCM solution was added dropwise to methanol to precipitate the resulting polymer, **P26** as a white solid (see below). Polymer was dried under high vacuum to obtain a yield of 90%.

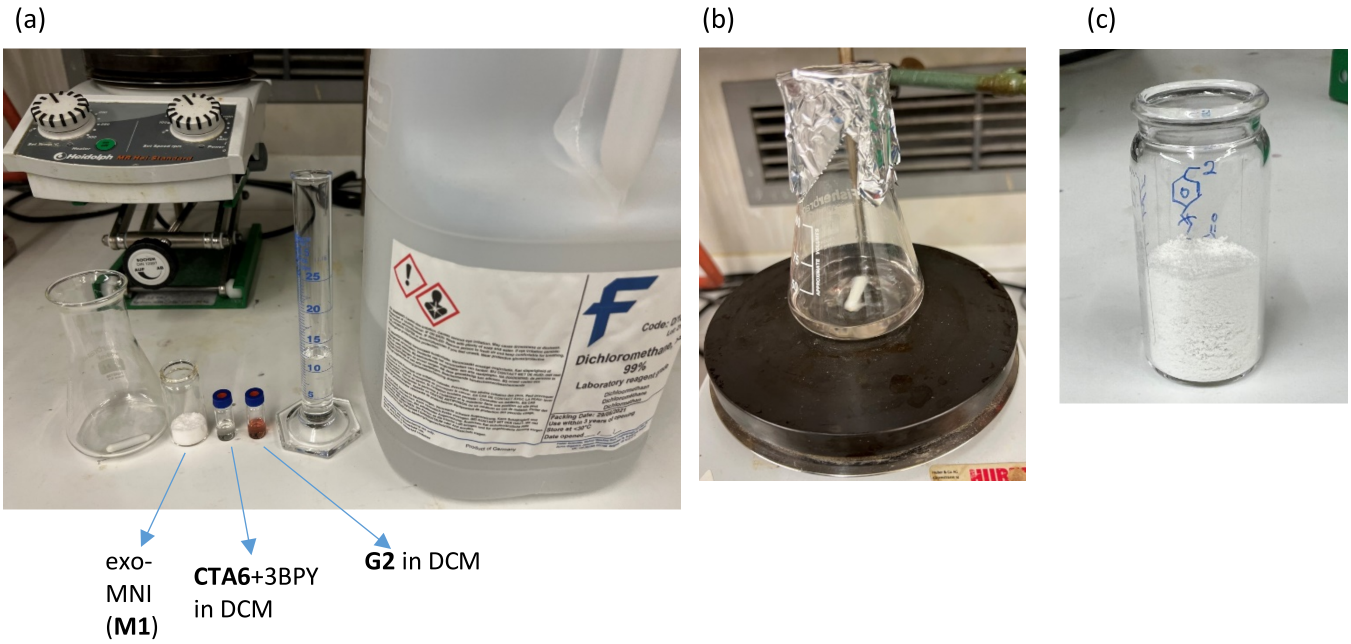

Fig. S16: Reaction set up for catalytic ROMP under non-degassed condition. (a) An Erlenmeyer flask equipped with magnetic stirrer bar, **M1** was weighed in a 10 mL vial, **CTA6** and 3-bromopyridine (**3BPY**) along with **G2** catalyst were dissolved in the dichloromethane (Laboratory reagent grade as shown). (b) **M1**, **CTA6** and **3BPY** were dissolved in DCM followed by addition of **G2**. An aluminum foil was used to cover up the flask. (c) Picture of the precipitated polymer.

Antimicrobial ROMP polymer:

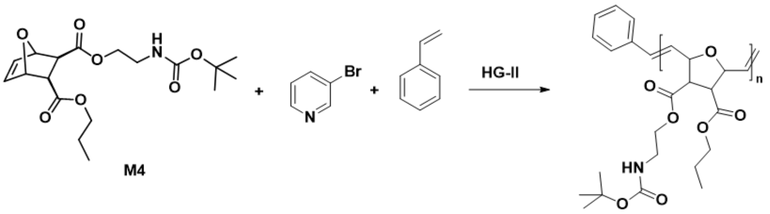

In an oven-dried schlenk flask, **M4** (1260 equiv., 4.015 mmol, 1.49 g), **3BPY** (40 equiv., 0.128 mmol, 20 mg) and styrene (**CTA1**) (150 equiv., 0.48 mmol, 50 mg) were mixed in 16 mL dry DCM. The resulting solution was degassed through three freeze-thaw pump cycle. Then, **HG-II** (1 equiv., 0.0032 mmol, 2 mg) dissolved in 0.2 mL dry degassed DCM was added to the Schlenk flask in one shot. Full consumption of monomer was observed after two and half hour by ^{1}H NMR spectroscopy. Few drops of ethyl vinyl ether was added to quench the active catalyst. DCM was evaporated under reduced pressure and the polymer was precipitated in pentane to obtain **P27** (1.3 g, 85% yield) ($M_{n,\,CHCl3}$= 2.8 kDa, Đ= 1.77).

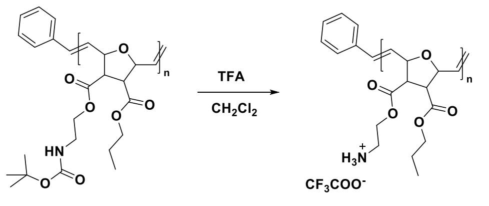

P27 (1 equiv., 2.5 mmol, 1.2 g) was dissolved in 8 mL dry DCM and cooled to 0^0 C. To this pre-cooled mixture, trifluoroacetic acid (51 equiv., 0.13 mmol, 10 mL) was added dropwise and the solution was stirred at room temperature overnight. The resulting solution was concentrated, and the polymer was precipitated in cold diethyl ether to obtain **P28** (1.05 g, 84 %yield) as a colorless solid. **P28** was soluble in both methanol and water.

Effect of additives on catalytic ROMP:

(a)Pyridine as an additive-

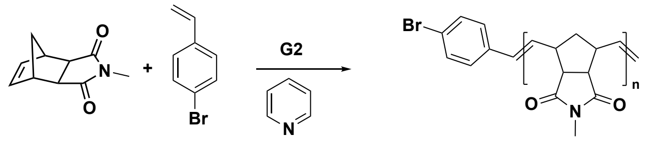

In an NMR tube, **M1** (400 equiv., 471.2 µmol, 84 mg), **CTA2** (20 equiv., 23.56 µmol, 4.3 mg) and pyridine (30 equiv., 35.34 µmol, 2.8 mg) were dissolved 1.0 mL $CDCl_3$ and ^{1}H NMR was recorded over time. Then, **G2** (1 equiv., 1.17 µmol, 1 mg) dissolved in 0.2 mL $CDCl_3$ was added to the NMR tube and the polymerization was followed by ^{1}H NMR spectroscopy over time. Monomer consumption was >99% observed while the **CTA** consumption was 87%.

Due to the better chelation ability of pyridine as a ligand to the Grubbs complexes as compared to 3-bromopyridine, the overall polymerization became sluggish. This only slows down the polymerization kinetics, meaning consumption of both monomer and CTA happened slowly, maintaining the same kinetic chain transfer mechanism. This also, as expected, did not affect the control over molecular weight of the polymer.

The $CDCl_3$ solution was, then, concentrated under reduced pressure until a volume of 0.2 mL was reached and further precipitated from cold methanol to obtain polymer **P31** which was further dried under high vacuum.

$M_{n,theoretical}$ (monomer/CTA) = 3.7 kDa

$M_{n,\ observed}$ (SEC,$CHCl_3$)= 5.0 kDa, $M_{n,\ observed}$ (^{1}H NMR)= 4.5 kDa Đ= 2.0

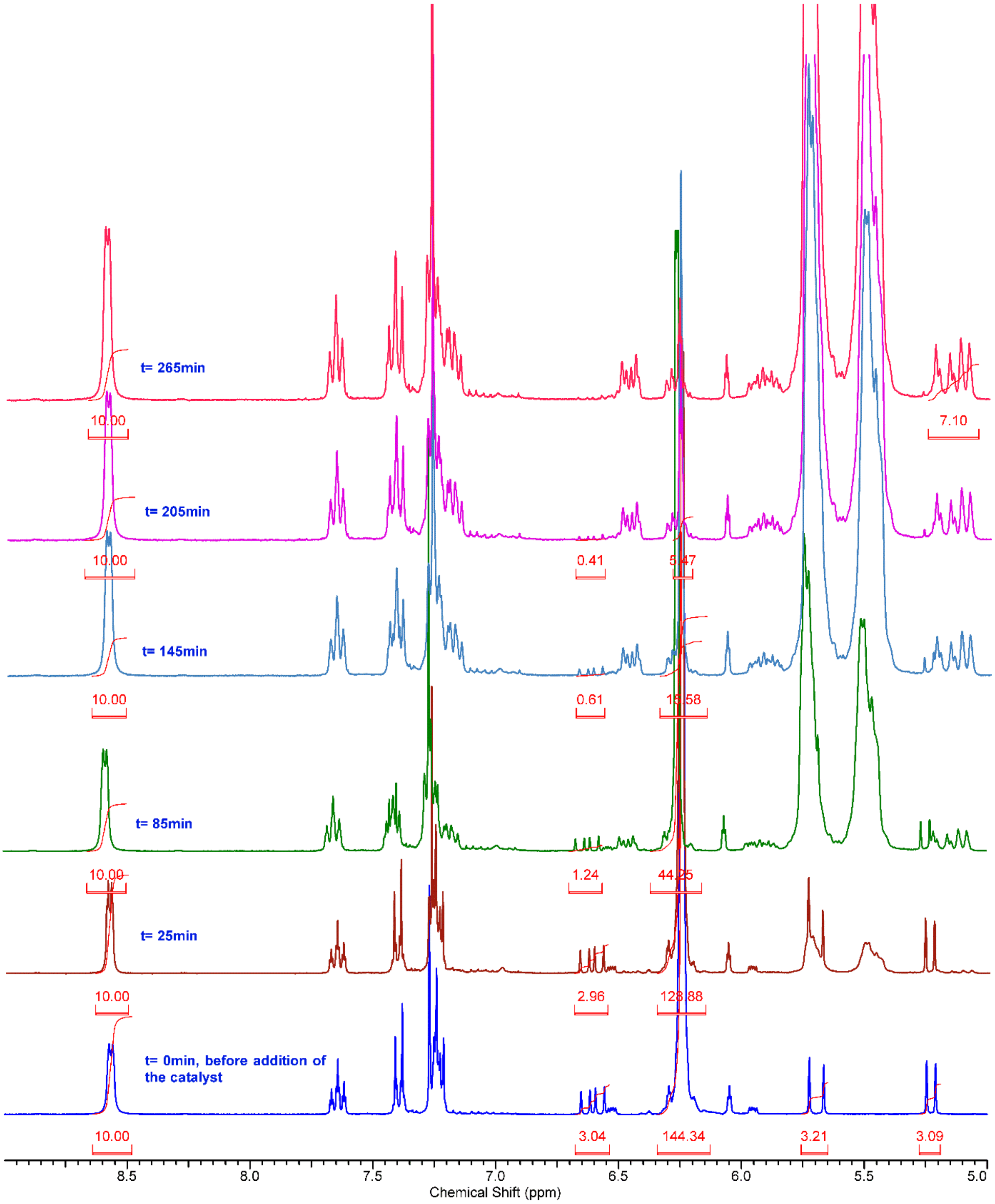

Fig. S17: ^{1}H NMR polymerization reaction of **M1** (400 equiv.) with **CTA2** (20 equiv.) using **G2** (1 equiv.). Stacked ^{1}H NMR spectra of olefinic region over time showing consumption of both monomer (6.25 ppm) and CTA (6.53 ppm). Peak at 8.51 ppm corresponds to the pyridine which considered as an internal standard for integration.

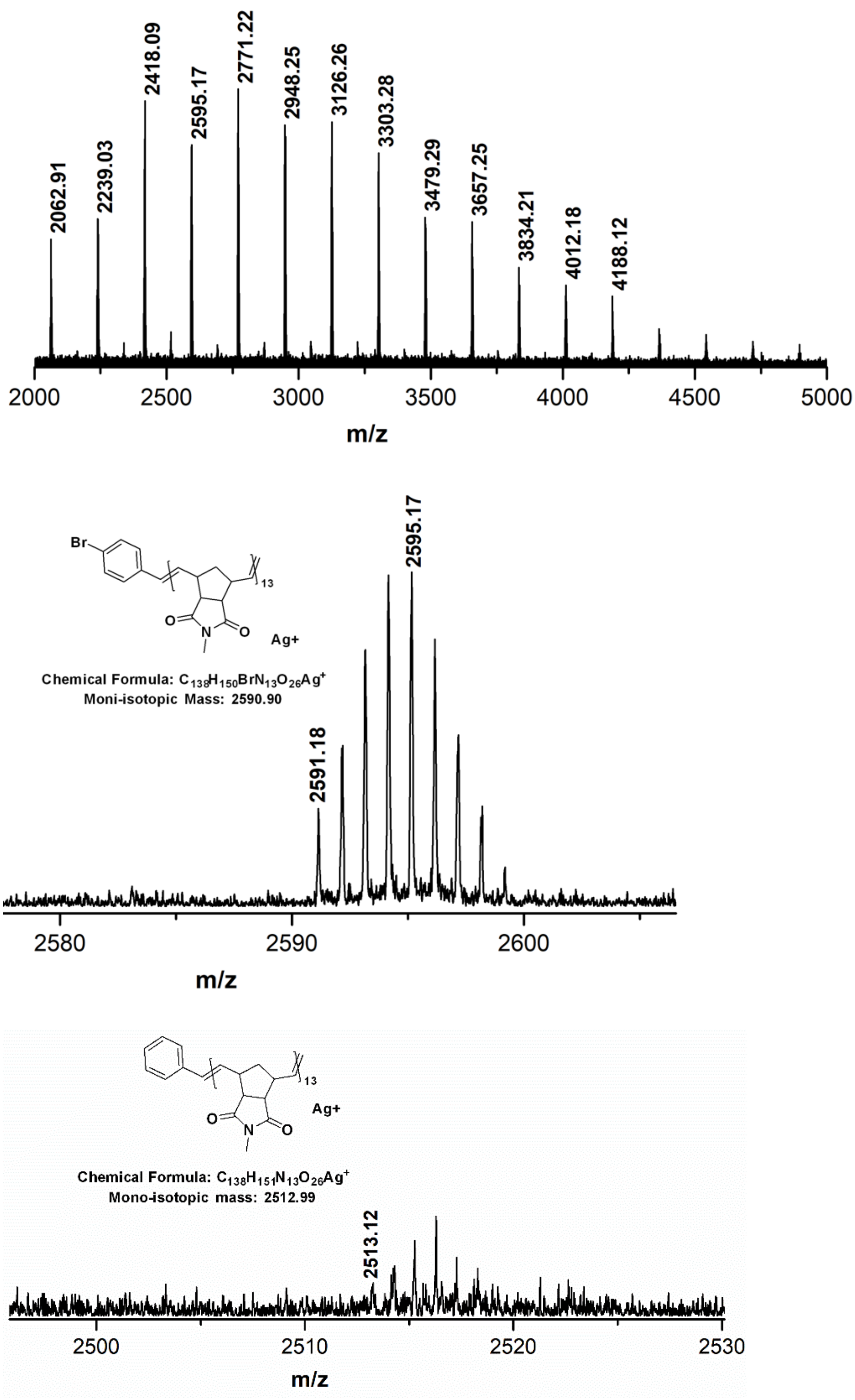

Fig. S18: MALDI-ToF (DCTB, AgTFA) mass spectrum of **P31**. The smaller distribution did not match with any possible non-regioselective metathesis events that may generate homotelechelic chain ends rather it's came from Grubbs' catalyst (**G2**).

(b)Triphenylphosphine as an additive-

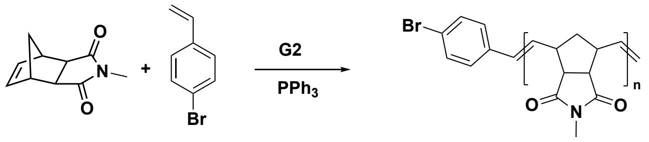

In an NMR tube, **M1** (400 equiv., 471.2 µmol, 84 mg), **CTA2** (20 equiv., 23.56 µmol, 4.3 mg) and triphenylphosphine (30 equiv., 35.34 µmol, 9.3 mg) were dissolved 1.0 mL $CDCl_3$ and ^1H NMR was recorded over time. Then, **G2** (1 equiv., 1.17 µmol, 1 mg) dissolved in 0.2 mL $CDCl_3$ was added to the NMR tube and the polymerization was followed by ^1H NMR spectroscopy over time. Monomer consumption was >99% observed while the **CTA** consumption was >99%.

As explained before, PPh_3 also slower down the polymerization kinetics. The $CDCl_3$ solution was, then, concentrated under reduced pressure until a volume of 0.2 mL was reached and further precipitated from cold methanol to obtain polymer **P32** which was further dried under high vacuum.

$M_{n,theoretical}$ (monomer/CTA) = 3.7 kDa

$M_{n, observed}$ (SEC,$CHCl_3$)= 5.2 kDa, $M_{n, observed}$ (^1H NMR)= 4.6 kDa Đ= 1.9

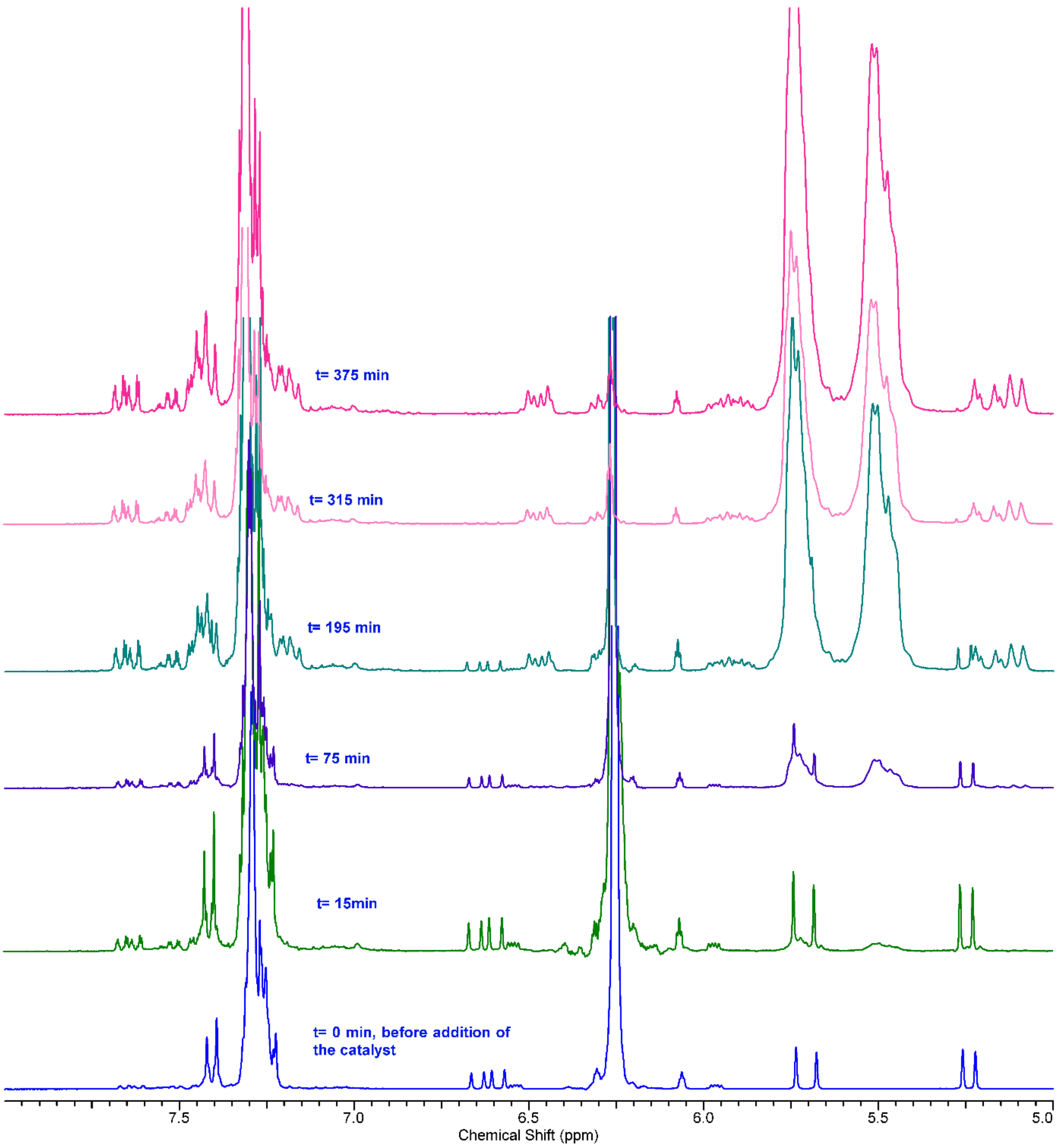

Fig. S19: ^{1}H NMR polymerization reaction of **M1** (400 equiv.) with **CTA2** (20 equiv.) using **G2** (1 equiv.). Stacked ^{1}H NMR spectra of olefinic region over time showing consumption of both monomer (6.25 ppm) and CTA (6.53 ppm). Peaks at 7.25 ppm is from PPh_3.

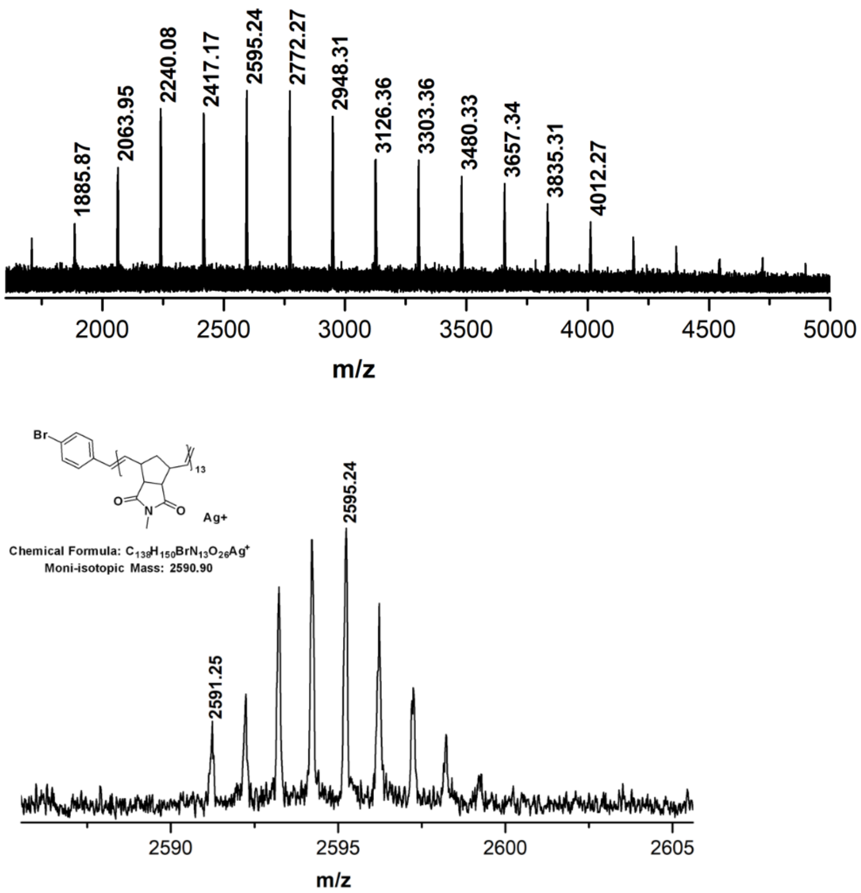

Fig. S20: MALDI-ToF (DCTB, AgTFA) mass spectrum of **P32**.

Slow propagating monomer for catalytic ROMP:

Endo-N-methylnorbornene imide (endo-MNI) was used as a slow propagating monomer for this study.

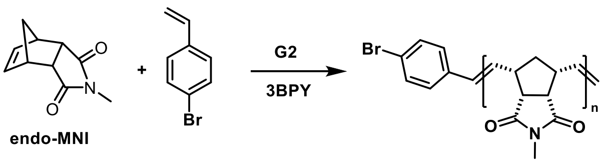

In an NMR tube, **endo-MNI** (400 equiv., 471.2 µmol, 84 mg), and **CTA2** (20 equiv., 23.56 µmol, 4.3 mg) and 3BPY (30 equiv., 35.34 µmol, 5.6 mg) were dissolved 1.0 mL $CDCl_3$ and ^1H NMR was recorded over time. Then, **G2** (1 equiv., 1.17 µmol, 1 mg) dissolved in 0.2 mL $CDCl_3$ was added to the NMR tube and the polymerization was followed by ^1H NMR spectroscopy over time.

Monomer conversion was 35% whereas CTA conversion was 71.2%. Clearly, catalyst decomposition competes over monomer propagation/chain transfer under the reaction condition here (adding 3BPY).

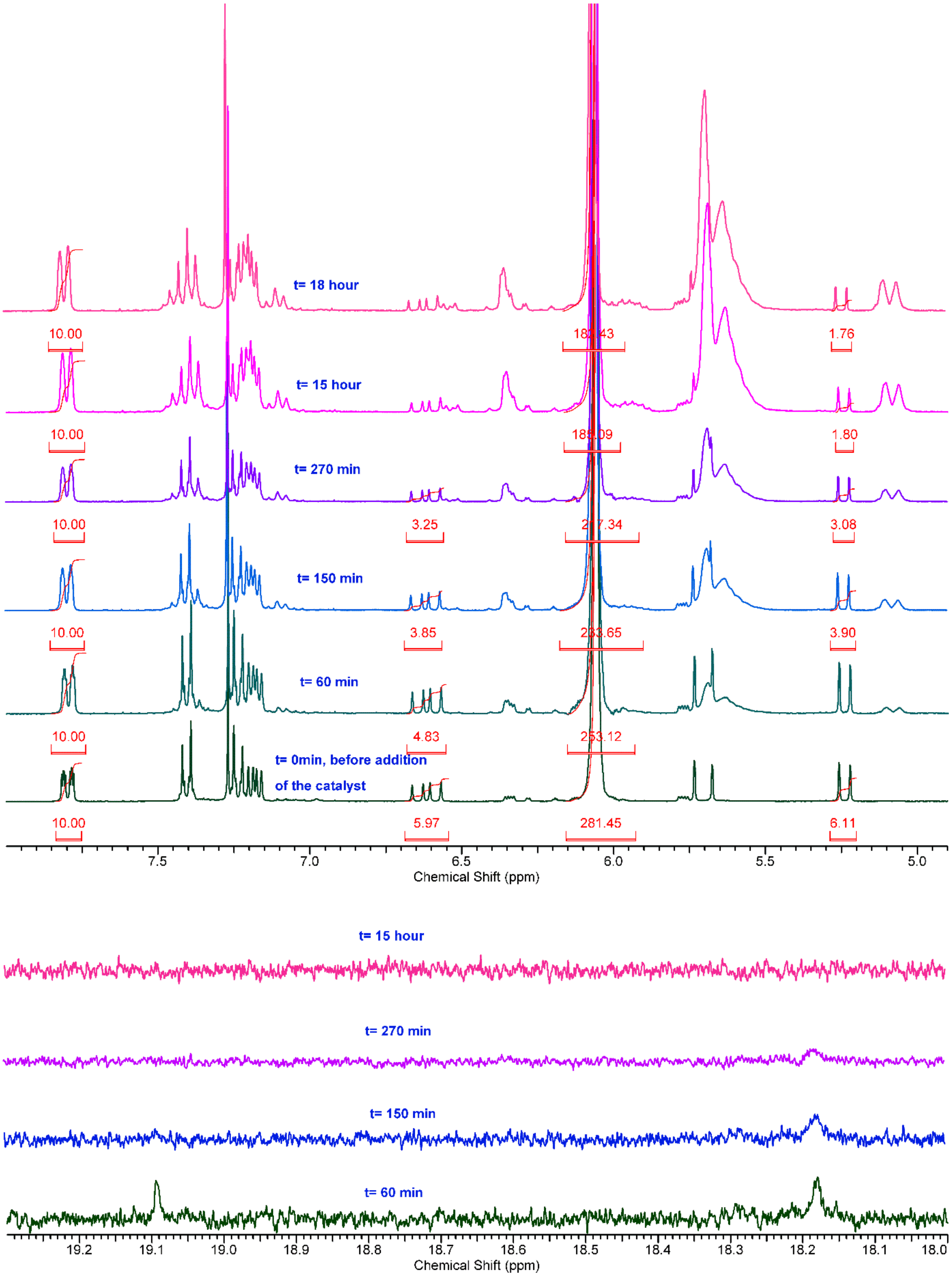

Fig. S21: ^{1}H NMR polymerization reaction of endo-**MNI** (400 equiv.) with **CTA2** (20 equiv.) using **G2** (1 equiv.). Top spectra: stacked ^{1}H NMR spectra of olefinic region over time showing consumption of both monomer (6.1 ppm) and CTA (6.53 ppm). Peaks at 7.8 ppm is from 3BPY. Bottom spectra: stacked ^{1}H NMR spectra of carbene region over time showing decomposition of catalyst over time. (**G2**-benzilidine signal at 19.1 ppm, propagating endo-MNI signal at 18.18 ppm).

Next, we perform the same polymerization in the absence of any additive (3BPY)-

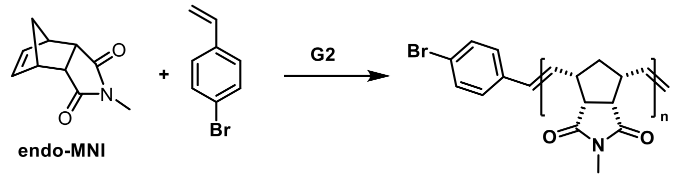

In an NMR tube, **endo-MNI** (400 equiv., 471.2 µmol, 84 mg), and **CTA2** (20 equiv., 23.56 µmol, 4.3 mg) were dissolved 1.0 mL $CDCl_3$ and ^{1}H NMR was recorded over time. Then, **G2** (1 equiv., 1.17 µmol, 1 mg) dissolved in 0.2 mL $CDCl_3$ was added to the NMR tube and the polymerization was followed by ^{1}H NMR spectroscopy over time.

In this case, 96% consumption was monomer was observed, while the CTA consumed fully before whole monomer was polymerized. This led to control over number average molecular weight of the final polymer. But, as no additive was used non-regioselective chain transfer was observed which produced mixture of chain ends, as proved via MALDI-ToF mass spectrometry (see below).

Another important observation for slow propagating monomer was since almost all the CTA was consumed (in this case 90%) when monomer conversion was only 50%, during consumption of the rest of the 50% of the monomer, propagating Ru alkylidene species could also do the chain transfer with the polymeric chain ends capped with a methylene group. If this chain transfer is non-regioselective, that could also lead to homotelechelic chain ends.

The $CDCl_3$ solution was concentrated under reduced pressure until a volume of 0.2 mL was reached and further precipitated from cold methanol to obtain polymer **P33** which was further dried under high vacuum.

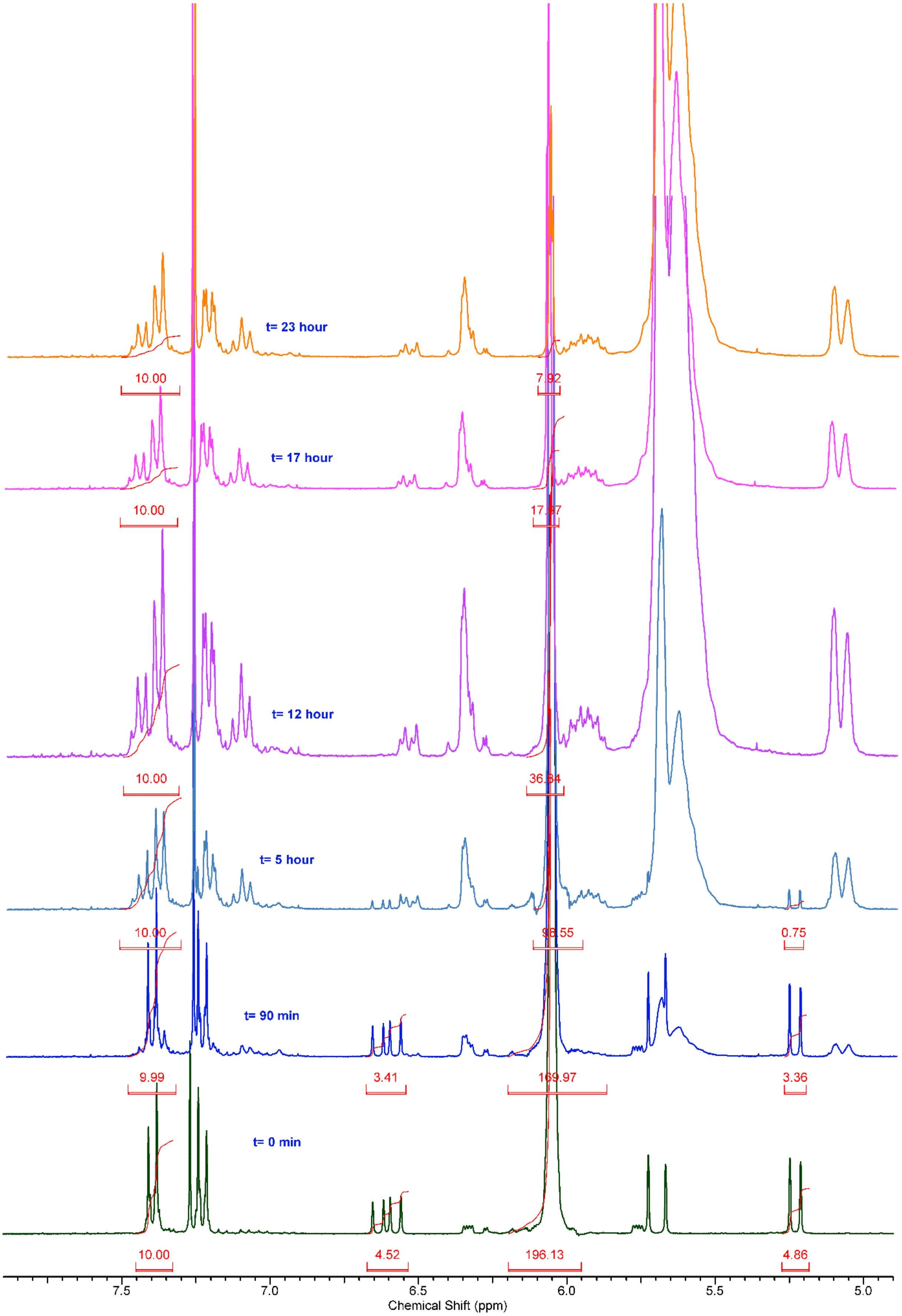

Fig. S22: ^{1}H NMR polymerization reaction of endo-**MNI** (400 equiv.) with **CTA2** (20 equiv.) using **G2** (1

equiv.). Stacked ^{1}H NMR spectra of olefinic region over time showing consumption of monomer (6.1 ppm) and CTA (6.53 ppm).

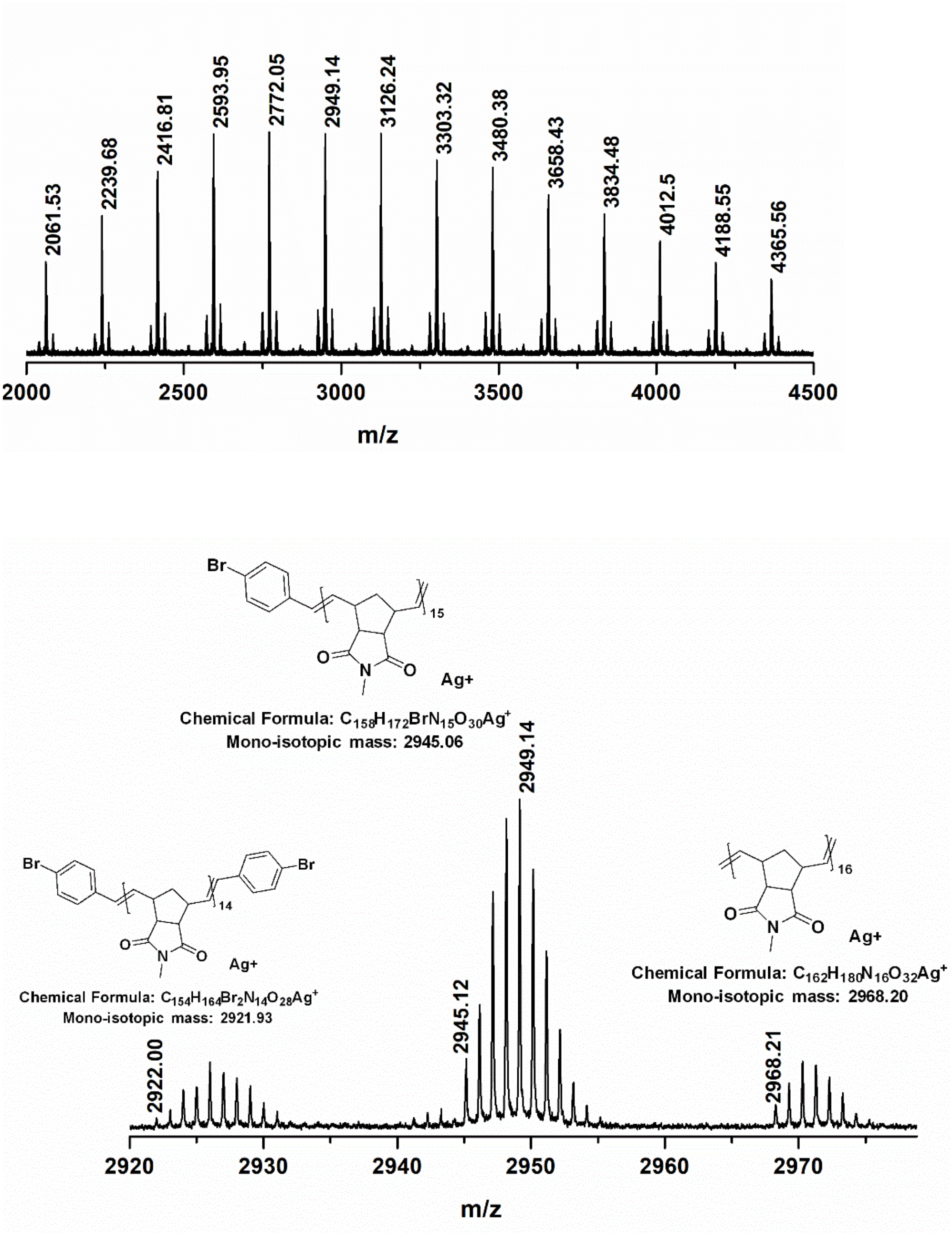

Fig. S23: MALDI-ToF (DCTB, AgTFA) mass spectrum of **P33**.

Catalytic polymerization at higher temperature:

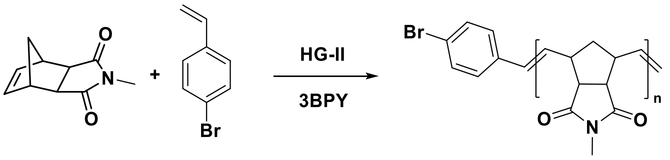

In a round-bottom flask, **M1** (1000 equiv., 2.39 mmol, 423 mg), **CTA2** (50 equiv., 119.3 µmol, 22 mg) and **3BPY** (30 equiv., 71.6 µmol, 11.3 mg) were dissolved 6.0 mL 1, 2-dichloroethane. The flask was immersed to a preheated oil-bath of temperature 45^{0}C. Then, **HG-II** (1 equiv., 2.39 µmol, 1.5 mg), dissolved in 0.1 mL 1, 2-dichloroethane was added to the flask and the mixture was stirred at 45^{0}C for 30 mins, upon which ^{1}H NMR spectroscopic measurement showed >97% of monomer consumption. The oil-bath was removed, and few drops of ethyl vinyl ether was added. The solvent was removed under reduced pressure up to a vol and the crude was precipitated from cold methanol to obtain a colorless polymer **P34**.

Polymerization temperature was restricted to 45^{0}C, as higher temperature produces side reactions such as intermolecular chain transfer (backbiting or secondary metathesis) which would give mixture of chain ends in the final polymer.

$M_{n,theoretical}$ (monomer/CTA) = 3.7 kDa

$M_{n,\ observed}$ (SEC,$CHCl_3$)= 5.0 kDa, $M_{n,\ observed}$ (^{1}H NMR)= 4.5 kDa Đ= 2.1

Kinetics Experiment:

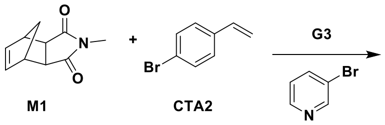

In an NMR tube, **M1** (1000 equiv., 1.13 mmol, 200 mg), **CTA2** (50 equiv., 56.53 µmol, 10.3 mg) and 3-bromopyridine (**3BPY**) (60 equiv., 68 µmol, 11 mg) were dissolved 0.9 mL $CDCl_3$ and ^{1}H NMR was recorded. Then, **G3** (1 equiv., 1.13 µmol, 1 mg) dissolved in 0.2 mL $CDCl_3$ was added to the NMR tube and the polymerization was followed by ^{1}H NMR spectroscopy over time. After 80 mins, 98.8% consumption of monomer was observed while the **CTA** consumption was 94.5%.

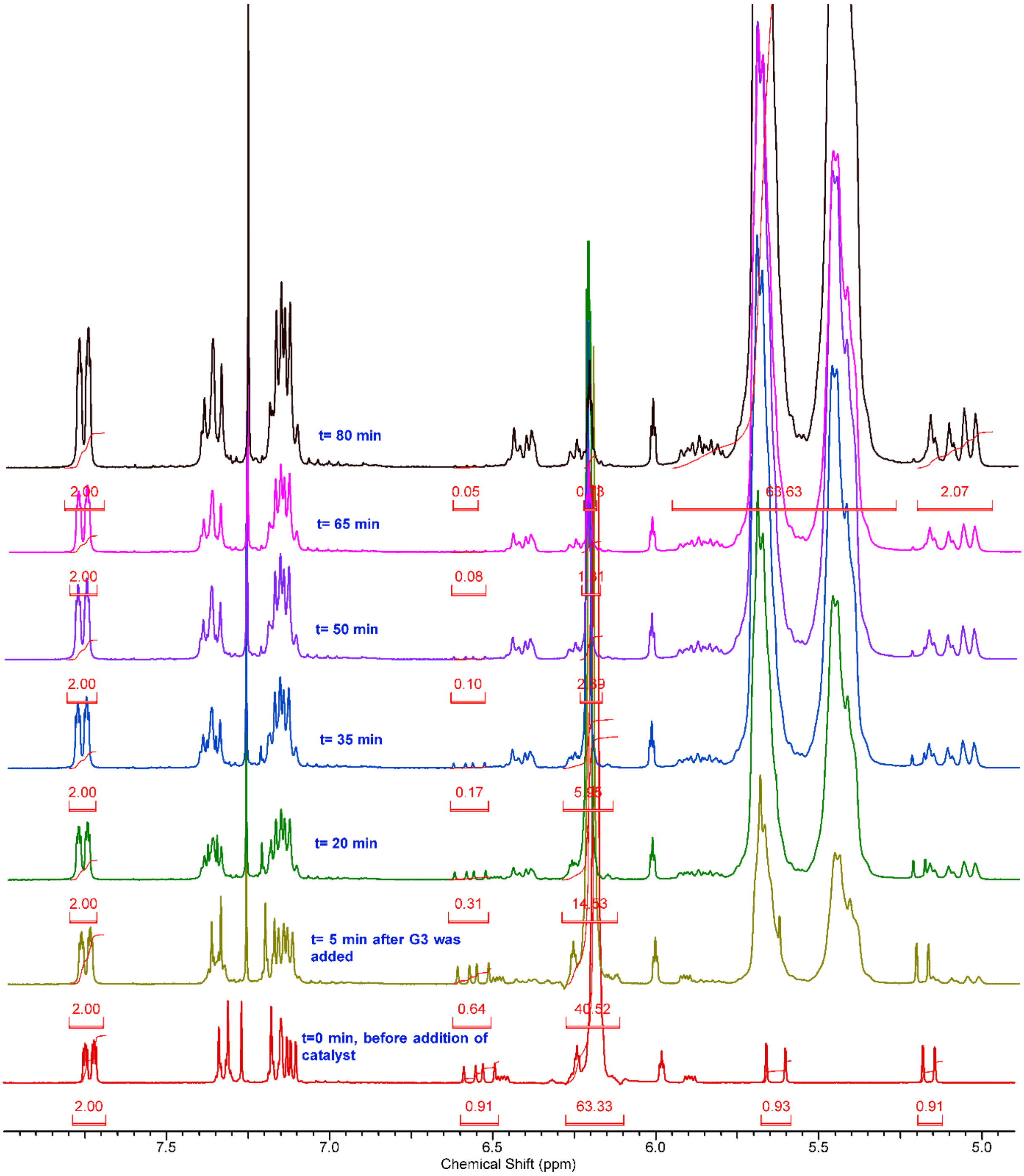

Fig. S24: ^{1}H NMR ($CDCl_3$, 300 MHz) polymerization reaction of **M1** (1000 equiv.) with **CTA2** (50 equiv.) using **G3** (1 equiv.). Stacked ^{1}H NMR spectra of olefinic region over time showing consumption of both monomer and CTA. Peak at 5.95 ppm is the endo-isomer of **M1**.

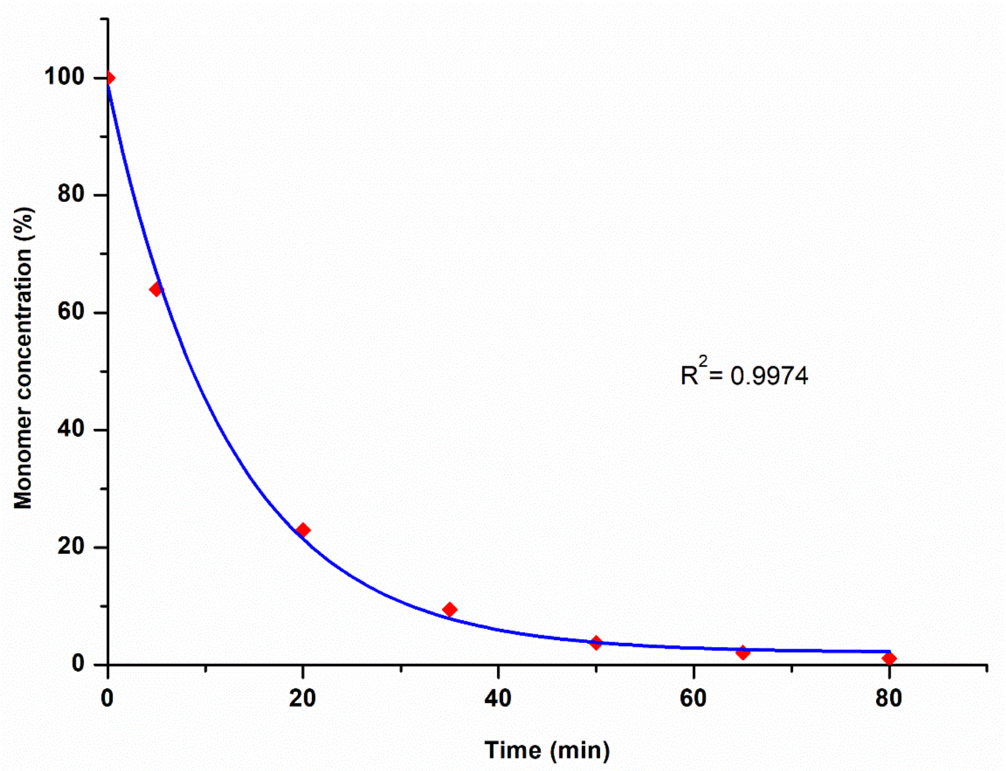

Fig. S25: A plot of monomer (**M1**) concentration vs. time determined by ^{1}H NMR spectroscopy ($CDCl_3$, 300 MHz) showing an exponential decay in concentration of monomer during the polymerization.

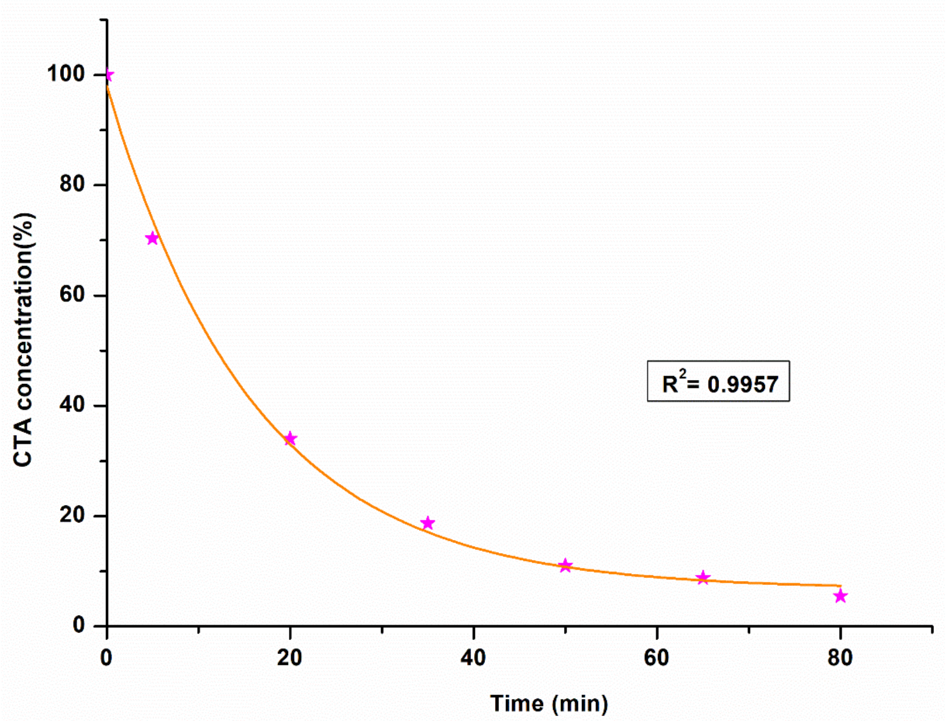

Fig. S26: A plot of **CTA** (**CTA2**) concentration vs. time determined by ^{1}H NMR spectroscopy ($CDCl_3$, 300 MHz) showing an exponential decay in concentration of CTA during the polymerization.

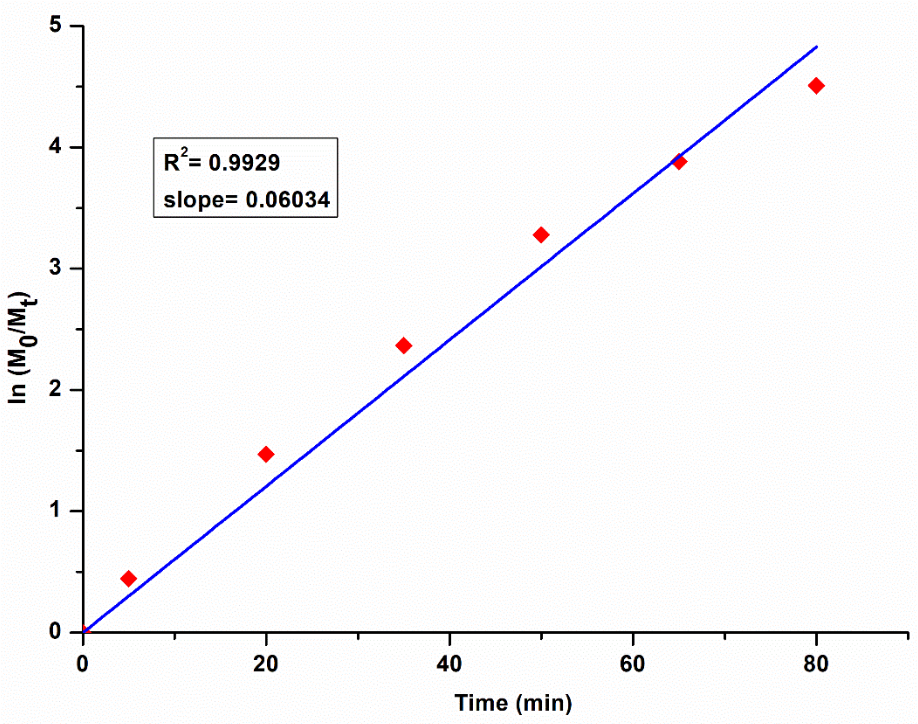

Fig. S27: Plot of ln (M_0/M_t) against time. M_0= initial concentration of monomer (**M1**) before the addition of catalyst and M_t= the concentration of **M1** at a given time.

ln (M_0/M_t) against time provided a linear correlation and the slope of the linear regression corresponded to the rate constant for monomer consumption (k_{M1}) under the given reaction conditions. Thus, k_{M1}= 6.0*10^{-2} min^{-1}

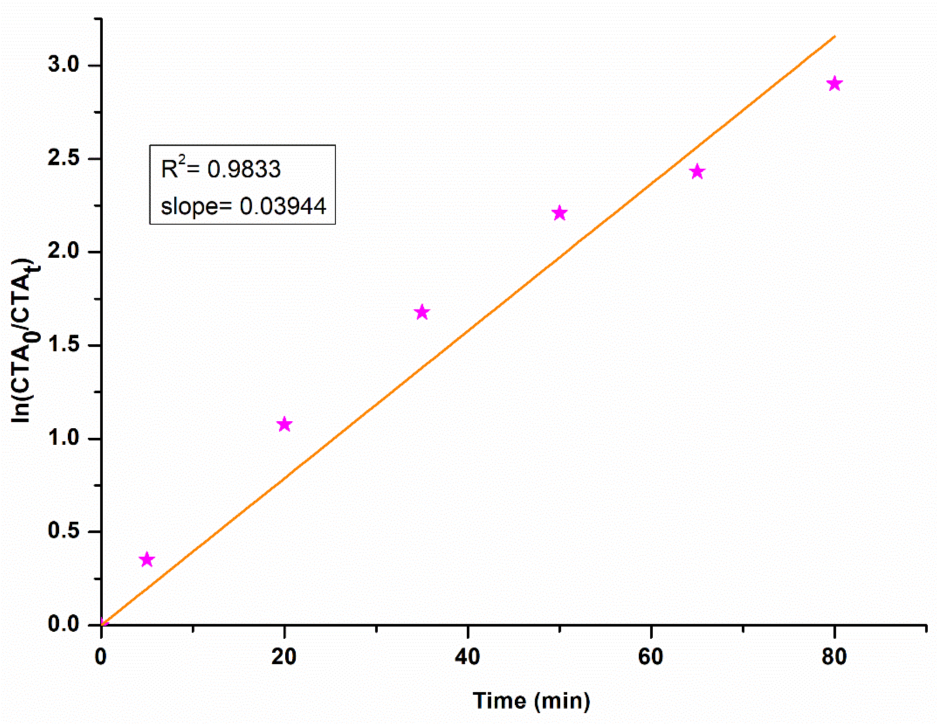

Fig. S28: Plot of ln (CTA_0/CTA_t) against time. CTA_0= initial concentration of **CTA2** before the addition of catalyst and CTA_t= the concentration of **CTA2** at a given time.

From the linear plot, slope i.e. **CTA2** consumption rate constant (k_{CTA2}) is 3.9*10^{-2} min^{-1}.

Thus, rate constant for the consumption of monomer was 1.53 times higher than the rate constant for the consumption of CTA, explaining why the observed number average molecular weight of the synthesized polymers a little higher than theoretically predicted.

Determination of rate constants by Mayo equation:

For a chain transfer, chain growth polymerization, the degree of polymerization (DP) should follow the Mayo equation[6,7] as given below-

$$\frac{1}{DP} = \frac{1}{DP_0} + \frac{k_{CTA}[CTA]}{k_M[monomer]}$$

DP_0= degree of polymerization in the absence of any chain transfer/chain termination reaction.

This equation holds under the steady-state conditions. In fact, when the CTA concentration is high (which approximates our reaction conditions), $1/DP_0$ term can be neglected. Nonetheless, a plot of 1/DP vs [CTA]/[monomer] provided a linear graph as shown below-

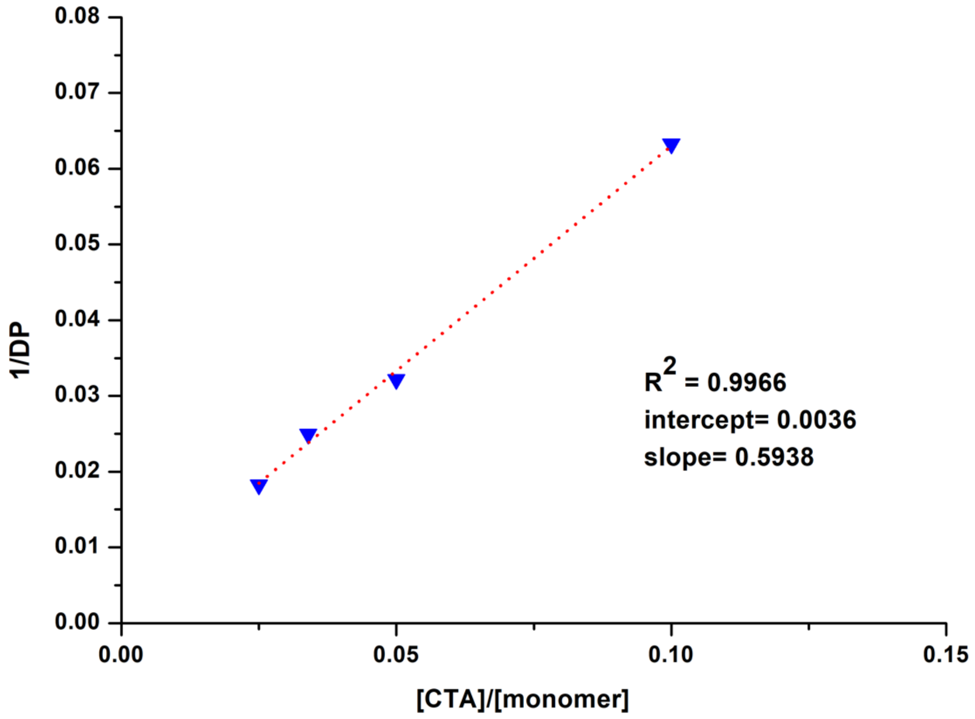

Fig. S29: Linear relationship between 1/DP vs [CTA]/[monomer].

The plotted data were taken from the polymers **P4**, **P5**, **P6**, **P7**.

Slope= $\frac{k_{CTA}}{k_M}$ = 0.5938 or k_M= 1.68 k_{CTA}. Thus, propagation under this condition is 1.68 times faster than the transfer constant of the CTA. This value is very close with our determined consumption rate constant (k_M/k_{CTA}= 1.53) using the plot of ln (S_0/S_t) against time (S= substrate) (Fig. S27 and S28). Thus, both approach is viable to quantitatively determine the rate constants for our catalytic ROMP method.

Zoomed MALDI-ToF with mono-isotopic mass simulation:

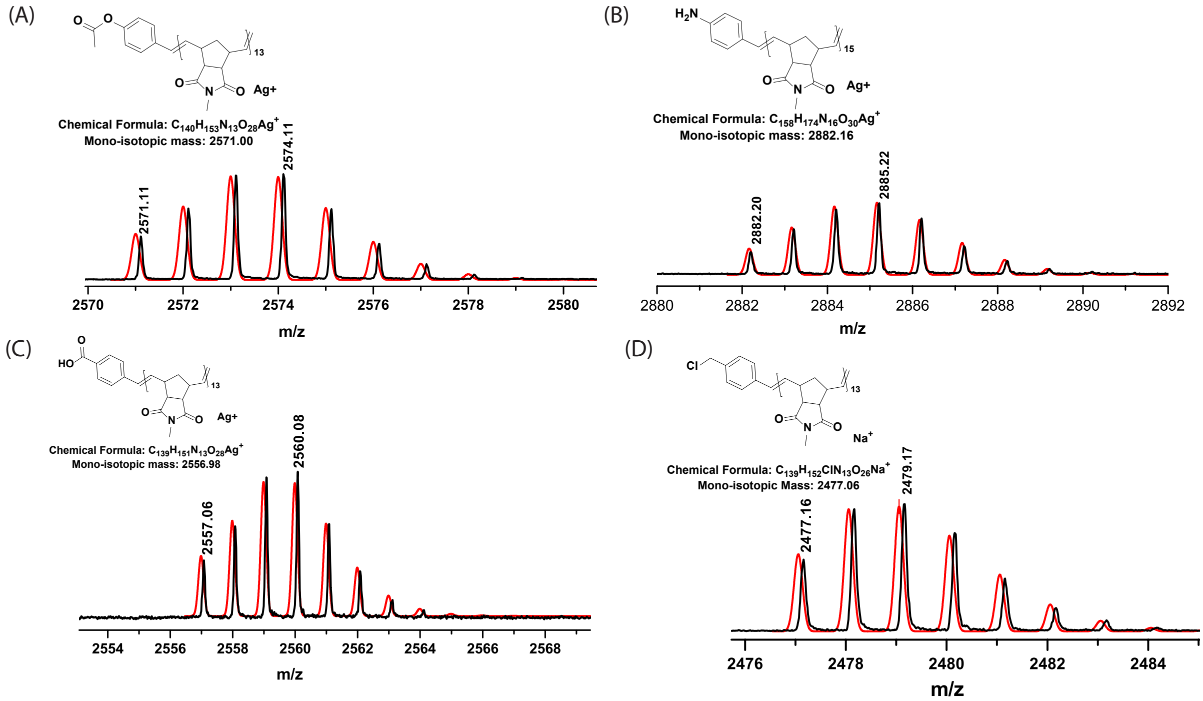

Fig. S30: Selected zoomed region of the isotopically resolved MALDI-ToF (DCTB, AgTFA/NaTFA) spectra for (A) **P10**, (B) **P24**, (C) **P25**, and (D) **P26** showing a comparison in the isotopic distributions between the simulated spectra (red lines) and the experimentally observed spectra (black lines). The maximum mass difference between the two spectra observed was 0.11 (Fig. S30 A) which could be explained on the basis that the MALDI-ToF measurements were performed using polystyrene (M_n= 4 kDa) as a calibrant that has a slightly different mass than that of our polymers.

NMR spectra of chain transfer agents and monomers:

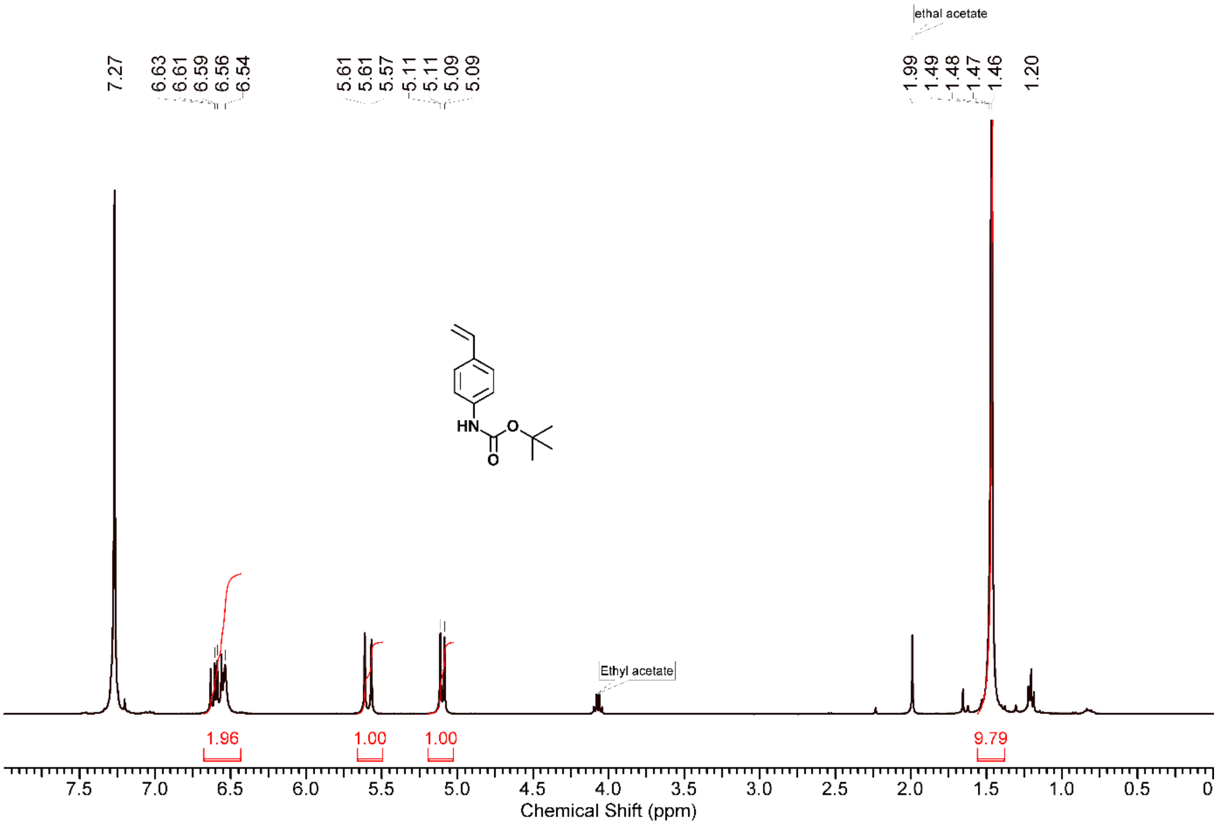

Fig. S31: ^{1}H NMR ($CDCl_3$, 400 MHz) spectrum of **CTA7**.

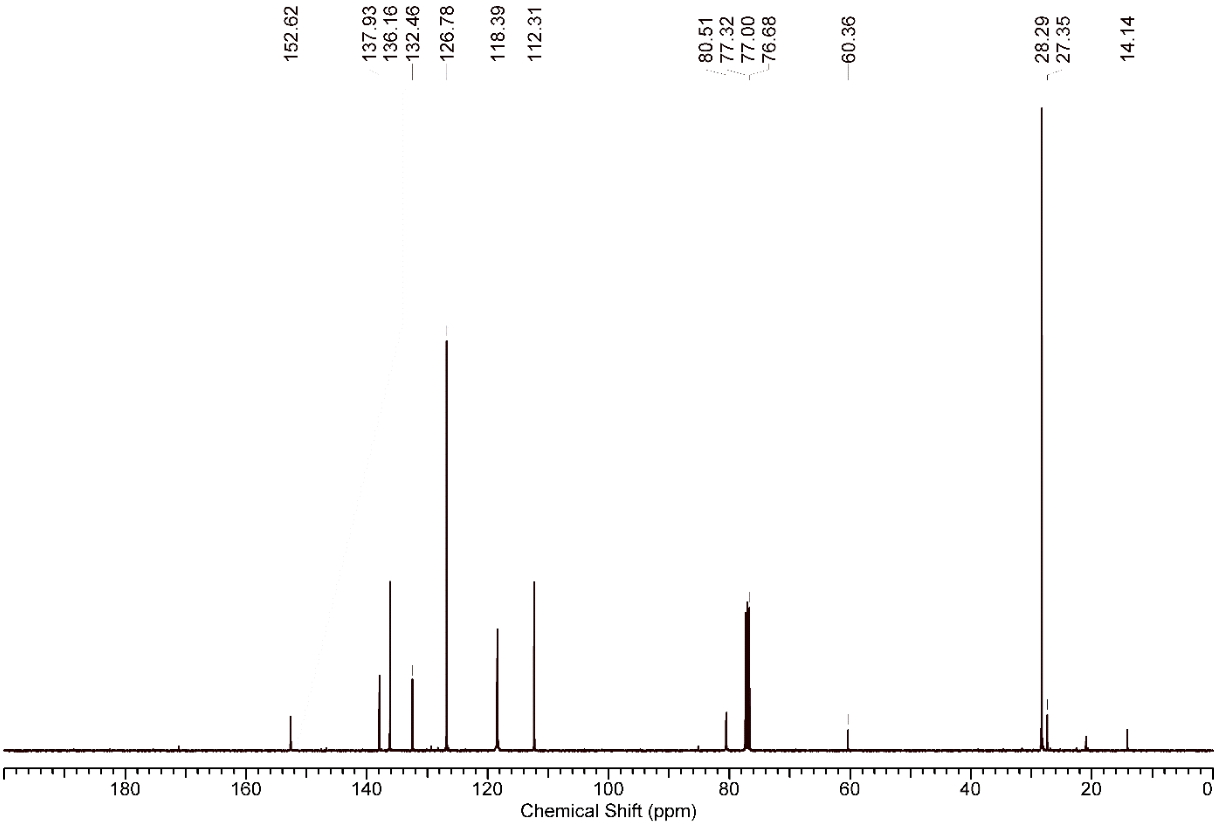

Fig. S32: ^{13}C NMR ($CDCl_3$, 101 MHz) spectrum of **CTA7**.

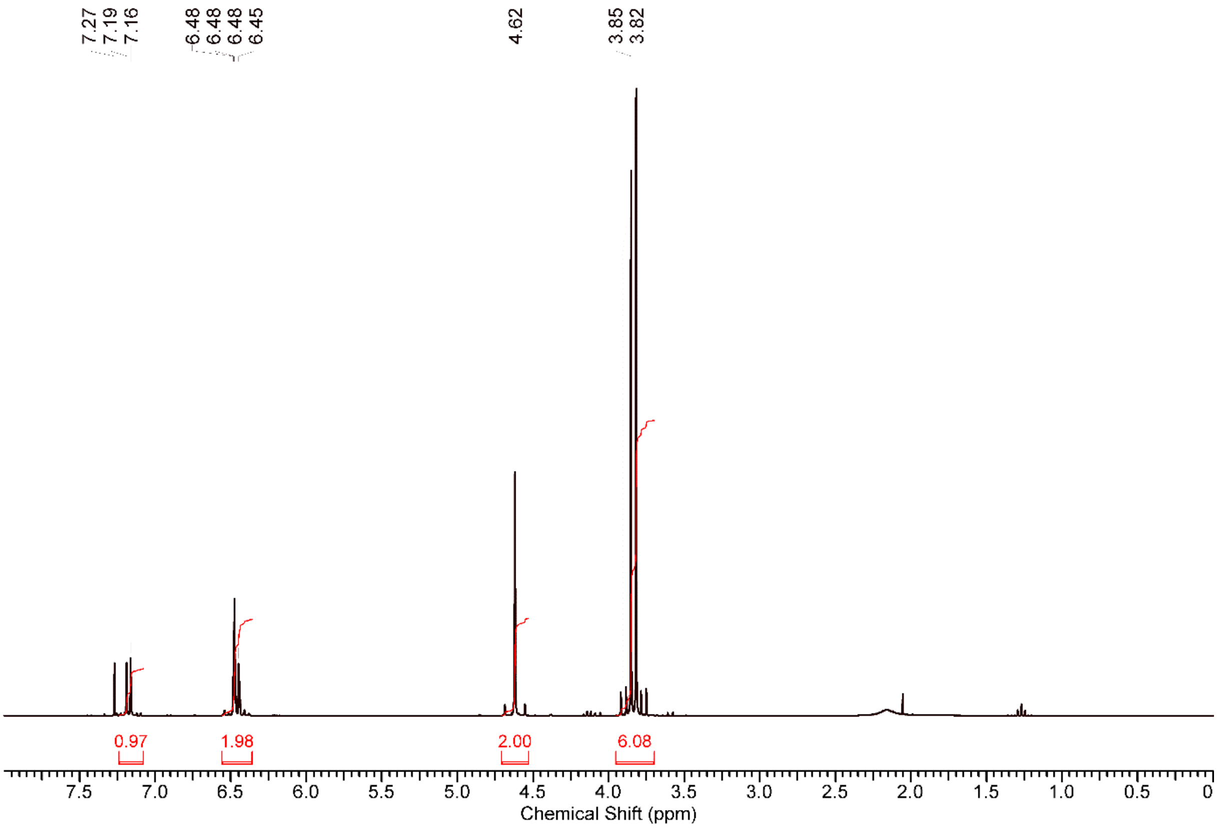

Fig. S33: ^{1}H NMR ($CDCl_3$, 400 MHz) spectrum of **I1**.

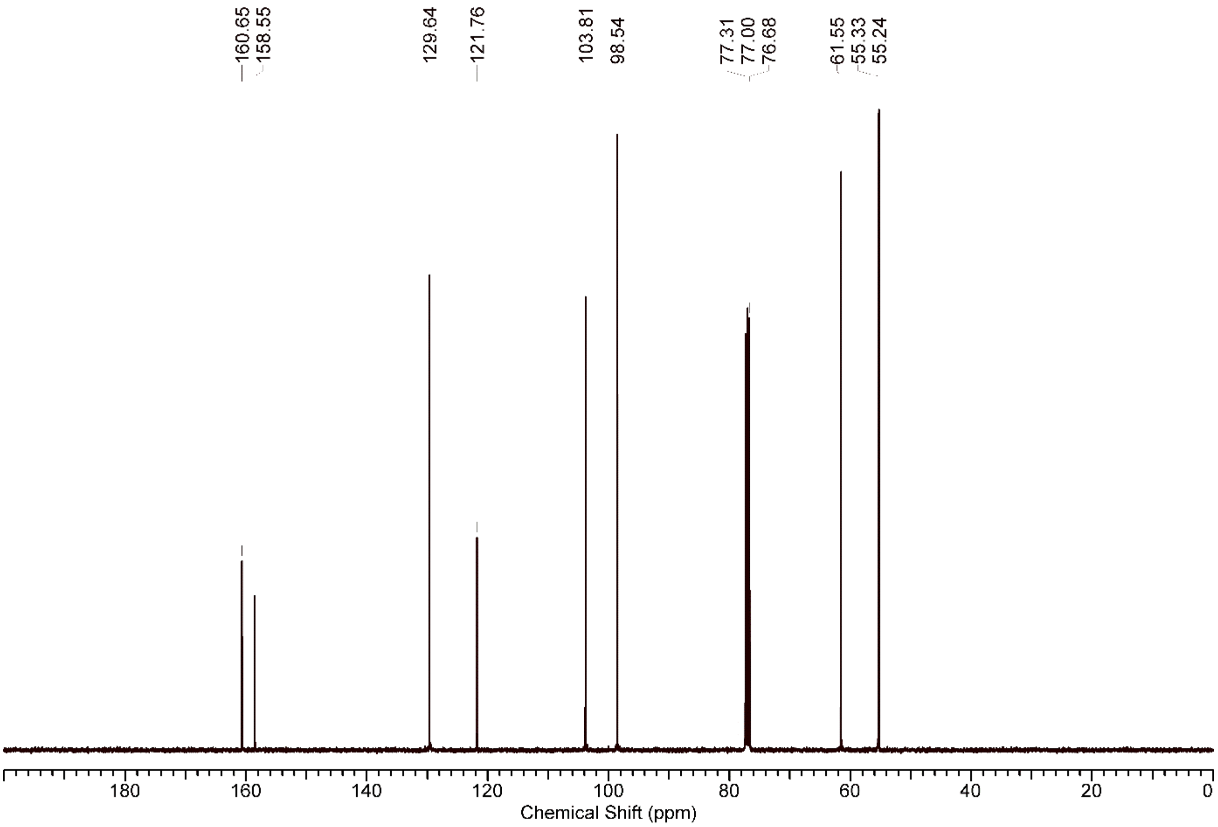

Fig. S34: ^{13}C NMR ($CDCl_3$, 101 MHz) spectrum of **I1**.

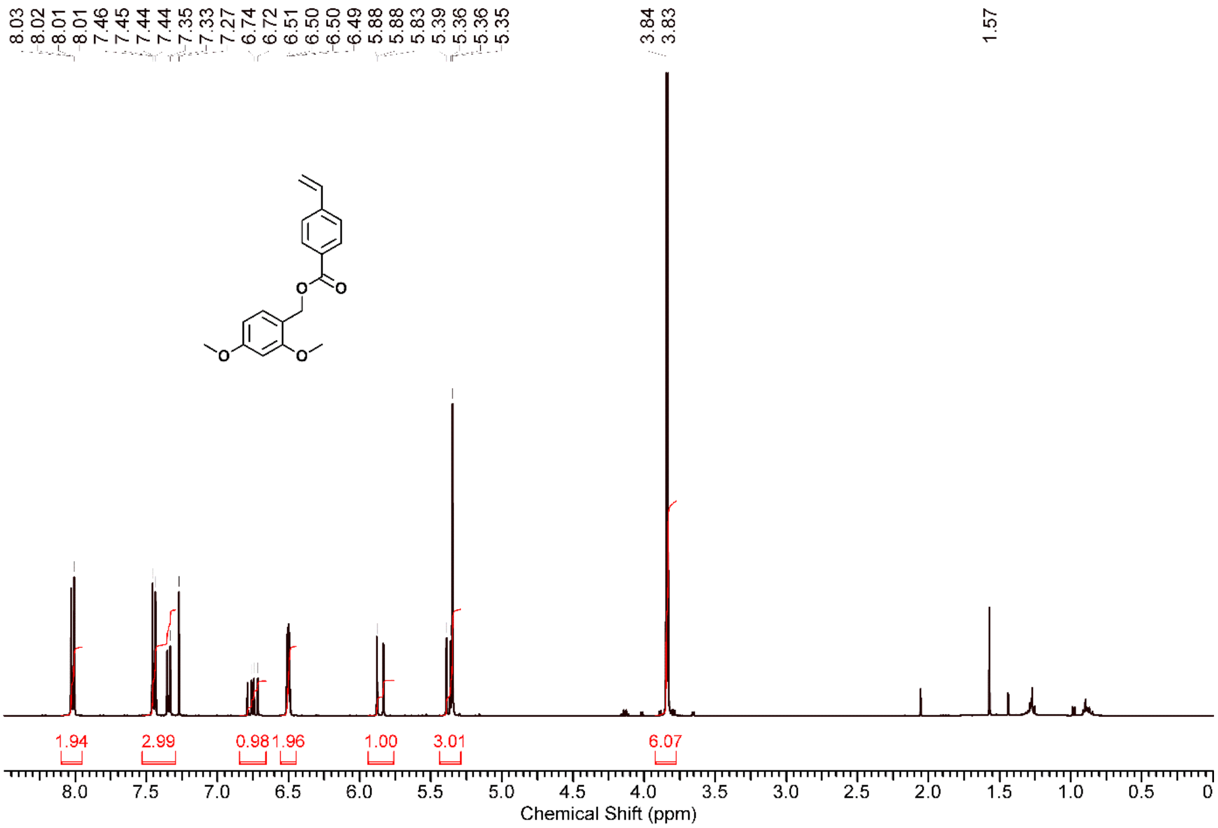

Fig. S35: ^{1}H NMR ($CDCl_3$, 400 MHz) spectrum of **CTA8**.

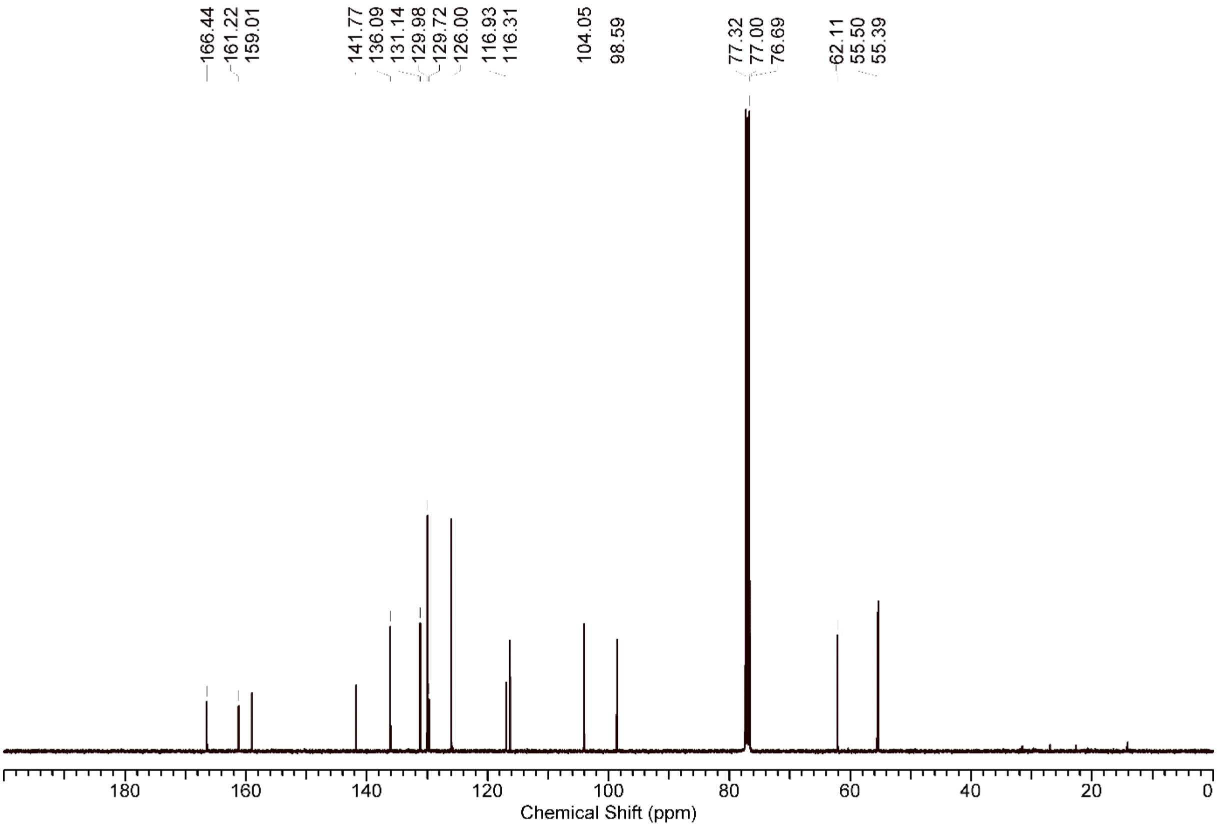

Fig. S36: ^{13}C NMR ($CDCl_3$, 101 MHz) spectrum of **CTA8.**

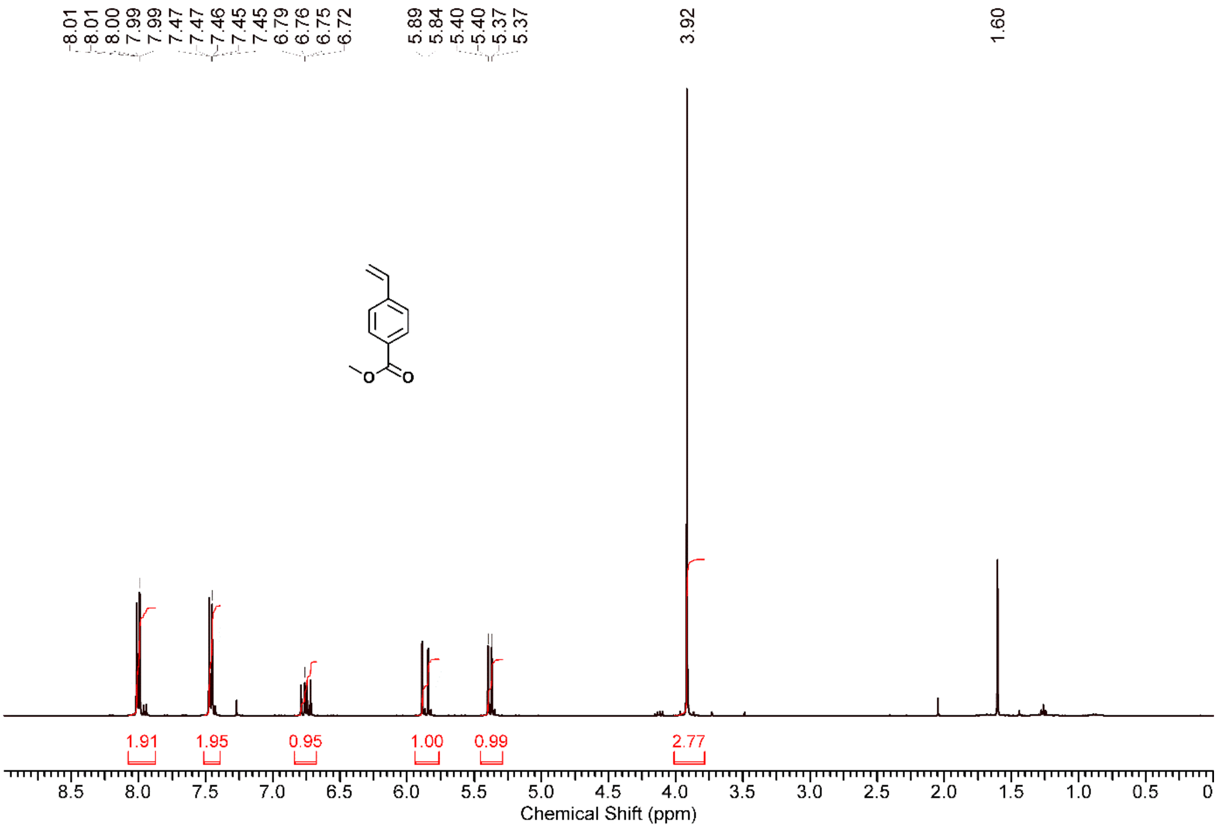

Fig. S37: ^{1}H NMR ($CDCl_3$, 400 MHz) spectrum of **CTA9**.

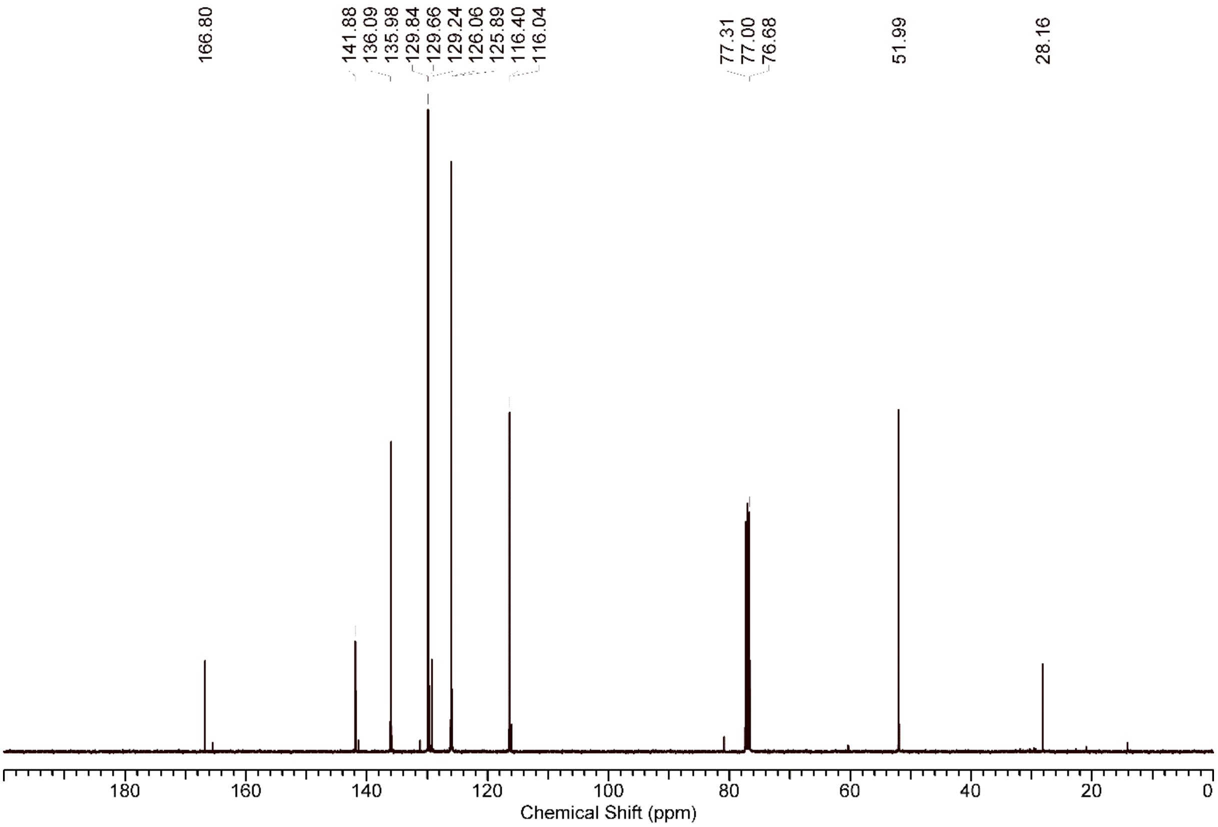

Fig. S38: ^{13}C NMR ($CDCl_3$, 101 MHz) spectrum of **CTA9.**

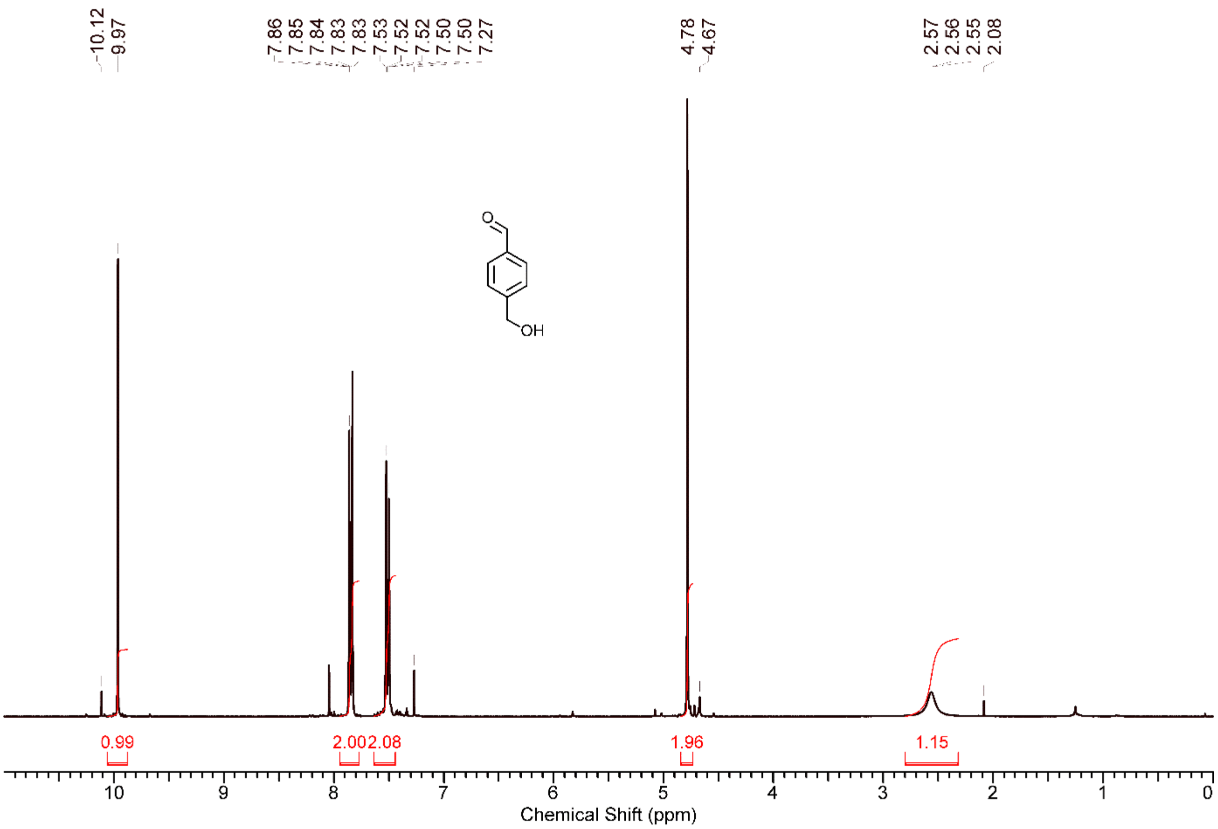

Fig. S39: ^{1}H NMR ($CDCl_3$, 300 MHz) spectrum of **I2**.

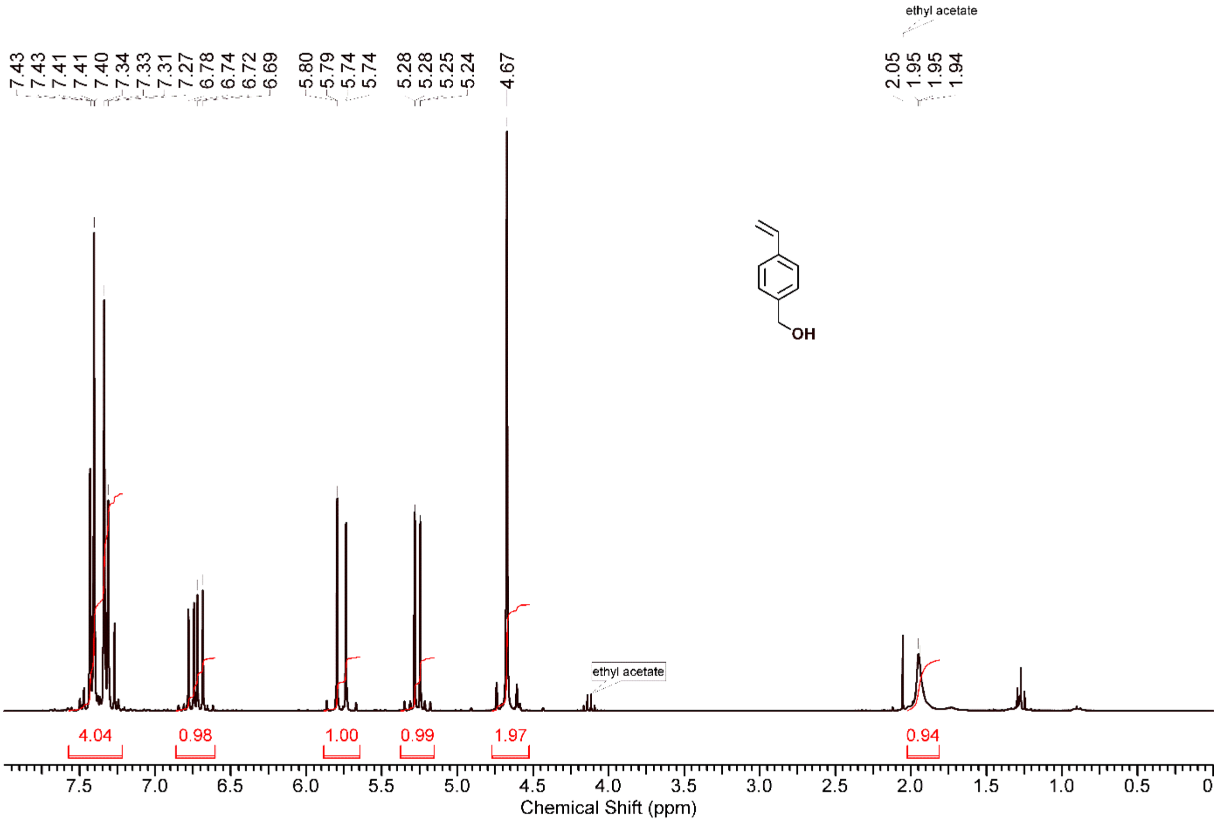

Fig. S40: ^{1}H NMR ($CDCl_3$, 300 MHz) spectrum of **CTA10**.

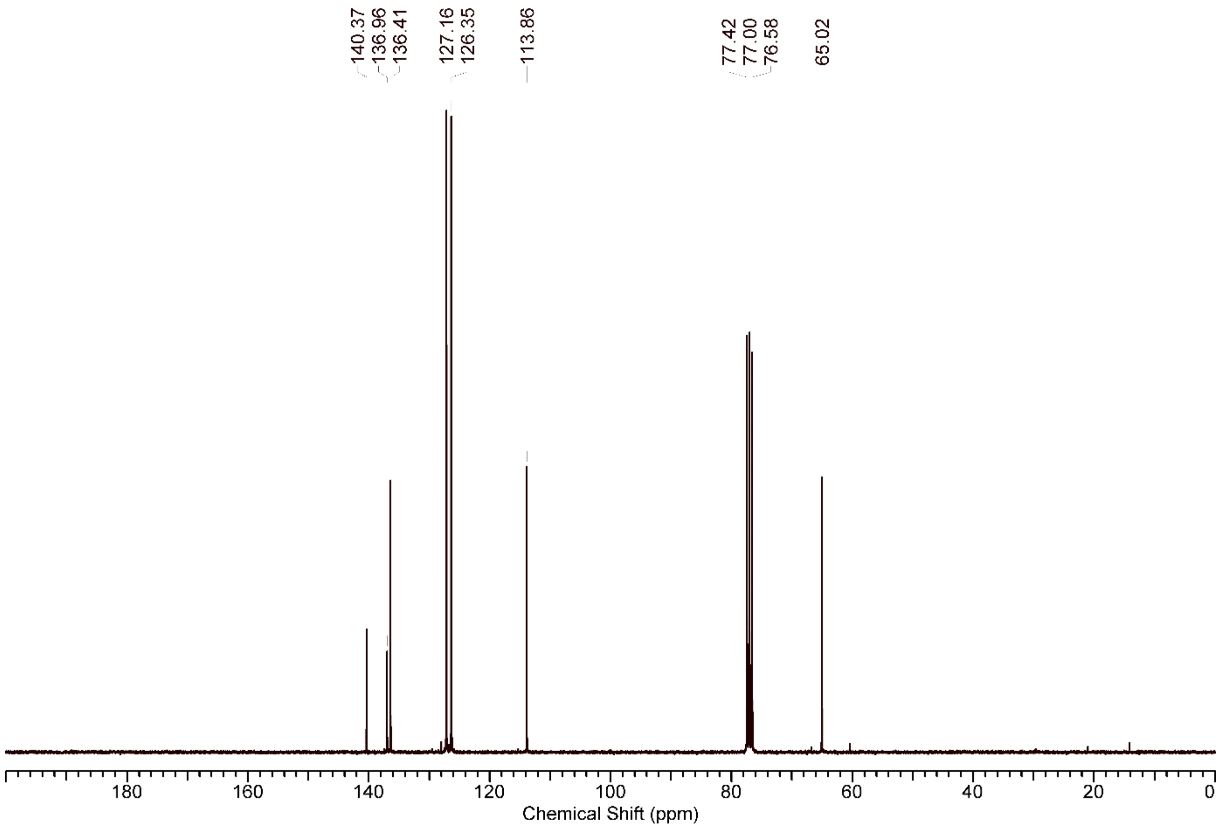

Fig. S41: ^{13}C NMR ($CDCl_3$, 75 MHz) spectrum of **CTA10.**

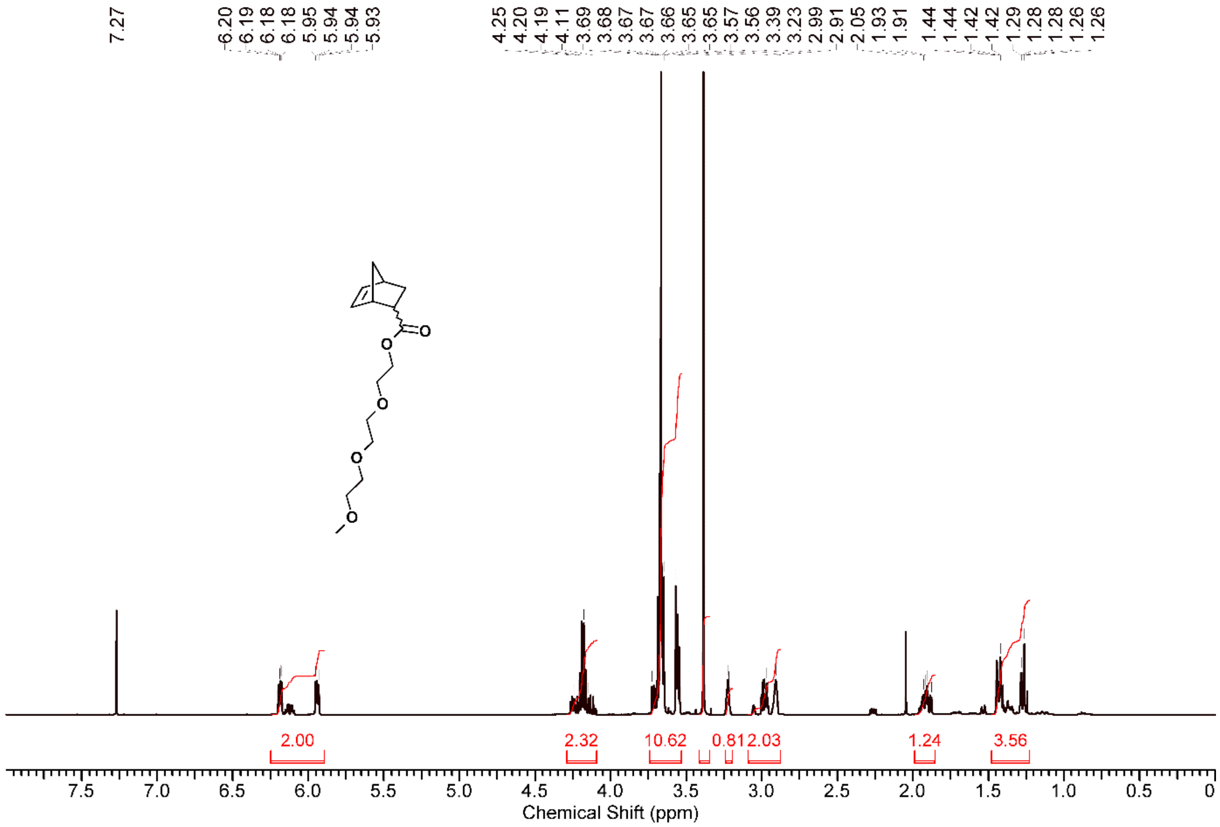

Fig. S42: ^{1}H NMR ($CDCl_3$, 400 MHz) spectrum of **M3**.

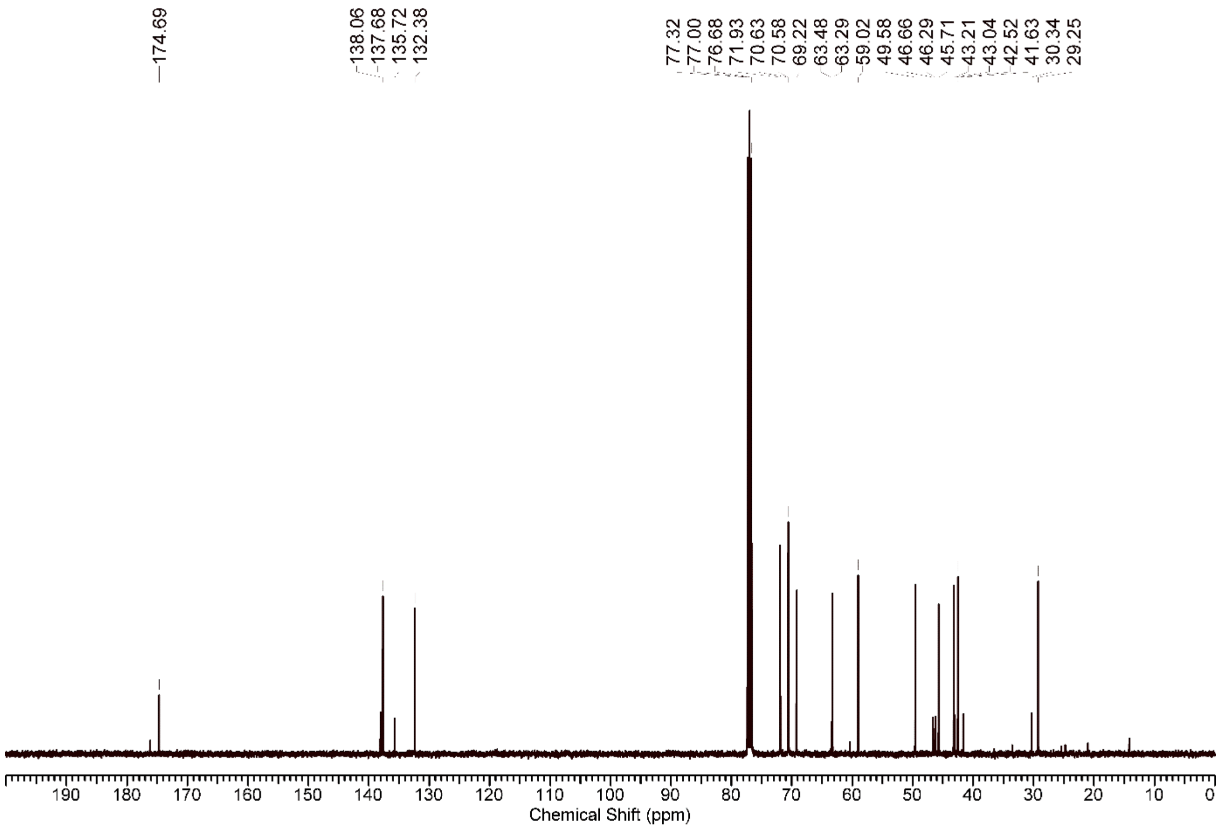

Fig. S43: ^{13}C NMR ($CDCl_3$, 101 MHz) spectrum of **M3.**

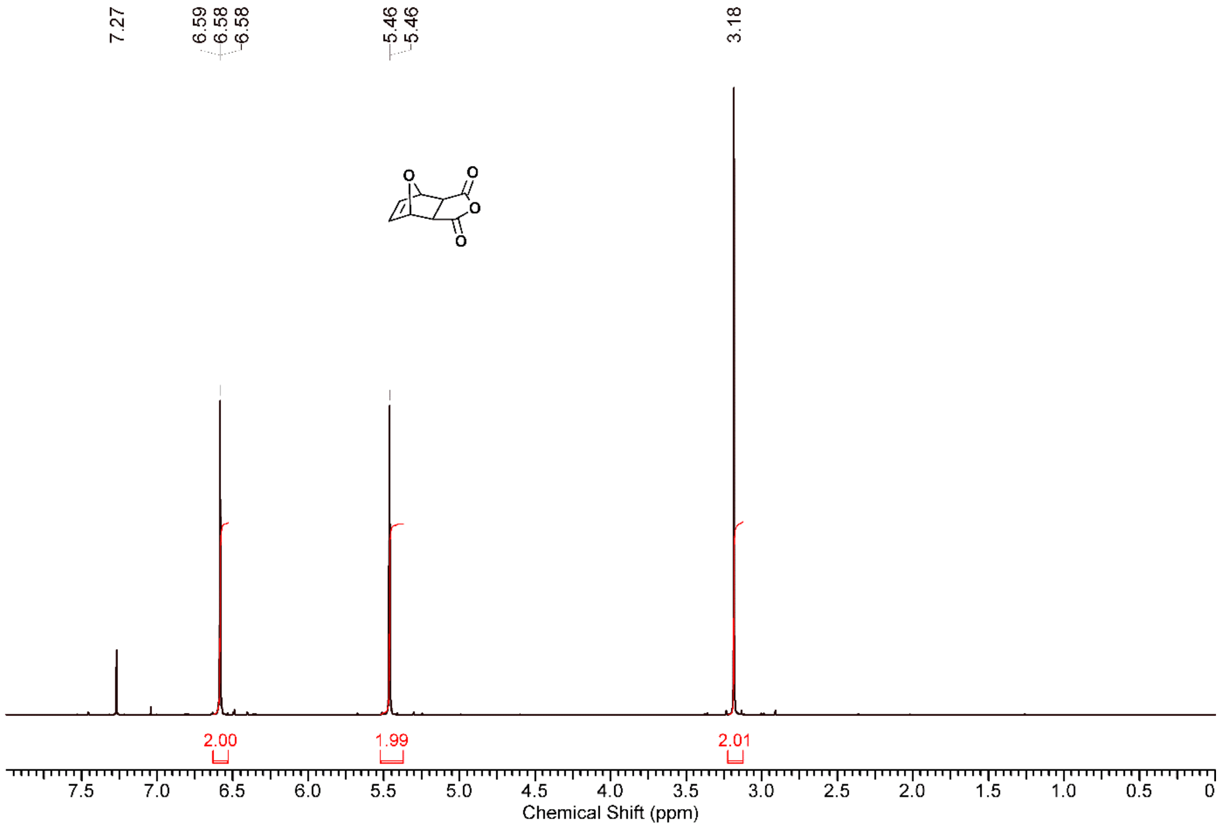

Fig. S44: ^{1}H NMR ($CDCl_3$, 400 MHz) spectrum of **I3**.

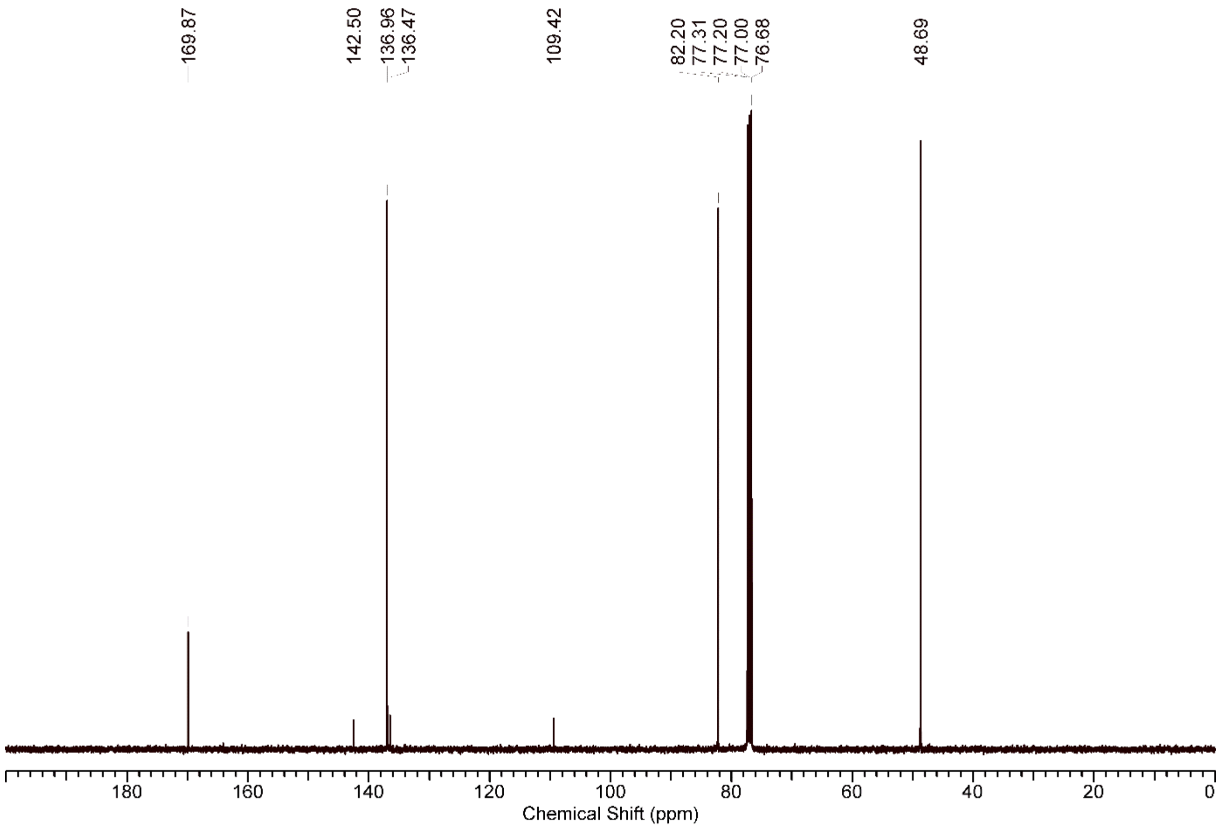

Fig. S45: ^{13}C NMR ($CDCl_3$, 101 MHz) spectrum of **I3.**

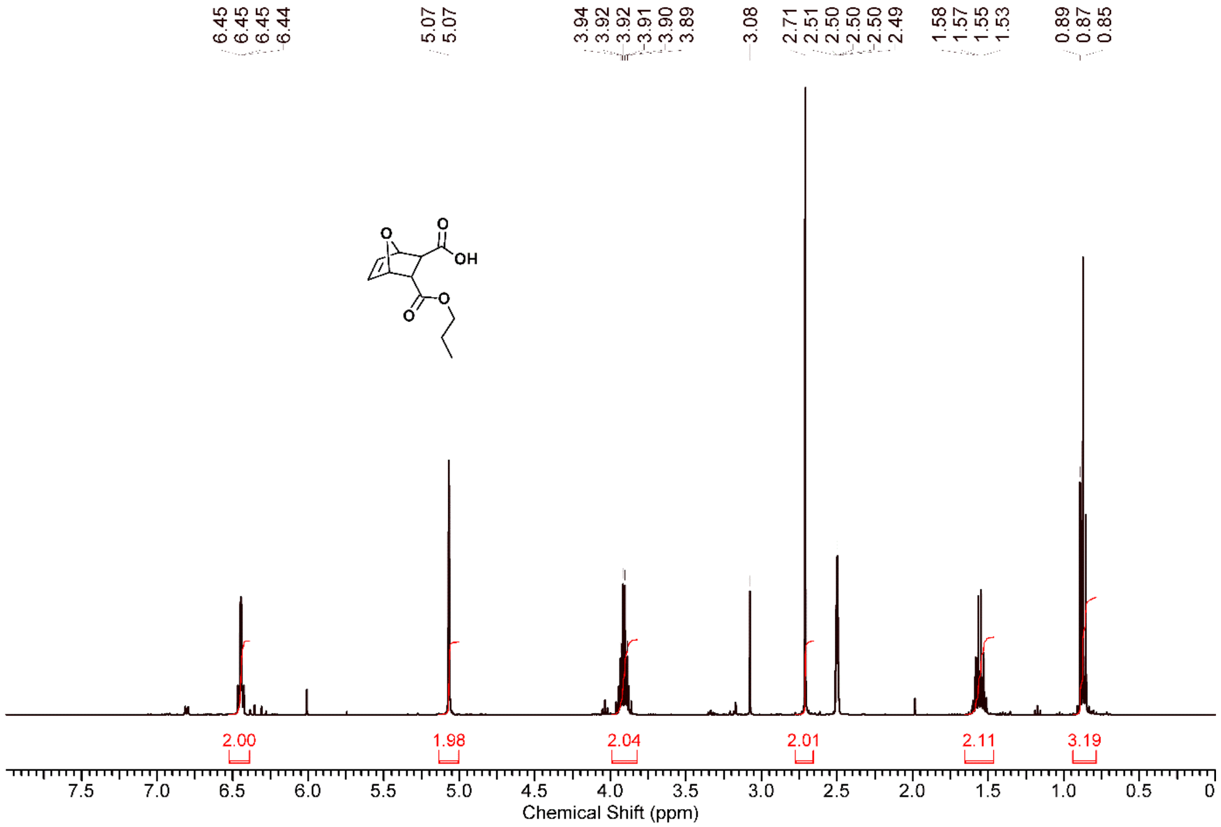

Fig. S46: ^{1}H NMR (DMSO-d_6, 400 MHz) spectrum of **I4**.

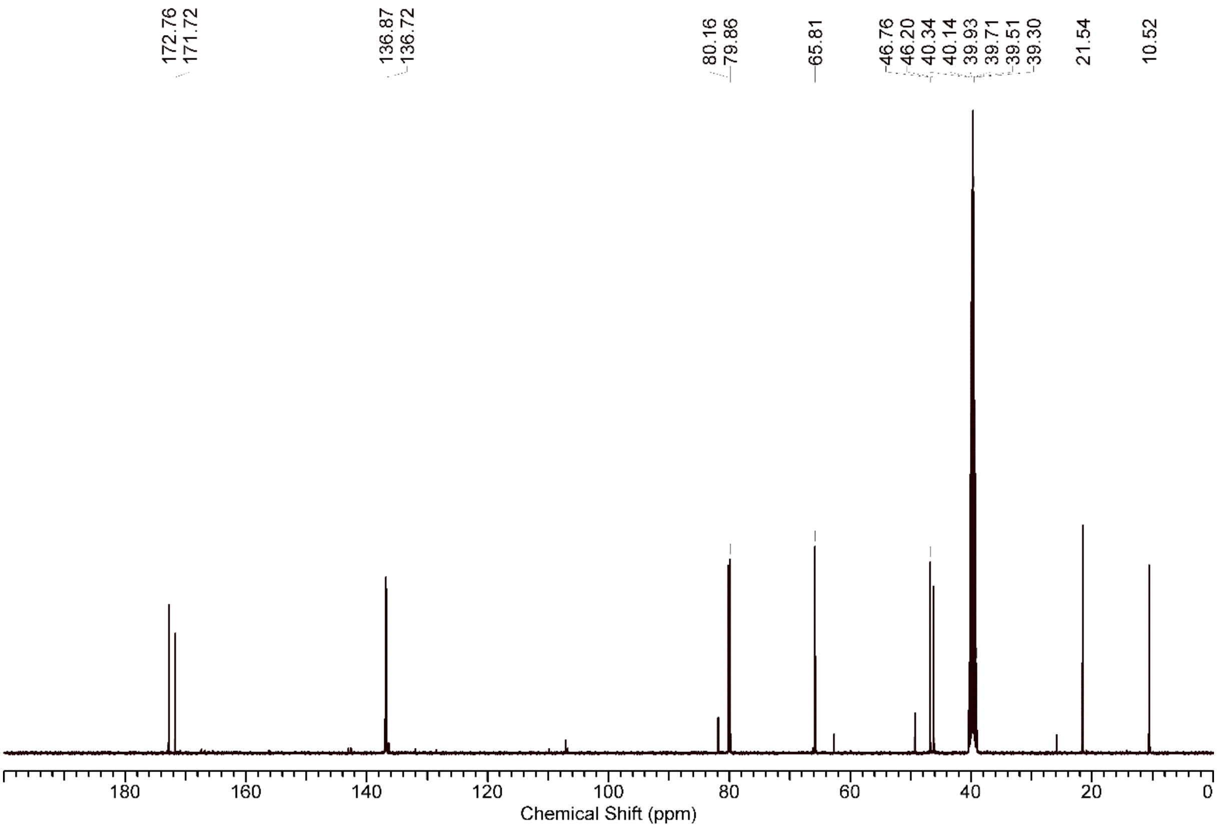

Fig. S47: ^{13}C NMR (DMSO-d_6, 400 MHz) spectrum of **I4**.

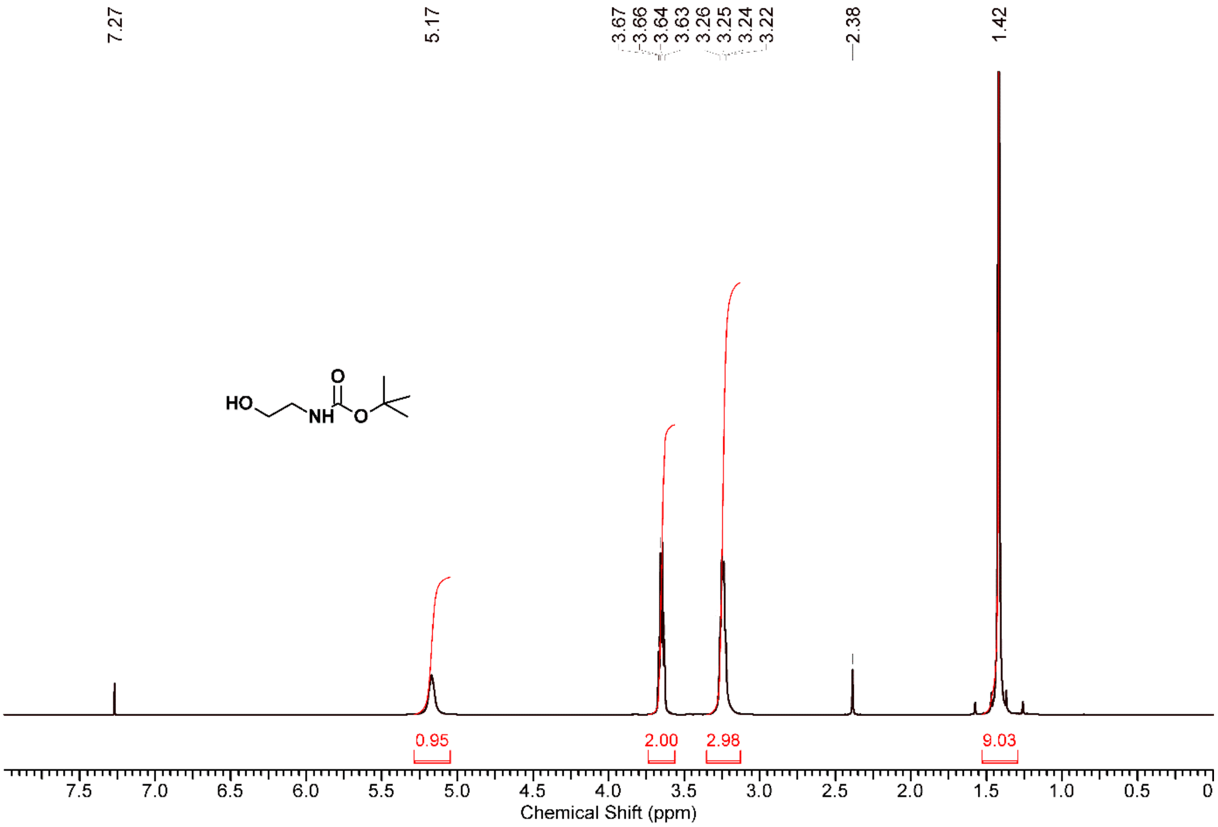

Fig. S48: ^{1}H NMR ($CDCl_3$, 400 MHz) spectrum of **I5**.

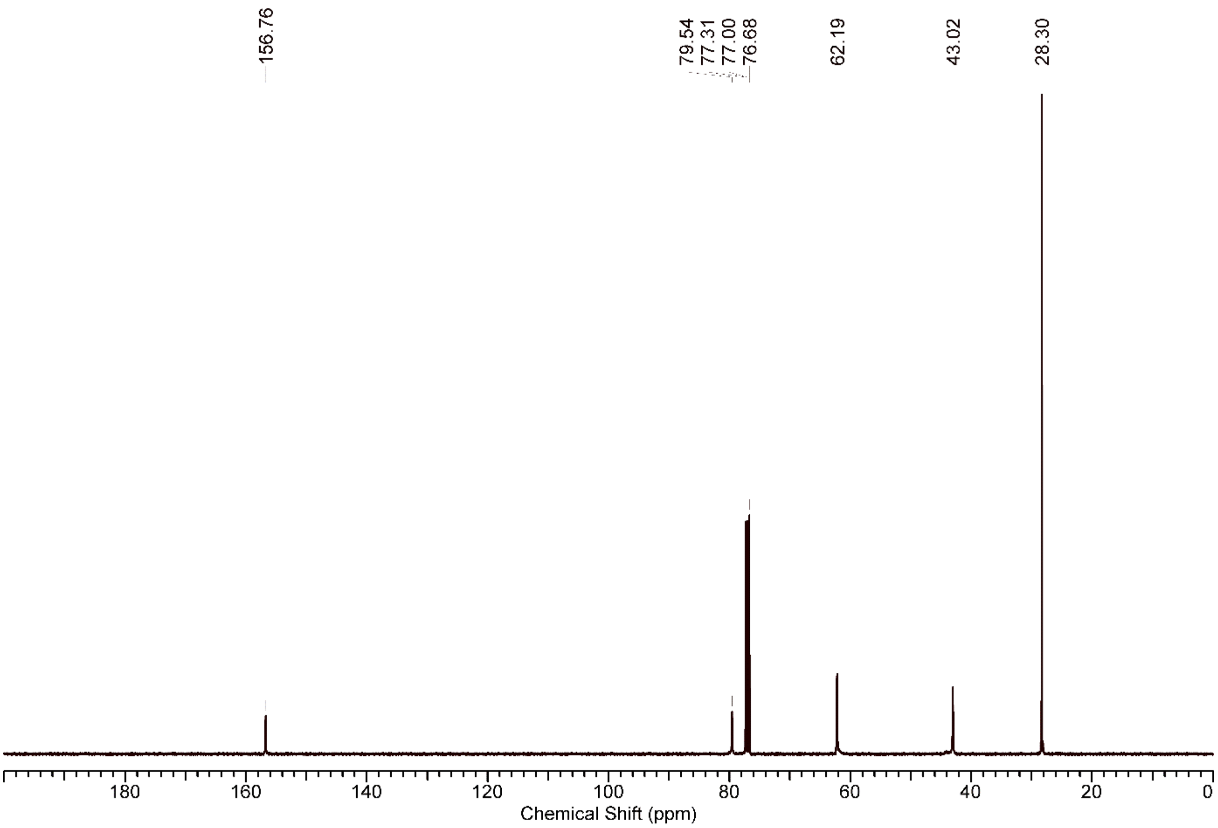

Fig. S49: ^{13}C NMR ($CDCl_3$, 400 MHz) spectrum of **I5**.

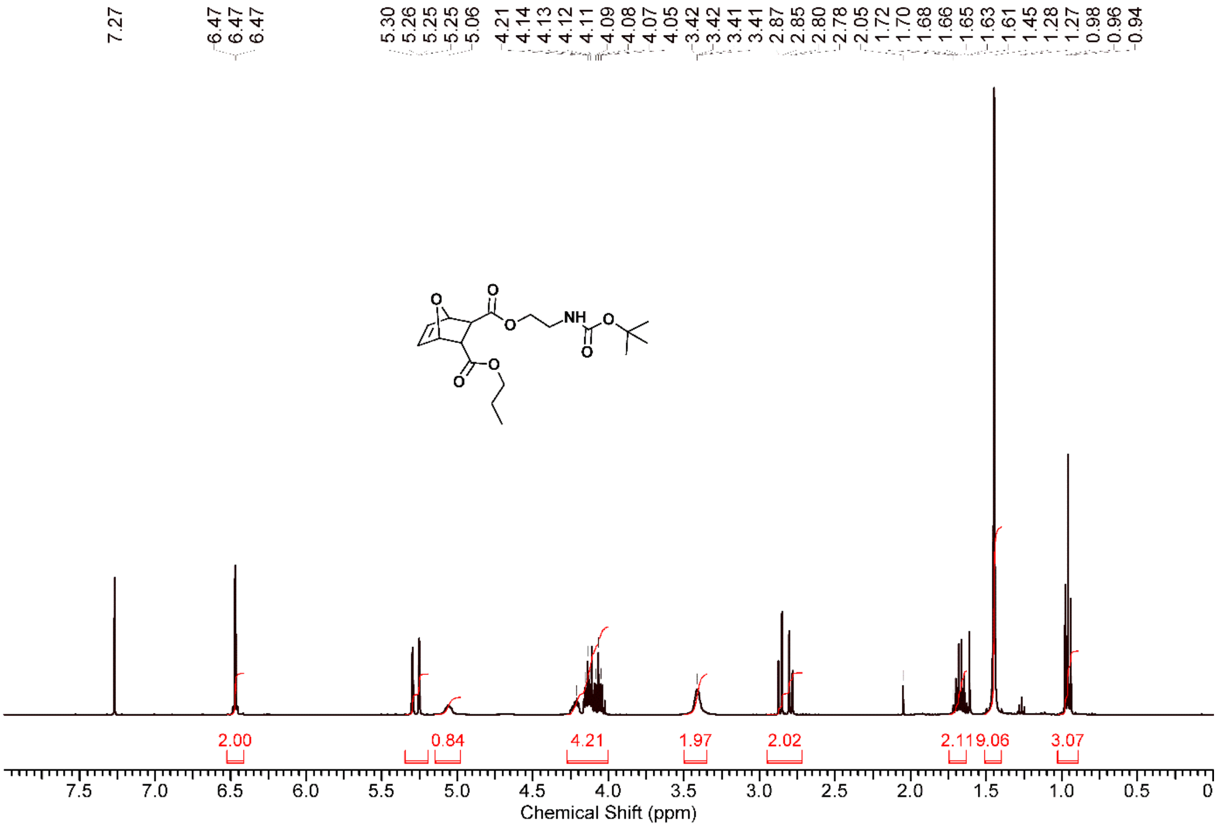

Fig. S50: ^{1}H NMR ($CDCl_3$, 400 MHz) spectrum of **M4**.

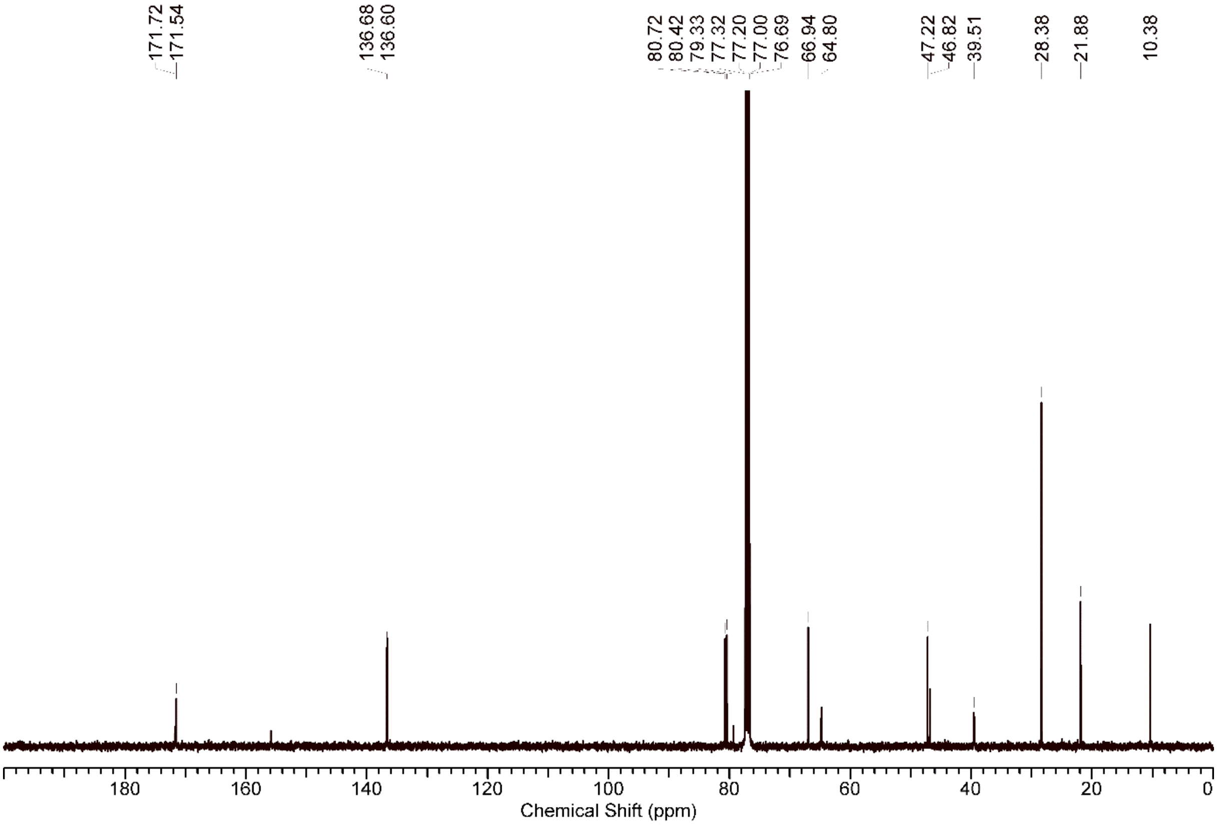

Fig. S51: ^{13}C NMR ($CDCl_3$, 400 MHz) spectrum of **M4**.

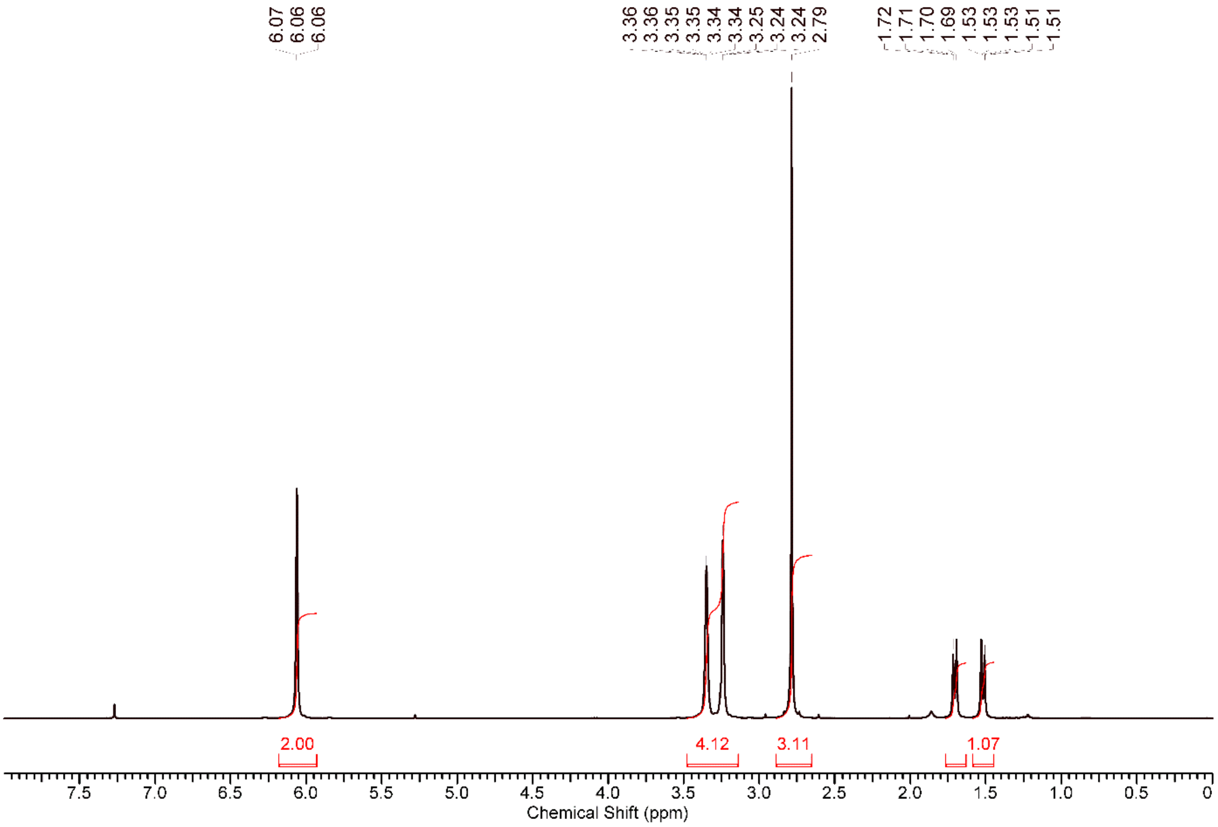

Fig. S52: ^1H NMR ($CDCl_3$, 400 MHz) spectrum of **endo-MNI**.

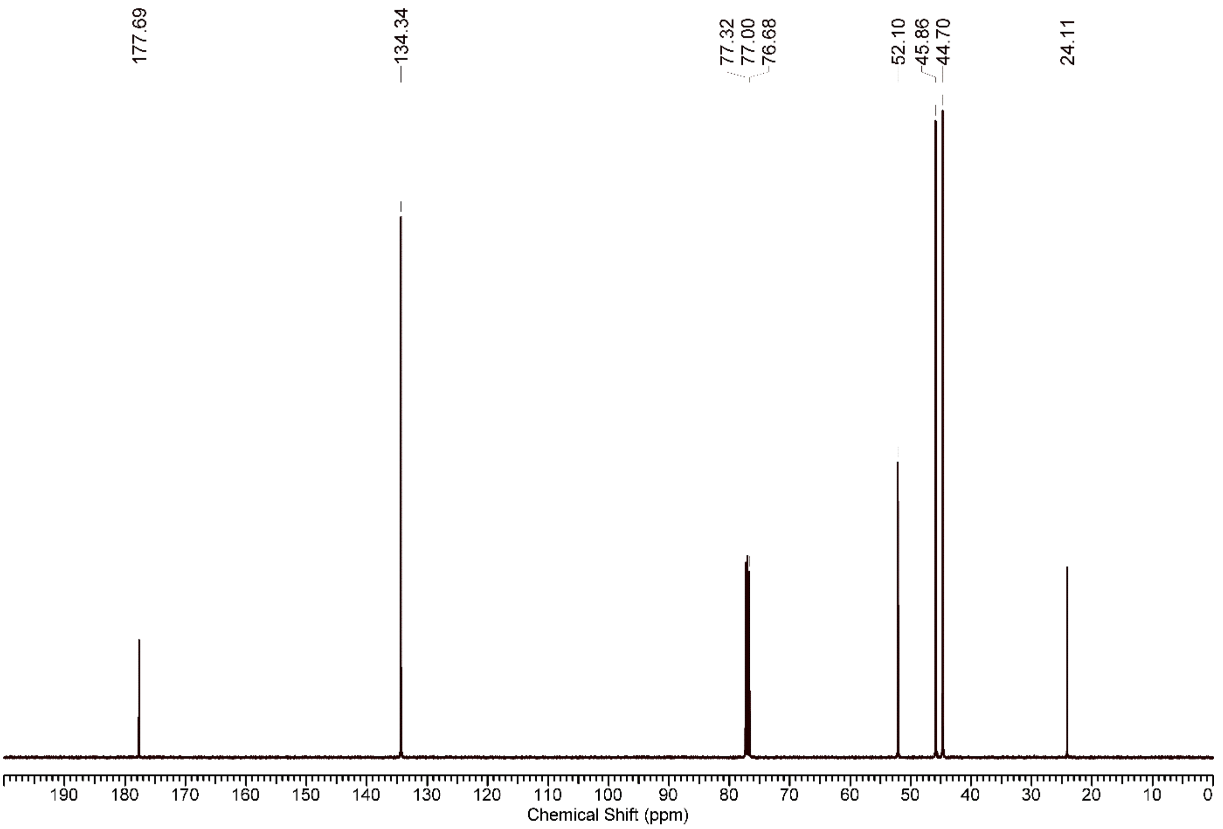

Fig. S53: ^{13}C NMR ($CDCl_3$, 400 MHz) spectrum of **endo-MNI**.

NMR spectra of Polymers:

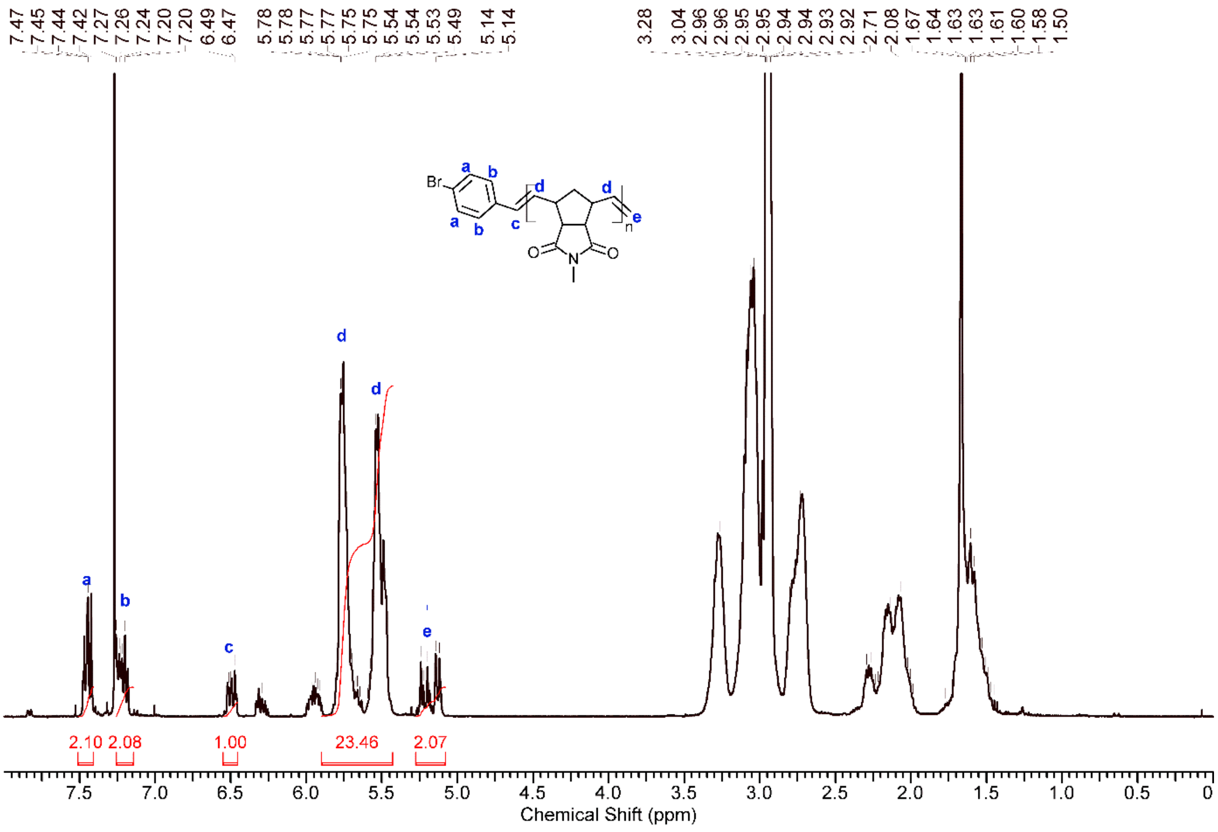

Fig. S54: ^{1}H NMR ($CDCl_3$, 400 MHz) spectrum of **P4**.

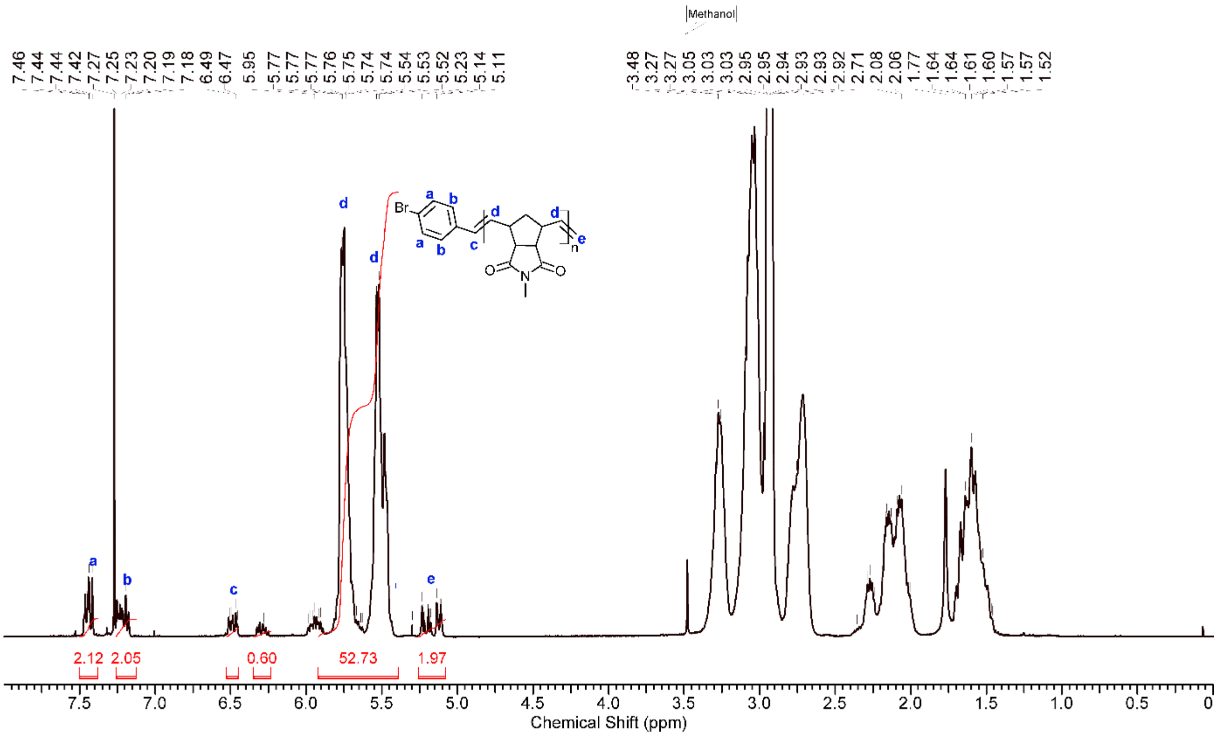

Fig. S55: ^{1}H NMR ($CDCl_3$, 400 MHz) spectrum of **P5**.

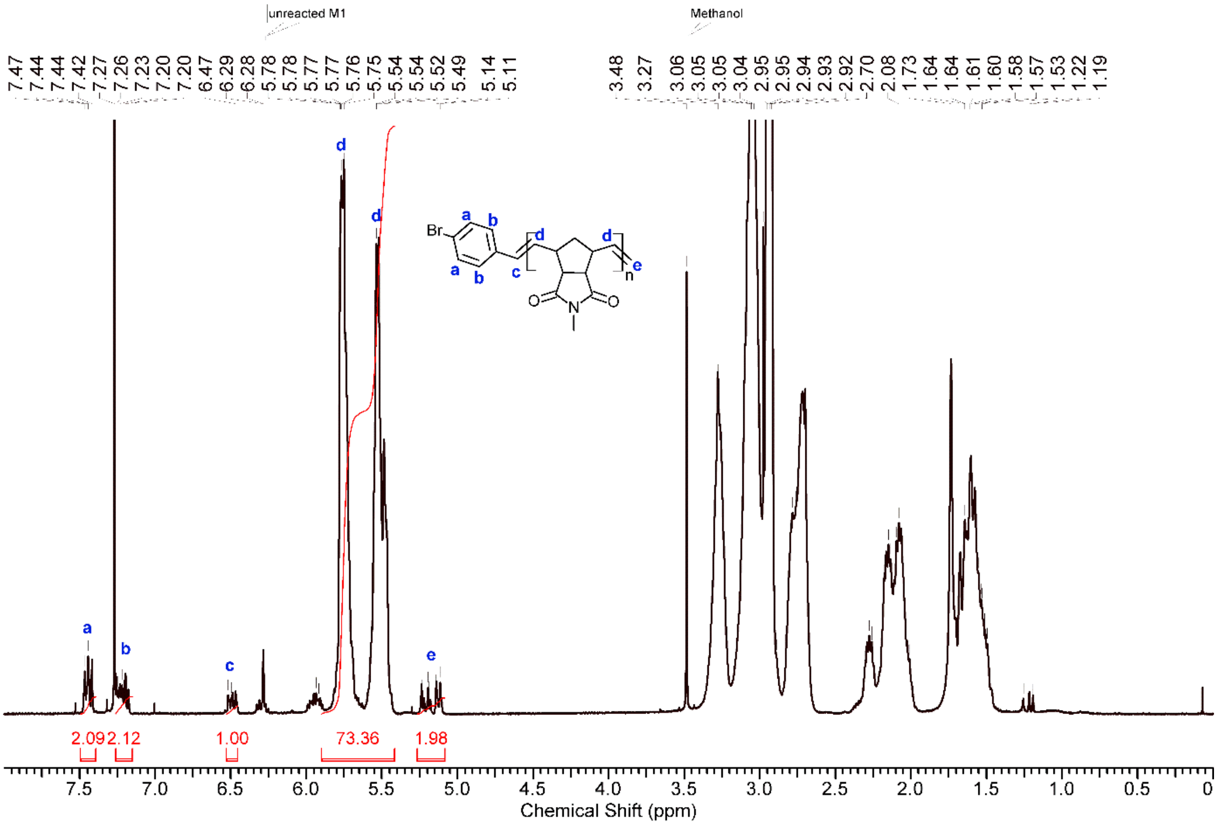

Fig. S56: ^{1}H NMR ($CDCl_3$, 400 MHz) spectrum of **P6**.

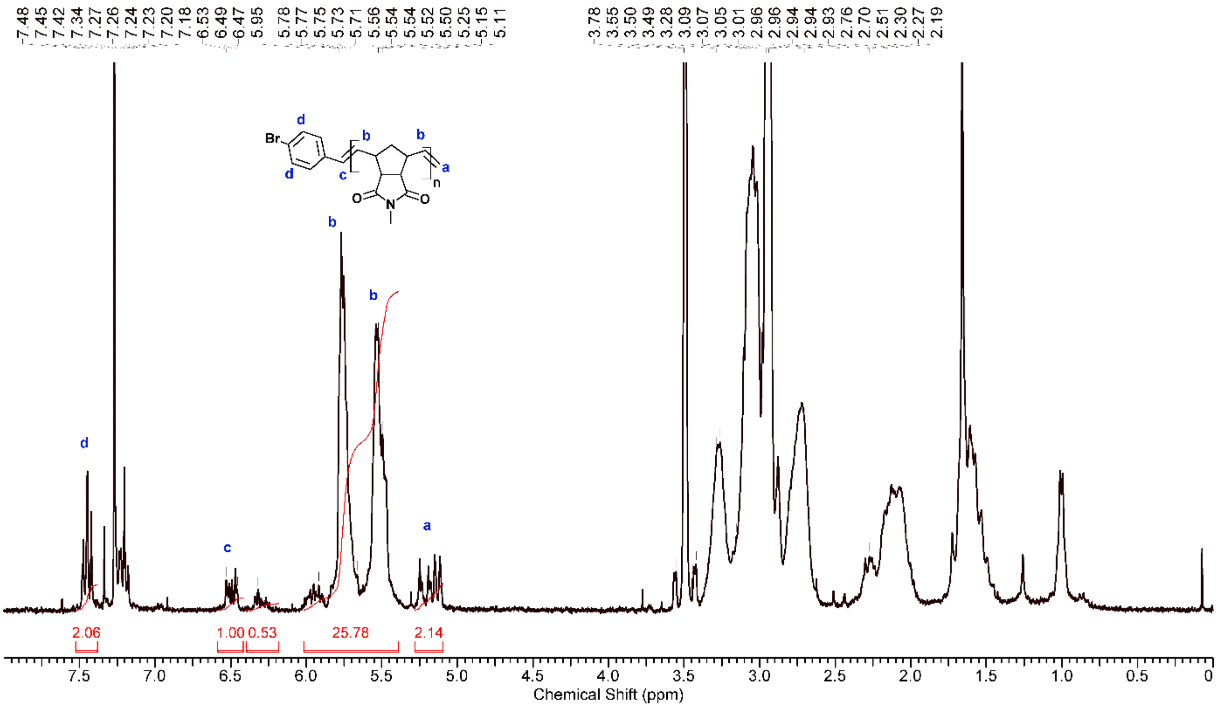

Fig. S57: ^{1}H NMR ($CDCl_3$, 400 MHz) spectrum of **P8**.

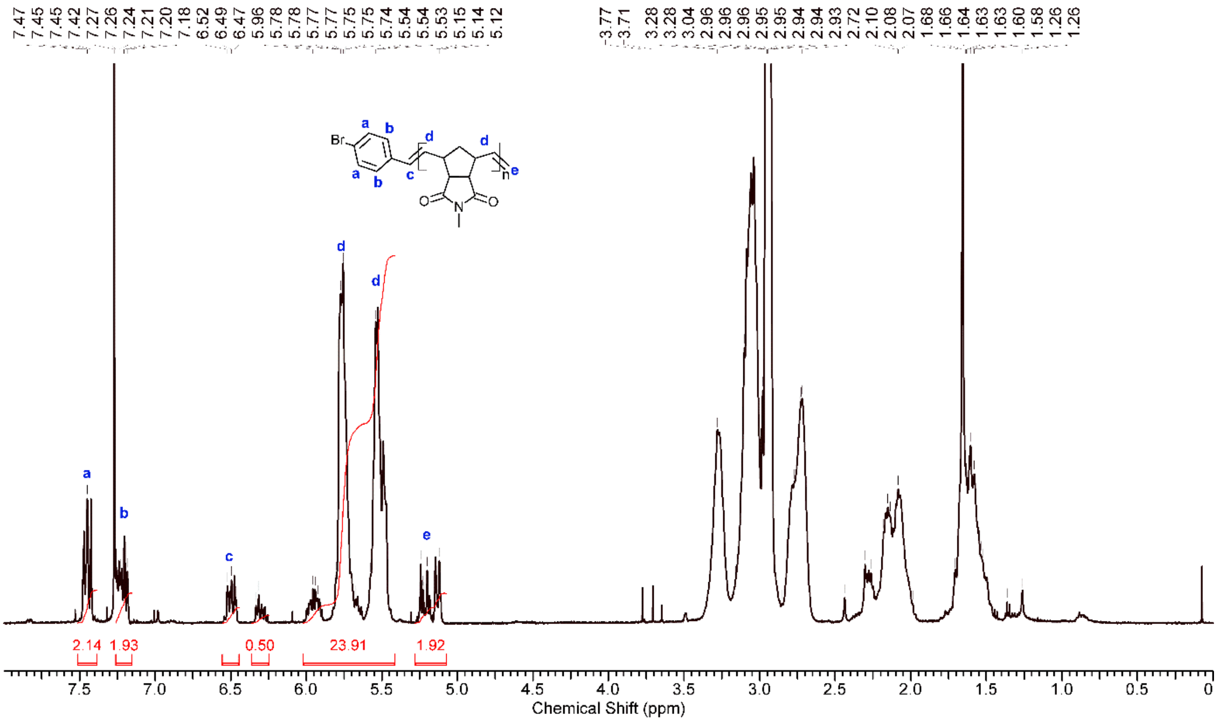

Fig. S58: ^{1}H NMR ($CDCl_3$, 400 MHz) spectrum of **P9**.

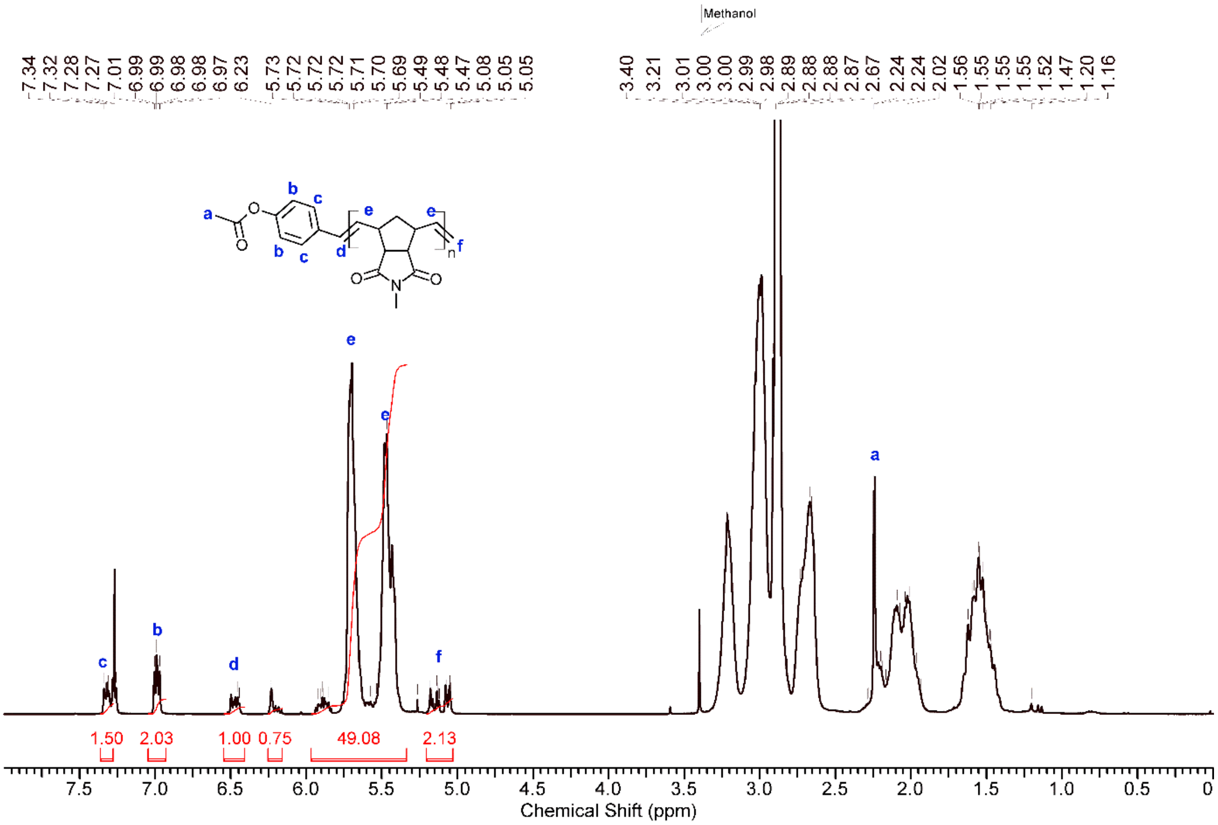

Fig. S59: ^{1}H NMR ($CDCl_3$, 400 MHz) spectrum of **P10**.

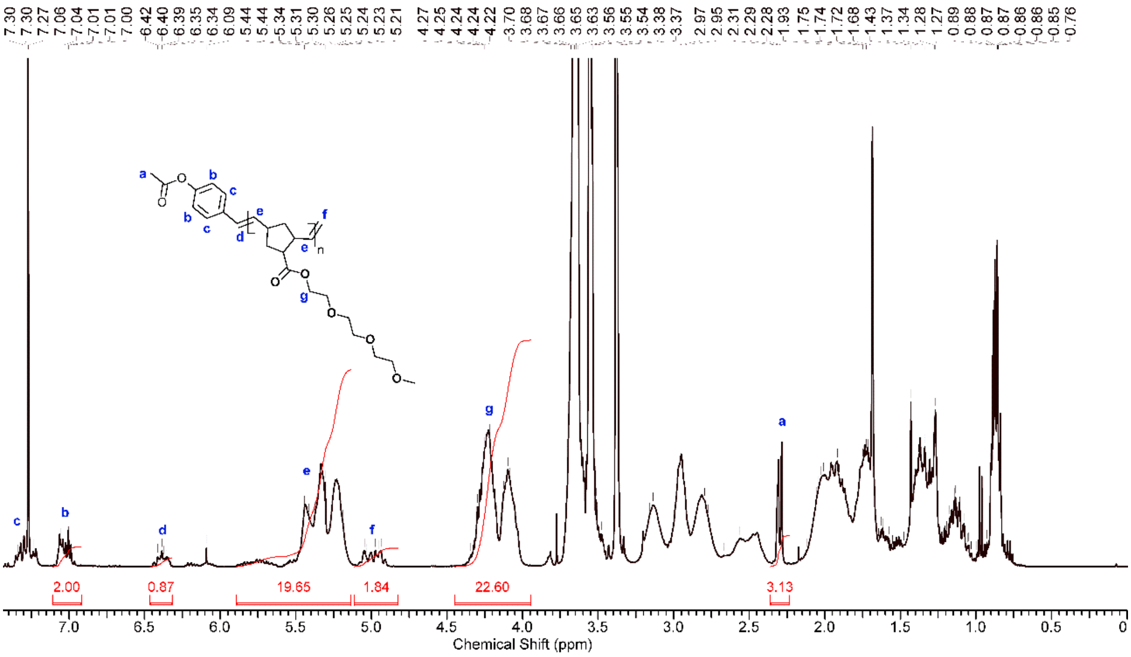

Fig. S60: ^{1}H NMR ($CDCl_3$, 400 MHz) spectrum of **P11**.

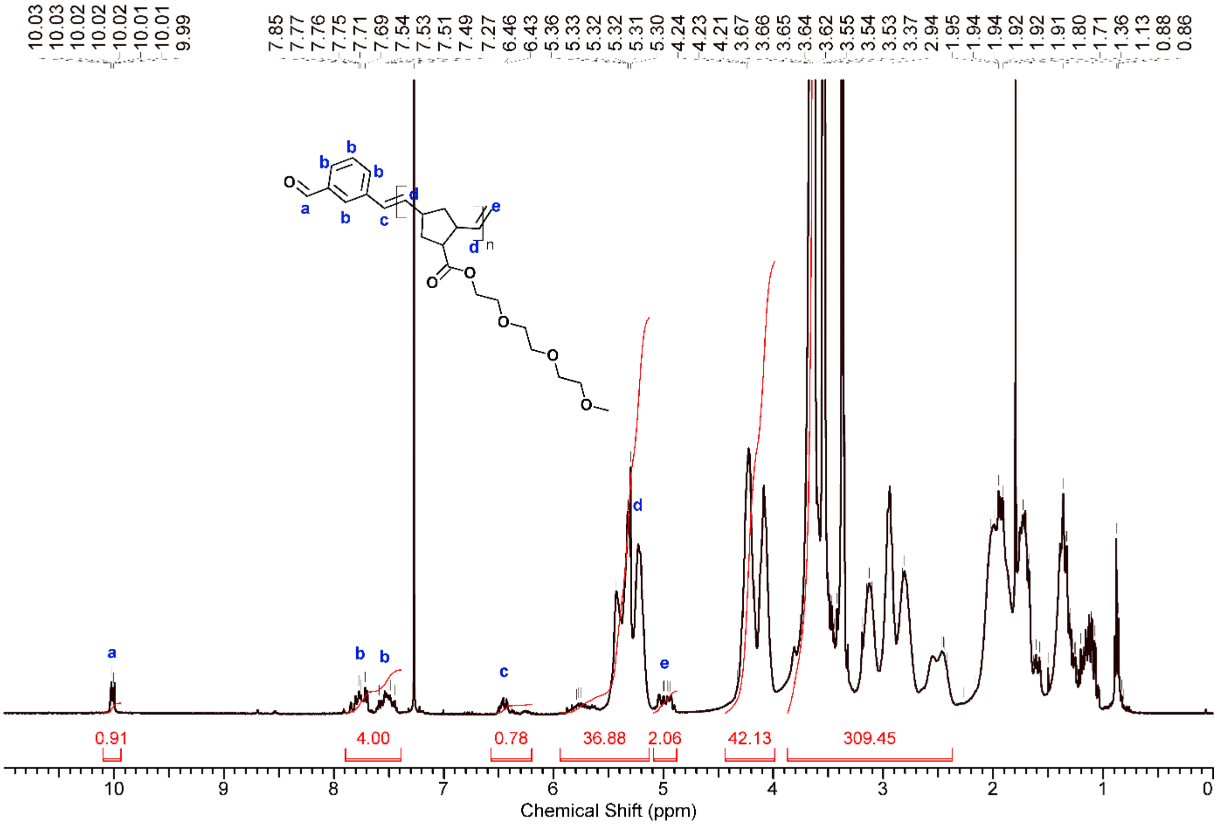

Fig. S61: ^{1}H NMR ($CDCl_3$, 400 MHz) spectrum of **P12**.

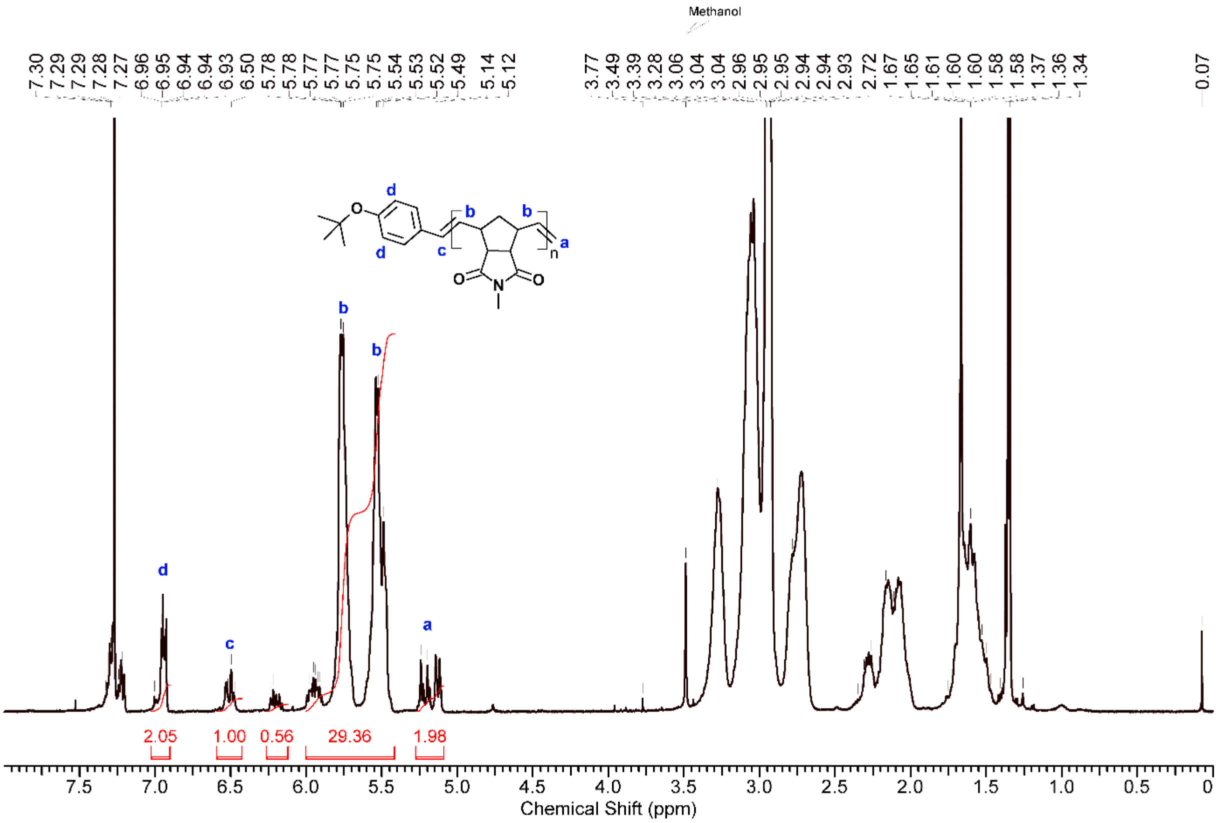

Fig. S62: ^{1}H NMR ($CDCl_3$, 400 MHz) spectrum of **P13**.

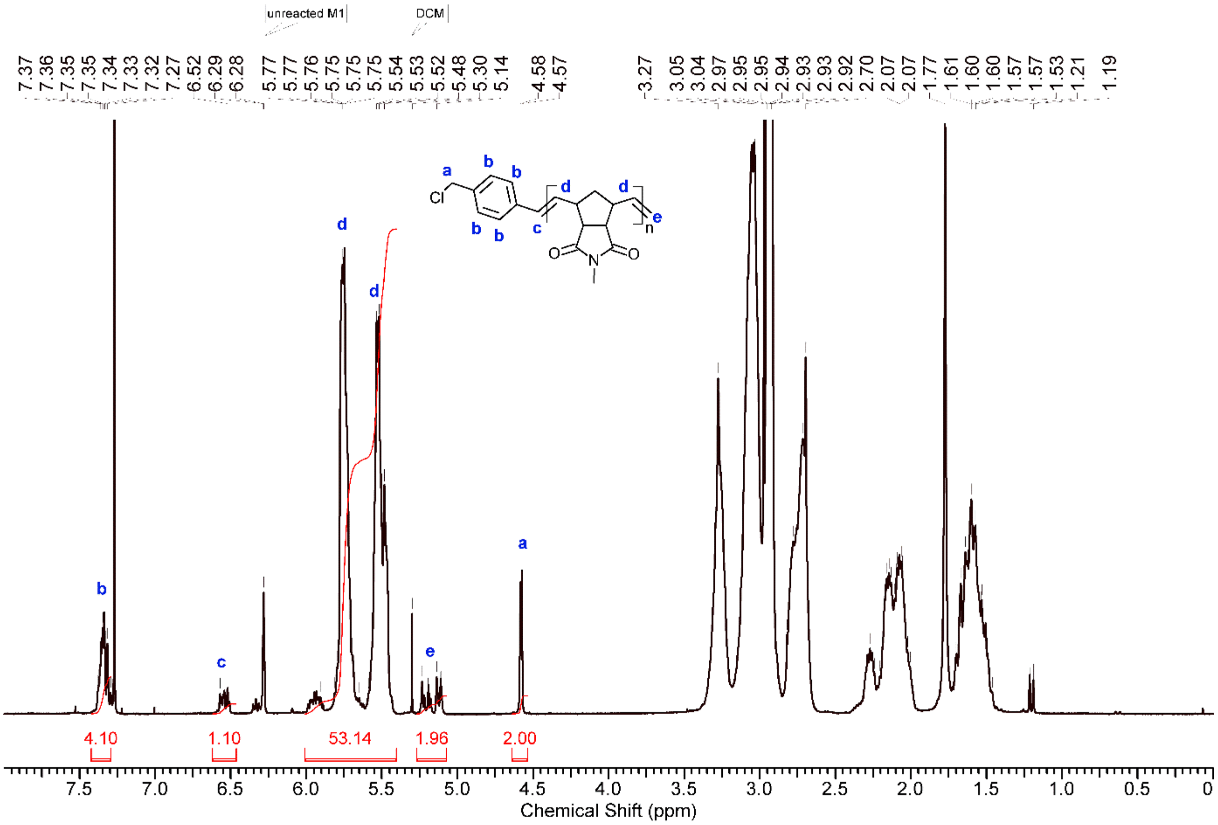

Fig. S63: ^{1}H NMR ($CDCl_3$, 400 MHz) spectrum of **P15**.

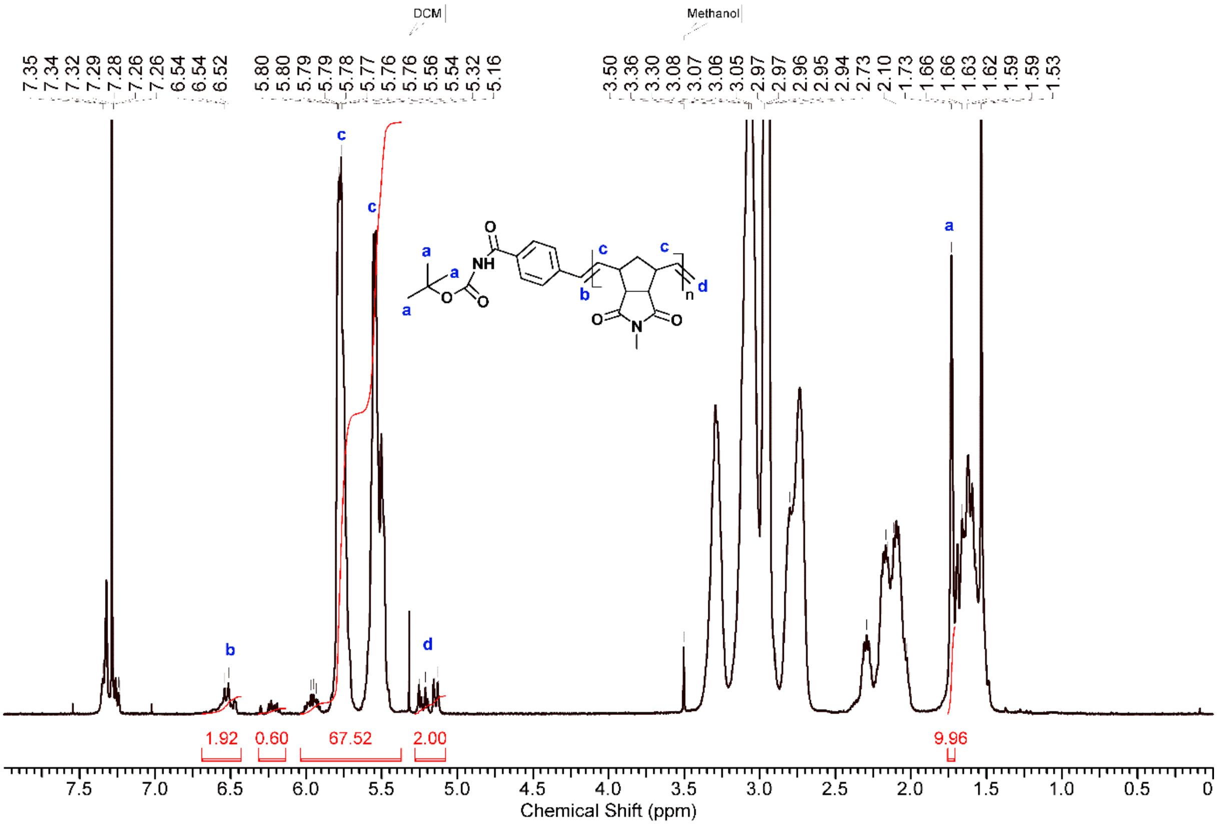

Fig. S64: ^{1}H NMR ($CDCl_3$, 400 MHz) spectrum of **P16**.

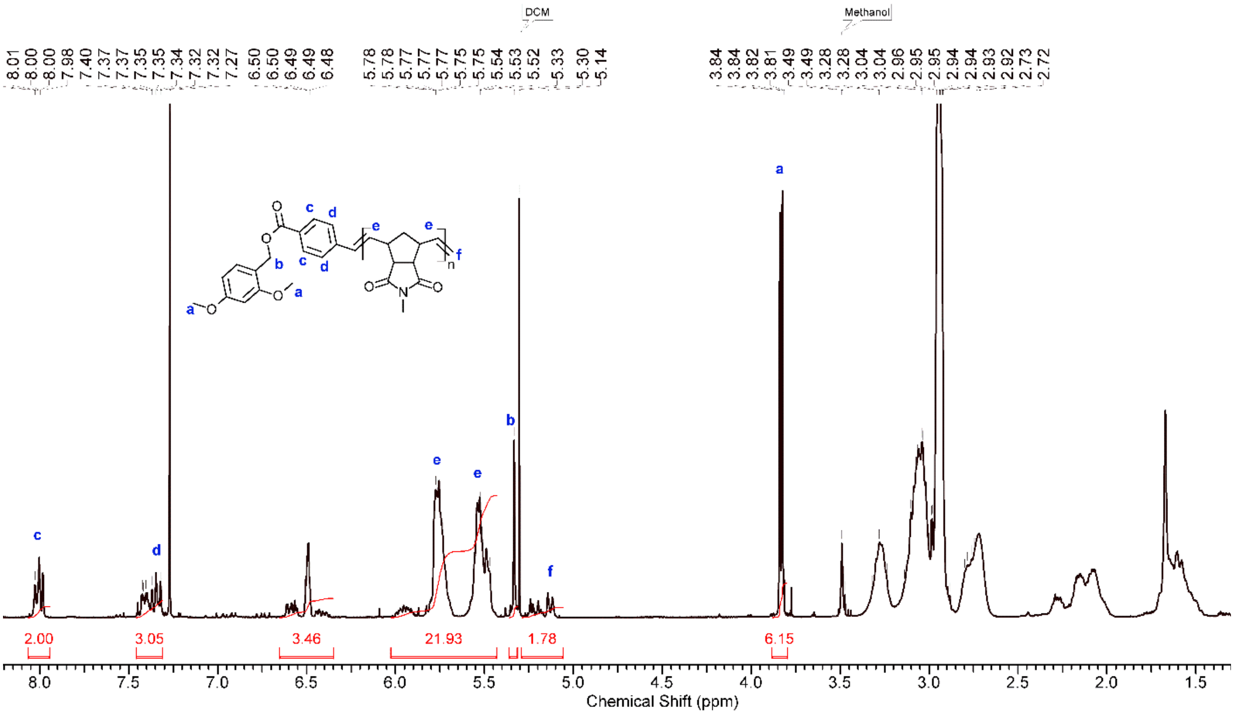

Fig. S65: ^{1}H NMR ($CDCl_3$, 400 MHz) spectrum of **P17**.

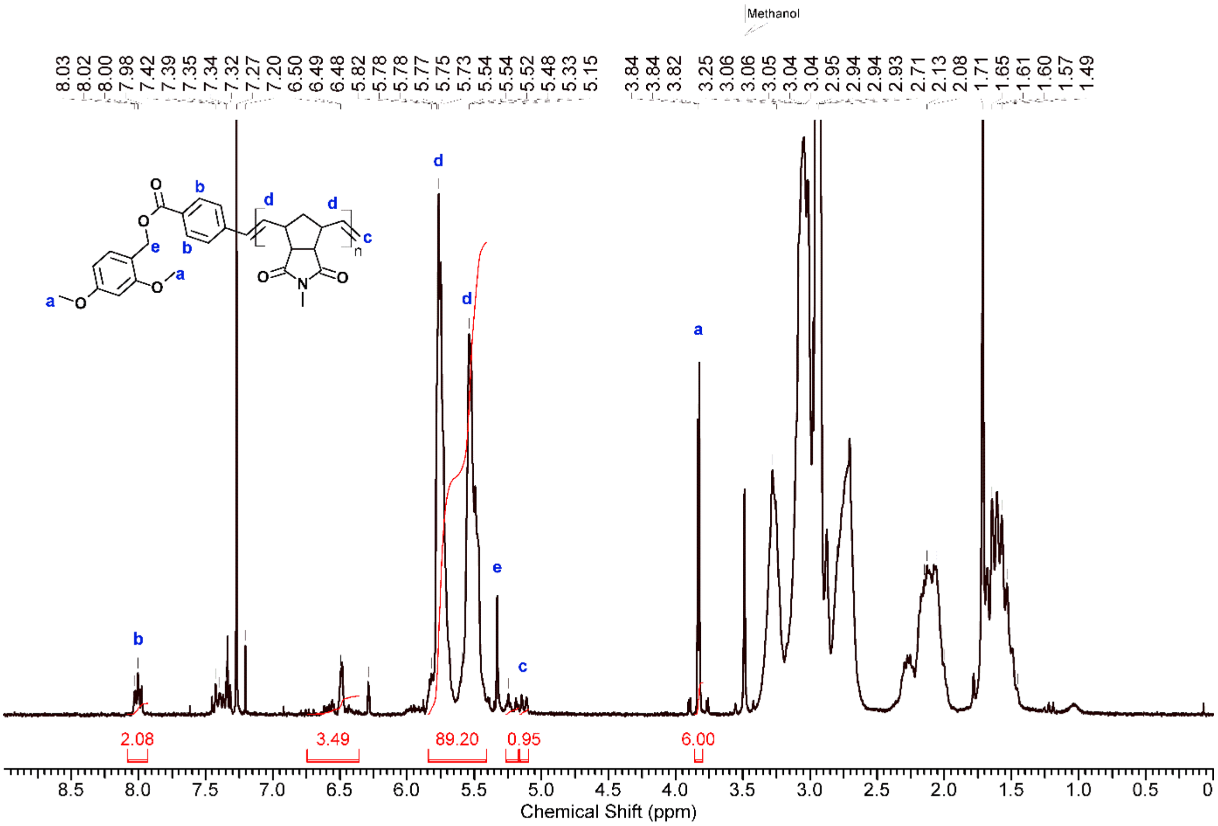

Fig. S66: ^{1}H NMR ($CDCl_3$, 400 MHz) spectrum of **P18**.

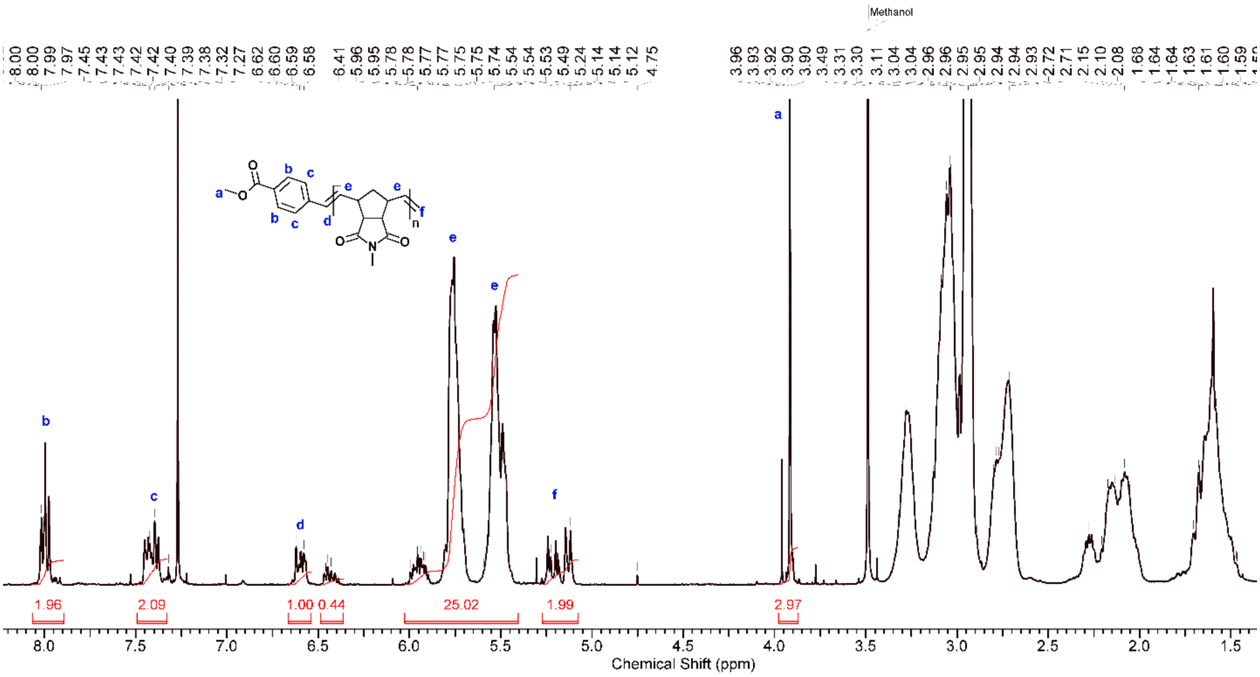

Fig. S67: ^{1}H NMR ($CDCl_3$, 400 MHz) spectrum of **P19**.

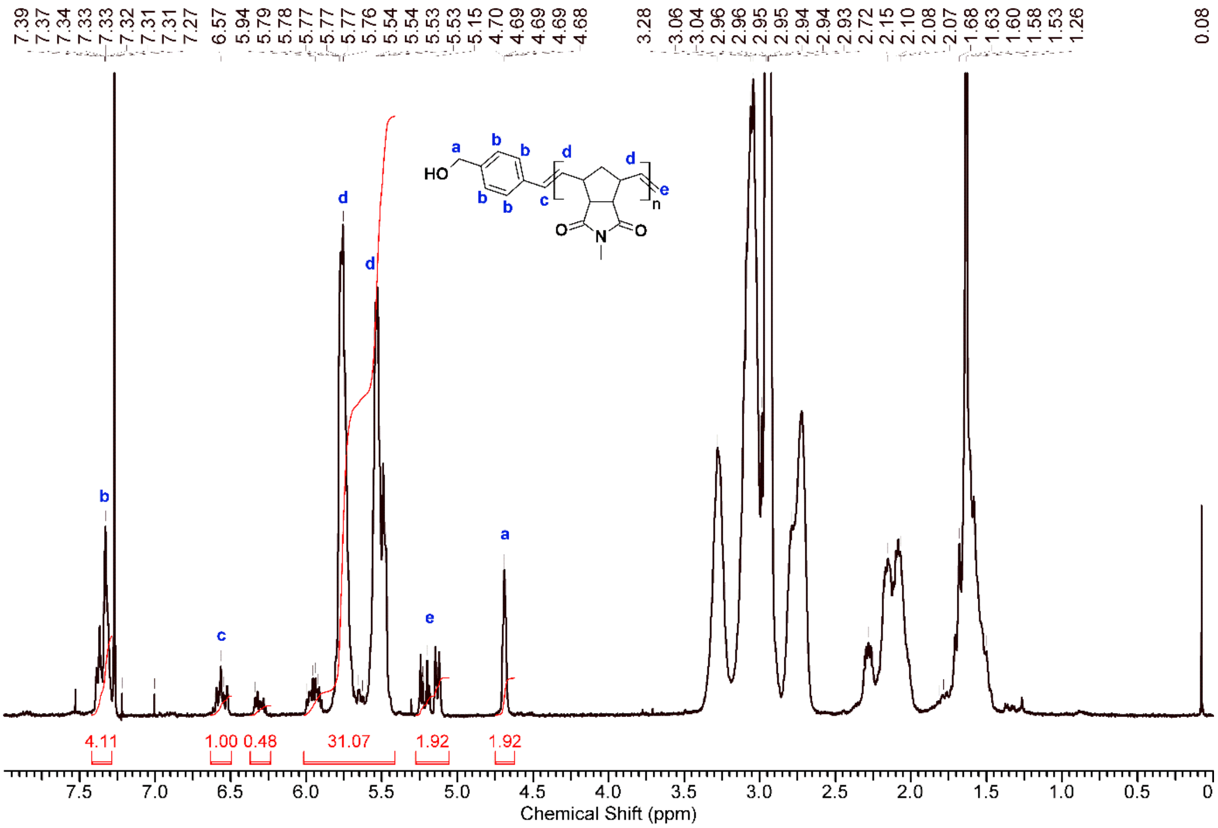

Fig. S68: ^{1}H NMR ($CDCl_3$, 400 MHz) spectrum of **P20**.

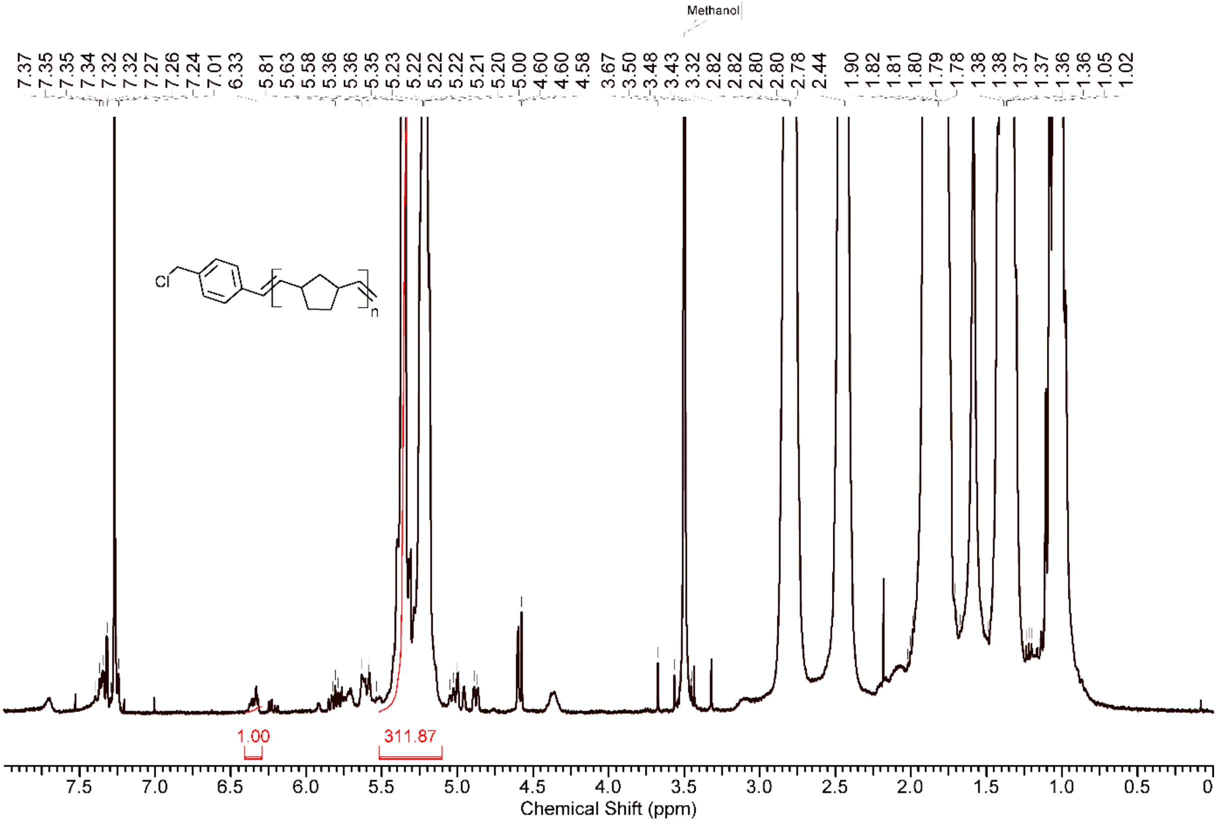

Fig. S69: ^{1}H NMR ($CDCl_3$, 400 MHz) spectrum of **P21**.

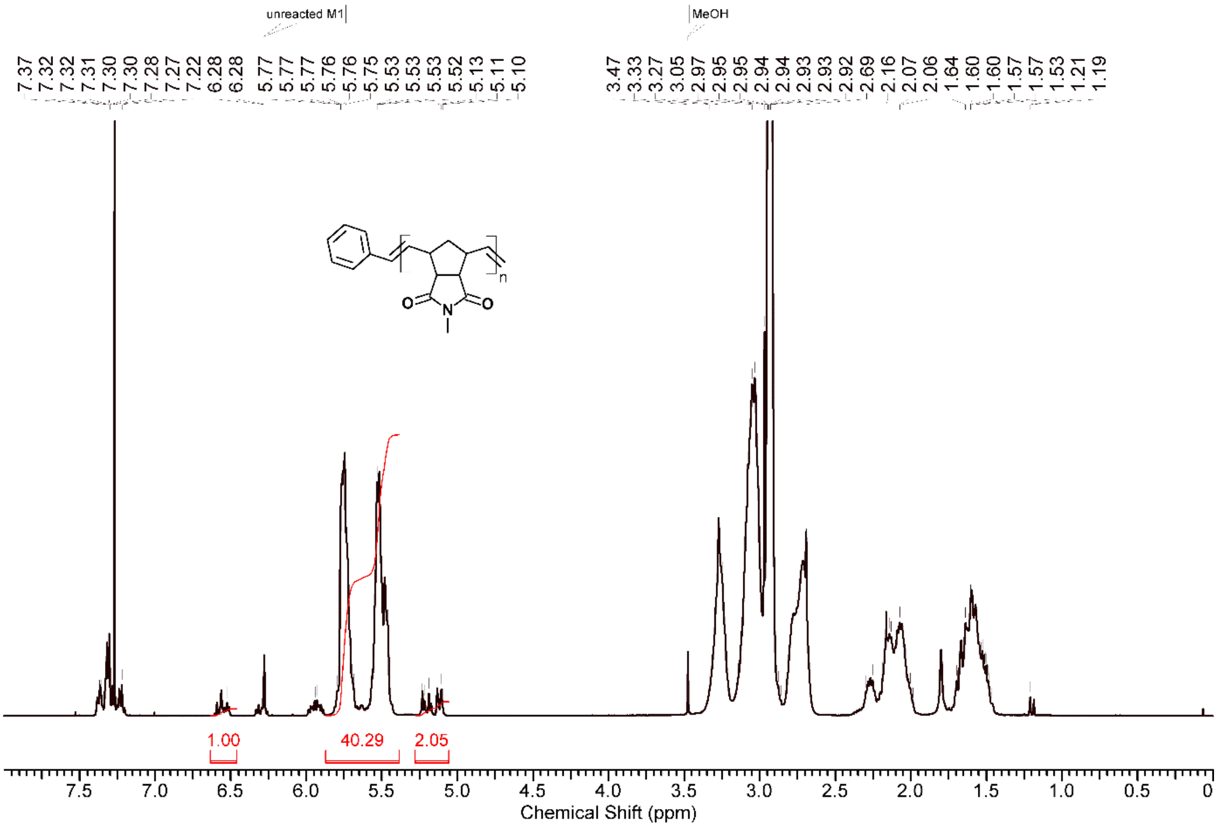

Fig. S70: ^{1}H NMR ($CDCl_3$, 400 MHz) spectrum of **P23**.

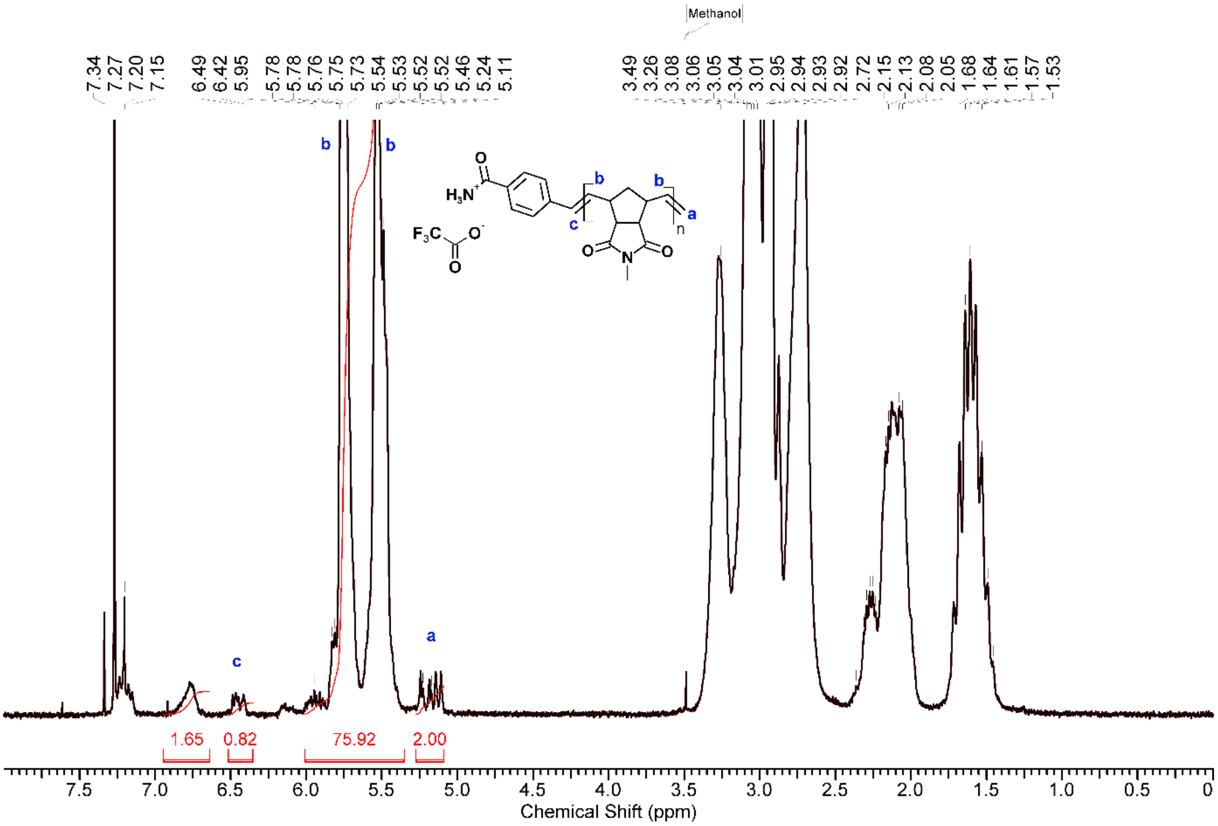

Fig. S71: ^{1}H NMR ($CDCl_3$, 400 MHz) spectrum of **P24**.

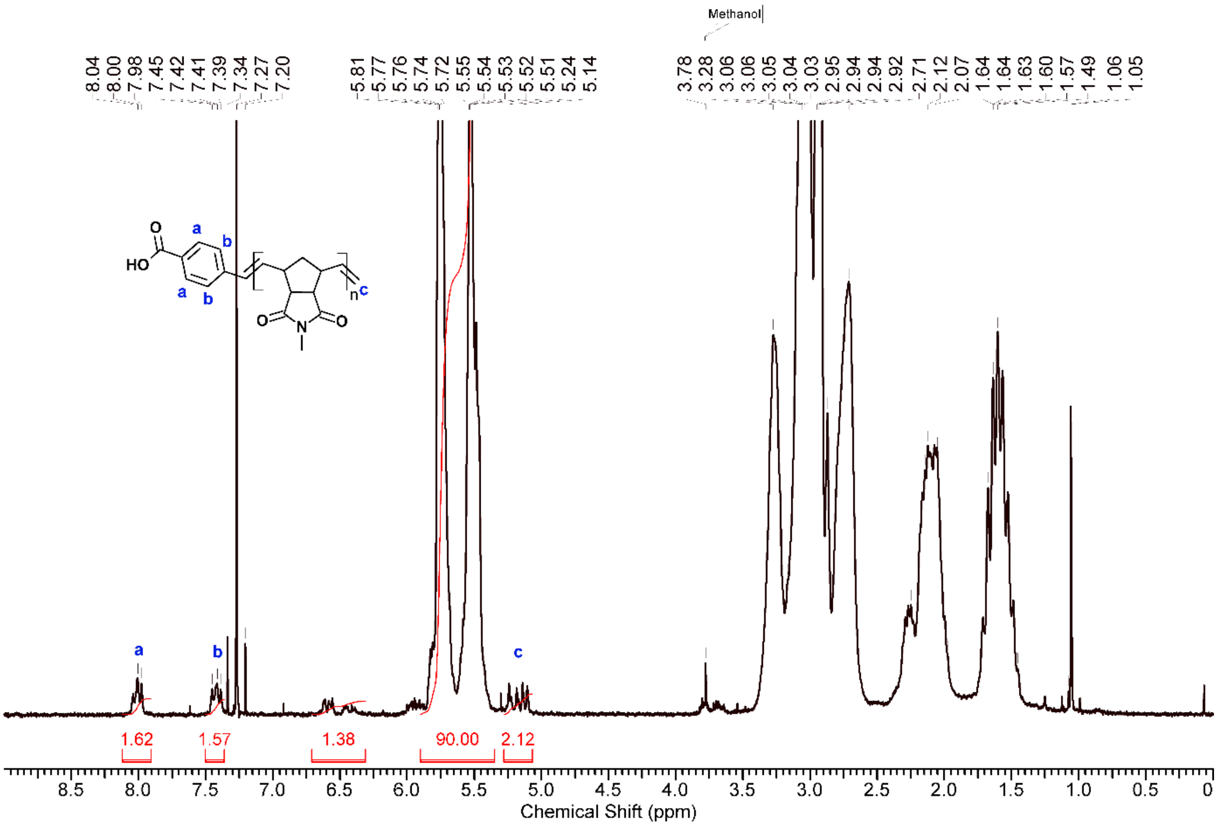

Fig. S72: ^{1}H NMR ($CDCl_3$, 400 MHz) spectrum of **P25**.

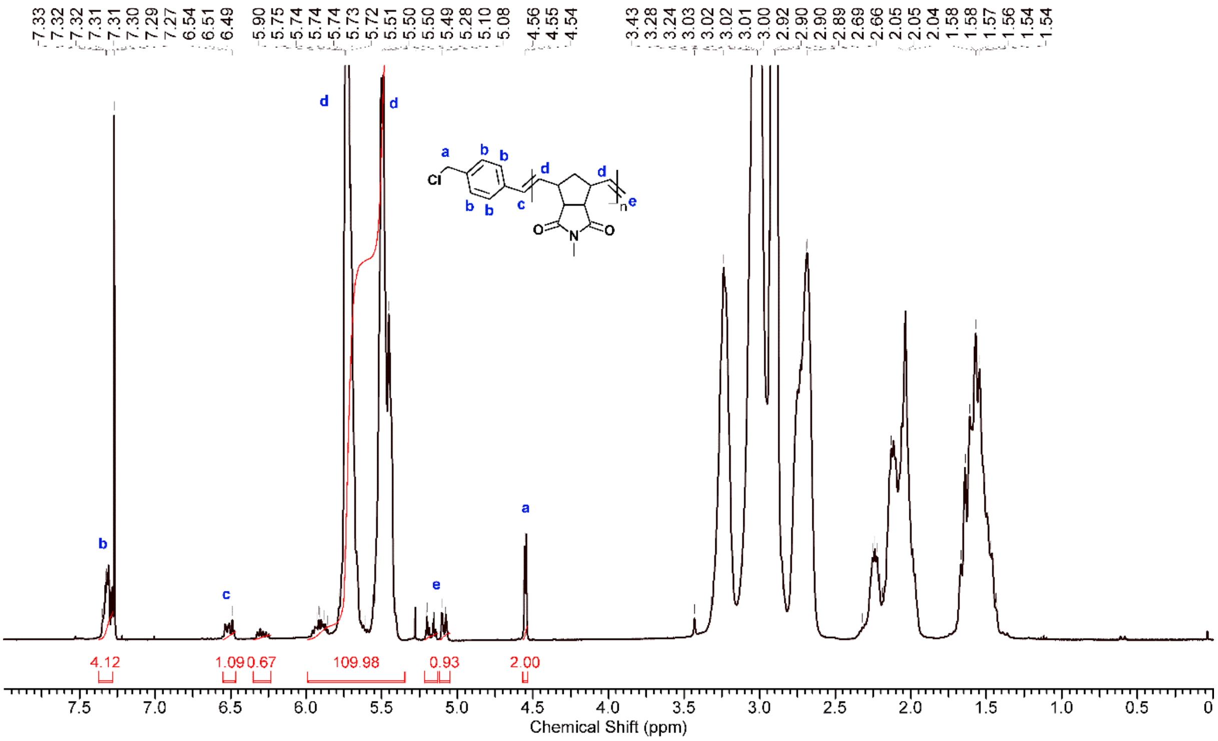

Fig. S73: ^{1}H NMR ($CDCl_3$, 400 MHz) spectrum of **P26**.

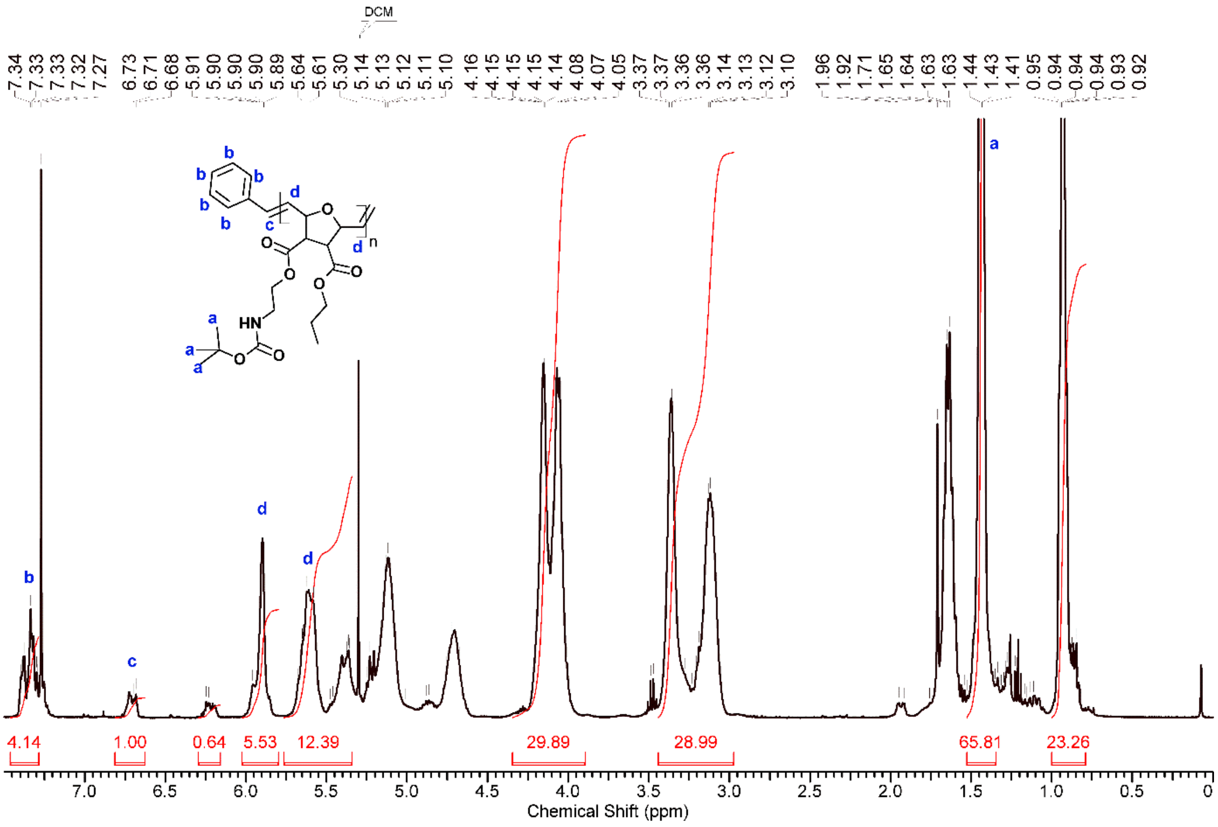

Fig. S74: ^{1}H NMR (CDCl$_3$, 400 MHz) spectrum of **P27**.

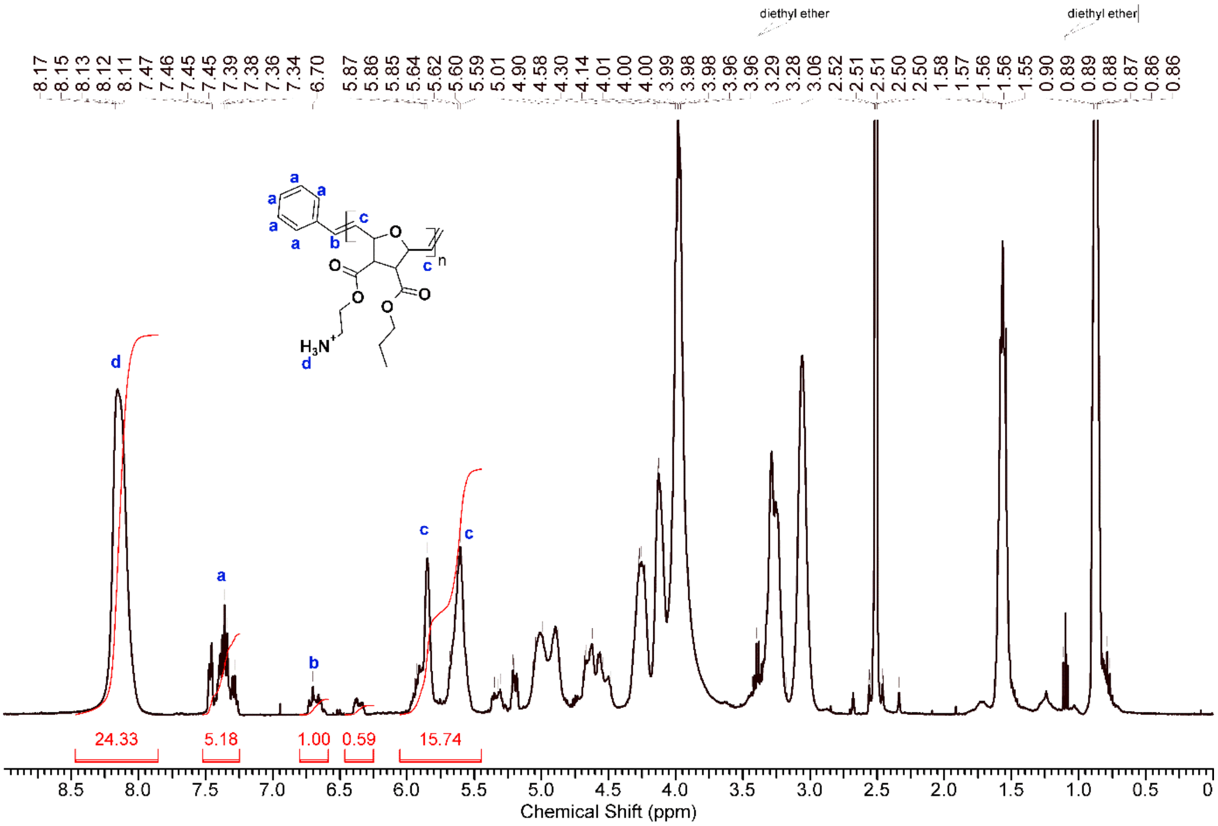

Fig. S75: ^{1}H NMR (DMSO-d6, 400 MHz) spectrum of **P28**.

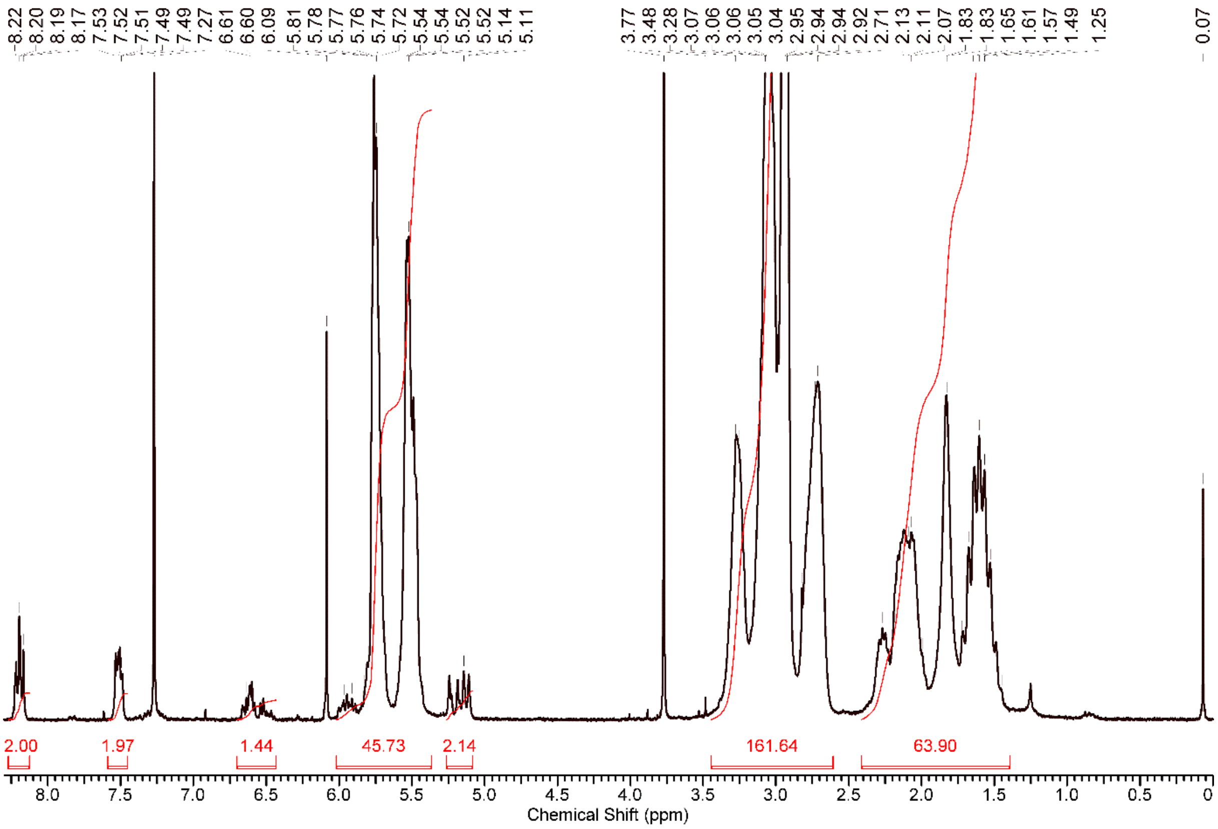

Fig. S76: ^{1}H NMR ($CDCl_3$, 400 MHz) spectrum of **P29**.

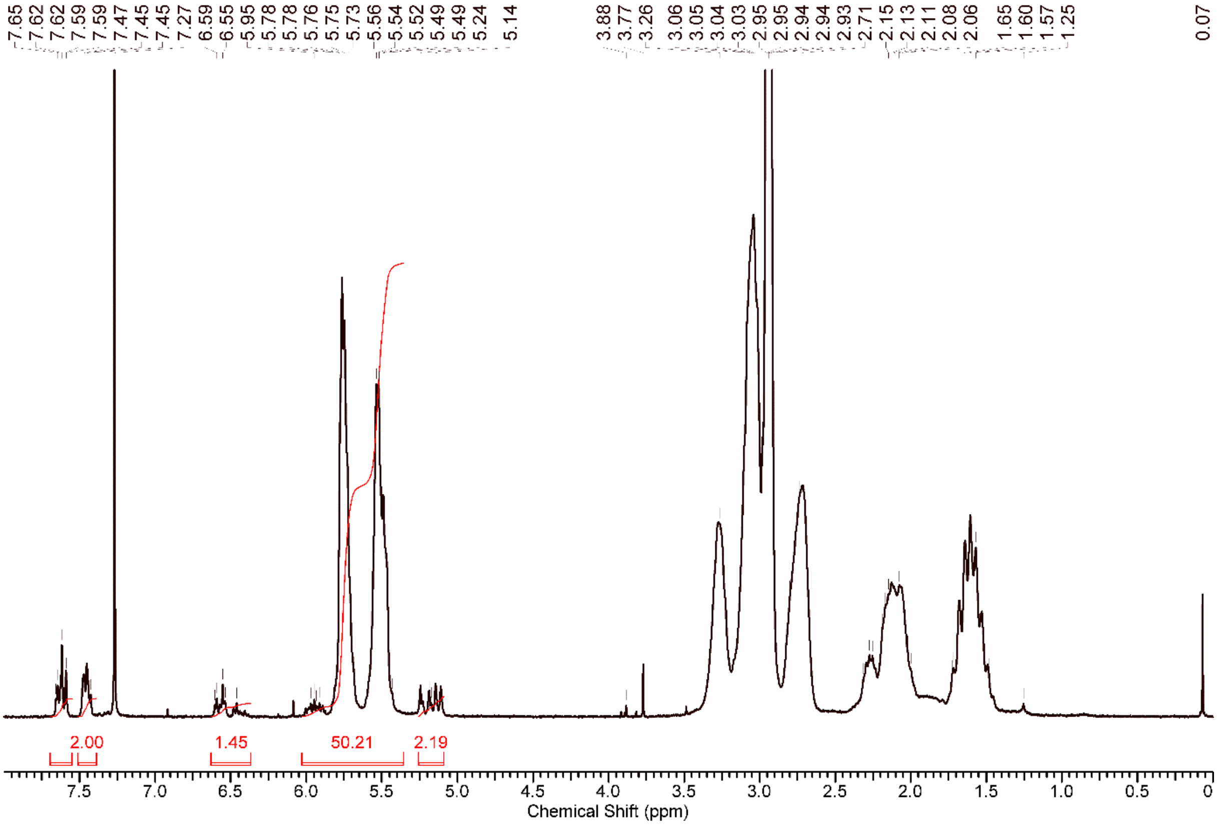

Fig. S77: ^{1}H NMR ($CDCl_3$, 400 MHz) spectrum of **P30**.

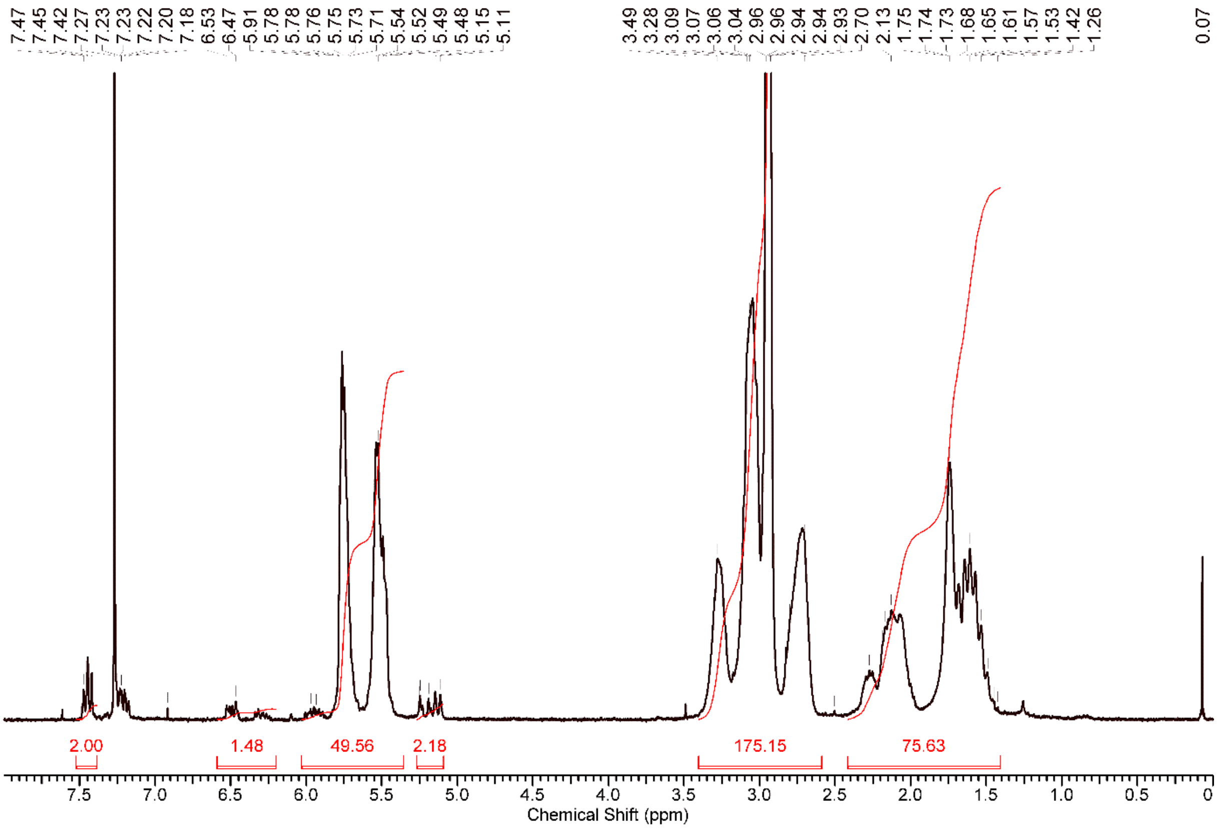

Fig. S78: ^{1}H NMR ($CDCl_3$, 400 MHz) spectrum of **P31**.

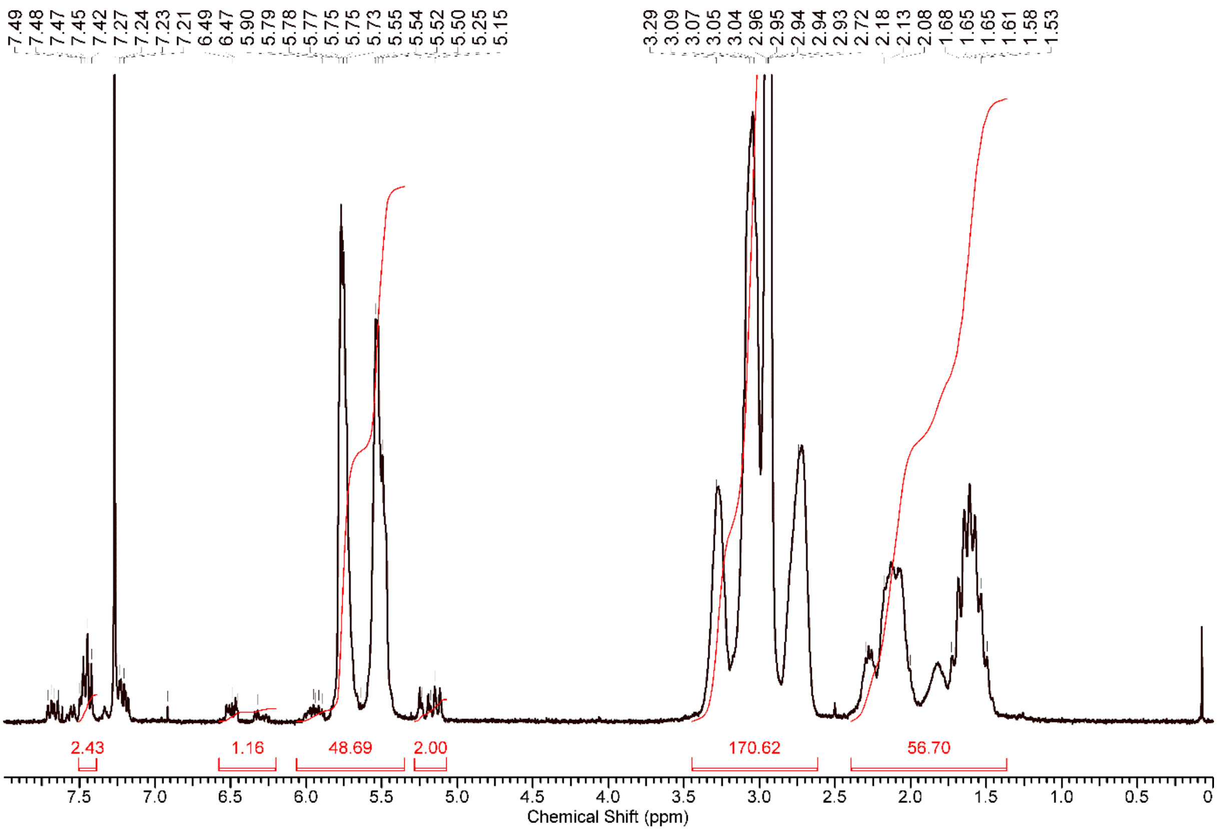

Fig. S79: ^{1}H NMR ($CDCl_3$, 400 MHz) spectrum of **P32**.

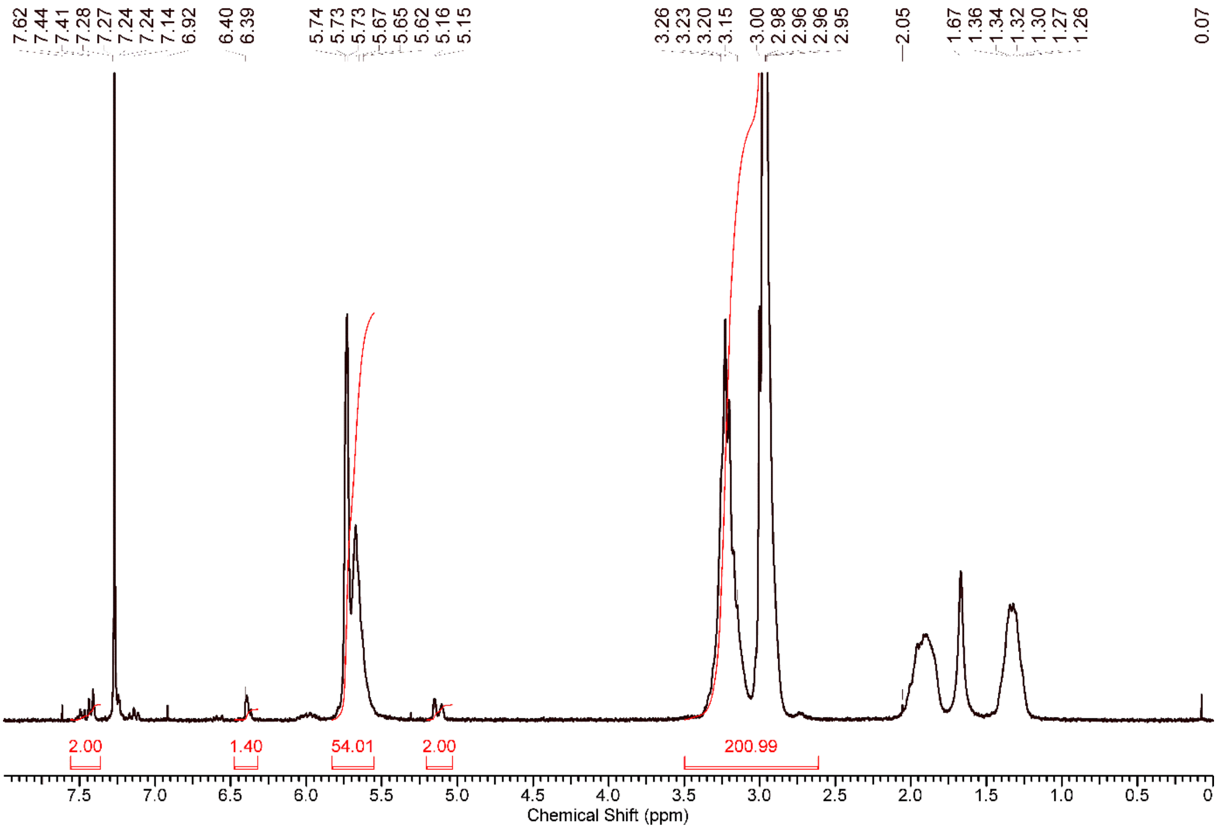

Fig. S80: ^{1}H NMR ($CDCl_3$, 400 MHz) spectrum of **P33**.

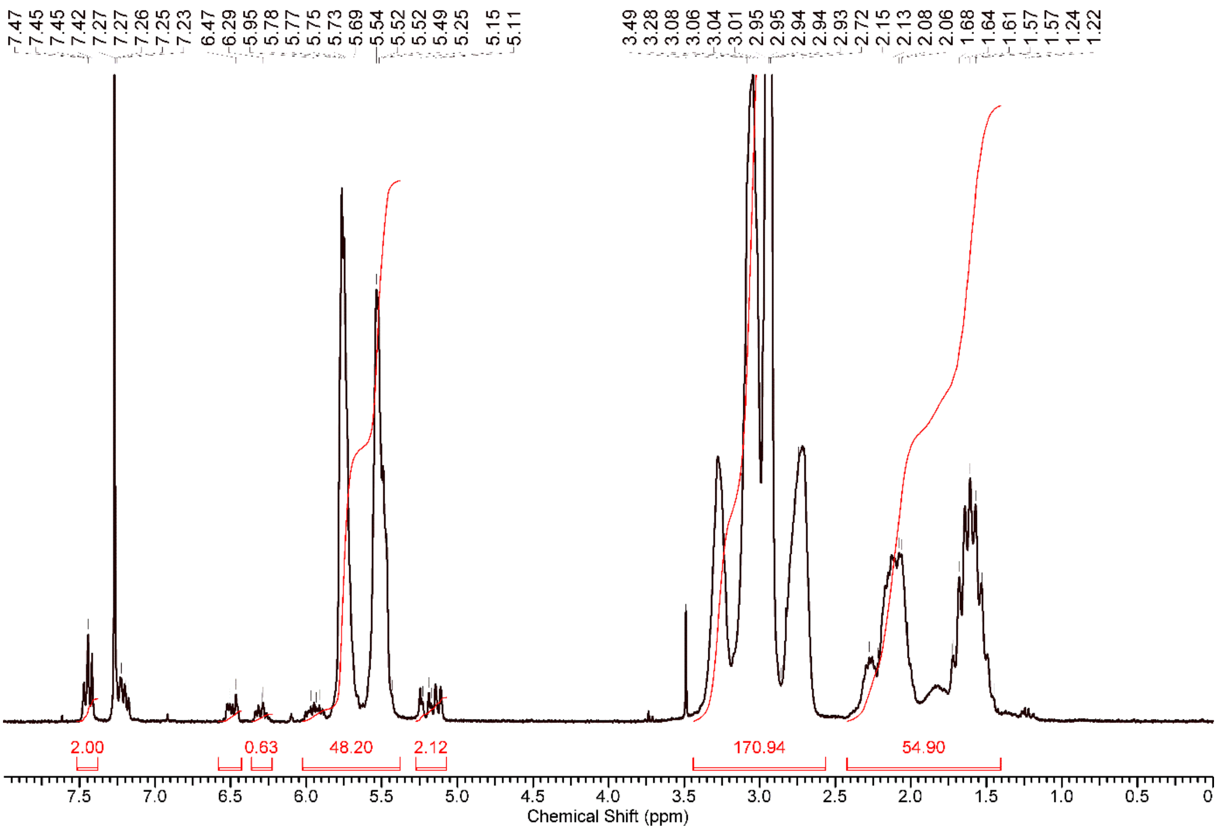

Fig. S81: ^{1}H NMR ($CDCl_3$, 400 MHz) spectrum of **P34**.

MALDI-ToF mass spectra:

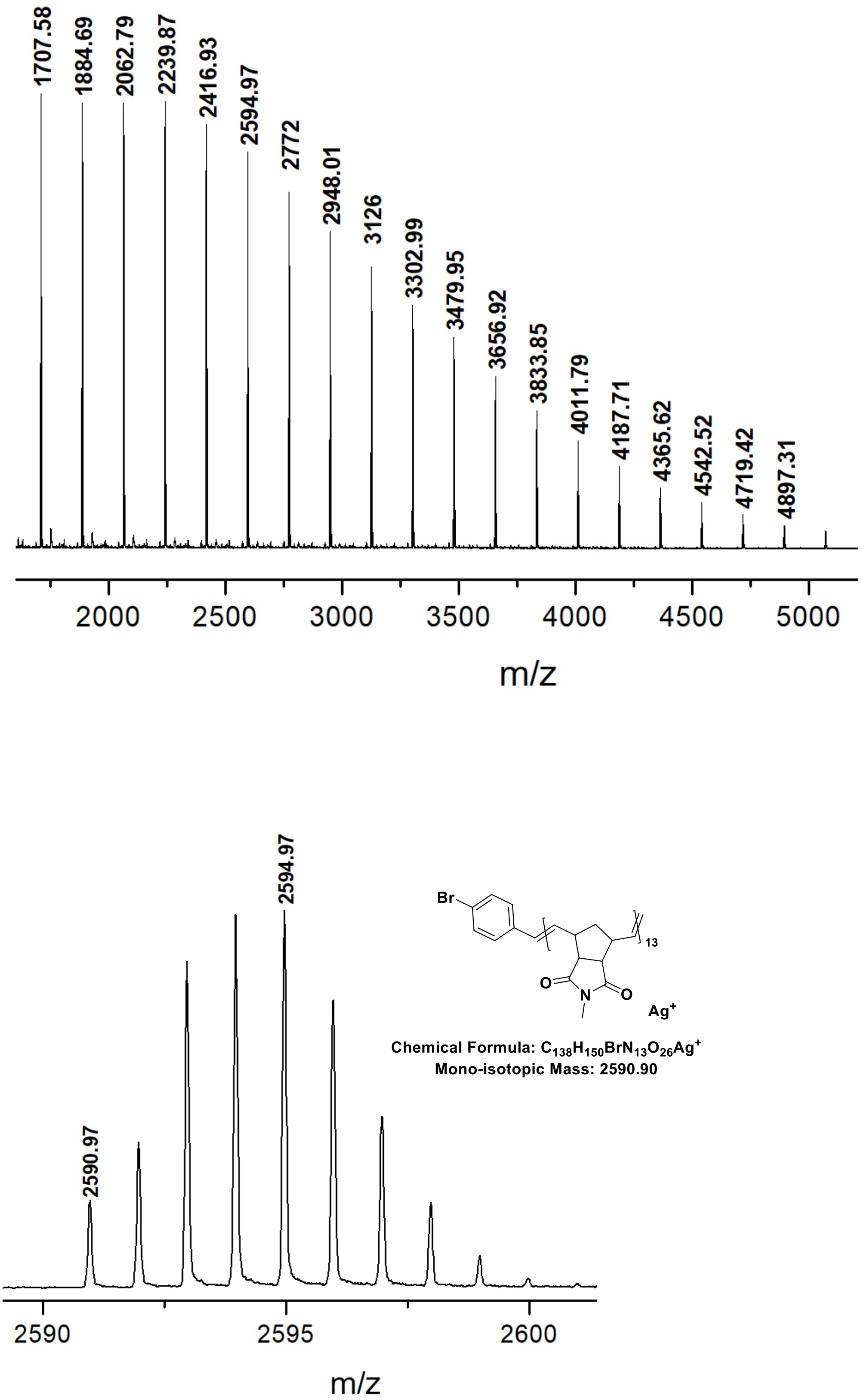

Fig. S82: MALDI-ToF (DCTB, AgTFA) mass spectrum of **P4**.

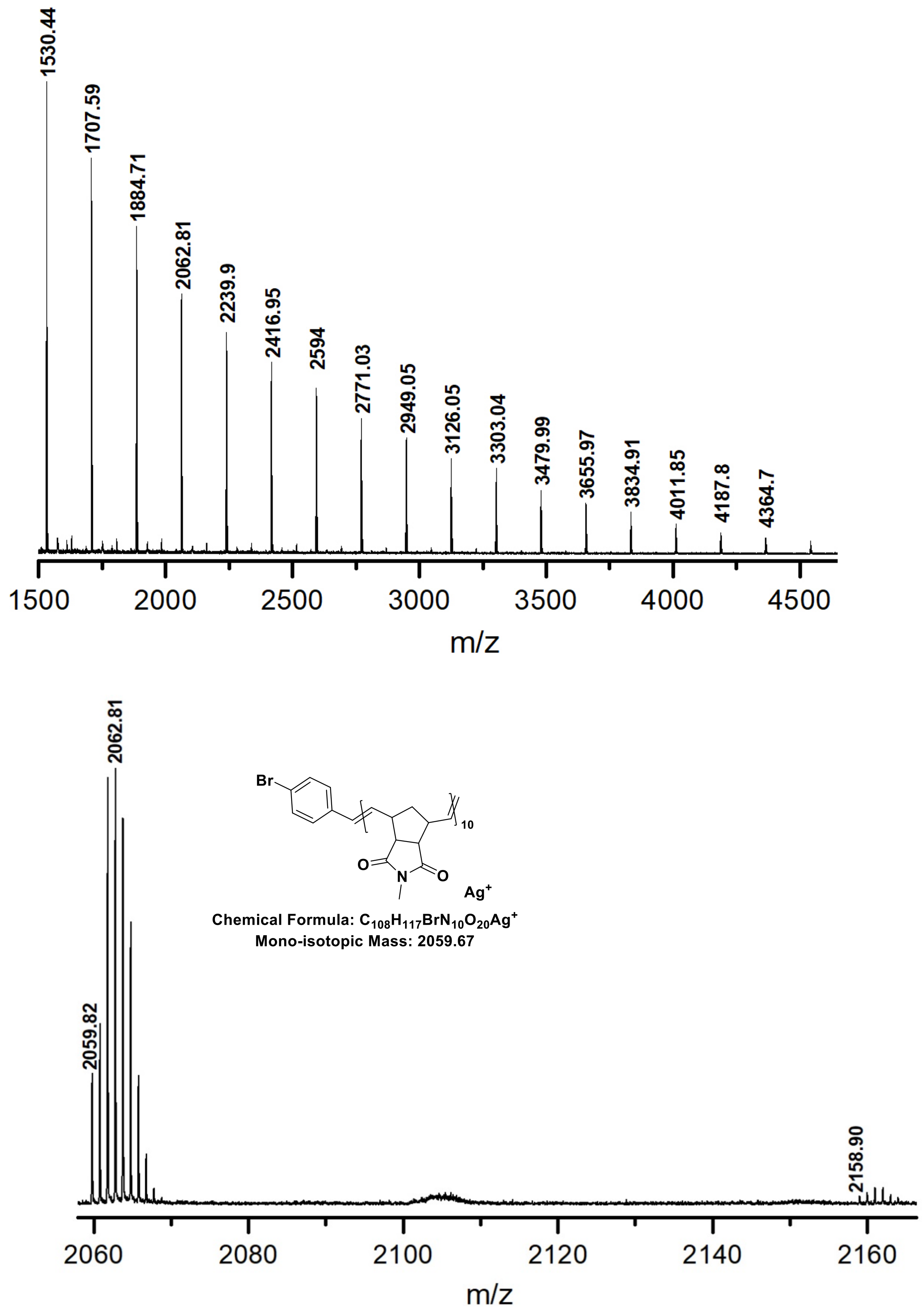

Fig. S83: MALDI-ToF (DCTB, AgTFA) mass spectrum of **P8**.

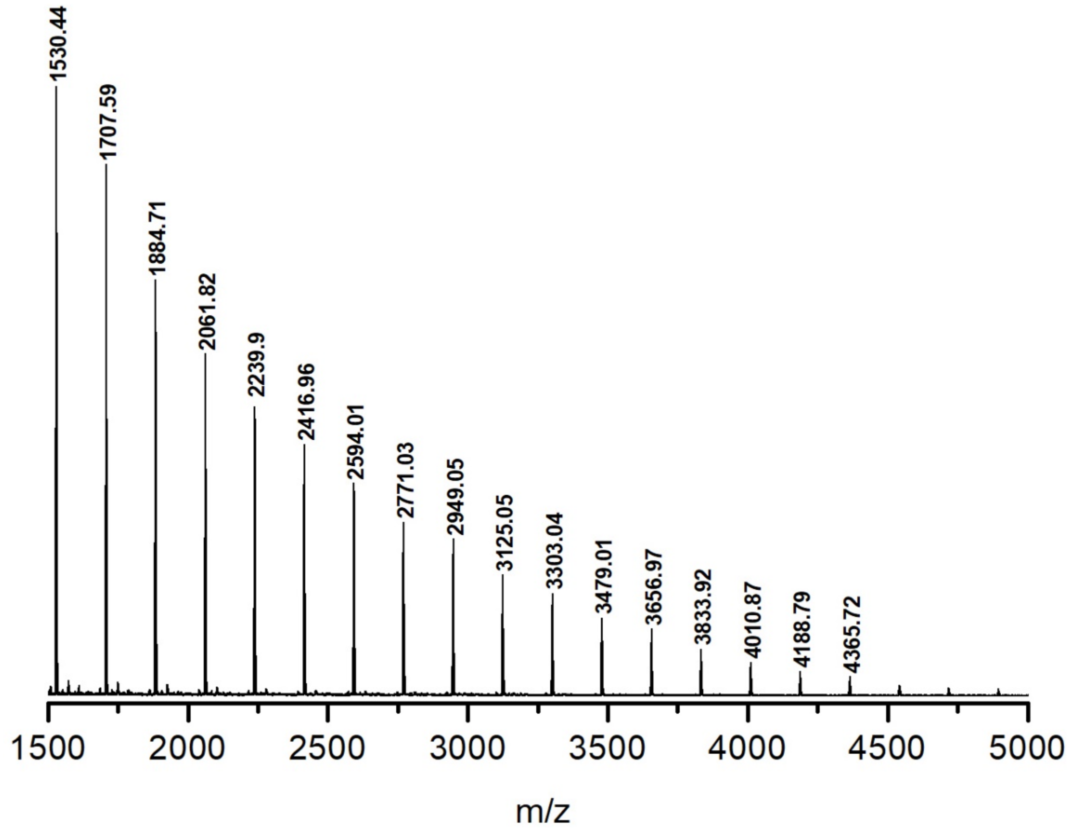

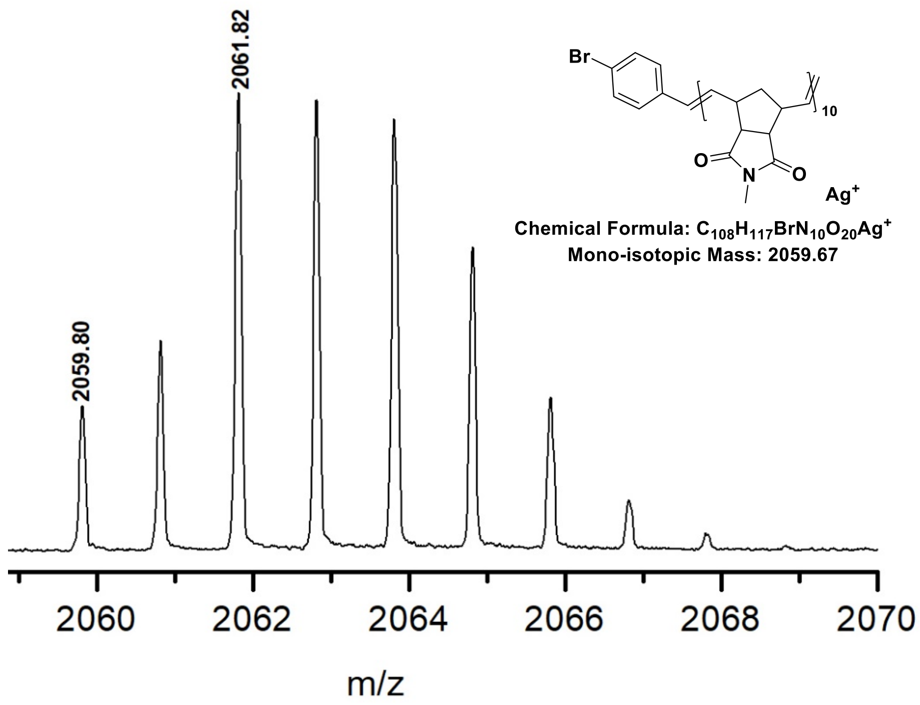

Fig. S84: MALDI-ToF (DCTB, AgTFA) mass spectrum of **P9**.

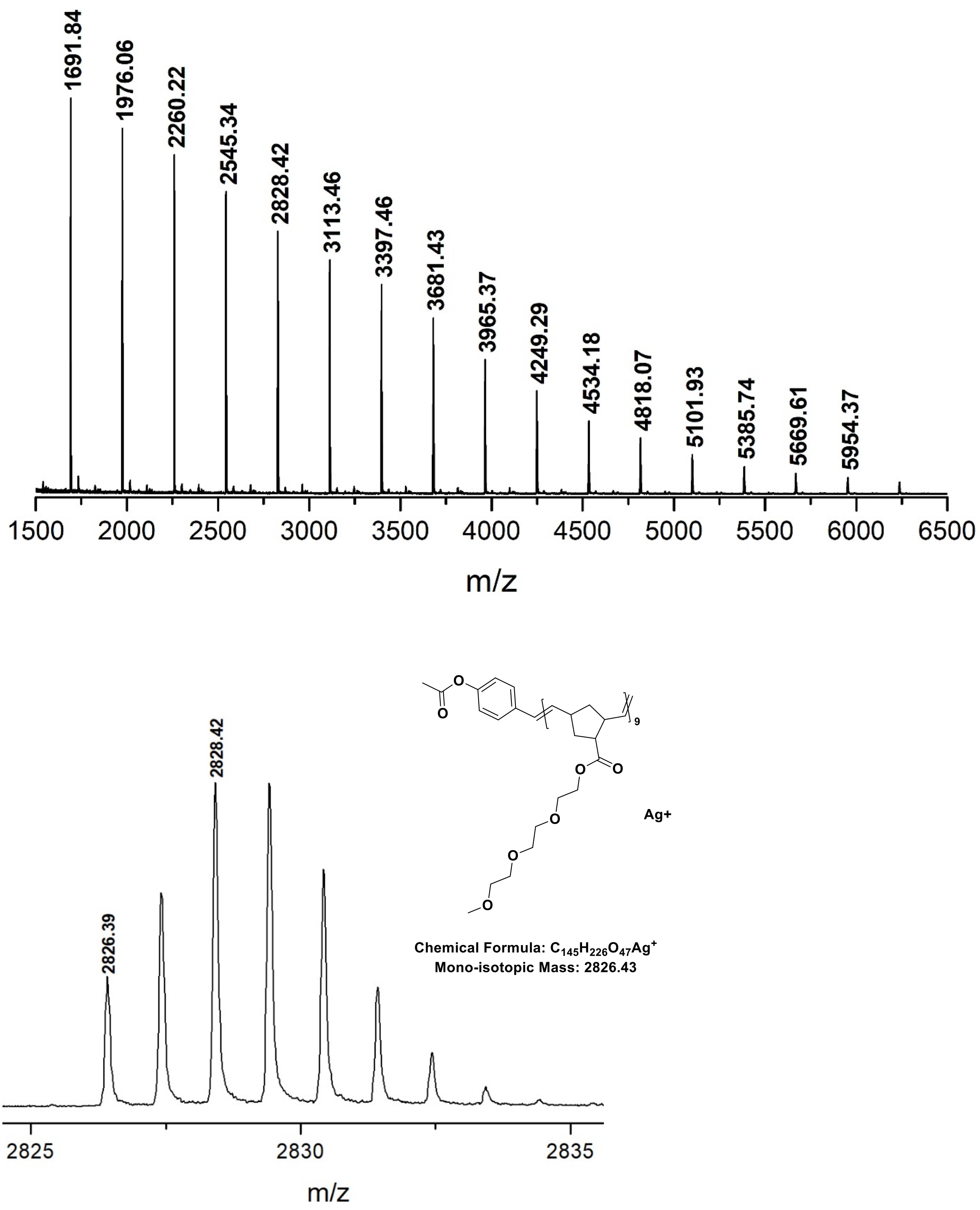

Fig. S85: MALDI-ToF (DCTB, AgTFA) mass spectrum of **P11**.

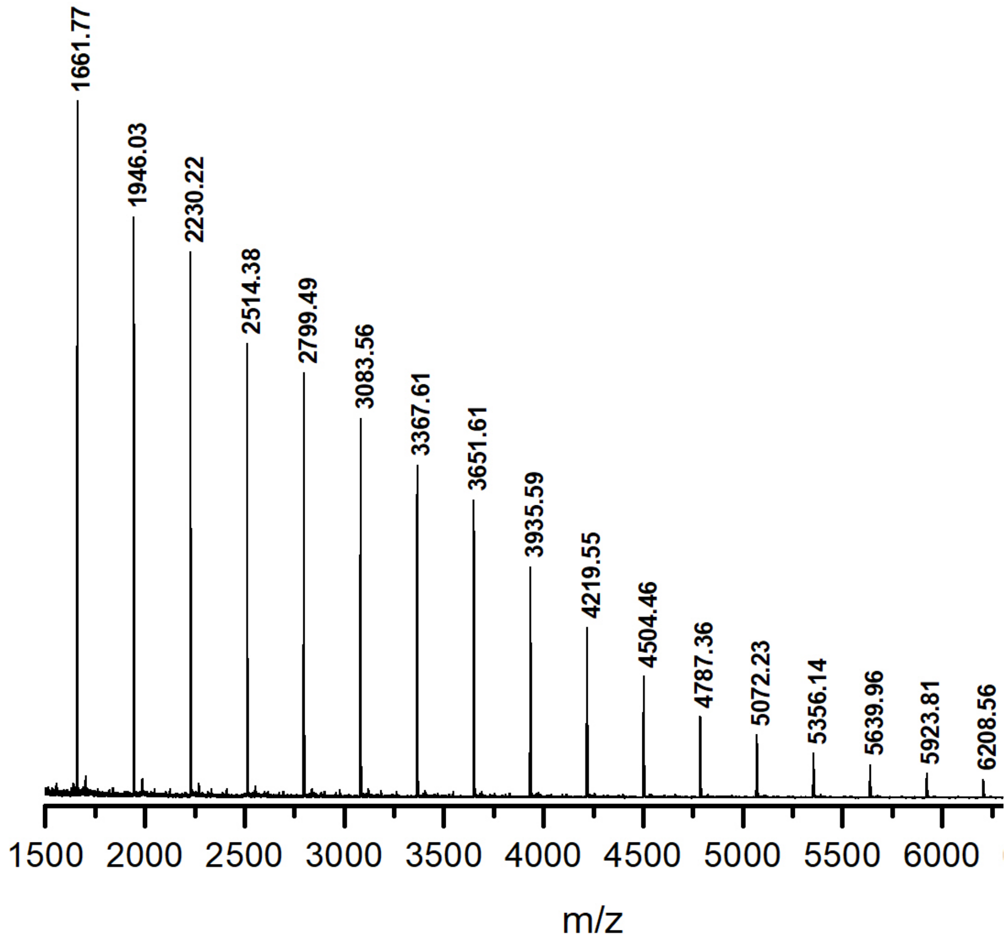

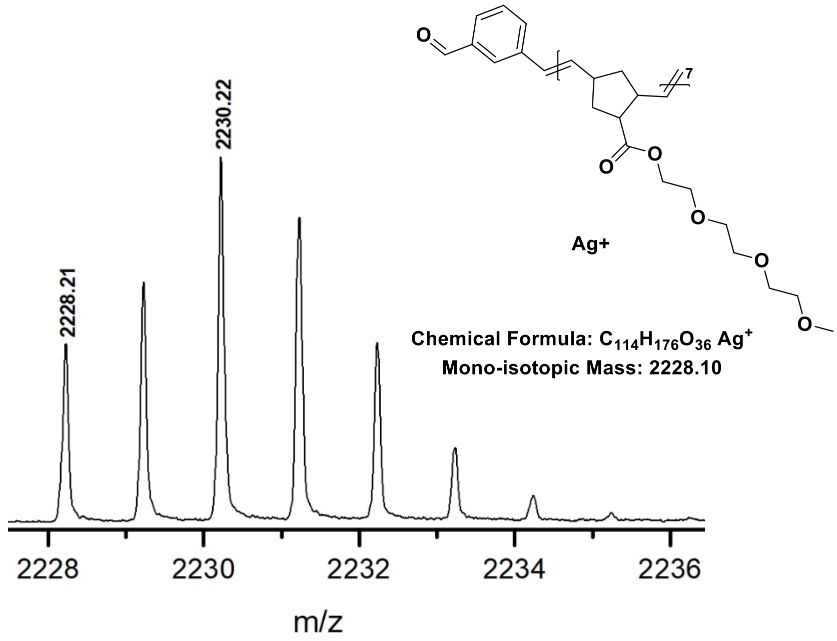

Fig. S86: MALDI-ToF (DCTB, AgTFA) mass spectrum of **P12**.

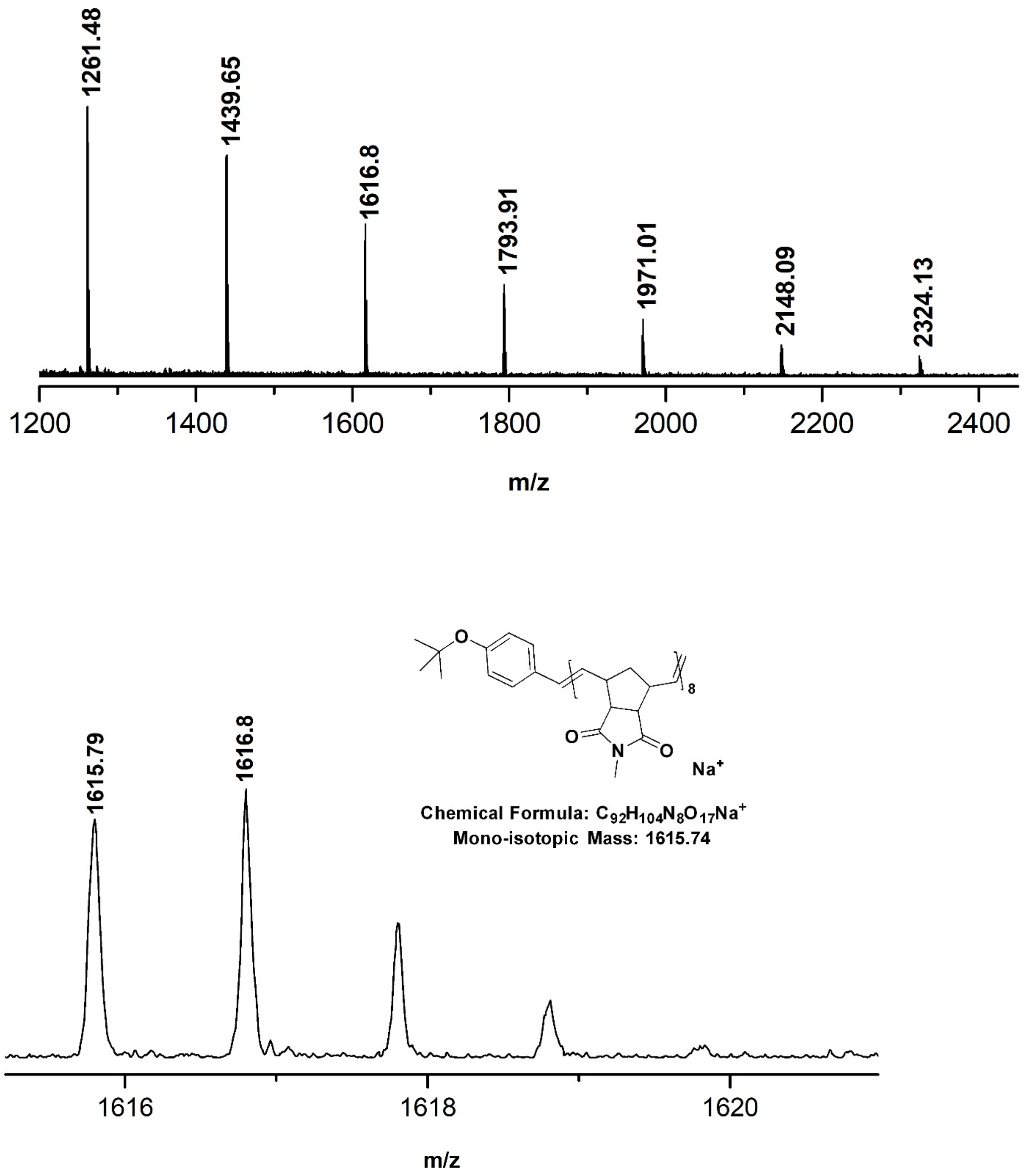

Fig. S87: MALDI-ToF (DCTB, NaTFA) mass spectrum of **P13**.

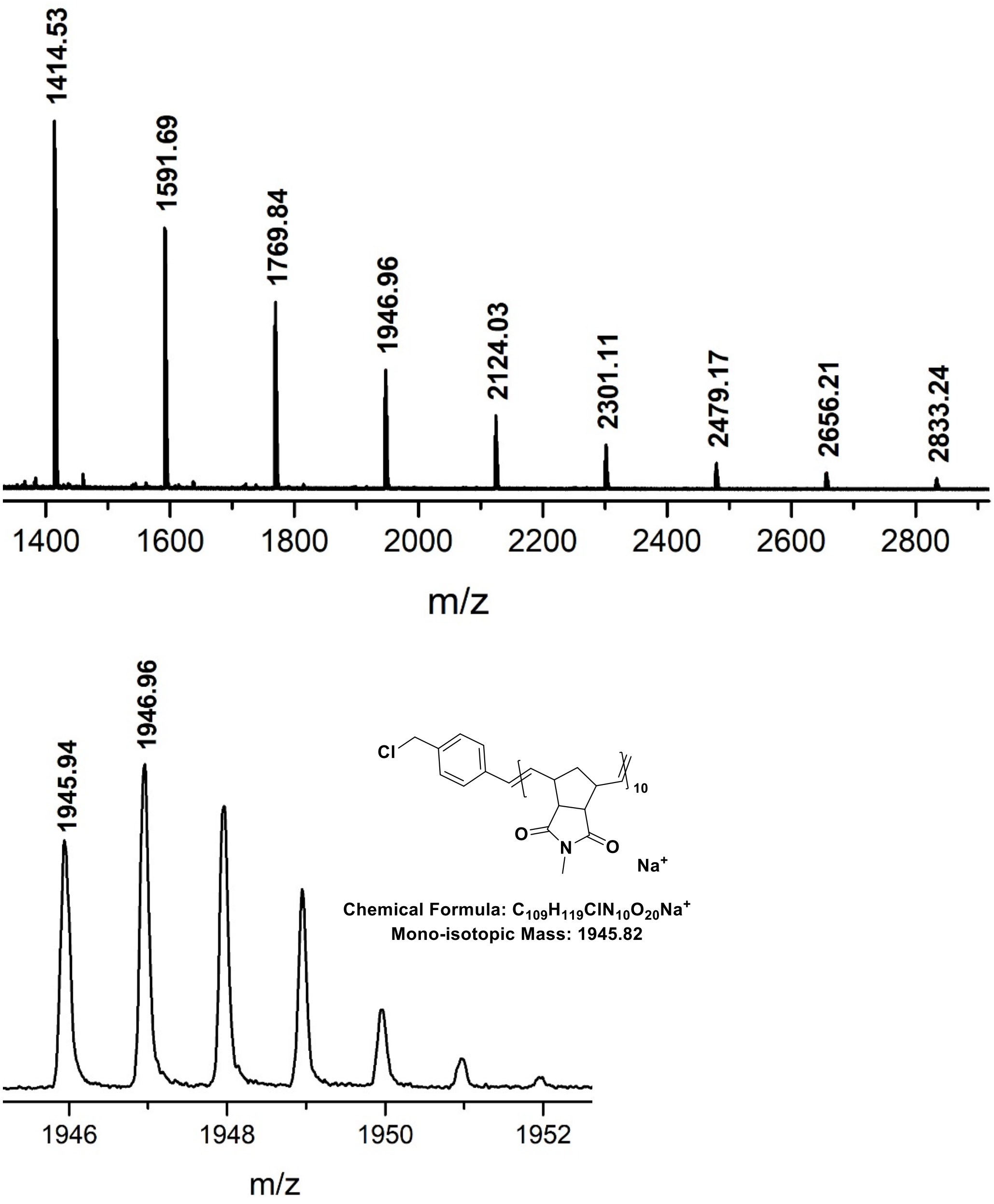

Fig. S88: MALDI-ToF (DCTB, NaTFA) mass spectrum of **P15**.

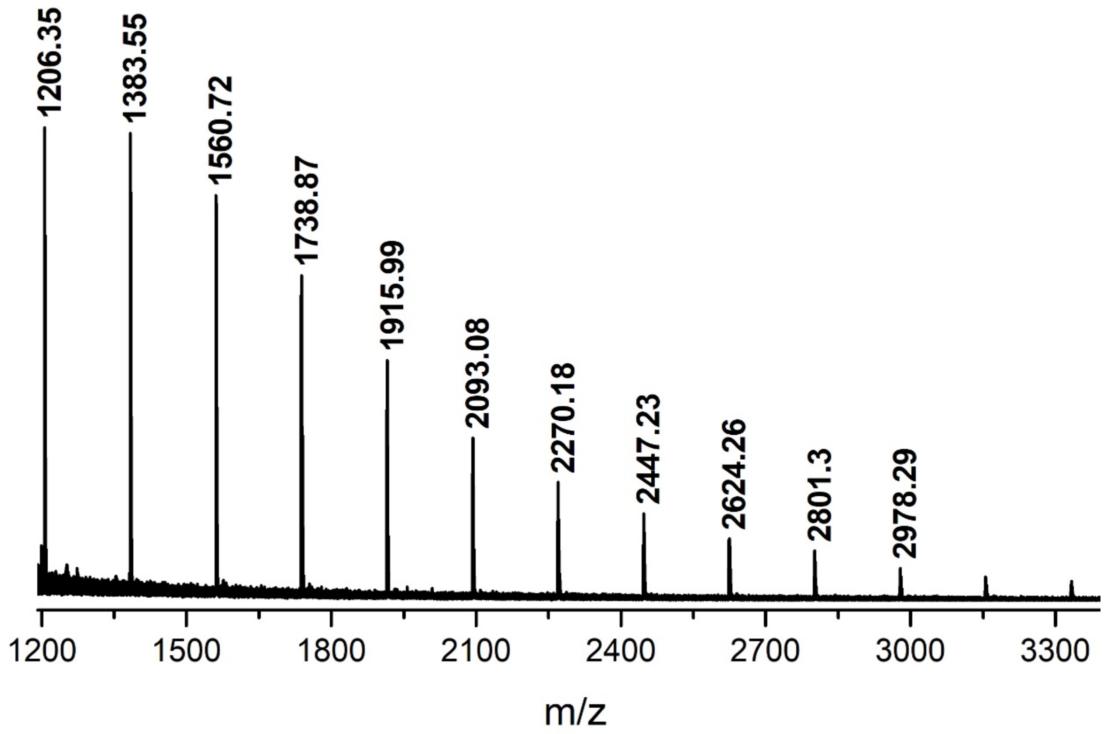

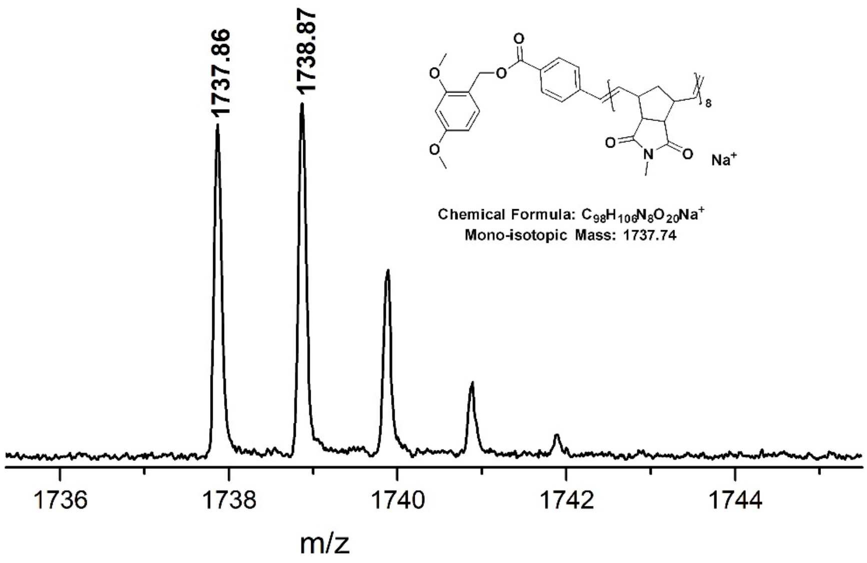

Fig. S89: MALDI-ToF (DCTB, NaTFA) mass spectrum of **P18**.

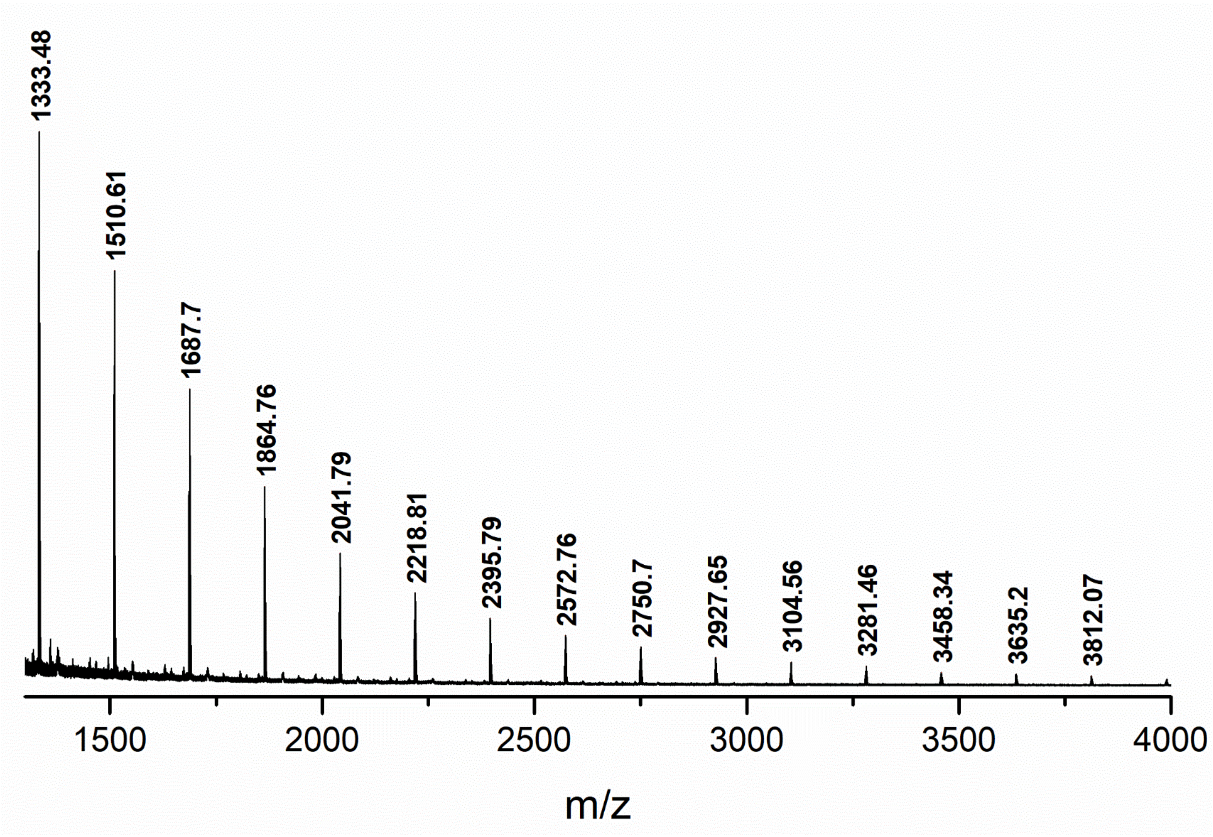

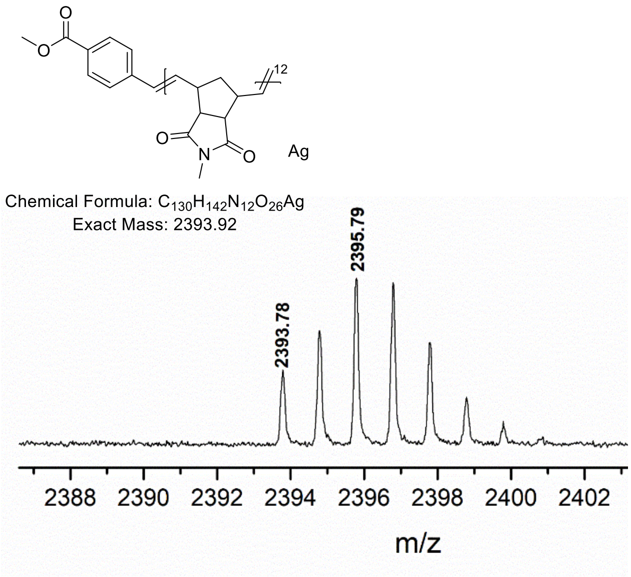

Fig. S90: MALDI-ToF (DCTB, AgTFA) mass spectrum of **P19**.

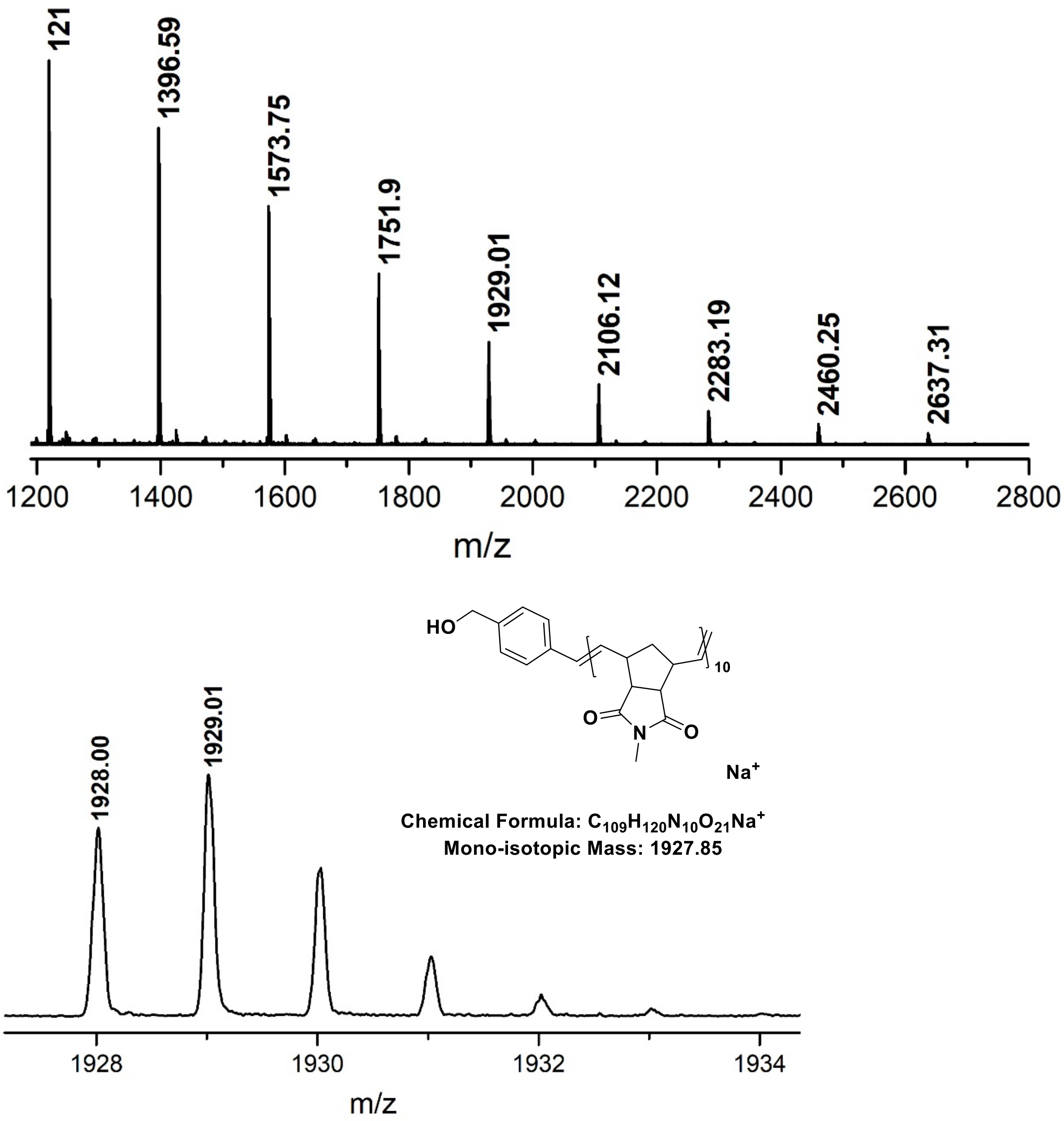

Fig. S91: MALDI-ToF (DCTB, NaTFA) mass spectrum of **P20**.

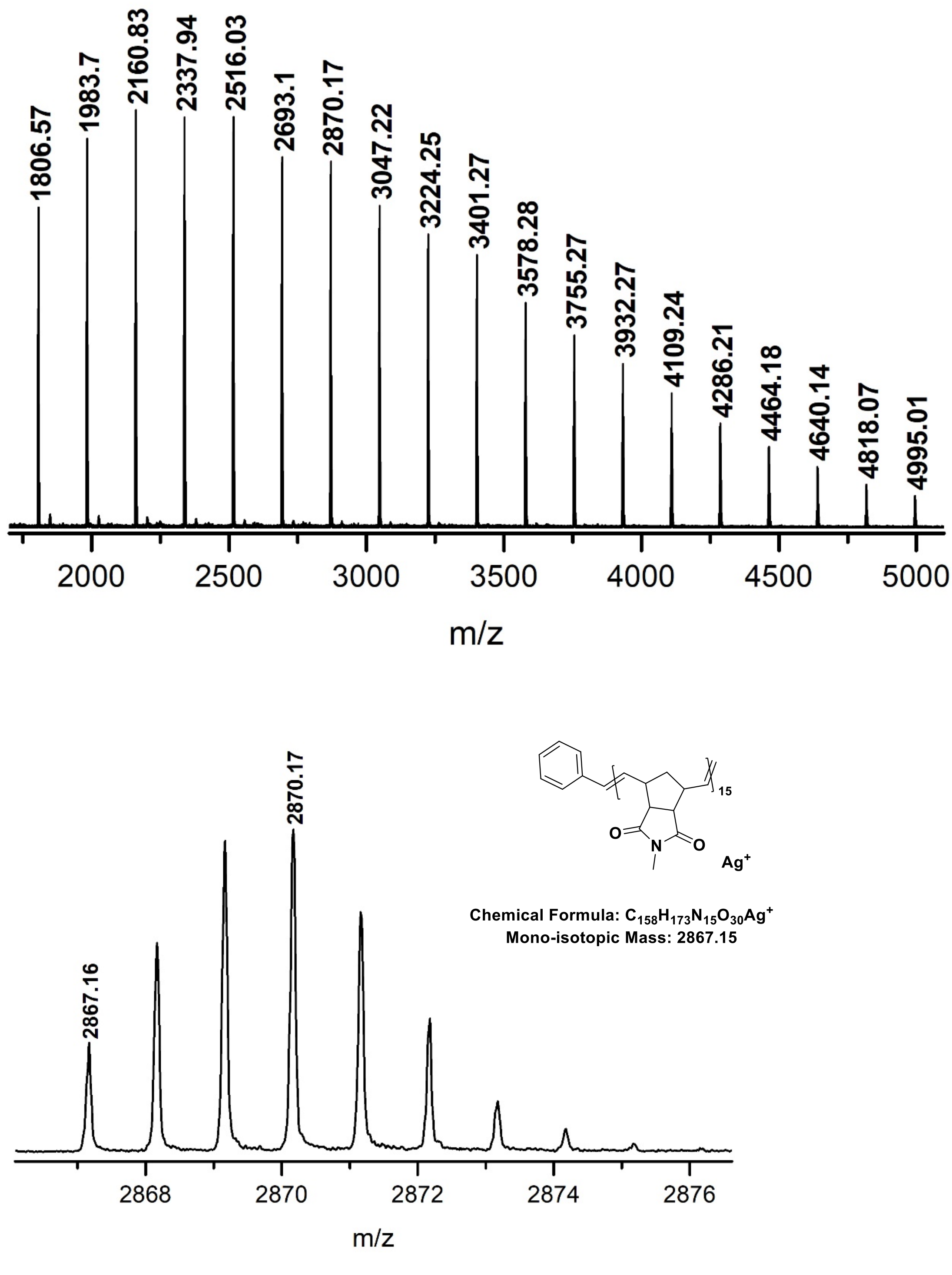

Fig. S92: MALDI-ToF (DCTB, AgTFA) mass spectrum of **P23**.

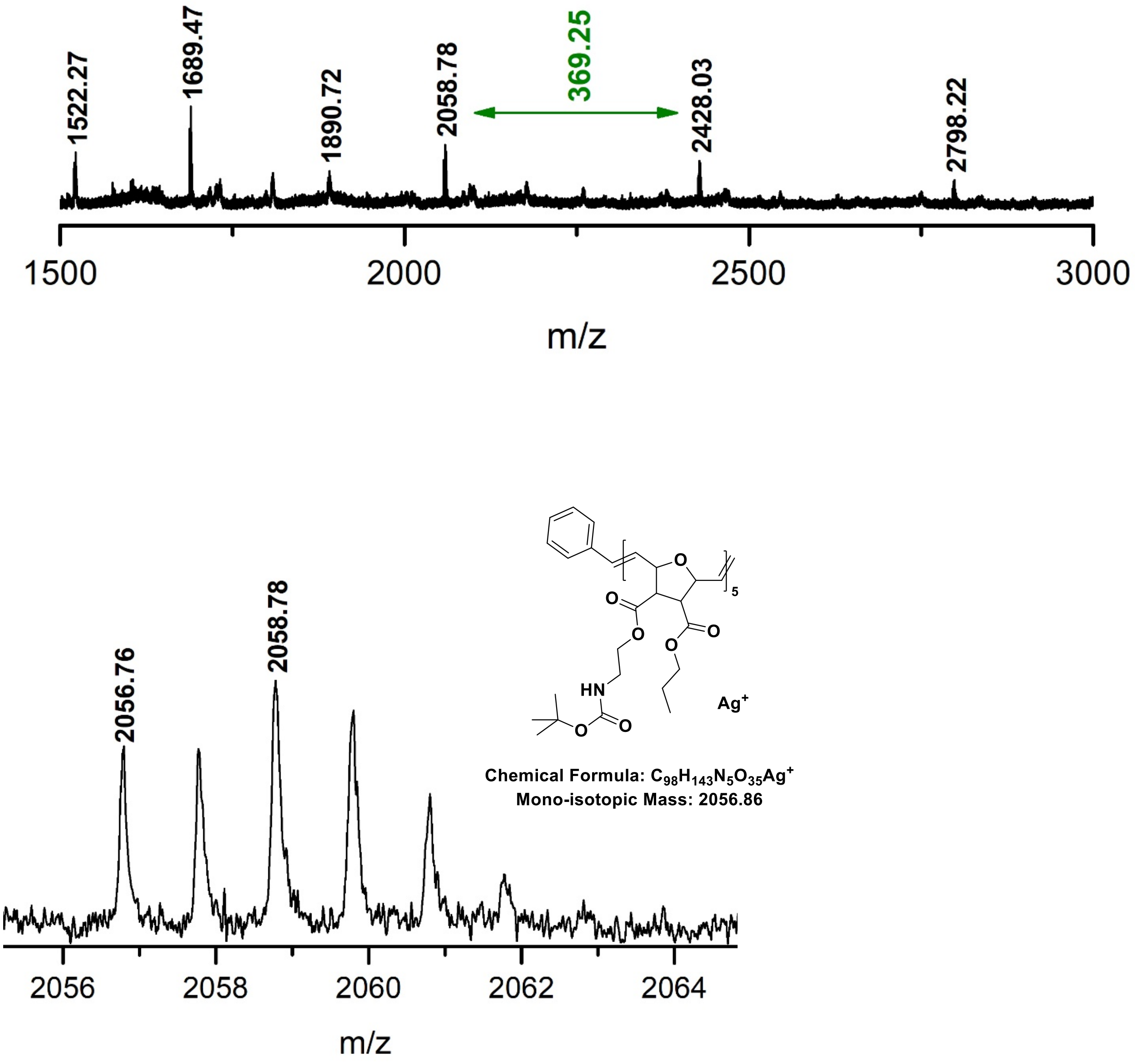

Fig. S93: MALDI-ToF (DCTB, AgTFA) mass spectrum of **P27**.

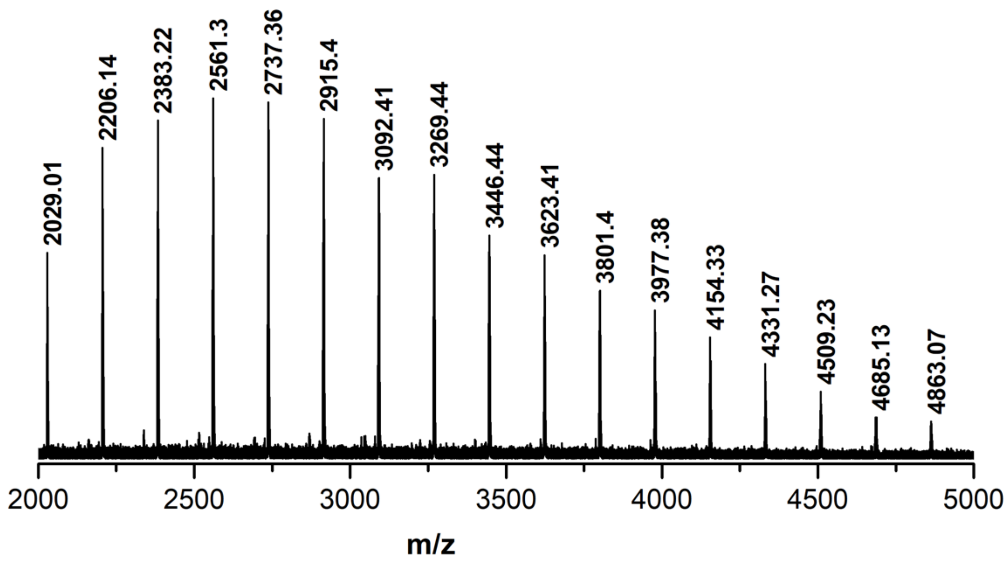

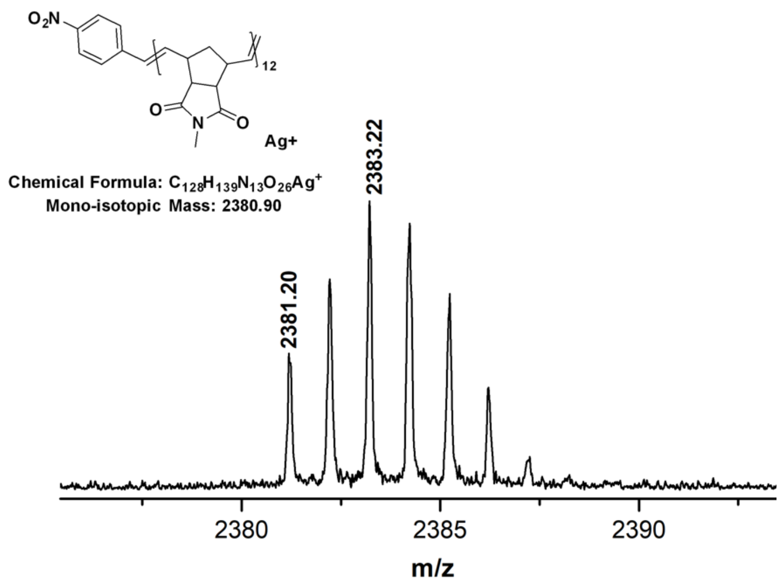

Fig. S94: MALDI-ToF (DCTB, AgTFA) mass spectrum of **P29**.

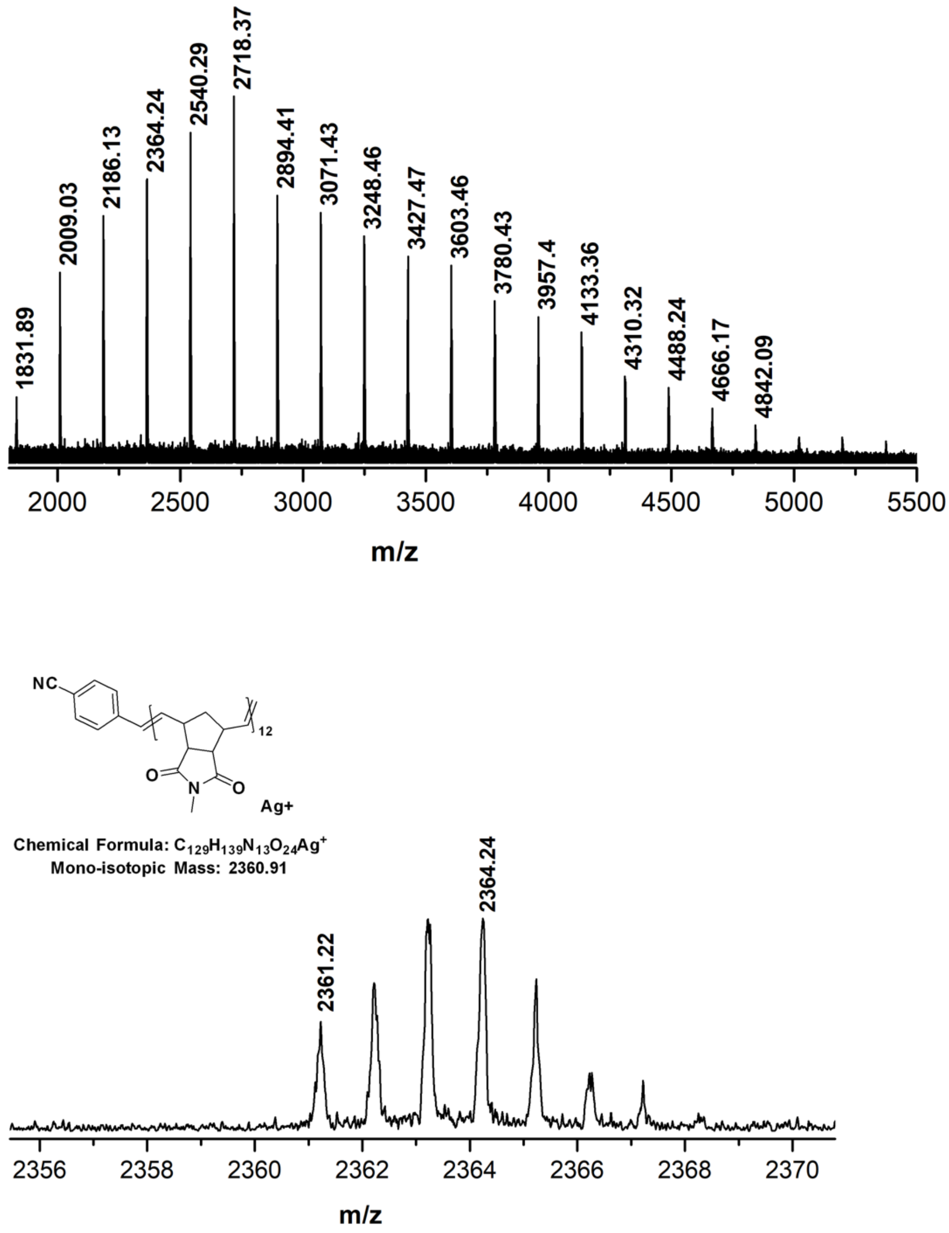

Fig. S95: MALDI-ToF (DCTB, AgTFA) mass spectrum of **P30**.

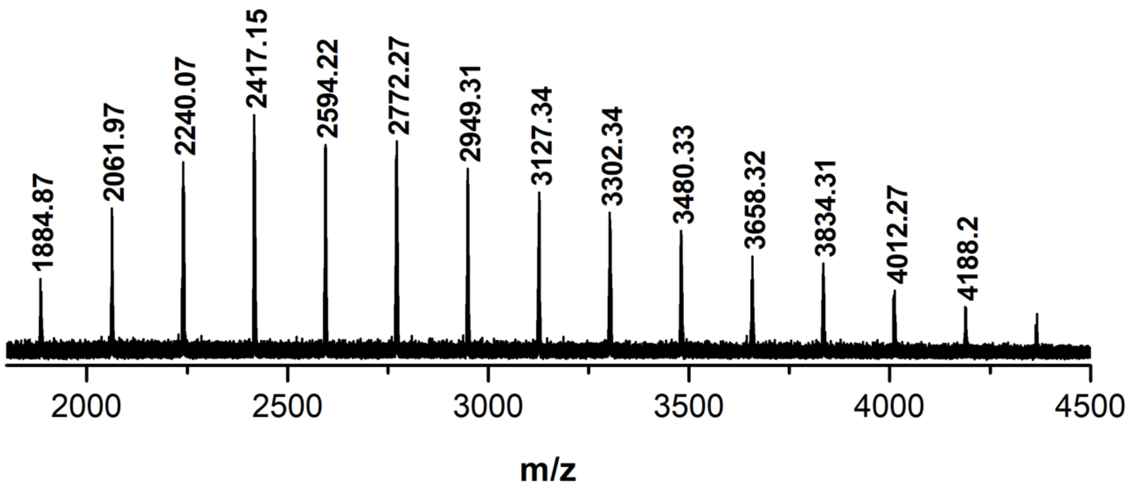

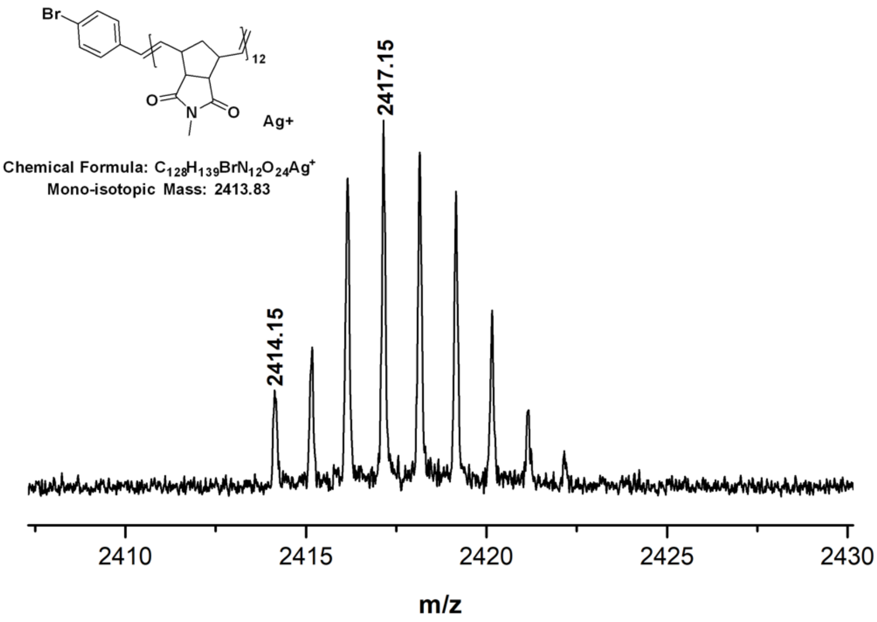

Fig. S96: MALDI-ToF (DCTB, AgTFA) mass spectrum of **P34**.

SEC data of polymers:

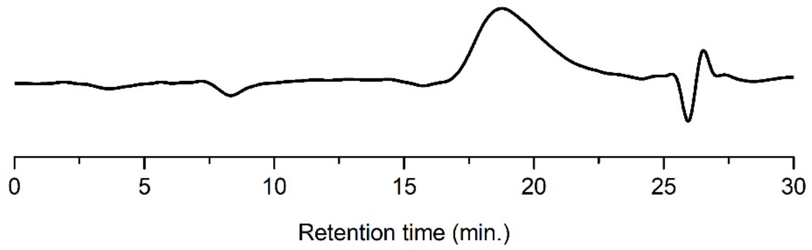

Fig. S97: SEC ($CHCl_3$) trace of **P1**.

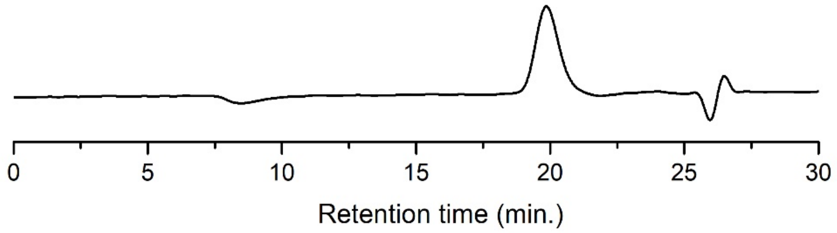

Fig. S98: SEC ($CHCl_3$) trace of **P2**.

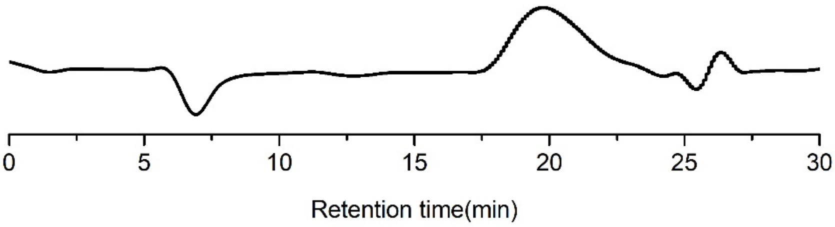

Fig. S99: SEC ($CHCl_3$) trace of **P3**.

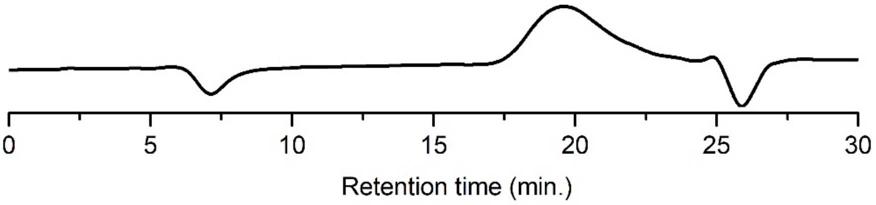

Fig. S100: SEC ($CHCl_3$) trace of **P4**.

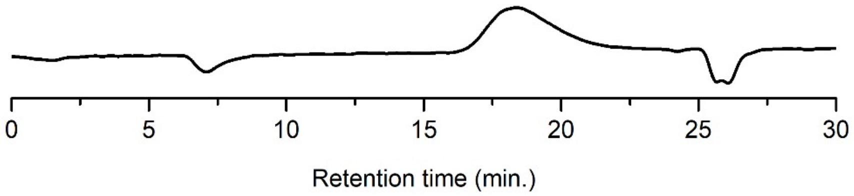

Fig. S101: SEC ($CHCl_3$) trace of **P5**.

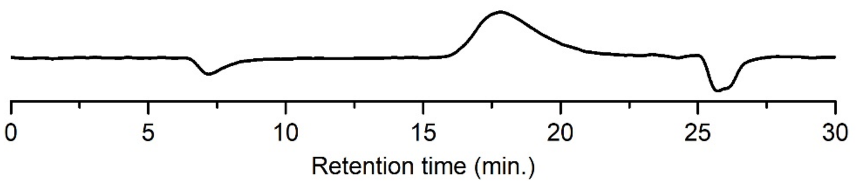

Fig. S102: SEC ($CHCl_3$) trace of **P6**.

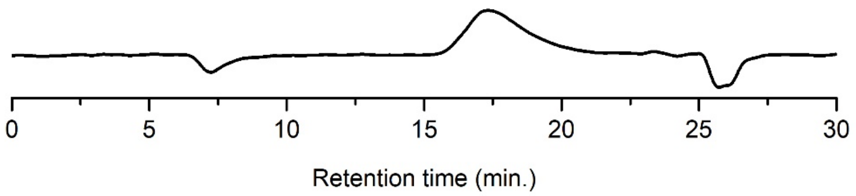

Fig. S103: SEC ($CHCl_3$) trace of **P7**.

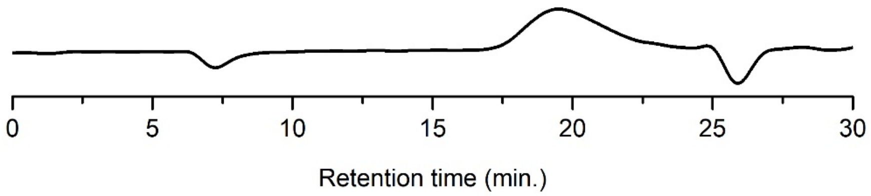

Fig. S104: SEC ($CHCl_3$) trace of **P8**.

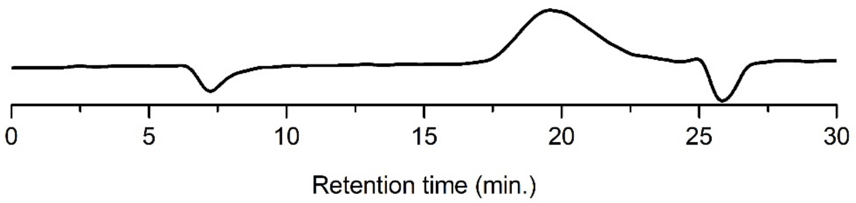

Fig. S105: SEC ($CHCl_3$) trace of **P9**.

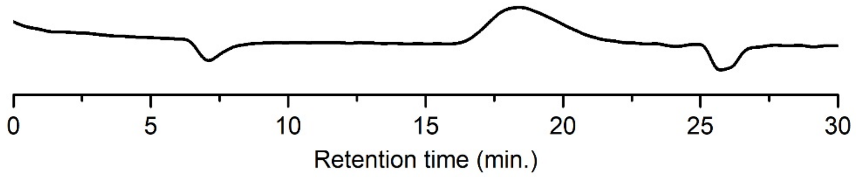

Fig. S106: SEC ($CHCl_3$) trace of **P10**.

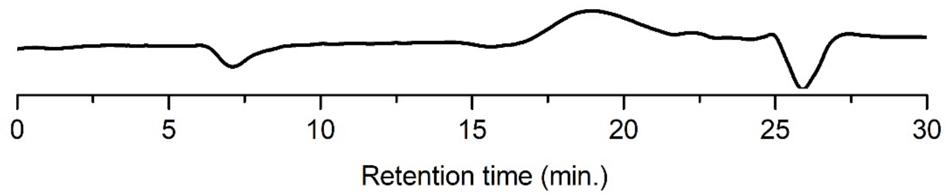

Fig. S107: SEC ($CHCl_3$) trace of **P11**.

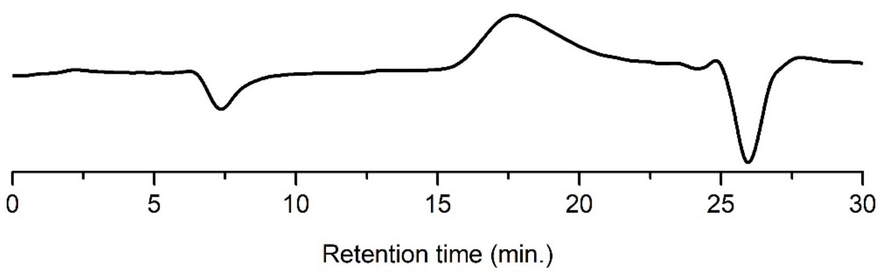

Fig. S108: SEC ($CHCl_3$) trace of **P12**.

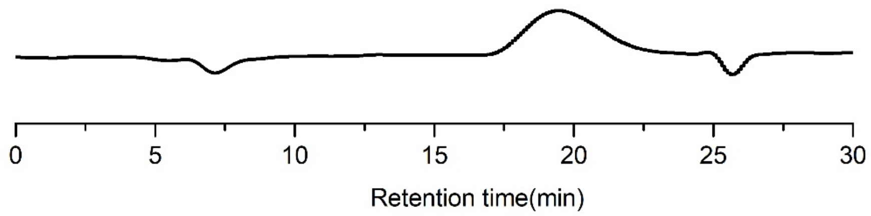

Fig. S109: SEC ($CHCl_3$) trace of **P13**.

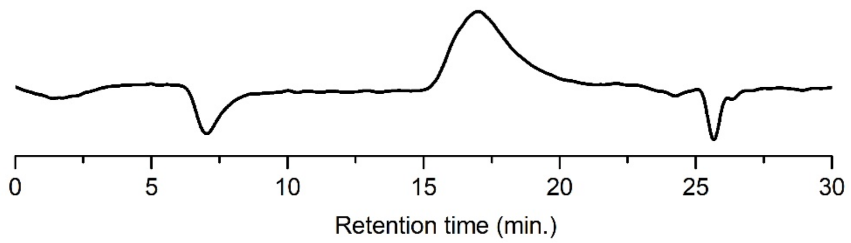

Fig. S110: SEC ($CHCl_3$) trace of **P14**.

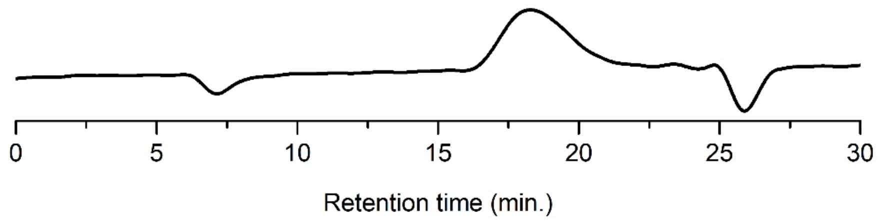

Fig. S111: SEC ($CHCl_3$) trace of **P15**.

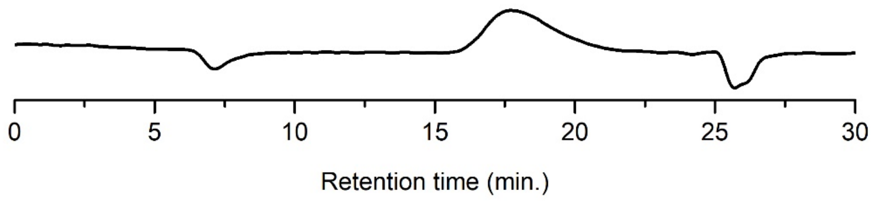

Fig. S112: SEC ($CHCl_3$) trace of **P16**.

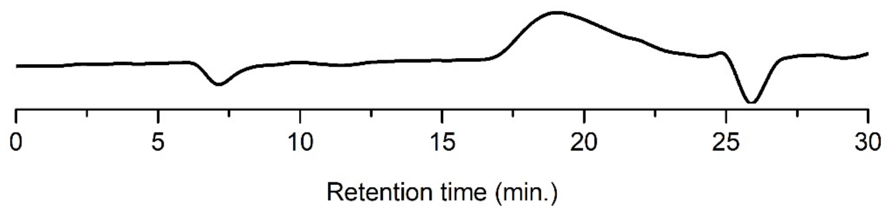

Fig. S113: SEC ($CHCl_3$) trace of **P17**.

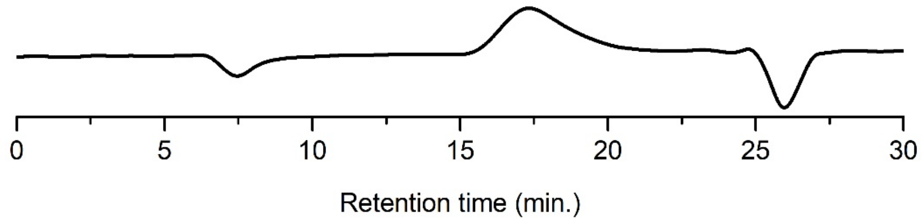

Fig. S114: SEC ($CHCl_3$) trace of **P18**.

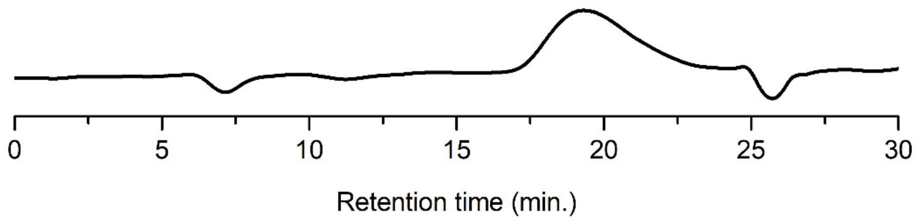

Fig. S115: SEC ($CHCl_3$) trace of **P19**.

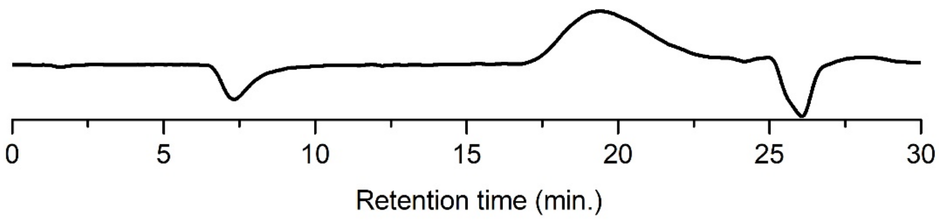

Fig. S116: SEC ($CHCl_3$) trace of **P20**.

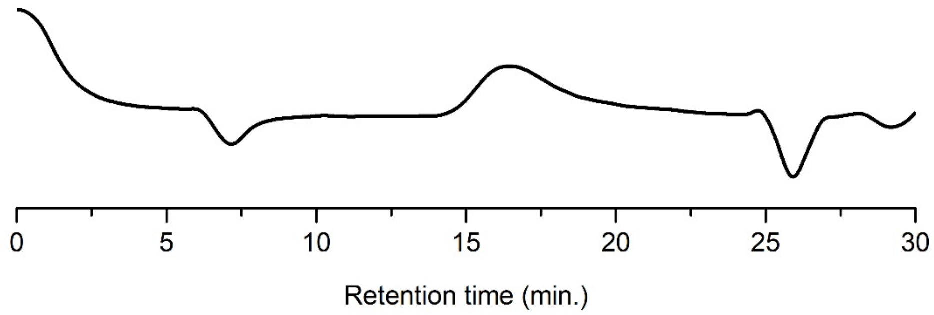

Fig. S117: SEC ($CHCl_3$) trace of **P21**.

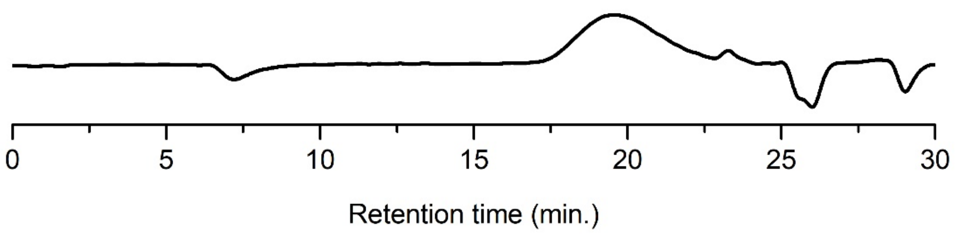

Fig. S118: SEC ($CHCl_3$) trace of **P22**.

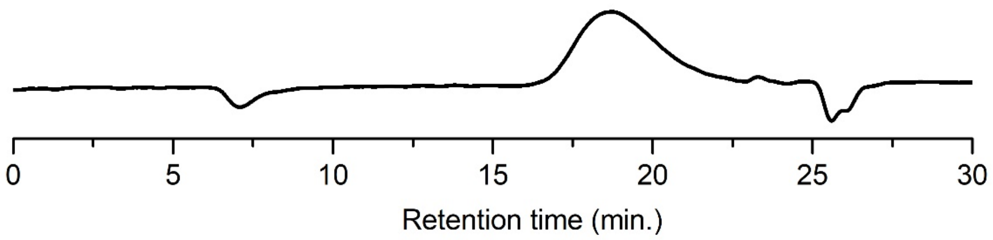

Fig. S119: SEC ($CHCl_3$) trace of **P23**.

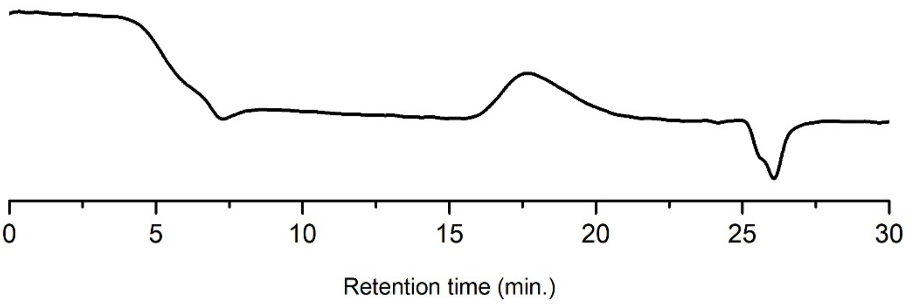

Fig. S120: SEC ($CHCl_3$) trace of **P24**.

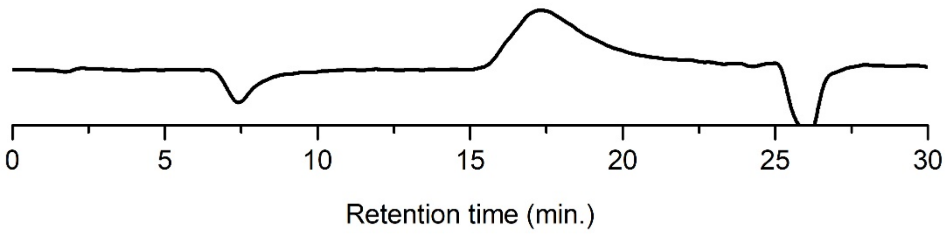

Fig. S121: SEC ($CHCl_3$) trace of **P25**.

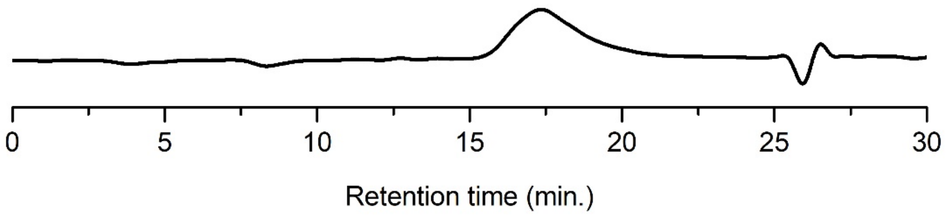

Fig. S122: SEC ($CHCl_3$) trace of **P26**.

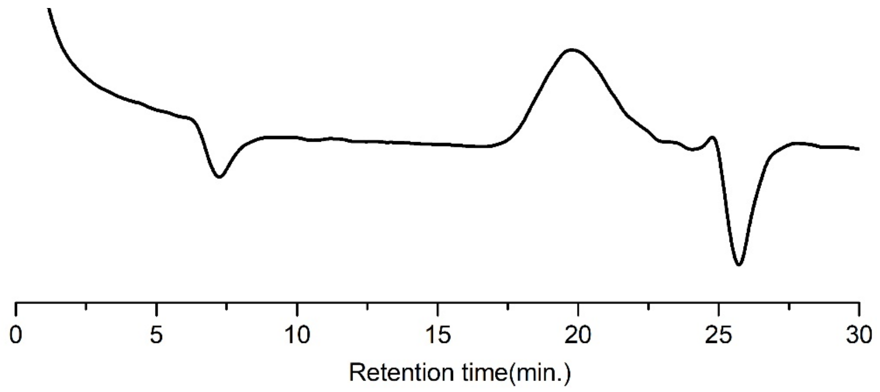

Fig. S123: SEC ($CHCl_3$) trace of **P27**.

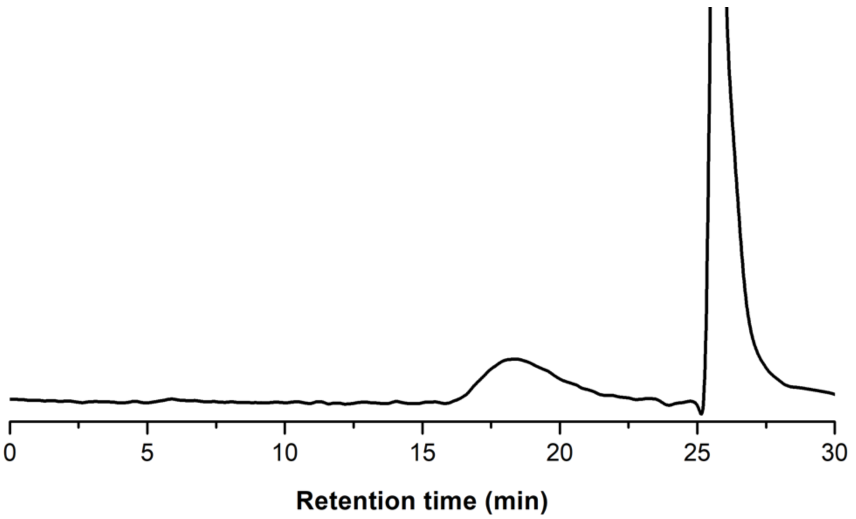

Fig. S124: SEC ($CHCl_3$) trace of **P29**.

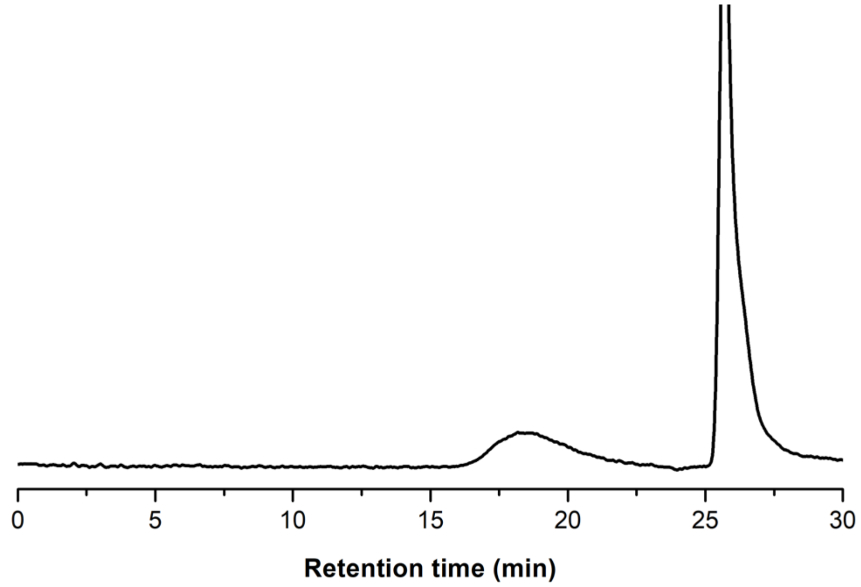

Fig. S125: SEC ($CHCl_3$) trace of **P30**.

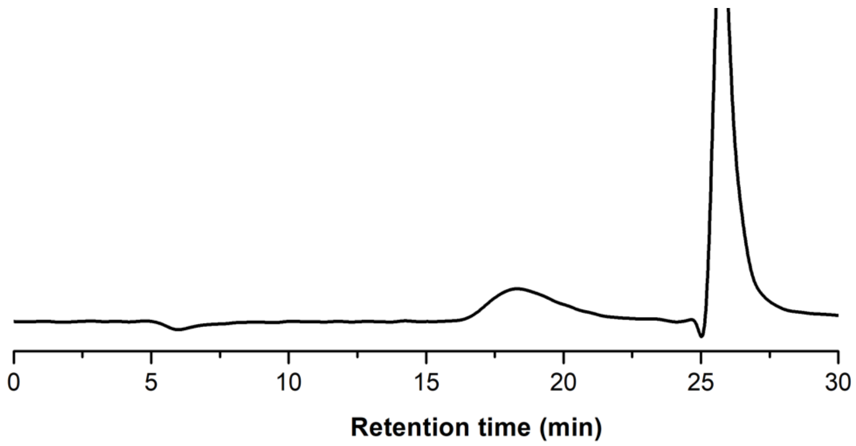

Fig. S126: SEC ($CHCl_3$) trace of **P31**.

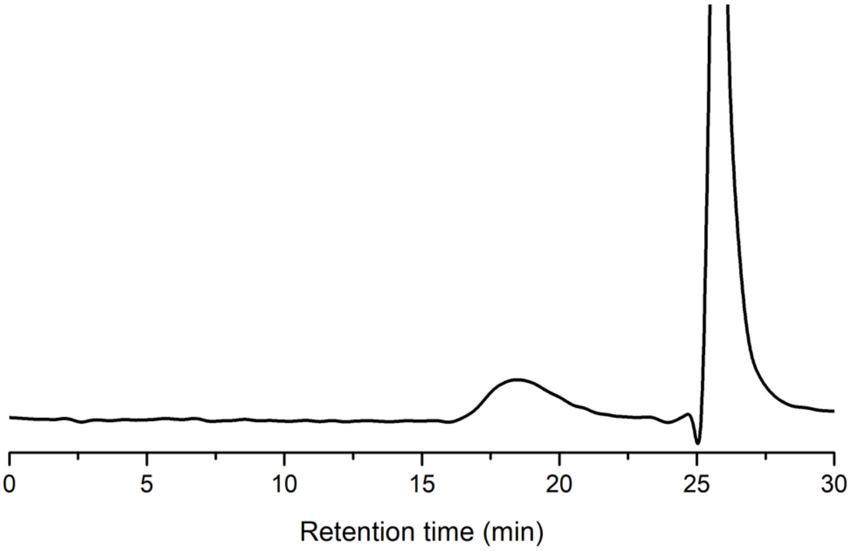

Fig. S127: SEC ($CHCl_3$) trace of **P32**.

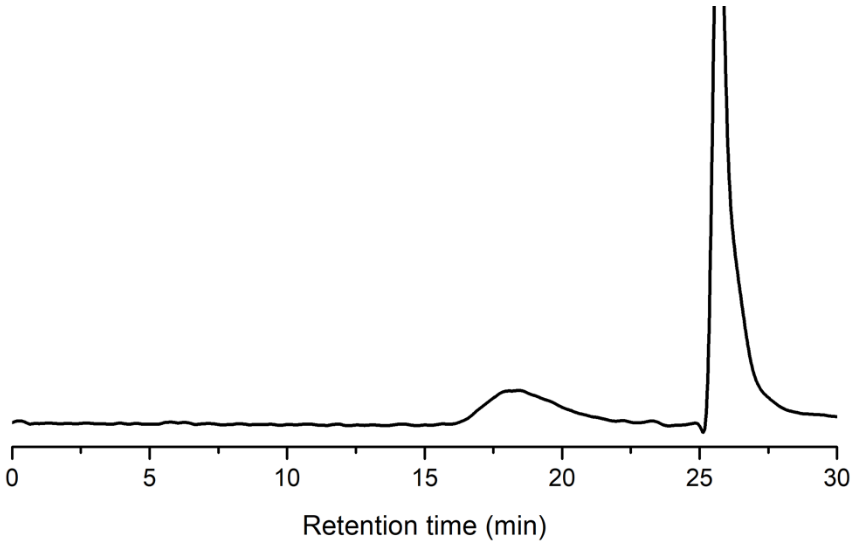

Fig. S128: SEC ($CHCl_3$) trace of **P34**.

High-Resolution Mass Spectrometric Data (HRMS data):

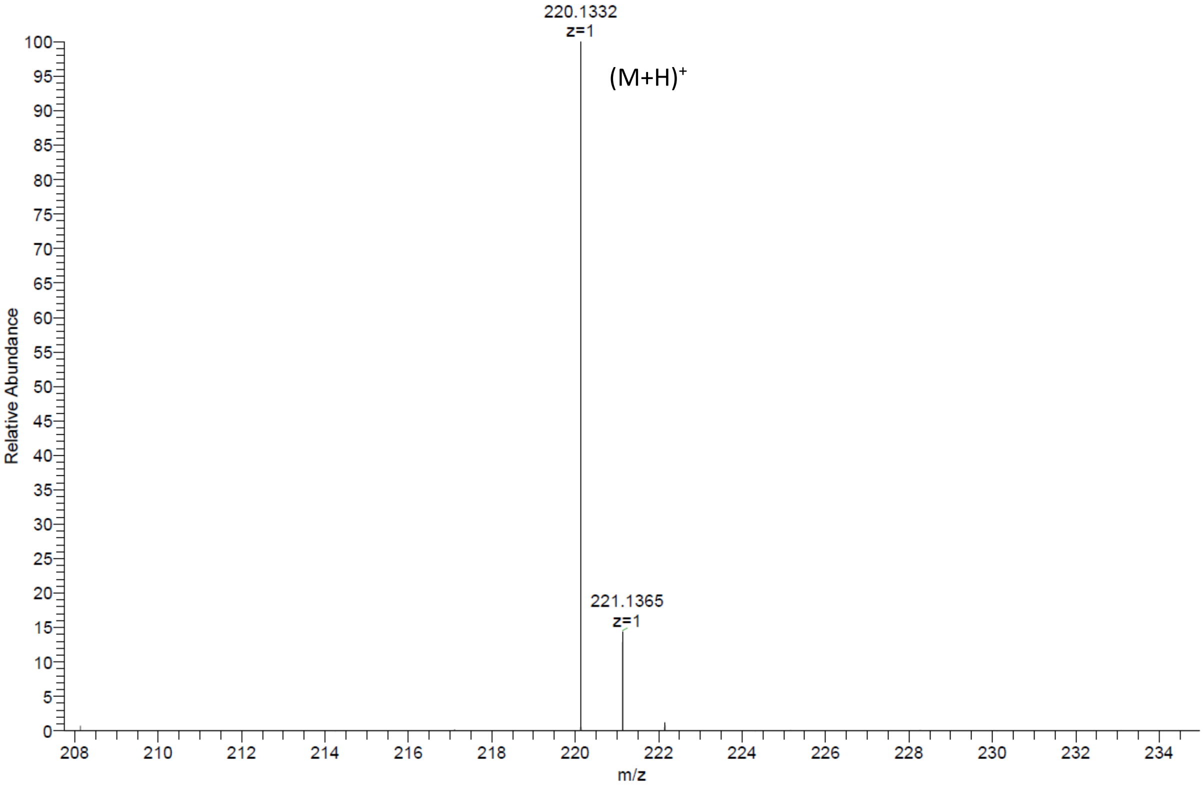

Fig. S129: HR-MS spectrum of **CTA7**.

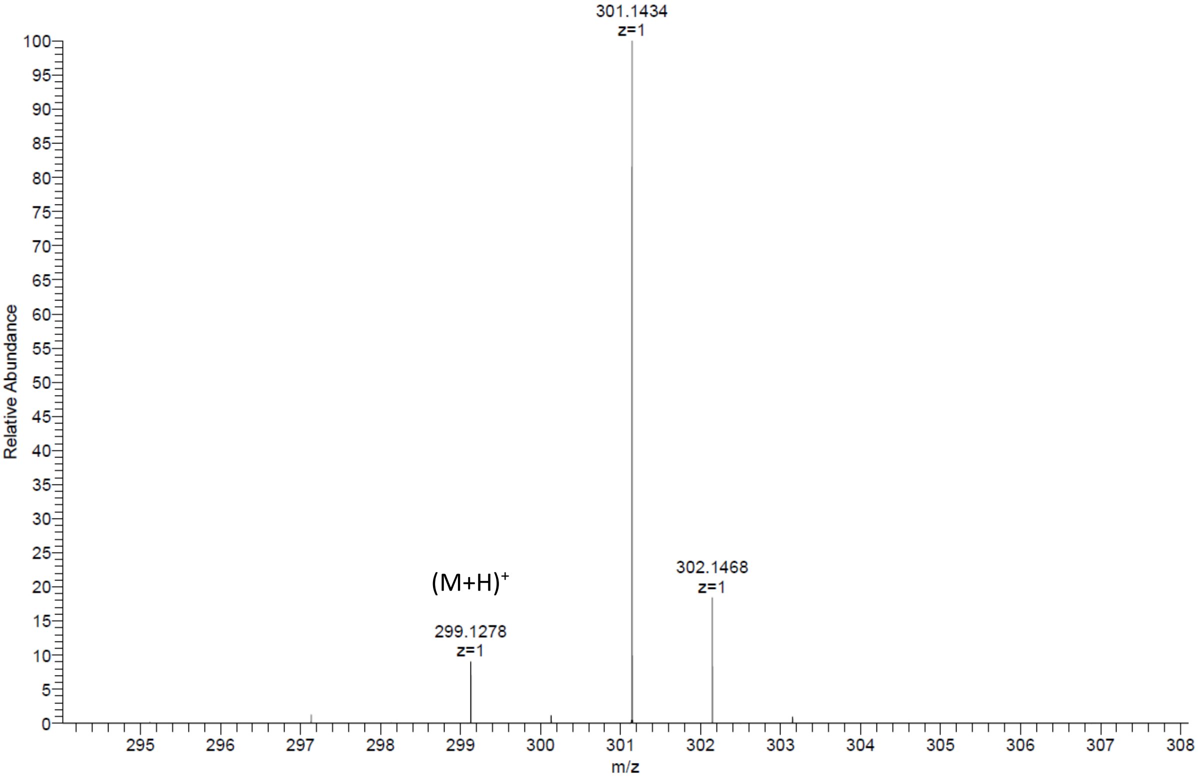

Fig. S130: HR-MS spectrum of **CTA8**.

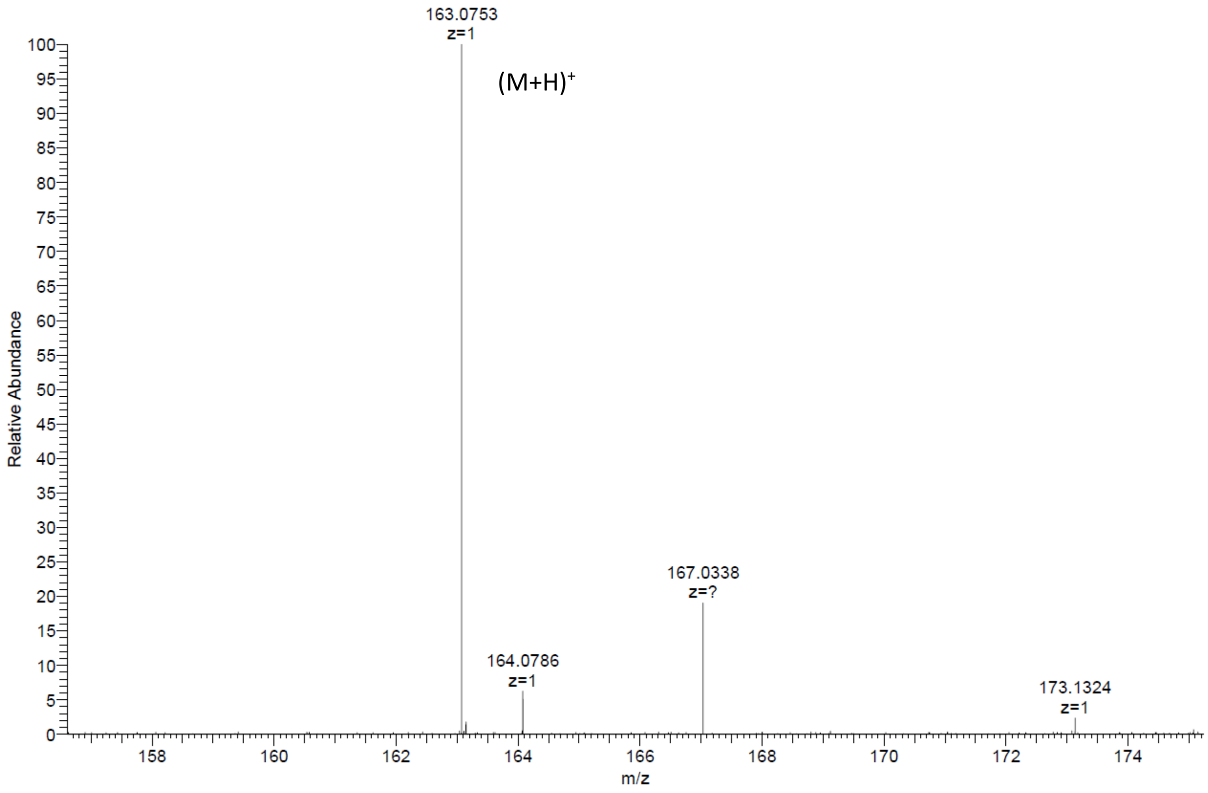

Fig. S131: HR-MS spectrum of **CTA9**.

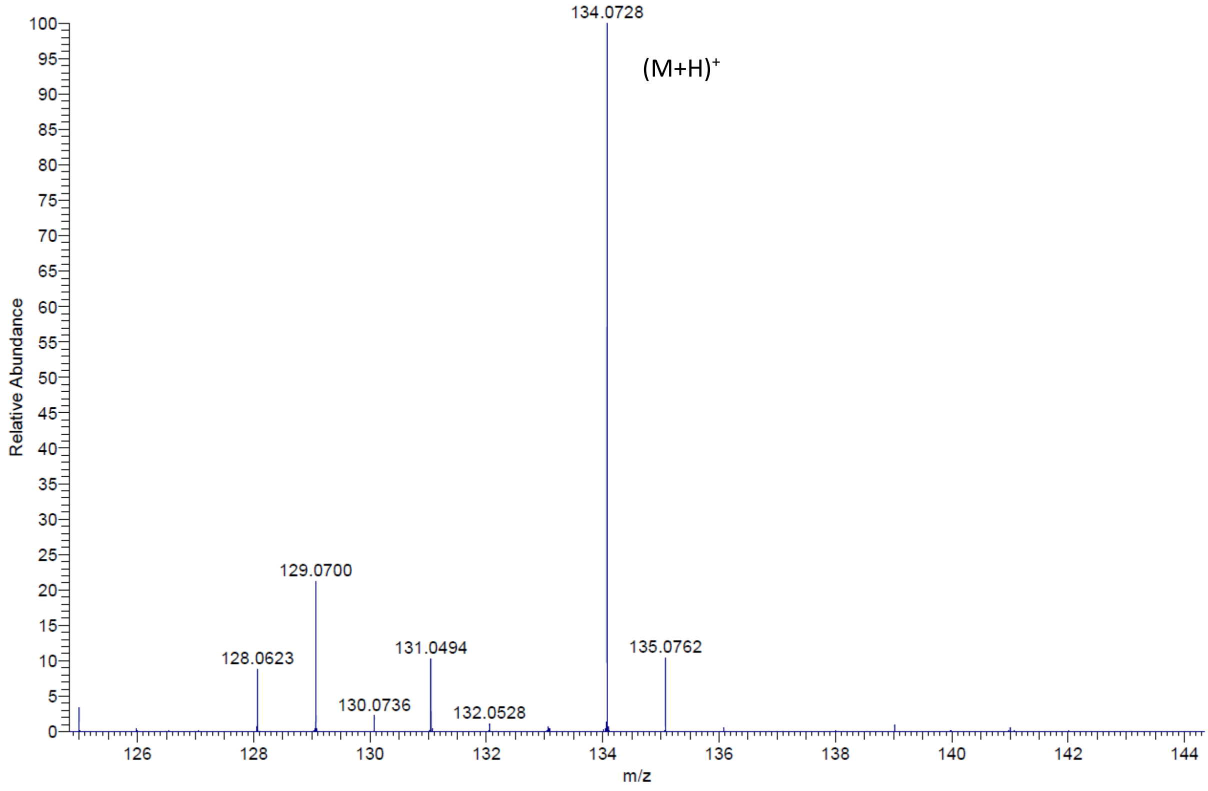

Fig. S132: HR-MS spectrum of **CTA10**.

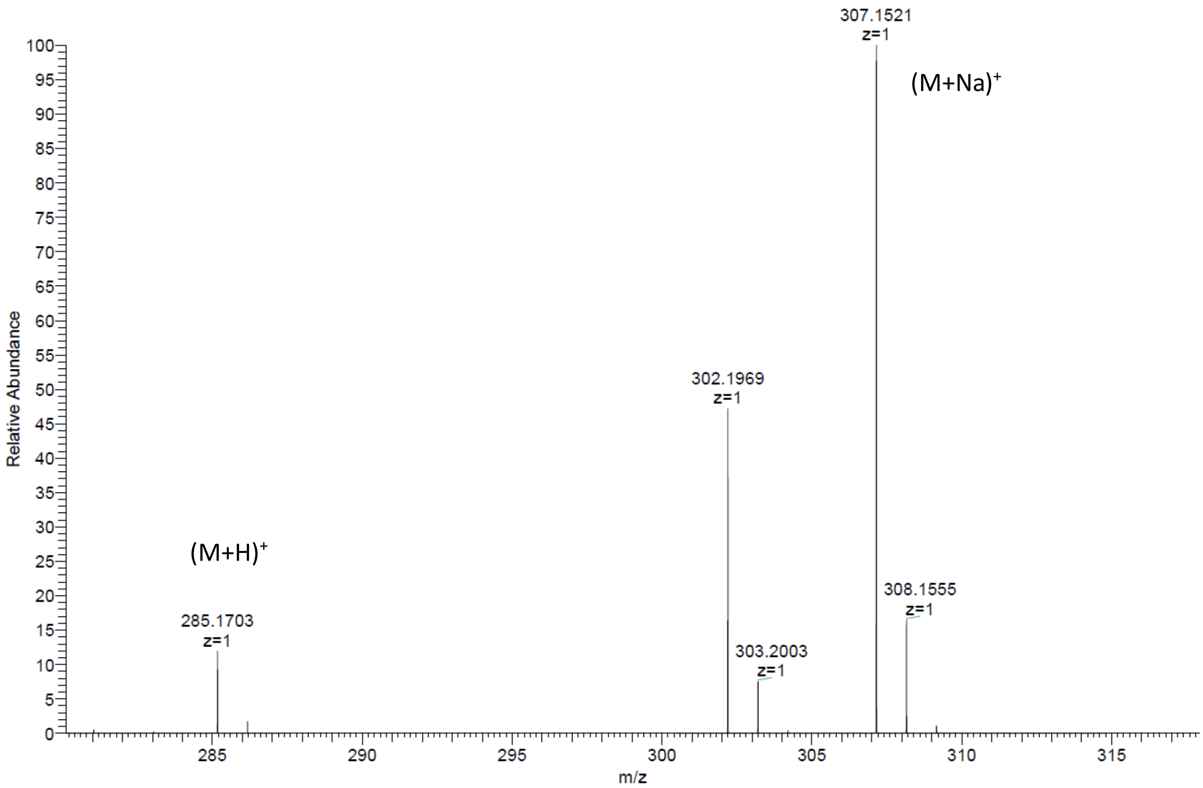

Fig. S133: HR-MS spectrum of **M3**.

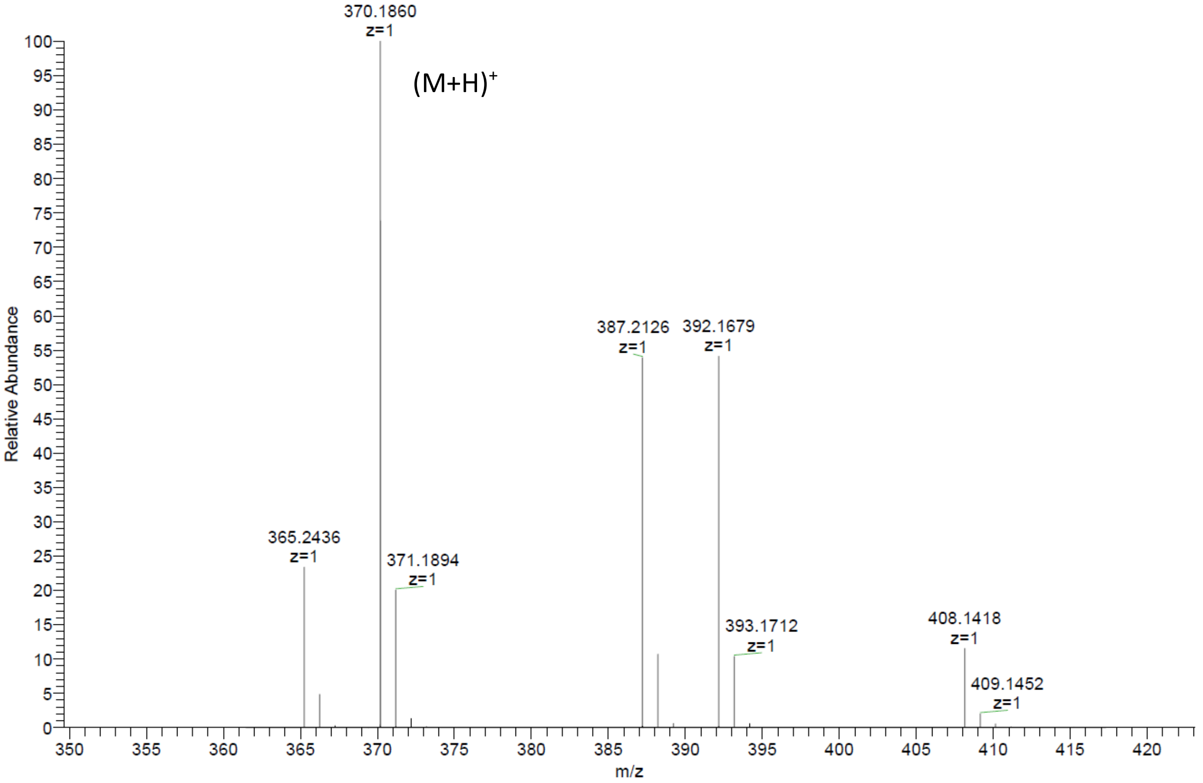

Fig. S134: HR-MS spectrum of **M4.**

References:

[1] Love, J. A.; Morgan, J. P.; Trnka, T. M.; Grubbs, R. H. A Practical and Highly Active Ruthenium-Based Catalyst That Effects the Cross Metathesis of Acrylonitrile. *Angew. Chemie - Int. Ed.* **2002**, *41*, 4035–4037.

[2] Klauck, F. J. R.; Yoon, H.; James, M. J.; Lautens, M.; Glorius, F. Visible-Light-Mediated Deaminative Three-Component Dicarbofunctionalization of Styrenes with Benzylic Radicals. *ACS Catal.* **2019**, *9*, 236–241.

[3] Liu, P.; Yasir, M.; Kilbinger, A. F. M. Catalytic Living Ring Opening Metathesis Polymerisation: The Importance of Ring Strain in Chain Transfer Agents. *Angew. Chemie - Int. Ed.* **2019**, *58*, 15278–15282.

[4] Lienkamp, K.; Madkour, A. E.; Musante, A.; Nelson, C. F.; Nüsslein, K.; Tew, G. N. Antimicrobial Polymers Prepared by ROMP with Unprecedented Selectivity: A Molecular Construction Kit Approach. *J. Am. Chem. Soc.* **2008**, *130*, 9836–9843.

[5] Mantovani, G.; Lecolley, F.; Tao, L.; Haddleton, D. M.; Clerx, J.; Cornelissen, J. J. L. M.; Velonia, K. Design and Synthesis of N -Maleimido-Functionalized Hydrophilic Polymers via Copper-Mediated Living Radical Polymerization: A Suitable Alternative to PEGylation Chemistry. *J. Am. Chem. Soc.* **2005**, *127*, 2966–2973.

[6] Mayo, F. R. Chain Transfer in the Polymerization of Styrene: The Reaction of Solvents with Free Radicals. *J.Am. Chem. Soc.* **1943**, *65* (12), 2324–2329.

[7] Hyatt, M. G.; Guironnet, D. Silane as Chain Transfer Agent for the Polymerization of Ethylene Catalyzed by a Palladium(II) Diimine Catalyst. *ACS Catal.* **2017**, *7* (9), 5717–5720.
